# Enantioselective Synthesis of Spirocyclic Isoxazolones Using a Conia-Ene Type Reaction

**DOI:** 10.1021/acs.joc.4c02921

**Published:** 2025-03-05

**Authors:** Martin Kamlar, Salil Putatunda, Ivana Císařová, Jan Veselý

**Affiliations:** † Department of Organic Chemistry, Faculty of Science, Charles University, Hlavova 2030, 128 43 Praha 2, Czech Republic; ‡ Department of Inorganic Chemistry, Faculty of Science, Charles University, Hlavova 2030, 128 43 Praha 2, Czech Republic

## Abstract

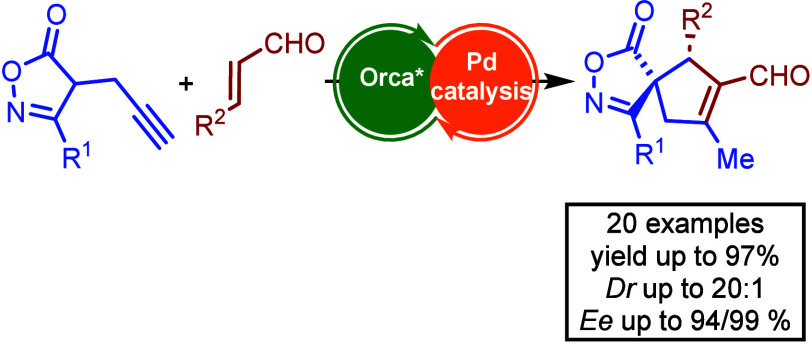

Stereoselective synthesis of spirocyclic compounds containing heterocyclic motifs represents a formidable challenge in enantioselective synthesis. Here, we present a cascade reaction between α,β-unsaturated aldehydes and isoxazolones under synergistic catalysis of a chiral secondary amine and a palladium(0) catalyst. This strategy allows access to chiral spiroisoxazolone derivatives with a large substrate scope tolerance and high levels of diastereoselectivity (dr up to 20:1) and enantioselectivity (up to 99% ee). Furthermore, the utility of this methodology is showcased by the transformation of chiral spiroisoxazolones into structurally attractive and enantiomerically enriched cyclopentene carboxylic acids with two stereogenic centers.

## Introduction

**Scheme 1:**
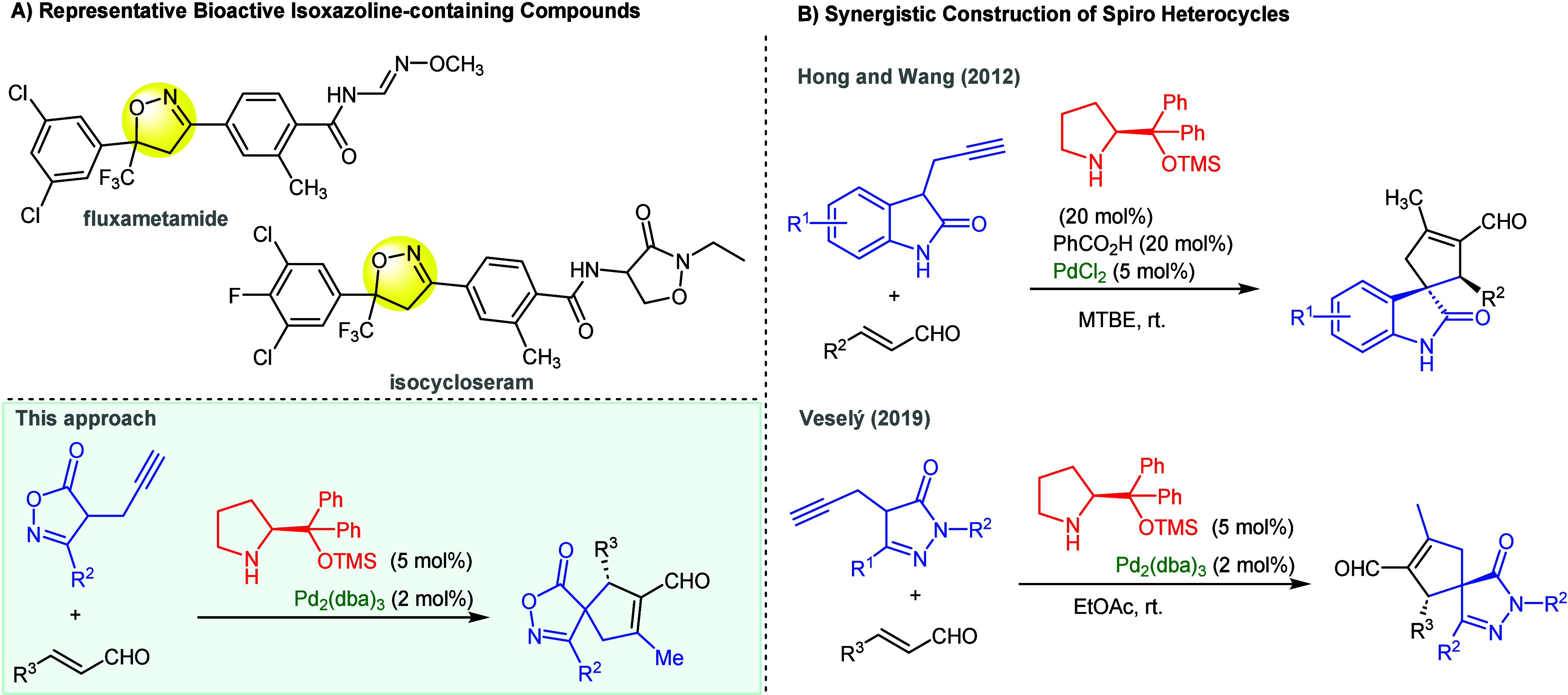
(A) Bioactive Isoxazoline-Containing Compounds and (B) Synergistic Catalysis in the Construction of Spiro Heterocycles via the Conia-Ene Reaction

Isoxazolines are versatile five-membered heterocyclic scaffolds with significant biological activities that also serve as important precursors in natural product synthesis to access amino acids^1^ or hydroxy ketones.^2^ Moreover, isoxazoline-based molecules have been explored for their anti-inflammatory,^3^ antibacterial,^4^ antifungal,^5^ and anticancer^6^ activities. Some compounds with the isoxazoline moiety, such as fluxametamide and isocycloseram, are used as agrochemicals for crop protection (Scheme 1A).^7^ Thus, the incorporation of an isoxazoline moiety as a bioactive framework into highly complex organic compounds can be attractive from the perspective of medicinal chemistry. It is noteworthy that spirocycles with increased rigidity are popular in medicinal chemistry today, resulting in various approved drugs and drug candidates. A combination of spatial rigidity caused by the presence of spirocyclic carbon centers and a heterocyclic moiety often leads to improvements in physicochemical properties and pharmacokinetic profiles.^8^ Therefore, enantioselective synthesis of spiro (hetero)compounds represents one of the most challenging tasks in synthetic organic chemistry.^9^ Among other strategies, synergistic catalysis, which combines chiral organocatalysis with transition metal catalysis, has established itself as a suitable toolbox for synthesizing these molecules, which, in general, are challenging to prepare via a single catalytic mode.^10^ The most widely used strategy includes either tetrahydro-1*H*-oxepino ring expansion^11^ or vinylcyclopropane ring contraction^12^ of the corresponding heterocycle under transition metal catalyst activation, forming a corresponding zwitterionic species, which subsequently reacts with the iminium/enamine intermediate formed from enal under chiral secondary amine catalyst activation.

Despite the remarkable significance of the catalytic Conia-ene reaction in total synthesis under transition metal catalysis,^13^ approaches employing an organocatalytic Conia-ene reaction in the synthesis of enantiopure spiro heterocycles are considerably less explored.^14^ Pioneering work employing synergistic catalysis of homogeneous Pd(II) complexes and secondary amines in the enantioselective synthesis of spirocyclopentenes with an oxindole moiety was introduced by Hong and Wang (Scheme 1B).^14e^ Soon thereafter, Córdova and Johnston reported a concept using a combined heterogeneous Pd(0)/chiral amine relay catalysis.^13d^ Besides oxindole, pyrazolone, another nitrogen-containing heterocycle, has been successfully used to construct chiral spiropyrazolones. In 2016, Enders’s research group developed a sequential organocatalytic and silver catalytic approach,^14c^ and a few years later, our research group published the synthesis of spiropyrazolones using a synergistic Pd(0)/chiral secondary amine-promoted Conia-ene reaction (Scheme 1B).^14b^ Conversely, the enantioselective synthesis of spirocyclic compounds bearing an isoxazolone moiety has not yet been investigated under the catalytic Conia-ene reaction. With this regard and based on our continuous effort in the area of stereoselective synthesis,^11a,11c,11e,12a,12b,14b^ we speculated that a propargyl-substituted isoxazolone derivative could under activation with a transition metal catalyst undergo a cyclization domino reaction with α,β-unsaturated aldehydes activated by a chiral secondary amine catalyst providing access to optically pure spiro isoxazolone derivatives (Scheme 1B).

## Results and Discussion

To probe the viability of this project, we commenced our investigation with the reaction between 3-phenyl-4-propargyl isoxazolone derivative **1a** and cinnamaldehyde **2a** in acetonitrile as a solvent under catalysis with a combination of tris(dibenzylideneacetone)dipalladium and a Hayashi–Jørgensen secondary amine catalyst (Table 1, entry 1). To our delight, the reaction provided the corresponding spiroisoxazolone derivative **3a** as a separable mixture of two diastereoisomers (dr 2.4:1) in good yield. The major diastereoisomer was isolated in 50% yield (20% yield of the minor one) with an enantiomeric purity of 48% ee for the major diastereoisomer and 98% ee for the minor diastereoisomer. Prompted by this result, a small group of organic solvents have been studied in order to improve the stereocontrol of the studied transformation (Table 1, entries 2–5). The obtained results clearly indicated that diastereocontrol of the reaction remained unchanged, but enantiocontrol was significantly improved in all cases, especially for the major diastereoisomer. Ethyl acetate, as the most effective solvent, provided **3a** in a high yield of 63% for the major diastereoisomer and 30% for the minor diastereoisomer. Moreover, a high level of enantiomeric purity for both diastereoisomers (88% ee for the major diastereoisomer and 99% ee for the minor diastereoisomer, entry 5) was observed. Next, the effects of other transition metals and organocatalysts were studied (Table 1, entries 6–15). First, the role of a chiral organocatalyst was investigated, and apart from the Hayashi–Jørgensen secondary amine, other chiral secondary amines in combination with Pd_2_(dba)_3_ were tested. The obtained experimental data showed that the expected cyclization was also observed in the presence of other catalysts derived from diphenyl prolinols **II–V**.

**Table 1:**
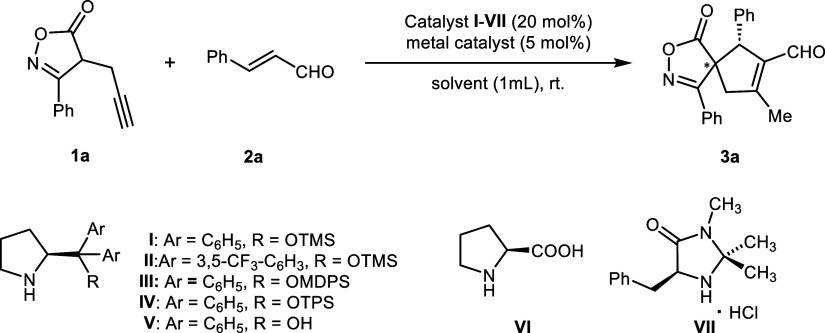
Optimization of the Reaction Conditions between Isoxazolone **1a** and Cinnamaldehyde **2a**^a^

entry	organocatalyst	metal catalyst	solvent	time (h)	conversion (%)	yield of the major/minor diasteroisomer (%)	dr^b^	ee of the major/minor diasteroisomer (%)^c^
1	**I**	Pd_2_(dba)_3_	MeCN	24	100	50/20	2.4:1	48/98
2	**I**	Pd_2_(dba)_3_	toluene	24	100	60/30	2:1	80/99
3	**I**	Pd_2_(dba)_3_	DCM	24	100	35/25	2.5:1	78/98
4	**I**	Pd_2_(dba)_3_	MTBE	24	100	40/18	1.3:1	68/99
5	**I**	Pd_2_(dba)_3_	EtOAc	24	100	63/30	2:1	88/99
6	**II**	Pd_2_(dba)_3_	EtOAc	72	100	24/12	2:1	56/92
7	**III**	Pd_2_(dba)_3_	EtOAc	120	100	45/39	1:1	64/98
8	**IV**	Pd_2_(dba)_3_	EtOAc	120	100	39/33	1:1	72/98
9	**V**	Pd_2_(dba)_3_	EtOAc	120	30	15/nd	2.5:1	18/–
10	**VI**	Pd_2_(dba)_3_	EtOAc	120	>10	nd/nd	–	–/–
11	**VII**	Pd_2_(dba)_3_	EtOAc	120	>10	nd/nd	–	–/–
12	**I**	Pd(PPh_3_)_4_	EtOAc	120	0	nd/nd	–	–/–
13	**I**	PdCl_2_	EtOAc	120	0	nd/nd	–	–/–
14	**I**	Pd(OAc)_2_	EtOAc	72	100	38/26	2:1	75/96
15	**I**	AuCl	EtOAc	120	0	nd/nd	–	–/–
16^d^	**I**	Pd_2_(dba)_3_	EtOAc	120	90	43/25	2:1	80/99
17^e^	**I**	Pd_2_(dba)_3_	EtOAc	120	0	nd/nd	–	–/–
18^f^	**I**	Pd_2_(dba)_3_	EtOAc	120	96	42/27	1.6:1	82/99

a Reaction conditions: catalyst **I**–**VII** (0.024 mmol, 20 mol %), enal **2a** (0.12 mmol, 1.0 equiv), isoxazolone **1a** (0.18 mmol, 1.5 equiv), and a solvent (1 mL) at rt.

b Determined by ^1^H NMR.

c Determined by chiral HPLC.

d With 2 mol % Pd_2_(dba)_3_.

e With 2 mol % Pd_2_(dba)_3_ and 5 mol % **I**.

f Reaction carried out at 0 °C.

However, the reaction carried out in the presence of other silyl-protected diphenyl prolinol catalysts showed a lower level of stereoselectivity (entries 6–8). The reaction catalyzed with non-protected diphenyl prolinol catalyst **V** provided spirocycle **3a** in a low yield and stereocontrol (entry 9). It is noteworthy that (*S*)-proline **VI** and imidazolidinone **VII** were found to be inefficient (entries 10 and 11, respectively). To probe the compatibility with model Hayashi–Jørgensen secondary amine catalyst **I**, a small set of achiral transition metal salts has been examined (Table 1, entries 12–15). We observed that not only Pd_2_(dba)_3_ but also Pd(OAc)_2_ was effective together with a chiral Hayashi–Jørgensen secondary amine catalyst. However, when Pd(OAc)_2_ was used, **3a** was isolated in moderate yield and enantiomeric purity for the major diastereoisomer (entry 14). Surprisingly, gold(I) chloride, known as an effective species in activating triple bonds in various transformations,^15^ was futile in our reaction. Next, the role of catalytic loading in the course of the reaction was tested (Table 1, entries 16 and 17). The obtained data clearly indicate that the decreased loading of both organocatalysts and transition metal catalysts has a negative effect on the course of the reaction. This resulted in the complete ceasing of the transformation when 2 mol % Pd_2_(dba)_3_ and 5 mol % organocatalyst **I** were used. We also demonstrated that a reaction carried out at 0 °C did not provide product **3a** with better stereoselectivity (entry 18).

**Scheme 2:**
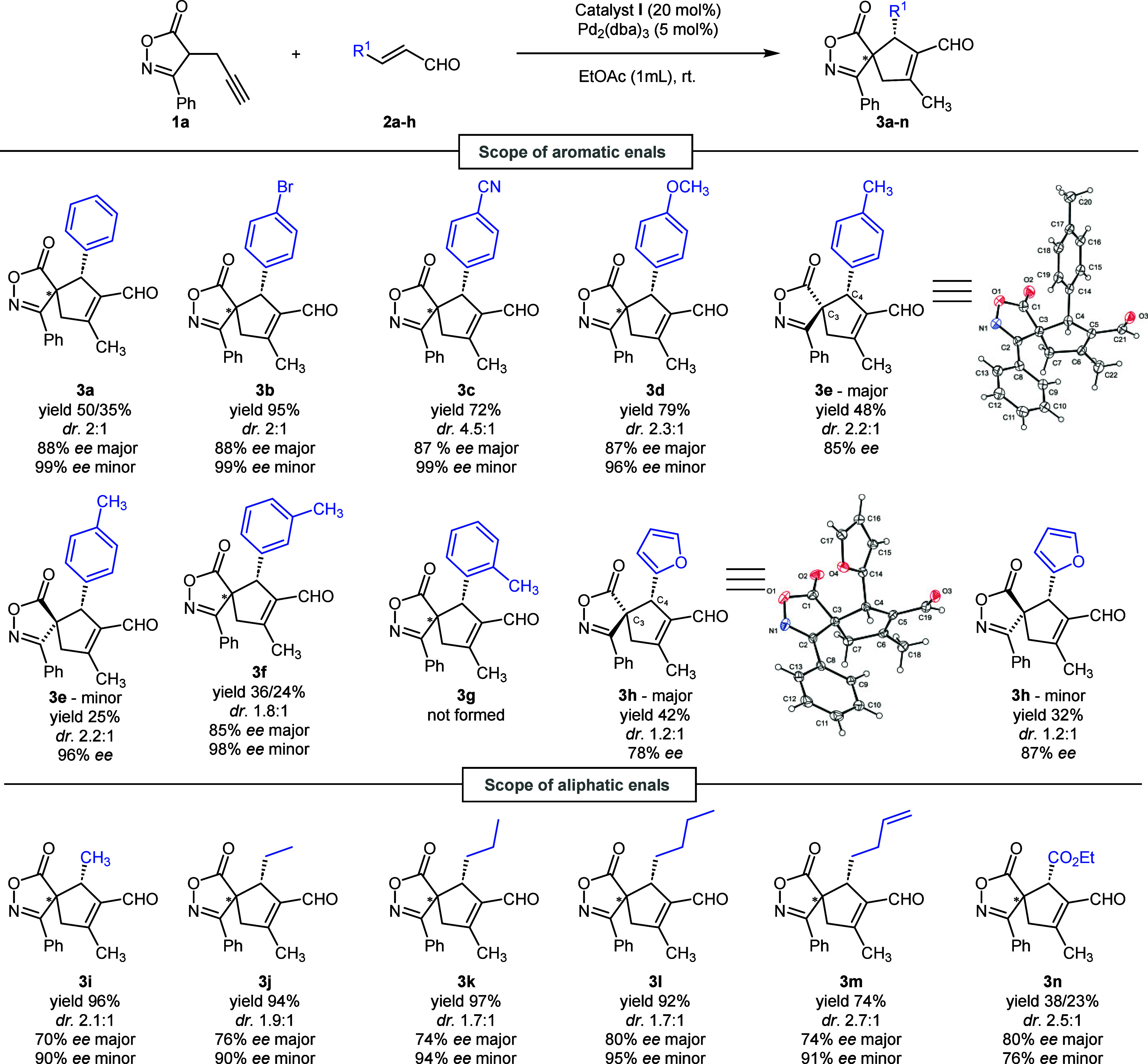
Reaction Scope of α,β-Unsaturated Aldehyde Derivatives **2a**–**n** Reaction conditions: catalyst **I** (0.024 mmol, 20 mol %), aldehyde **2a**–**h** (0.12 mmol, 1.0 equiv), isoxazolone **1a** (0.18 mmol, 1.5 equiv), and EtOAc (1 mL) at rt.

With our optimized conditions in hand, the scope of the method was probed using different types of substrates. First, we examined the effect of substitution on the aromatic ring of cinnamaldehyde in the reaction with the isoxazolone derivative (**1a**) (Scheme 2). When *para*-brominated cinnamaldehyde (**2b**) was used with isoxazolone **1a**, spirocycle **3b** was delivered as a mixture of diastereoisomers in high yield (95%) and with a high level of enantiomeric purity for the major (88% ee) and minor (99% ee) diastereoisomers. On the other hand, the reaction proceeded with only a low diastereoselectivity (dr 2:1). An increased level of diastereocontrol of the spirocyclization reaction (dr 4.5:1) was observed using *p*-cyano-substituted cinnamaldehyde **2c**. Spiroisoxazolone derivative **3c** was isolated in 72% yield as an inseparable mixture of diastereoisomers with a high level of enantiomeric purity for both diastereoisomers (87% and 99% ee for the major and minor enantiomers, respectively). Electron-donating methoxy and methyl groups in the *para* position of cinnamaldehyde were also well tolerated. Selectivities for both reactions were comparable (dr 2.3:1 to 2.2:1 and 87% and 96% ee to 85% and 96% ee for **3d** and **3e**, respectively), although the reactivities of both aldehydes were different. Whereas a full conversion of *p*-methoxy-substituted cinnamaldehyde (**2d**) was reached after 6 days, *p*-methyl-substituted cinnamaldehyde (**2e**) delivered the desired spiroisoxazolene **3e** after 3 days as a separable mixture of two diastereoisomers (48% and 25% for the major and minor enantiomers, respectively). Most importantly, a major diastereoisomer of **3e** was obtained as a crystalline material suitable for X-ray diffraction analysis, leading to the conclusion that the absolute configurations on the stereogenic centers were (*S*)-C3 and (*R*)-C4. Additionally, *m*-methyl-substituted cinnamaldehyde on the aromatic ring (**2f**) was successfully tested, affording spiroisoxazolene derivative **3f** as a separable mixture of two diastereoisomers with lower yields of 36% for the major diastereoisomer and 24% for the minor diastereoisomer, with high enantiomeric purities for both diastereoisomers (85% and 98% ee, respectively). Conversely, *o*-methyl-substituted cinnamaldehyde **1g** did not lead to the formation of spirocycle **3g** even after a prolonged reaction, probably due to steric hindrance of the methyl group in the *ortho* position on the aromatic ring. We also successfully tested an α,β-unsaturated aldehyde bearing a heteroaromatic furyl unit (**2h**). Product **3h** was delivered after 7 days as a separable mixture of diastereoisomers (42% and 32%) with a good level of enantiomeric purity for both diastereoisomers (78% and 87% ee). Again, the major diastereoisomer of **3h** was obtained as a single crystal suitable for X-ray analysis, confirming the same absolute configuration on both carbon stereogenic centers.

**Scheme 3:**
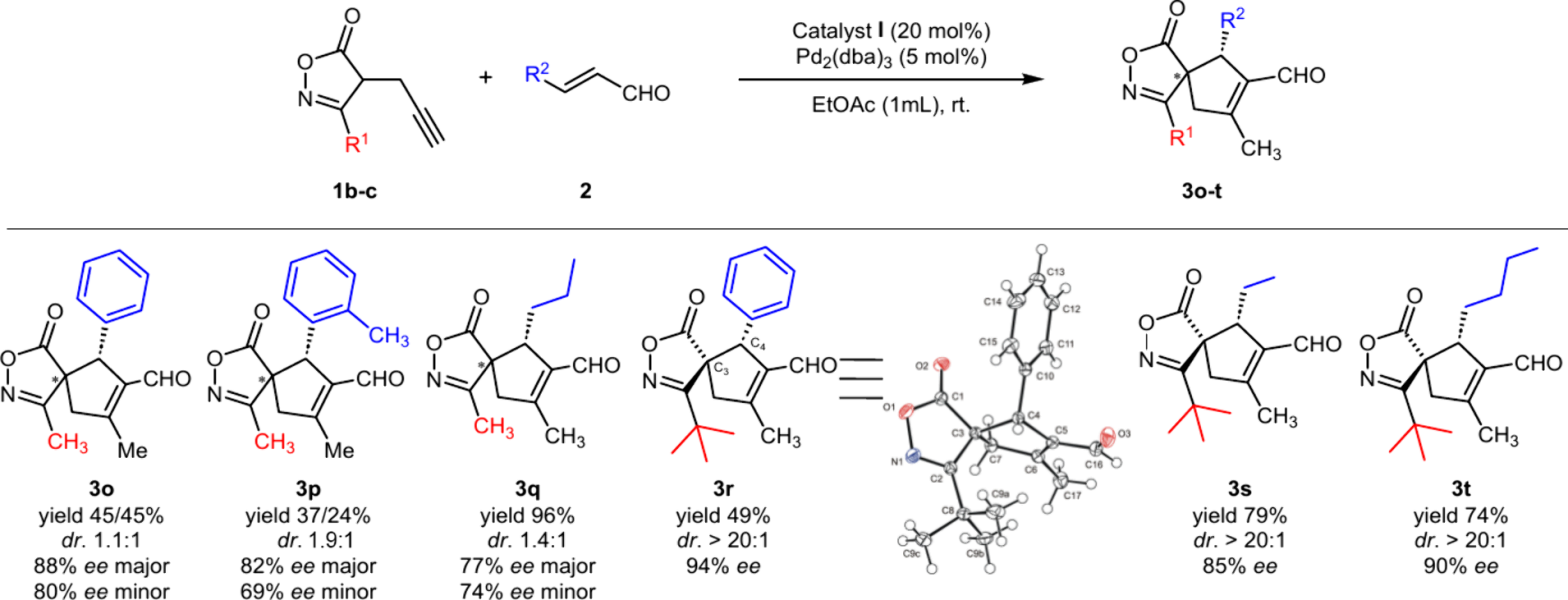
Reaction Scope of 3-Aliphatic-Substituted Isoxazolone Derivatives **1b** and **1c** Reaction conditions: catalyst **I** (0.024 mmol, 20 mol %), aldehyde **2** (0.12 mmol, 1.0 equiv), isoxazolone **1a**–**c** (0.18 mmol, 1.5 equiv), and EtOAc (1 mL) at rt.

Next, we turned our attention to isoxazolones substituted with an R^1^ alkyl group at position 3 (Scheme 3). When 3-methyl-4-propargyl isoxazolone (**1b**) was employed in the cyclization reaction with either aromatic or aliphatic α,β-unsaturated aldehydes (**2a**, **2g**, and **2k**), stereocontrol of the corresponding reactions was low. For example, the reaction between isoxazolone **1b** and aldehyde **2k** led to spirocycle **3q** with a low level of diastereoselectivity (dr 1.4:1) with enantioselectivities of 77% ee for the major diastereoisomer and 74% ee for the minor diastereoisomer. To improve diastereocontrol of the spirocyclization reaction, the sterically demanding 3-*tert*-butyl-4-propargyl isoxazolone (**1c**) was prepared and used in the cyclization reaction with cinnamic aldehyde **2a**. Increased steric hindrance resulted in a slow reaction course reaching full conversion after 7 days. On the other hand, excellent diastereoselectivity of the transformation (dr >20:1) led to the formation of product **3r** as a single diastereoisomer with a high level of enantiomeric purity (94% ee) in 49% yield. The absolute configuration of spirocycle **3r** was confirmed by X-ray diffraction analysis, having the configuration on both carbon stereogenic centers (3*S* and 4*R*) identical to the previously determined configurations on the major diastereoisomers of **3f** and **3h**, respectively. To bring the reactivity forward, cyclization reactions of isoxazolone **1c** with the sterically less demanding aliphatic *trans*-2-pentenal (**2i**) and *trans*-2-heptenal (**2k**) were carried out. To our delight, full conversion was reached in both cases after 24 h, and both reactions proceeded with a high level of diastereoselectivity (dr >20:1). Products **3s** and **3t** were isolated in good yields (79% for **3s** and 74% for **3t**) with a high level of enantiomeric excess (85% ee for **3s** and 90% ee for **3t**).

**Scheme 4:**
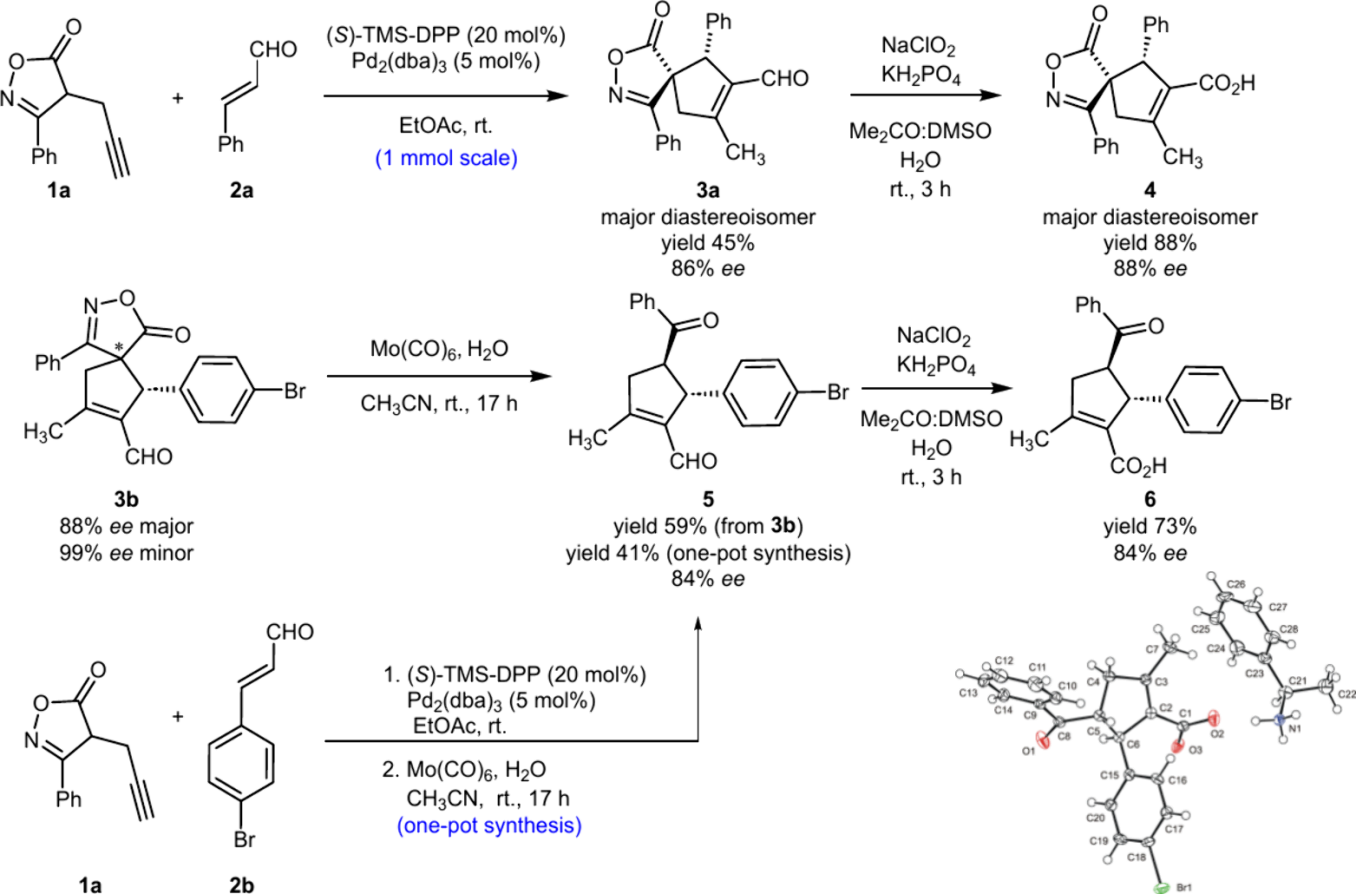
Subsequent Transformation of Spiroisoxazolone Derivatives **3a** and **3b**

The robustness and versatility of the derivatives prepared as described above were demonstrated in the following transformations on model derivative **3a** and bromo derivative **3b** (Scheme 4). First, a scale-up experiment was carried out using 1 mmol of cinnamaldehyde (**2a**) and 1.5 mmol of isoxazolone **1a**. Spirocycle **3a** was delivered as a separable mixture of two diastereoisomers in 45% yield for the major diastereoisomer and 27% yield for the minor diastereoisomer with a retained level of stereoselectivity (dr 2:1, 86% and 99% ee for the major and minor enantiomers, respectively) compared to the model reaction. Next, we showed that the aldehyde moiety in the major diastereoisomer of spiroisoxazolone derivative **3a** could be readily converted into the carboxylic group by the formation of derivative **4** in a high yield of 88% with an enantiomeric excess of 88% ee. We also demonstrated that the isoxazolone moiety in derivative **3b** can be cleaved in the presence of Mo(CO)_6_, forming compound **5** as a single diastereoisomer in 59% yield with an enantiomeric purity of 84% ee. We also showed that compound **5** can be obtained directly from starting isoxazolone derivative **1a** and enal **2b** using a one-pot synthetic procedure, including enantioselective spirocyclization/isoxazolone cleavage, in 41% yield with the same enantiomeric purity (84% ee). The aldehyde moiety of **5** was then successfully oxidized using Pinnick type oxidation, affording carboxylic acid **6** in 73% yield with retained enantiomeric excess (84% ee). The absolute configuration of **6** was then assigned on both stereogenic centers as 4*R*,5*R* after its co-crystallization with (*R*)-1-phenylethylamine.

**Scheme 5:**
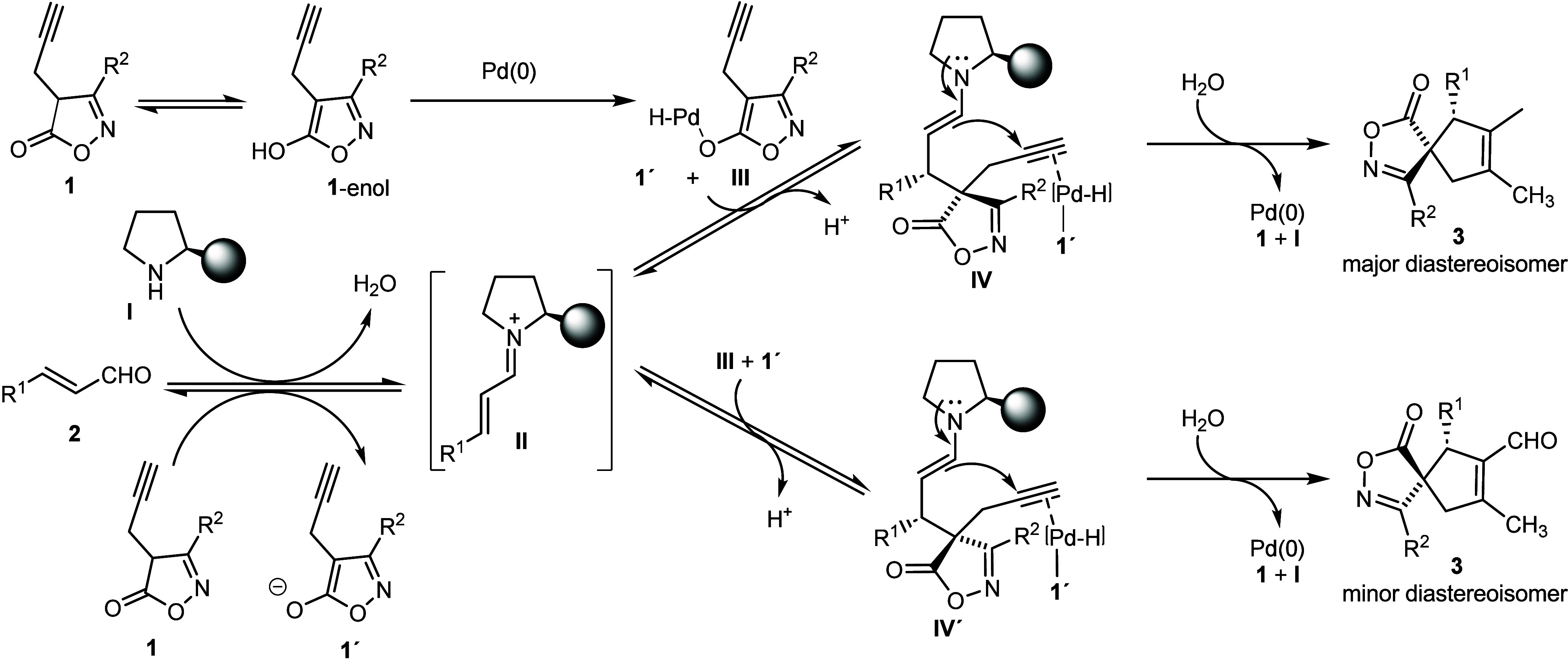
Proposed Combined Iminium/Enamine and Pd-Catalyzed Michael/Carbocyclization between Isoxazolone **1** and Enal **2**

Based on our previous report about the computational and mechanistic study of the Conia-ene reaction,^14b^ we proposed the reaction mechanism and key intermediates of the spirocyclization reaction of isoxazolones **1** with enals **2** to rationalize the stereochemistry of the cascade catalytic reaction (Scheme 5). We assume that the Pd(0) catalyst forms Pd(II) hydride species **III** with the enol form of isoxazolone **1** via oxidative addition.^13h−13j^ Next, intermediate **III** undergoes conjugated addition with chiral iminium intermediate **II**, resulting in chiral enamine **IV**. After carbocyclization of **IV**, spiroheterocycle **3** is released from the catalytic cycle together with recycled chiral organocatalyst **I** and Pd(0).

## Conclusions

In summary, we have developed a stereoselective approach to enantiomerically enriched spiroisoxazolone derivatives using easily accessible α,β-unsaturated aldehydes and propargyl-substituted isoxazolones. This cascade Conia-ene reaction effectively catalyzed by the synergy of chiral organocatalysis and tris(dibenzylideneacetone)dipalladium(0) exhibits high versatility and provides a wide array of spiroisoxazolones in good to high yields (36–97%) with high levels of diastereoselectivity (dr up to 20:1) and high enantioselectivities (ee up to 94% and 99% for the major and minor diastereoisomers, respectively). Furthermore, the synthetic utility was demonstrated on selected follow-up transformations, leading to enantiomerically enriched functionalized products.

## Experimental Section

### General Information

Chemicals and solvents were either purchased (puriss p.A.) from commercial suppliers or purified by standard techniques. For thin-layer chromatography (TLC), silica gel plates Merck 60 F_254_ were used, and compounds were visualized by irradiation with UV light and/or by treatment with a solution of phosphomolybdenic acid (25 g), Ce(SO_4_)_2_·H_2_O (10 g), concentrated H_2_SO_4_ (60 mL), and H_2_O (940 mL) followed by heating. Column chromatography was performed using silica gel Merck 60 (particle size of 0.040–0.063 mm). ^1^H NMR, ^13^C NMR, and 2D NMR spectra were recorded with a Bruker DPX600 NMR instrument. Chemical shifts (δ) are reported in parts per million relative to residual solvent signals (CHCl_3_, 7.26 ppm for ^1^H NMR; CDCl_3_, 77.1 ppm for ^13^C NMR). High-resolution mass spectra were recorded on an LCQ Fleet spectrometer using a Bruker Compact QTOF-MS instrument controlled by Compass 1.9 Control software to measure the ESI high-resolution mass spectra. The monoisotopic mass values were calculated using Data analysis software version 4.4. The analysis was conducted in positive ion mode over a scan range from 50 to 1000, and nitrogen was used as a nebulizer gas at a pressure of 4 psi and a flow of 3 L/min for the dry gas. The capillary voltage and temperature were set at 4500 V and 220 °C, respectively. Optical rotations were performed on an AU-Tomatica polarimeter; Autopol III. IR DRIFT spectra were recorded with a Nicolet AVATAR 370 FT-IR instrument in cm^−1^ instrument. HPLC analysis was performed on a LC20AD Shimadzu liquid chromatograph with an SPD-M20A diode array detector with a Daicel Chiralpak column.

Isoxazolones **1a** and **1b** are known compounds prepared according to the published methods.^16^ The spectral data of **1a** and **1b** are consistent with data published in the literature.^16b,17^

### 3-(*tert*-Butyl)-4-(prop-2-yn-1-yl)isoxazol-5(4*H*)-one (**1c**)

The title compound was prepared according to the same two-step procedure.^16^ The crude product was purified by silica gel flash chromatography with *n*-hexane/ethyl acetate (2:1) as the eluent to give **1c** as a pale red solid in 21% yield (0.9 g): ^1^H NMR (600 MHz, CDCl_3_) δ 3.51 (t, *J* = 4.7 Hz, 1H), 2.91 (qdd, *J* = 17.4 Hz, *J′* = 4.7 Hz, *J″* = 2.6 Hz, 2H), 2.12 (t, *J* = 2.6 Hz, 1H), 1.32 (s, 9H); ^13^C{^1^H} NMR (151 MHz, CDCl_3_) δ 177.4, 173.8, 77.2, 73.0, 45.4, 35.5, 28.2, 19.5; HRMS (ESI) *m*/*z* calcd for C_10_H_13_NO_2_Na [M + Na]^+^ 202.0838, found 202.0839.

Enal **2a** is a commercially available compound, and enals **2b**–**n** are known compounds, which were prepared and characterized according to the published methods.^18^

### General Procedure for the Synthesis of Spiroisoxazolones **3a**–**t**

To a screw cap septum vial containing EtOAc (1 mL) was added catalyst (*S*)-DPP-TMS **I** (0.024 mmol, 0.20 equiv), followed by the addition of (*E*)-α,β-unsaturated aldehydes **2a**–**t** (0.12 mmol, 1.0 equiv) and isoxazolone derivatives **1a**–**c** (0.18 mmol, 1.5 equiv). Then, Pd_2_(dba)_3_ (0.006 mmol, 0.05 equiv) was added. After full conversion was reached (TLC monitoring), the solvent was removed *invacuo* and the crude reaction mixture was purified through silica gel flash chromatography (*n*-Hex/EtOAc), affording the desired spirocyclic compounds **3a**–**t**.

#### (5*S*,6*R*)-8-Methyl-1-oxo-4,6-diphenyl-2-oxa-3-azaspiro[4.4]nona-3,7-diene-7-carbaldehyde (**3a**) (major diastereoisomer)

Purification of crude (dr 2:1) by silica gel chromatography (2:1 *n*-Hex/EA): yellow semisolid (20 mg, 50% yield); ^1^H NMR (600 MHz, CDCl_3_) δ 10.07 (s, 1H), 7.73–7.72 (m, 2H), 7.59–7.56 (m, 1H), 7.52–7.49 (m, 2H), 7.29–7.26 (m, 3H), 7.02–6.98 (m, 2H), 4.99 (s, 1H), 3.33–3.25 (m, 2H), 2.42 (s, 3H); ^13^C{^1^H} NMR (151 MHz, CDCl_3_) δ 186.7, 178.4, 167.1, 159.4, 136.2, 135.1, 132.3, 129.7 (2C), 128.7 (2C), 128.5, 128.2 (2C), 126.9, 126.6 (2C), 60.0, 56.7, 47.6, 14.8; FT-IR (ATR) ν 3563, 3405, 3315, 3025, 2851, 1670, 1347, 1269, 1242, 1180 cm^–1^; HPLC analysis ee (major diastereoisomer) 88% (Daicel Chiralpak IA column, 90:10 heptane/*iso*-propanol, 1.0 mL/min, λ = 250 nm; retention times, *t*_major_ = 11.1 min, *t*_minor_ = 13.1 min) at 25 °C; [α]_D_^rt^ = −68.2° (*c* = 0.76, CHCl_3_); HRMS (ESI) *m*/*z* calcd for C_21_H_18_NO_3_ [M + H]^+^ 332.1281, found 332.1281.

#### (5*R*,6*R*)-8-Methyl-1-oxo-4,6-diphenyl-2-oxa-3-azaspiro[4.4]nona-3,7-diene-7-carbaldehyde (**3a**) (minor diastereoisomer)

Purification of crude (dr 2:1) by silica gel chromatography (2:1 *n*-Hex/EA): yellow semisolid (14 mg, 35% yield); ^1^H NMR (600 MHz, CDCl_3_) δ 10.05 (s, 1H), 7.34–7.32 (m, 1H), 7.20–7.17 (m, 2H), 7.14–7.12 (m, 2H), 7.01–6.97 (m, 1H), 6.94–6.92 (m, 2H), 6.68–6.67 (m, 2H), 5.00 (s, 1H), 3.43 (d, *J* = 19.2 Hz, 1H), 3.29 (d, *J* = 19.2 Hz, 1H), 2.45 (s, 3H); ^13^C{^1^H} NMR (151 MHz, CDCl_3_) δ 186.8, 182.4, 167.4, 158.3, 136.6, 134.7, 131.3, 128.5 (2C), 128.2 (2C), 128.0 (2C), 127.9, 126.9 (3C), 61.7, 57.2, 47.6, 14.9; FT-IR (ATR) ν 3405, 3028, 2932, 2914, 1799, 1428, 1350, 1242, 1177, 1102, 1075, 1048 cm^–1^; HPLC analysis ee (minor diastereoisomer) 99% (Daicel Chiralpak IC column, 70:30 heptane/*iso*-propanol, 1.0 mL/min, λ = 243 nm; retention times, *t*_minor_ = 21.4 min, *t*_major_ = 24.8 min) at 25 °C; [α]_D_^rt^ = −55.4° (*c* = 0.33, CHCl_3_); HRMS (ESI) *m*/*z* calcd for C_21_H_18_NO_3_ [M + H]^+^ 332.1281, found 332.1282.

#### (5*S*/5*R*,6*R*)-6-(4-Bromophenyl)-8-methyl-1-oxo-4-phenyl-2-oxa-3-azaspiro[4.4]nona-3,7-diene-7-carbaldehyde (**3b**) (3:1 mixture of diastereoisomers)

Purification of crude (dr 2:1) by silica gel chromatography (2.5:1 *n*-Hex/EA): yellow semisolid (47 mg, 95% yield) as a mixture of diastereoisomers (dr 3:1); ^1^H NMR major diastereoisomer (600 MHz, CDCl_3_) δ 10.05 (s, 1H), 7.71–7.69 (m, 2H), 7.59–7.56 (m, 1H), 7.52–7.49 (m, 2H), 7.39 (d, *J* = 8.4 Hz, 2H), 6.87 (d, *J* = 8.4 Hz, 2H), 4.92 (s, 1H), 3.29 (m, 2H), 2.42 (s, 3H); ^13^C{^1^H} NMR major diastereoisomer (151 MHz, CDCl_3_) δ 186.4, 178.3, 166.9, 160.1, 136.0, 134.2, 132.4, 131.8 (2C), 129.9 (2C), 129.8 (2C), 126.7, 126.6 (2C), 122.6, 59.3, 56.5, 47.6, 14.8; HPLC analysis ee (major diastereoisomer) 88% (Daicel Chiralpak IC column, 70:30 heptane/*iso*-propanol, 1.0 mL/min, λ = 190 nm; retention times, *t*_major_ = 13.9 min, *t*_minor_ = 16.0 min) at 25 °C; ^1^H NMR minor diastereoisomer (600 MHz, CDCl_3_) δ 10.04 (s, 1H), 7.40–7.37 (m, 1H), 7.24–7.22 (m, 2H), 7.14–7.13 (m, 2H), 7.04 (d, *J* = 8.1 Hz, 2H), 6.53 (d, *J* = 8.1 Hz, 2H), 4.90 (s, 1H), 3.45 (d, *J* = 19.4 Hz, 1H), 3.28 (d, *J* = 19.4 Hz, 1H), 2.44 (s, 3H); ^13^C{^1^H} NMR minor diastereoisomer (151 MHz, CDCl_3_) δ 186.4, 182.1, 167.2, 159.2, 136.3, 133.9, 131.5, 131.2 (2C), 129.6 (2C), 128.6 (2C), 128.1, 126.8 (2C), 121.9, 60.9, 56.9, 47.6, 14.9; HPLC analysis ee (minor diastereoisomer) 99% (Daicel Chiralpak IC column, 70:30 heptane/*iso*-propanol, 1.0 mL/min, λ = 190 nm; retention times, *t*_minor_ = 20.1 min, *t*_major_ = 25.0 min) at 25 °C; FT-IR (ATR) ν 2911, 2875, 2845, 1670, 1649, 1449, 1404, 1362, 1240, 1201, 1078 cm^–1^; [α]_D_^rt^ = −101.5° (*c* = 1.36, CHCl_3_); HRMS (ESI) *m*/*z* calcd for C_21_H_17_BrNO_3_ [M + H]^+^ 410.0386, found 410.0385.

#### 4-((5*S*/5*R*,6*R*)-7-Formyl-8-methyl-1-oxo-4-phenyl-2-oxa-3-azaspiro[4.4]nona-3,7-dien-6-yl)benzonitrile (**3c**) (3.8:1 mixture of diastereoisomers)

Purification of crude (dr 4.5:1) by silica gel chromatography (1.5:1 *n*-Hex/EA): pale yellow semisolid (31 mg, 72% yield) as a mixture of diastereoisomers (dr 3.8:1); ^1^H NMR major diastereoisomer (600 MHz, CDCl_3_) δ 10.10 (s, 1H), 7.71–7.69 (m, 2H), 7.61–7.58 (m, 1H), 7.56 (d, *J* = 8.4 Hz, 2H), 7.54–7.51 (m, 2H), 7.10 (d, *J* = 8.4 Hz, 2H), 4.98–4.96 (m, 1H), 3.34–3.30 (m, 2H), 2.46 (s, 3H); ^13^C{^1^H} NMR major diastereoisomer (151 MHz, CDCl_3_) δ 186.2, 178.1, 166.7, 160.9, 140.6, 135.7, 132.6, 132.4 (2C), 129.9 (2C), 129.1 (2C), 126.6 (2C), 126.4, 118.6, 112.5, 59.4, 56.3, 47.9, 14.8; HPLC analysis ee (major diastereoisomer) 87% (Daicel Chiralpak IA column, 80:20 heptane/*iso*-propanol, 1.0 mL/min, λ = 197 nm; retention times, *t*_major_ = 11.8 min, *t*_minor_ = 23.7 min) at 25 °C; ^1^H NMR minor diastereoisomer (600 MHz, CDCl_3_) δ 10.11 (s, 1H), 7.40–7.38 (m, 1H), 7.24 (d, *J* = 8.0 Hz, 2H), 7.22–7.20 (m, 2H), 7.13–7.10 (m, 2H), 6.76 (d, *J* = 8.0 Hz, 2H), 4.98–4.96 (m, 1H), 3.49 (d, *J* = 19.5 Hz, 1H), 3.34 (d, *J* = 19.5 Hz, 1H), 2.48 (s, 3H); ^13^C{^1^H} NMR minor diastereoisomer (151 MHz, CDCl_3_) δ 186.0, 181.8, 166.7, 160.1, 140.2, 136.0, 131.9, 131.7 (2C), 128.8 (2C), 128.8 (2C), 127.9, 126.7 (2C), 118.4, 111.7, 61.1, 56.6, 47.9, 14.9; HPLC analysis ee (minor diastereoisomer) 99% (Daicel Chiralpak IA column, 80:20 heptane/*iso*-propanol, 1.0 mL/min, λ = 197 nm; retention times, *t*_major_ = 15.2 min, *t*_minor_ = 17.0 min) at 25 °C; FT-IR (ATR) ν 3061, 2962, 2911, 2226, 1793, 1667, 1503, 1422, 1365, 1290, 1213, 1174, 1105 cm^–1^; [α]_D_^rt^ = −144.1° (*c* = 0.47, CHCl_3_); HRMS (ESI) *m*/*z* calcd for C_22_H_16_N_2_NaO_3_ [M + Na]^+^ 379.1053, found 379.1054.

#### (5*S*/5*R*,6*R*)-6-(4-Methoxyphenyl)-8-methyl-1-oxo-4-phenyl-2-oxa-3-azaspiro[4.4]nona-3,7-diene-7-carbaldehyde (**3d**) (2.4:1 mixture of diastereoisomers)

Purification of crude (dr 2.3:1) by silica gel chromatography (2:1 *n*-Hex/EA): pale yellow semisolid (34 mg, 79% yield) as a mixture of diastereoisomers (dr 2.4:1); ^1^H NMR major diastereoisomer (600 MHz, CDCl_3_) δ 10.04 (s, 1H), 7.73–7.71 (m, 2H), 7.58–7.55 (m, 1H), 7.52–7.49 (m, 2H), 6.93 (d, *J* = 8.7 Hz, 2H), 6.79 (d, *J* = 8.7 Hz, 2H), 4.96–4.94 (m, 1H), 3.76 (s, 3H), 3.27–3.26 (m, 2H), 2.41 (s, 3H); ^13^C{^1^H} NMR major diastereoisomer (151 MHz, CDCl_3_) δ 186.9, 178.6, 167.2, 159.6, 159.1, 136.3, 132.2, 129.7 (2C), 129.4 (2C), 127.0, 126.9, 126.6 (2C), 114.1 (2C), 59.6, 56.9, 55.3, 47.4, 14.8; HPLC analysis ee (major diastereoisomer) 87% (Lux-amylosa column, 80:20 heptane/*iso*-propanol, 1.0 mL/min, λ = 254 nm; retention times, *t*_major_ = 14.6 min, *t*_minor_ = 26.4 min) at 25 °C; ^1^H NMR minor diastereoisomer (600 MHz, CDCl_3_) δ 10.02 (s, 1H), 7.36–7.33 (m, 1H), 7.22–7.19 (m, 2H), 7.17–7.15 (m, 2H), 6.59 (d, *J* = 8.8 Hz, 2H), 6.45 (d, *J* = 8.8 Hz, 2H), 4.96–4.94 (m, 1H), 3.64 (s, 3H), 3.42 (d, *J* = 19.7 Hz, 1H), 3.29 (d, *J* = 19.7 Hz, 1H), 2.43 (s, 3H); ^13^C{^1^H} NMR minor diastereoisomer (151 MHz, CDCl_3_) δ 186.9, 182.4, 167.5, 159.1, 158.1, 136.7, 131.2, 129.1 (2C), 128.5 (2C), 127.2, 126.9 (2C), 126.9, 113.6 (2C), 61.2, 57.4, 55.3, 47.3, 14.9; HPLC analysis ee (minor diastereoisomer) 96% (Lux-amylosa column, 80:20 heptane/*iso*-propanol, 1.0 mL/min, λ = 254 nm; retention times, *t*_minor_ = 18.8 min, *t*_major_ = 20.9 min) at 25 °C; FT-IR (ATR) ν 3007, 2956, 2935, 2839, 1667, 1607, 1467, 1305, 1293, 1207, 1177 cm^–1^; [α]_D_^rt^ = −143.5° (*c* = 0.58, CHCl_3_); HRMS (ESI) *m*/*z* calcd for C_22_H_20_NO_4_ [M + H]^+^ 362.1387, found 362.1388.

#### (5*S*/5*R*,6*R*)-8-Methyl-1-oxo-4-phenyl-6-(*p*-tolyl)-2-oxa-3-azaspiro[4.4]nona-3,7-diene-7-carbaldehyde (**3e**) (major diastereoisomer)

Purification of crude (dr 2.2:1) by silica gel chromatography (2:1 *n*-Hex/EA): pale yellow semisolid (19 mg, 48% yield); ^1^H NMR (600 MHz, CDCl_3_) δ 10.05 (s, 1H), 7.72 (dd, *J* = 7.2 Hz, *J′* = 1.8 Hz, 2H), 7.61–7.54 (m, 1H), 7.50 (t, *J* = 7.7 Hz, 2H), 7.07 (d, *J* = 7.8 Hz, 2H), 6.89 (d, *J* = 7.8 Hz, 2H), 4.96 (q, *J* = 2.2 Hz, 1H), 3.27 (dt, *J* = 3.4 Hz, *J′* = 1.7 Hz, 2H), 2.41 (s, 3H), 2.29 (s, 3H); ^13^C{^1^H} NMR (151 MHz, CDCl_3_) δ 186.9, 178.5, 167.2, 159.2, 138.2, 136.3, 132.2 (2C), 132.0, 129.7 (2C), 129.4 (2C), 128.1 (2C), 126.7 (2C), 59.8, 56.8, 47.5, 21.3, 14.8; FT-IR (ATR) ν 3316, 3018, 2949,2923, 2901, 1790, 1668, 1444, 1236, 1174, 885 cm^–1^; HPLC analysis ee (major diastereoisomer) 85% (Daicel Chiralpak IB column, 80:20 heptane/*iso*-propanol, 1.0 mL/min, λ = 254 nm; retention times, *t*_major_ = 14.0 min, *t*_minor_ = 16.6 min) at 25 °C; [α]_D_^rt^ = −95.2° (*c* = 0.53, CHCl_3_); HRMS (ESI) *m*/*z* calcd for C_22_H_19_NNaO_3_ [M + Na]^+^ 368.1257, found 368.1255.

#### (5*S*/5*R*,6*R*)-8-Methyl-1-oxo-4-phenyl-6-(*p*-tolyl)-2-oxa-3-azaspiro[4.4]nona-3,7-diene-7-carbaldehyde (**3e**) (minor diastereoisomer)

Purification of crude (dr. 2.2:1) by silica gel chromatography (2:1 *n*-Hex/EA): pale yellow semisolid (10 mg, 25% yield); ^1^H NMR (600 MHz, CDCl_3_) δ 10.02 (s, 1H), 7.34 (t, *J* = 7.3 Hz, 1H), 7.19 (t, *J* = 7.8 Hz, 2H), 7.14 (dd, *J* = 8.0 Hz, *J′* = 1.6 Hz, 2H), 6.73 (d, *J* = 7.7 Hz, 2H), 6.56 (d, *J* = 7.7 Hz, 2H), 4.96 (d, *J* = 2.2 Hz, 1H), 3.41 (d, *J* = 19.3 Hz, 1H), 3.26 (d, *J* = 19.3 Hz, 1H), 2.43 (s, 3H), 2.13 (s, 3H); ^13^C{^1^H} NMR (151 MHz, CDCl_3_) δ 186.9, 182.4, 167.5, 158.1, 137.6, 136.6, 131.7, 131.2, 128.8 (2C), 128.4 (2C), 128.3, 127.9 (2C), 127.0 (2C), 61.5, 57.4, 47.4, 21.1, 14.9; FT-IR (ATR) ν 3309, 3053, 2920, 2854, 1786, 1662, 1637, 1446, 1174, 773 cm^–1^; HPLC analysis ee 96% (Daicel Chiralpak IB column, 80:20 heptane/*iso*-propanol, 1.0 mL/min, λ = 254 nm; retention times, *t*_minor_ = 14.0 min, *t*_major_ = 25.6 min) at 25 °C; [α]_D_^rt^ = −88.7° (*c* = 0.31, CHCl_3_); HRMS (ESI) *m*/*z* calcd for C_22_H_19_NNaO_3_ [M + Na]^+^ 368.1257, found 368.1255.

#### (5*S*,6*R*)-8-Methyl-1-oxo-4-phenyl-6-(*m*-tolyl)-2-oxa-3-azaspiro[4.4]nona-3,7-diene-7-carbaldehyde (**3f**) (major diastereoisomer)

The crude product (reaction time of 3 days, dr 1.8:1) was purified by silica gel flash chromatography with a 3:1 *n*-hexane/ethyl acetate eluent to give **3f** as a pale yellow oil in 36% yield (16 mg): ^1^H NMR (600 MHz, CDCl_3_) δ 10.10 (s, 1H), 7.71–7.69 (m, 2H), 7.61–7.58 (m, 1H), 7.56 (d, *J* = 8.4 Hz, 2H), 7.54–7.51 (m, 2H), 7.10 (d, *J* = 8.4 Hz, 2H), 4.98–4.96 (m, 1H), 3.34–3.30 (m, 2H), 2.46 (s, 3H); ^13^C{^1^H} NMR (151 MHz, CDCl_3_) δ 186.2, 178.1, 166.7, 160.9, 140.6, 135.7, 132.6, 132.4 (2C), 129.9 (2C), 129.1 (2C), 126.6 (2C), 126.4, 118.6, 112.5, 59.4, 56.3, 47.9, 14.8; FT-IR (ATR) ν 3012, 2922, 2868, 1797, 1662, 1442, 1365, 1264, 1078 cm^–1^; [α]_D_^rt^ = −61.9° (*c* = 0.42, CHCl_3_); HPLC analysis ee 85% (Daicel Chiralpak IA column, 80:20 heptane/*iso*-propanol, 0.5 mL/min, λ = 254 nm; retention times, *t*_major_ = 18.2 min, *t*_minor_ = 20.9 min) at 25 °C; HRMS (ESI) *m*/*z* calcd for C_22_H_19_NO_3_ [M + H]^+^ 346.1438, found 346.1437.

#### (5*R*,6*R*)-8-Methyl-1-oxo-4-phenyl-6-(*m*-tolyl)-2-oxa-3-azaspiro[4.4]nona-3,7-diene-7-carbaldehyde (**3f**) (minor diastereoisomer)

Purification of crude (dr 1.8:1) by silica gel chromatography (3:1 *n*-Hex/EA): pale yellow semisolid (10 mg, 24% yield); ^1^H NMR (600 MHz, CDCl_3_) δ 10.11 (s, 1H), 7.40–7.38 (m, 1H), 7.24 (d, *J* = 8.0 Hz, 2H), 7.22–7.20 (m, 2H), 7.13–7.10 (m, 2H), 6.76 (d, *J* = 8.0 Hz, 2H), 4.98–4.96 (m, 1H), 3.49 (d, *J* = 19.5 Hz, 1H), 3.34 (d, *J* = 19.5 Hz, 1H), 2.48 (s, 3H); ^13^C{^1^H} NMR (151 MHz, CDCl_3_) δ 186.0, 181.8, 166.7, 160.1, 140.2, 136.0, 131.9, 131.7 (2C), 128.8 (2C), 128.8 (2C), 127.9, 126.7 (2C), 118.4, 111.7, 61.1, 56.6, 47.9, 14.9; FT-IR (ATR) ν 3288, 3024, 2918, 2850, 1784, 1672, 1433, 1182, 1099 cm^–1^; HPLC analysis ee 98% (Daicel Chiralpak IC column, 80:20 heptane/*iso*-propanol, 1.0 mL/min, λ = 197 nm; retention times, *t*_major_ = 34.9 min, *t*_minor_ = 31.0 min) at 25 °C; [α]_D_^rt^ = −94.5° (*c* = 0.28, CHCl_3_); HRMS (ESI) *m*/*z* calcd for C_22_H_19_NO_3_ [M + H]^+^ 346.1438, found 346.1436.

#### (5*S*,6*R*)-6-(Furan-2-yl)-8-methyl-1-oxo-4-phenyl-2-oxa-3-azaspiro[4.4]nona-3,7-diene-7-carbaldehyde (**3h**) (major diastereoisomer)

Purification of crude (dr 1.2:1) by silica gel chromatography (2:1 *n*-Hex/EA): pale yellow semisolid (16 mg, 42% yield); mp 155–157 °C (*iso*-propanol); ^1^H NMR (600 MHz, CDCl_3_) δ 10.05 (s, 1H), 7.69–7.59 (m, 2H), 7.59–7.51 (m, 1H), 7.48 (t, *J* = 7.7 Hz, 2H), 7.32 (d, *J* = 1.8 Hz, 1H), 6.31 (dd, *J* = 3.3 Hz, *J′* = 1.9 Hz, 1H), 6.11 (d, *J* = 3.2 Hz, 1H), 5.13–4.95 (m, 1H), 3.47–3.29 (m, 1H), 3.19 (dt, *J* = 19.5 Hz, *J′* = 1.3 Hz, 1H), 2.38 (s, 3H); ^13^C{^1^H} NMR (151 MHz, CDCl_3_) δ 186.3, 178.2, 167.2, 159.2, 148.7, 142.8, 134.6, 132.2, 129.7 (2C), 126.8, 126.6 (2C), 110.9, 109.1, 55.2, 53.0, 47.7, 14.8; FT-IR (ATR) ν 3159, 2925, 2854, 1797, 1664, 1552, 1498, 1234, 1014, 891 cm^–1^; HPLC analysis ee 78% (Daicel Chiralpak IB column, 80:20 heptane/*iso*-propanol, 1.0 mL/min, λ = 200 nm; retention times, *t*_major_ = 17.5 min, *t*_minor_ = 21.5 min) at 25 °C; [α]_D_^rt^ = −69.4° (*c* = 0.56, CHCl_3_); HRMS (ESI) *m*/*z* calcd for C_19_H_15_NNaO_4_ [M + Na]^+^ 344.0893, found 344.0891.

#### (5*R*,6*R*)-6-(furan-2-yl)-8-methyl-1-oxo-4-phenyl-2-oxa-3-azaspiro[4.4]nona-3,7-diene-7-carbaldehyde (**3h**) (minor diastereoisomer)

Purification of crude (dr 1.2:1) by silica gel chromatography (2:1 *n*-Hex/EA): pale yellow semisolid (12 mg, 32% yield); ^1^H NMR (600 MHz, CDCl_3_) δ 10.04 (s, 1H), 7.44–7.34 (m, 3H), 7.28 (m, 2H), 6.85 (d, *J* = 1.9 Hz, 1H), 5.96 (dd, *J* = 3.4 Hz, *J′* = 1.9 Hz, 1H), 5.86 (d, *J* = 3.3 Hz, 1H), 4.98 (d, *J* = 2.0 Hz, 1H), 3.40 (d, *J* = 19.3 Hz, 1H), 3.22 (d, *J* = 19.4 Hz, 1H), 2.39 (s, 3H); ^13^C{^1^H} NMR (151 MHz, CDCl_3_) δ 186.2, 181.7, 166.9, 158.1, 148.5, 141.8, 135.0, 131.4, 128.5 (2C), 127.3, 127.1 (2C), 110.6, 109.2, 56.0, 54.6, 47.6, 14.8; FT-IR (ATR) ν 3294, 3120, 2924, 2852, 1784, 1668, 1442, 1176, 883 cm^–1^; HPLC analysis ee 87% (Daicel Chiralpak IB column, 80:20 heptane/*iso*-propanol, 1.0 mL/min, λ = 200 nm; retention times, *t*_minor_ = 16.7 min, *t*_major_ = 31.0 min) at 25 °C; [α]_D_^rt^ = −76.1° (*c* = 0.36, CHCl_3_); HRMS (ESI) *m*/*z* calcd for C_19_H_15_NNaO_4_ [M + Na]^+^ 344.0893, found 344.0891.

#### (5*S*/5*R*,6*R*)-6,8-Dimethyl-1-oxo-4-phenyl-2-oxa-3-azaspiro[4.4]nona-3,7-diene-7-carbaldehyde (**3i**) (2.1:1 mixture of diastereoisomers)

Purification of crude (dr 2.2:1) by silica gel chromatography (3:1 *n*-Hex/EA): yellow semisolid (31 mg, 96% yield) as a mixture of diastereoisomers (dr 2.1:1); ^1^H NMR major diastereoisomer (600 MHz, CDCl_3_) δ 10.05 (s, 1H), 7.61–7.60 (m, 2H), 7.53–7.49 (m, 1H), 7.45–7.42 (m, 2H), 3.80–3.76 (m, 1H), 3.17–3.16 (m, 2H), 2.26 (s, 3H), 1.34 (d, *J* = 7.1 Hz, 3H); ^13^C{^1^H} NMR major diastereoisomer (151 MHz, CDCl_3_) δ 187.2, 179.5, 167.6, 157.5, 138.4, 132.1, 129.5 (2C), 126.9, 126.6 (2C), 55.1, 48.4, 47.4, 14.7, 14.3; HPLC analysis ee (major diastereoisomer) 70% (Daicel Chiralpak IC column, 90:10 heptane/*iso*-propanol, 1.0 mL/min, λ = 190 nm; retention times, *t*_minor_ = 26.2 min, *t*_major_ = 29.6 min) at 25 °C; ^1^H NMR minor diastereoisomer (600 MHz, CDCl_3_) δ 10.09 (s, 1H), 7.67–7.65 (m, 2H), 7.53–7.49 (m, 1H), 7.45–7.42 (m, 2H), 3.84–3.81 (m, 1H), 3.18 (d, *J* = 19.4 Hz, 1H), 3.05 (d, *J* = 19.4 Hz, 1H), 2.27 (s, 3H) 1.21 (d, *J* = 7.5 Hz, 3H); ^13^C{^1^H} NMR minor diastereoisomer (151 MHz, CDCl_3_) δ 187.1, 182.6, 167.7, 157.6, 138.6, 132.0, 129.3 (2C), 128.4, 127.2 (2C), 55.8, 52.1, 47.3, 14.5, 14.3; HPLC analysis ee (minor diastereoisomer) 90% (Daicel Chiralpak IC column, 90:10 heptane/*iso*-propanol, 1.0 mL/min, λ = 190 nm; retention times, *t*_major_ = 34.3 min, *t*_minor_ = 52.5 min) at 25 °C; FT-IR (KBr) ν 3064, 2917, 2765, 1784, 1643, 1419, 1350, 1242, 1183 cm^–1^; [α]_D_^rt^ = −13.8° (*c* = 1.30 in CHCl_3_); HRMS (ESI) *m*/*z* calcd for C_16_H_15_O_3_NNa [M + Na]^+^ 292.0944, found 292.0944.

#### (5*S*/5*R*,6*R*)-6-Ethyl-8-methyl-1-oxo-4-phenyl-2-oxa-3-azaspiro[4.4]nona-3,7-diene-7-carbaldehyde (**3j**) (2.1:1 mixture of diastereoisomers)

Purification of crude (dr 1.9:1) by silica gel chromatography (3:1 *n*-Hex/EA): yellow semisolid (32 mg, 94% yield) as a mixture of diastereoisomers (dr 2.1:1); ^1^H NMR major diastereoisomer (600 MHz, CDCl_3_) δ 10.04 (s, 1H), 7.60–7.58 (m, 2H), 7.53–7.50 (m, 1H), 7.45–7.42 (m, 2H), 3.62 (d, *J* = 11.1 Hz, 1H), 3.22–3.13 (m, 2H), 2.25 (s, 3H), 2.19–2.12 (m, 1H), 1.81–1.73 (m, 1H), 0.81 (t, *J* = 7.6 Hz, 3H); ^13^C{^1^H} NMR major diastereoisomer (151 MHz, CDCl_3_) δ 187.5, 180.1, 168.8, 157.6, 138.3, 132.1, 129.5 (2C), 126.7, 126.6 (2C), 55.8, 52.9, 49.1, 21.9, 14.3, 12.2; HPLC analysis ee (major diastereoisomer) 76% (Daicel Chiralpak IB column, 80:20 heptane/*iso*-propanol, 0.5 mL/min, λ = 200 nm; retention times, *t*_major_ = 19.1 min, *t*_minor_ = 22.1 min) at 25 °C; ^1^H NMR minor diastereoisomer (600 MHz, CDCl_3_) δ 10.07 (s, 1H), 7.69–7.68 (m, 2H), 7.53–7.50 (m, 1H), 7.45–7.42 (m, 2H), 3.67 (d, *J* = 8.9 Hz, 1H), 3.13 (d, *J* = 19.5 Hz, 1H), 3.04 (d, *J* = 19.5 Hz, 1H), 2.34–2.27 (m, 1H), 2.25 (s, 3H), 1.28–1.20 (m, 1H), 0.78 (t, *J* = 8.0 Hz, 3H); ^13^C{^1^H} NMR minor diastereoisomer (151 MHz, CDCl_3_) δ 187.5, 183.1, 168.0, 157.8, 138.3, 132.0, 129.3 (2C), 128.2, 127.1 (2C), 59.5, 54.6, 48.8, 21.6, 14.3, 12.4; HPLC analysis ee (minor diastereoisomer) 90% (Daicel Chiralpak IB column, 80:20 heptane/*iso*-propanol, 0.5 mL/min, λ = 200 nm; retention times, *t*_minor_ = 20.3 min, *t*_major_ = 28.0 min) at 25 °C; FT-IR (ATR) ν 3055, 2996, 2760, 1780, 1646, 1391, 1246, 1218, 1180, 1115 cm^–1^; [α]_D_^rt^ = −20.2° (*c* = 1.61 in CHCl_3_); HRMS (ESI) *m*/*z* calcd for C_17_H_17_O_3_NNa [M + Na]^+^ 306.1101, found 306.1100.

#### (5*S*/5*R*,6*R*)-8-Methyl-1-oxo-4-phenyl-6-propyl-2-oxa-3-azaspiro[4.4]nona-3,7-diene-7-carbaldehyde (**3k**) (2.1:1 mixture of diastereoisomers)

Purification of crude (dr 1.7:1) by silica gel chromatography (4:1 *n*-Hex/EA): yellow oil (35 mg, 97% yield) as a mixture of diastereoisomers (dr 2.1:1); ^1^H NMR major diastereoisomer (600 MHz, CDCl_3_) δ 10.04 (s, 1H), 7.59–7.58 (m, 2H), 7.52–7.49 (m, 1H), 7.44–7.42 (m, 2H), 3.62 (d, *J* = 11.1 Hz, 1H), 3.22–3.13 (m, 2H), 2.24 (s, 3H), 2.19–2.12 (m, 1H), 1.81–1.73 (m, 1H), 1.22–1.11 (m, 2H), 0.81 (t, *J* = 7.6 Hz, 3H); ^13^C{^1^H} NMR major diastereoisomer (151 MHz, CDCl_3_) δ 187.3, 180.0, 168.8, 157.2, 138.5, 132.1, 129.5 (2C), 126.9, 126.6 (2C), 53.8, 53.2, 49.2, 30.8, 21.0, 14.2, 14.0; HPLC analysis ee (major diastereoisomer) 74% (Daicel Chiralpak IC column, 90:10 heptane/*iso*-propanol, 1 mL/min, λ = 190 nm; retention times, *t*_major_ = 13.1 min, *t*_minor_ = 16.1 min) at 25 °C; ^1^H NMR minor diastereoisomer (600 MHz, CDCl_3_) δ 10.06 (s, 1H), 7.68–7.66 (m, 2H), 7.52–7.49 (m, 1H), 7.44–7.42 (m, 2H), 3.74 (d, *J* = 7.8 Hz, 1H), 3.15 (d, *J* = 19.3 Hz, 1H), 3.04 (d, *J* = 19.3 Hz, 1H), 2.24 (s, 3H), 2.19–2.13 (m, 2H), 1.22–1.11 (m, 2H), 0.72 (t, *J* = 7.1 Hz, 3H); ^13^C{^1^H} NMR minor diastereoisomer (151 MHz, CDCl_3_) δ 187.3, 182.9, 168.2, 157.3, 138.4, 132.0, 129.3 (2C), 128.4, 127.2 (2C), 57.2, 54.9, 48.8, 30.6, 21.1, 14.0, 13.9; HPLC analysis ee (minor diastereoisomer) 94% (Daicel Chiralpak IC column, 90:10 heptane/*iso*-propanol, 1 mL/min, λ = 190 nm; retention times, *t*_major_ = 18.7 min, *t*_minor_ = 23.1 min) at 25 °C; FT-IR (ATR) ν 3064, 2959, 2872, 1799, 1673, 1446, 1344, 1296, 1210, 1108 cm^–1^; [α]_D_^rt^ = −23.4° (*c* = 1.56 in CHCl_3_); HRMS (ESI) *m*/*z* calcd for C_18_H_19_O_3_NNa [M + Na]^+^ 320.1257, found 320.1256.

#### (5*S*/5*R*,6*R*)-6-Butyl-8-methyl-1-oxo-4-phenyl-2-oxa-3-azaspiro[4.4]nona-3,7-diene-7-carbaldehyde (**3l**) (2.2:1 mixture of diastereoisomers)

Purification of crude (dr 1.7:1) by silica gel chromatography (3:1 *n*-Hex/EA): yellow semisolid (34 mg, 92% yield) as a mixture of diastereoisomers (dr 2.2:1); ^1^H NMR major diastereoisomer (600 MHz, CDCl_3_) δ 10.03 (s, 1H), 7.60–7.58 (m, 2H), 7.52–7.49 (m, 1H), 7.45–7.41 (m, 2H), 3.67 (d, *J* = 11.0 Hz, 1H), 3.21–3.12 (m, 2H), 2.24 (s, 3H), 2.08–2.02 (m, 1H), 1.80–1.73 (m, 1H), 1.26–1.15 (m, 3H), 1.14–1.08 (m, 1H), 0.82 (t, *J* = 7.3 Hz, 3H); ^13^C{^1^H} NMR major diastereoisomer (151 MHz, CDCl_3_) δ 187.3, 180.1, 168.8, 157.1, 138.5, 132.1, 129.5 (2C), 126.9, 126.6 (2C), 54.0, 53.2, 49.2, 29.7, 28.4, 22.6, 14.2, 13.8; HPLC analysis ee (major diastereoisomer) 80% (Daicel Chiralpak IC column, 90:10 heptane/*iso*-propanol, 1.0 mL/min, λ = 200 nm; retention times, *t*_minor_ = 11.7 min, *t*_major_ = 13.9 min) at 25 °C; ^1^H NMR minor diastereoisomer (600 MHz, CDCl_3_) δ 10.06 (s, 1H), 7.69–7.66 (m, 2H), 7.52–7.49 (m, 1H), 7.45–7.41 (m, 2H), 3.72 (d, *J* = 11.1 Hz, 1H), 3.16 (d, *J* = 19.3 Hz, 1H), 3.05 (d, *J* = 19.3 Hz, 1H), 2.24 (s, 3H), 2.21–2.15 (m, 1H), 1.14–1.08 (m, 3H), 1.08–1.02 (m, 2H), 0.72 (t, *J* = 7.0 Hz, 3H); ^13^C{^1^H} NMR minor diastereoisomer (151 MHz, CDCl_3_) δ 187.3, 182.9, 168.1, 157.2, 138. 5, 132.0, 129.3 (2C), 128.4, 127.2 (2C), 57.4, 54.9, 48.9, 29.9, 28.1, 22.4, 13.8, 13.7; HPLC analysis ee (minor diastereoisomer) 95% (Daicel Chiralpak IC column, 90:10 heptane/*iso*-propanol, 1.0 mL/min, λ = 200 nm; retention times, *t*_major_ = 16.4 min, *t*_minor_ = 20.6 min) at 25 °C; FT-IR (ATR) ν 2959, 2935, 2854, 1670, 1640, 1425, 1347, 1254, 1180 cm^–1^; [α]_D_^rt^ = −30.1° (*c* = 1.15 in CHCl_3_); HRMS (ESI) *m*/*z* calcd for C_19_H_21_O_3_NNa [M + Na]^+^ 334.1414, found 334.1412.

#### (5*S*/5*R*,6*R*)-6-(But-3-en-1-yl)-8-methyl-1-oxo-4-phenyl-2-oxa-3-azaspiro[4.4]nona-3,7-diene-7-carbaldehyde (**3m**) (2.8:1 mixture of diastereoisomers)

Purification of crude (dr 2.7:1) by silica gel chromatography (3:1 *n*-Hex/EA): pale yellow semisolid (27 mg, 74% yield) as a mixture of diastereoisomers (dr 2.8:1); ^1^H NMR major diastereoisomer (600 MHz, CDCl_3_) δ 10.03 (s, 1H), 7.58–7.56 (m, 2H), 7.52–7.49 (m, 1H), 7.45–7.41 (m, 2H), 5.67–5.55 (m, 1H), 4.94–4.89 (m, 1H), 4.85 (d, *J* = 10.9 Hz, 1H), 3.73 (d, *J* = 11.0 Hz, 1H), 3.23–3.16 (m, 2H), 2.25 (s, 3H), 2.20–2.13 (m, 1H), 2.05–1.98 (m, 1H), 1.93–1.81 (m, 2H); ^13^C{^1^H} NMR major diastereoisomer (151 MHz, CDCl_3_) δ 187.2, 180.1, 168.6, 157.4, 138.4, 136.9, 132.1, 129.5 (2C), 126.9, 126.6 (2C), 116.3, 56.2, 52.7, 49.3, 31.7, 27.8, 14.3; HPLC analysis ee (major diastereoisomer) 74% (Daicel Chiralpak IC column, 90:10 heptane/*iso*-propanol, 1.0 mL/min, λ = 190 nm; retention times, *t*_minor_ = 13.5 min, *t*_major_ = 16.8 min) at 25 °C; ^1^H NMR minor diastereoisomer (600 MHz, CDCl_3_) δ 10.07 (s, 1H), 7.69–7.67 (m, 2H), 7.52–7.49 (m, 1H), 7.45–7.41 (m, 2H), 5.67–5.55 (m, 1H), 4.94–4.89 (m, 2H), 3.77 (d, *J* = 11.3 Hz, 1H), 3.14 (d, *J* = 19.3 Hz, 1H), 3.06 (d, *J* = 19.3 Hz, 1H), 2.36–2.28 (m, 1H), 2.25 (s, 3H), 1.93–1.81 (m, 2H), 1.37–1.30 (m, 1H); ^13^C{^1^H} NMR minor diastereoisomer (151 MHz, CDCl_3_) δ 187.2, 182.7, 168.0, 157.5, 138.4, 136.7, 132.1, 129.4 (2C), 128.3, 127.2 (2C), 116.3, 54.7, 53.0, 48.9, 31.8, 27.8, 14.3; HPLC analysis ee (minor diastereoisomer) 91% (Daicel Chiralpak IC column, 90:10 heptane/*iso*-propanol, 1.0 mL/min, λ = 190 nm; retention times, *t*_major_ = 20.4 min, *t*_minor_ = 23.9 min) at 25 °C; FT-IR (KBr) ν 3076, 2947, 2845, 2762, 1673, 1443, 1377, 1341, 1287, 1198, 1180 cm^–1^; [α]_D_^rt^ = −20.7° (*c* = 0.92 in CHCl_3_); HRMS (ESI) *m*/*z* calcd for C_19_H_19_NO_3_ [M + H]^+^ 309.1365, found 309.1364.

#### Ethyl (5*S*,6*R*)-7-Formyl-8-methyl-1-oxo-4-phenyl-2-oxa-3-azaspiro[4.4]nona-3,7-diene-6-carboxylate (**3n**) (major diastereoisomer)

Purification of crude (dr 2.5:1) by silica gel chromatography (4:1 *n*-Hex/EA): pale yellow semisolid (15 mg, 38% yield); ^1^H NMR (600 MHz, CDCl_3_) δ 10.07 (s, 1H), 7.61–7.50 (m, 2H), 7.48–7.42 (m, 1H), 4.43 (s, 1H), 4.25–4.06 (m, 2H), 3.33 (d, *J* = 19.5 Hz, 1H), 3.18 (d, *J* = 19.5 Hz, 1H), 2.32 (s, 3H), 1.34–1.13 (m, 3H); ^13^C{^1^H} NMR (151 MHz, CDCl_3_) δ 186.4, 179.3, 167.7, 167.1, 158.1, 134.2, 132.4, 129.7 (2C), 126.4 (2C), 62.3, 57.7, 52.5, 49.3, 14.5, 13.8; FT-IR (KBr) ν 3066, 2960, 2850, 1790, 1743, 1668, 1444, 1180 cm^–1^; HPLC analysis ee 80% (Daicel Chiralpak IB column, 70:30 heptane/*iso*-propanol, 1.0 mL/min, λ = 254 nm; retention times, *t*_major_ = 9.6 min, *t*_minor_ = 19.7 min) at 25 °C; [α]_D_^rt^ = −45.5° (*c* = 0.55 in CHCl_3_); HRMS (ESI) *m*/*z* calcd for C_18_H_17_NO_5_ [M + H]^+^ 327.1107, found 327.1108.

#### Ethyl (5*R*,6*R*)-7-Formyl-8-methyl-1-oxo-4-phenyl-2-oxa-3-azaspiro[4.4]nona-3,7-diene-6-carboxylate (**3n**) (minor diastereoisomer)

Purification of crude (dr 2.5:1) by silica gel chromatography (4:1 *n*-Hex/EA): pale yellow semisolid (9 mg, 23% yield); ^1^H NMR (600 MHz, CDCl_3_) δ 10.05 (s, 1H), 7.62 (d, *J* = 7.7 Hz, 2H), 7.49 (t, *J* = 7.6 Hz, 1H), 7.41 (t, *J* = 7.9 Hz, 2H), 4.49 (s, 1H), 3.84–3.67 (m, 1H), 3.64–3.52 (m, 1H), 3.25 (d, *J* = 19.5 Hz, 1H), 3.20 (d, *J* = 19.4 Hz, 1H), 2.32 (s, 3H), 0.95 (td, *J* = 7.2, 2.1 Hz, 3H); ^13^C{^1^H} NMR (151 MHz, CDCl_3_) δ 186.1, 181.5, 167.6, 166.3, 157.1, 134.5, 132.3, 129.1 (2C), 127.0 (2C), 126.5, 62.0, 59.1, 52.4, 48.8, 14.5, 13.6; FT-IR (KBr) ν 3060, 2985, 2854, 1792, 1174, 1670, 1188 cm^–1^; HPLC analysis ee 76% (Daicel Chiralpak IB column, 70:30 heptane/*iso*-propanol, 1.0 mL/min, λ = 254 nm; retention times, *t*_minor_ = 12.9 min, *t*_major_ = 15.4 min) at 25 °C; [α]_D_^rt^ = −36.4° (*c* = 0.22 in CHCl_3_); HRMS (ESI) *m*/*z* calcd for C_18_H_17_NO_5_ [M + H]^+^ 327.1107, found 327.1106.

#### (5*S*,6*R*)-4,8-Dimethyl-1-oxo-6-phenyl-2-oxa-3-azaspiro[4.4]nona-3,7-diene-7-carbaldehyde (**3o**) (major diastereoisomer)

Purification of crude (dr 1.1:1) by silica gel chromatography (2:1 *n*-Hex/EA): pale yellow solid (15 mg, 45% yield); mp 157.8 °C (*iso*-propanol); ^1^H NMR (600 MHz, CDCl_3_) δ 10.03 (s, 1H), 7.33–7.28 (m, 3H), 7.03–7.00 (m, 2H), 4.73 (s, 1H), 2.98 (d, *J* = 18.9 Hz, 1H), 2.90 (d, *J* = 18.9 Hz, 1H), 2.39 (s, 3H), 1.24 (s, 3H); ^13^C{^1^H} NMR (151 MHz, CDCl_3_) δ 186.4, 181.1, 167.5, 158.2, 136.4, 135.8, 129.2 (2C), 128.6, 127.8 (2C), 59.4, 56.7, 44.9, 14.7, 13.0; FT-IR (KBr) ν 2950, 2854, 1784, 1658, 1580, 1455, 1380, 1305, 1242, 1168 cm^–1^; HPLC analysis ee 88%, (Daicel Chiralpak IA column, 90:10 heptane/*iso*-propanol, 1.0 mL/min, λ = 233 nm; retention times, *t*_major_ = 15.7 min, *t*_minor_ = 17.8 min) at 25 °C; [α]_D_^rt^ = −165.3° (*c* = 0.38 in CHCl_3_); HRMS (ESI) *m*/*z* calcd for C_16_H_16_NO_3_ [M + H]^+^ 270.1125, found 270.1129.

#### (5*R*,6*R*)-4,8-Dimethyl-1-oxo-6-phenyl-2-oxa-3-azaspiro[4.4]nona-3,7-diene-7-carbaldehyde (**3o**) (minor diastereoisomer)

Purification of crude (dr 1.1:1) by silica gel chromatography (2:1 *n*-Hex/EA): pale yellow solid (15 mg, 45% yield); ^1^H NMR (600 MHz, CDCl_3_) δ 10.00 (s, 1H), 7.31–7.25 (m, 3H), 7.01–6.99 (m, 2H), 4.48 (s, 1H), 3.18 (d, *J* = 19.0 Hz, 1H), 2.78 (d, *J* = 19.0 Hz, 1H), 2.38 (s, 3H), 2.13 (s, 3H); ^13^C{^1^H} NMR (151 MHz, CDCl_3_) δ 186.3, 177.3, 168.1, 158.6, 136.7, 134.7, 128.8 (2C), 128.6, 128.1 (2C), 57.9, 56.9, 45.4, 14.6, 12.0; FT-IR (KBr) ν 3061, 3013, 1799, 1664, 1494, 1392, 1296, 1177 cm^–1^; HPLC analysis ee (minor diastereoisomer) 80% (Daicel Chiralpak IA column, 80:20 heptane/*iso*-propanol, 1.0 mL/min, λ = 190 nm; retention times, *t*_major_ = 8.4 min, *t*_minor_ = 9.3 min) at 25 °C; [α]_D_^rt^ = −9.4° (*c* = 0.32 in CHCl_3_); HRMS (ESI) *m*/*z* calcd for C_16_H_16_NO_3_ [M + H]^+^ 270.1125, found 270.1123.

#### (5*S*,6*R*)-4,8-Dimethyl-1-oxo-6-(*o*-tolyl)-2-oxa-3-azaspiro[4.4]nona-3,7-diene-7-carbaldehyde (**3p**) (major diastereoisomer)

Purification of crude oil (dr 1.9:1) by silica gel chromatography (2:1 *n*-Hex/EA): pale yellow semisolid (13 mg, 37% yield); ^1^H NMR (600 MHz, CDCl_3_) δ 10.00 (s, 1H), 7.22–7.16 (m, 2H), 7.12–7.08 (m, 1H), 6.89–6.87 (m, 1H), 4.95 (s, 1H), 3.06 (d, *J* = 19.1 Hz, 1H), 2.83 (d, *J* = 19.1 Hz, 1H), 2.40 (s, 3H), 2.26 (s, 3H), 1.14 (s, 3H); ^13^C{^1^H} NMR (151 MHz, CDCl_3_) δ 186.1, 181.5, 167.9, 157.6, 137.5, 136.8, 134.5, 131.7, 128.4, 126.9, 126.3, 55.2, 55.0, 45.4, 19.8, 14.6, 12.9; FT-IR (KBr) ν 2950, 2854, 1784, 1604, 1580, 1455, 1380, 1305, 1242, 1168 cm^–1^; HPLC analysis ee (major diastereoisomer) 82% (Daicel Chiralpak IA column, 95:5 heptane/*iso*-propanol, 1.0 mL/min, λ = 190 nm; retention times, *t*_minor_ = 20.3 min, *t*_major_ = 22.0 min) at 25 °C; [α]_D_^rt^ = −170.1° (*c* = 0.435 in CHCl_3_); HRMS (ESI) *m*/*z* calcd for C_17_H_18_NO_3_ [M + H]^+^ 284.1281, found 284.1289.

#### (5*R*,6*R*)-4,8-Dimethyl-1-oxo-6-(*o*-tolyl)-2-oxa-3-azaspiro[4.4]nona-3,7-diene-7-carbaldehyde (**3p**) (minor diastereoisomer)

Purification of crude (dr 1.9:1) by silica gel chromatography (2:1 *n*-Hex/EA): pale yellow semisolid (8 mg, 24% yield); ^1^H NMR (600 MHz, CDCl_3_) δ 9.99 (s, 1H), 7.18–7.10 (m, 3H), 6.89–6.87 (m, 1H), 4.73 (s, 1H), 3.25 (d, *J* = 19.2 Hz, 1H), 2.71 (d, *J* = 19.2 Hz, 1H), 2.40 (s, 3H), 2.24 (s, 3H), 2.11 (s, 3H); ^13^C{^1^H} NMR (151 MHz, CDCl_3_) δ 186.1, 177.0, 168.8, 158.0, 137.8, 136.0, 132.9, 131.2, 128.2, 127.6, 126.1, 55.2, 53.1, 46.1, 19.7, 14.5, 11.9; FT-IR (KBr) ν 3061, 2926, 1799, 1640, 1434, 1341, 1296, 1216 cm^–1^; HPLC analysis ee (minor diastereoisomer) 69% (Daicel Chiralpak IA column, 97:3 heptane/*iso*-propanol, 1.0 mL/min, λ = 223 nm; retention times, *t*_major_ = 26.7 min, *t*_minor_ = 31.5 min) at 25 °C; [α]_D_^rt^ = −24.6° (*c* = 0.29 in CHCl_3_); HRMS (ESI) *m*/*z* calcd for C_17_H_18_NO_3_ [M + H]^+^ 284.1281, found 284.1285.

#### (5*S*/5*R*,6*R*)-4,8-Dimethyl-1-oxo-6-propyl-2-oxa-3-azaspiro[4.4]nona-3,7-diene-7-carbaldehyde (**3q**) (1.4:1 mixture of diastereoisomers)

Purification of crude oil (dr 1.3:1) by silica gel chromatography (2:1 *n*-Hex/EA): pale brown semisolid (27 mg, 96% yield) as a mixture of diastereoisomers (dr 1.4:1); ^1^H NMR major diastereoisomer (600 MHz, CDCl_3_) δ 10.00 (s, 1H), 3.56–3.52 (m, 1H), 2.65 (d, *J* = 19.0 Hz, 1H), 2.60 (d, *J* = 19.0 Hz, 1H), 2.30–2.24 (m, 1H), 2.20 (s, 3H), 2.05 (s, 3H), 1.27–1.11 (m, 3H), 0.88 (t, *J* = 7.0 Hz, 3H); ^13^C{^1^H} NMR major diastereoisomer (151 MHz, CDCl_3_) δ 187.3, 181.2, 168.0, 156.9, 137.5, 56.5, 54.4, 46.3, 30.7, 20.9, 14.4, 14.1, 14.0; HPLC analysis ee (major diastereoisomer) 77% (Daicel Chiralpak IB column, 95:5 heptane/*iso*-propanol, 1.0 mL/min, λ = 202 nm; retention times, *t*_major_ = 21.1 min, *t*_minor_ = 23.5 min) at 25 °C; ^1^H NMR minor diastereoisomer (600 MHz, CDCl_3_) δ 9.97 (s, 1H), 3.23–3.20 (m, 1H), 3.11 (d, *J* = 19.0 Hz, 1H), 3.00 (d, *J* = 19.0 Hz, 1H), 2.20 (s, 3H), 1.95 (s, 3H), 1.74–1.70 (m, 2H), 1.27–1.11 (m, 2H), 0.85 (t, *J* = 7.3 Hz, 3H); ^13^C{^1^H} NMR minor diastereoisomer (151 MHz, CDCl_3_) δ 186.8, 179.1, 169.2, 156.6, 138.7, 54.0, 51.8, 46.5, 30.8, 21.4, 14.1, 13.9, 11.7; HPLC analysis ee (minor diastereoisomer) 74% (Daicel Chiralpak IB column, 95:5 heptane/*iso*-propanol, 1.0 mL/min, λ = 202 nm; retention times, *t*_minor_ = 18.2 min, *t*_major_ = 19.4 min) at 25 °C; FT-IR (KBr) ν 2962, 2872, 1787, 1673, 1443, 1383, 1338, 1266, 1210, 1117 cm^–1^; [α]_D_^rt^ = −18.4° (*c* = 0.95 in CHCl_3_); HRMS (ESI) *m*/*z* calcd for C_13_H_18_NO_3_ [M + H]^+^ 236.1281, found 236.1286.

#### (5*R*,6*R*)-4-(*tert*-Butyl)-8-methyl-1-oxo-6-phenyl-2-oxa-3-azaspiro[4.4]nona-3,7-diene-7-carbaldehyde (**3r**)

Purification of crude (dr 20:1) by silica gel chromatography (2:1 *n*-Hex/EA): pale yellow solid (18 mg, 49% yield); mp 87–89 °C (*iso*-propanol); ^1^H NMR (600 MHz, CDCl_3_) δ 10.01 (s, 1H), 7.31–7.23 (m, 3H), 7.02–6.95 (m, 2H), 4.82 (d, *J* = 1.9 Hz, 1H), 3.18 (ddd, *J* = 19.3 Hz, *J′* = 2.3 Hz, *J″* = 1.2 Hz, 1H), 3.11 (dt, *J* = 19.3 Hz, *J′* = 1.3 Hz, 1H), 2.36 (s, 1H), 1.40 (s, 9H); ^13^C{^1^H} NMR (151 MHz, CDCl_3_) δ 186.5, 178.4, 175.6, 159.6, 136.5, 135.1, 128.6, 128.5, 128.3, 58.5, 58.1, 46.3, 36.5, 29.7, 14.6; FT-IR (ATR) ν 3325, 2966, 2854, 1784, 1670, 1454, 1178, 1092 cm^–1^; HPLC analysis ee (major diastereoisomer) 94% (Daicel Chiralpak IC column, 80:20 heptane/*iso*-propanol, 1.0 mL/min, λ = 210 nm; retention times, *t*_major_ = 13.8 min, *t*_minor_ = 19.0 min) at 25 °C; [α]_D_^rt^ = 4.4° (*c* = 0.56 in CHCl_3_); HRMS (ESI) *m*/*z* calcd for C_19_H_22_NO_3_ [M + H]^+^ 312.1594, found 312.1593.

#### (5*R*,6*R*)-4-(*tert*-Butyl)-6-ethyl-8-methyl-1-oxo-2-oxa-3-azaspiro[4.4]nona-3,7-diene-7-carbaldehyde (**3s**)

Purification of crude (dr 20:1) by silica gel chromatography (2:1 *n*-Hex/EA): pale yellow semisolid (21 mg, 79% yield); ^1^H NMR (600 MHz, CDCl_3_) δ 9.98 (s, 1H), 3.56–3.46 (m, 1H), 3.06 (ddd, *J* = 19.4 Hz, *J′* = 2.3 Hz, *J″* = 1.3 Hz, 1H), 2.97 (dt, *J* = 19.3 Hz, *J′* = 1.1 Hz, 1H), 2.19 (d, *J* = 1.5 Hz, 3H), 1.97 (dqd, *J* = 14.4 Hz, *J′* = 7.7 Hz, *J″* = 3.3 Hz, 1H), 1.73 (ddd, *J* = 14.3 Hz, *J′* = 10.6 Hz, *J′* = 7.1 Hz, 1H), 1.29 (s, 9H), 0.83 (t, *J* = 7.4 Hz, 3H); ^13^C{^1^H} NMR (151 MHz, CDCl_3_) δ 187.2, 180.1, 176.9, 157.6, 138.8, 54.9, 54.0, 48.1, 36.5, 29.7 (3C), 22.0, 14.2, 12.6; FT-IR (ATR) ν 3296, 2968, 2937, 2879, 1776, 1655, 1574, 1462, 1178, 1028, 883 cm^–1^; HPLC analysis ee 85% (Daicel Chiralpak IB column, 95:5 heptane/*iso*-propanol, 1.0 mL/min, λ = 240 nm; retention times, *t*_major_ = 11.8 min, *t*_minor_ = 13.0 min) at 25 °C; [α]_D_^rt^ = 60.9° (*c* = 0.64 in CHCl_3_); HRMS (ESI) *m*/*z* calcd for C_15_H_21_NNaO_3_ [M + Na]^+^ 286.1414, found 286.1413.

#### (5*R*,6*R*)-4-(*tert*-Butyl)-6-butyl-8-methyl-1-oxo-2-oxa-3-azaspiro[4.4]nona-3,7-diene-7-carbaldehyde (**3t**)

Purification of crude (dr 20:1) by silica gel chromatography (2:1 *n*-Hex/EA): pale yellow semisolid (26 mg, 74% yield); ^1^H NMR (600 MHz, CDCl_3_) δ 9.97 (s, 1H), 3.55 (d, *J* = 10.6 Hz, 1H), 3.07 (d, *J* = 20.0 Hz, 1H), 2.96 (d, *J* = 19.4 Hz, 1H), 2.19 (s, 3H), 1.85 (tdd, *J* = 11.2 Hz, *J*′ = 5.8 Hz, *J″* = 3.0 Hz, 1H), 1.70 (dtd, *J* = 14.5 Hz, *J′* = 10.5 Hz, *J″* = 4.3 Hz, 1H), 1.28 (s, 9H), 1.26–1.23 (m, 2H), 1.22–1.13 (m, 1H), 1.11–1.03 (m, 1H), 0.84 (t, *J* = 7.3 Hz, 3H); ^13^C{^1^H} NMR (151 MHz, CDCl_3_) δ 187.1, 180.1, 176.8, 157.4, 139.0, 54.9, 52.4, 48.1, 36.5, 30.3, 29.7, 28.5, 22.7, 14.1, 13.9; FT-IR (ATR) ν 3060, 2958, 2931, 2871, 1780, 1668, 1178, 881 cm^–1^; HPLC analysis ee 90% (Daicel Chiralpak IB column, 98:2 heptane/*iso*-propanol, 1.0 mL/min, λ = 240 nm; retention times, *t*_major_ = 13.1 min, *t*_minor_ = 14.3 min) at 25 °C; [α]_D_^rt^ = 40.6° (*c* = 0.88 in CHCl_3_); HRMS (ESI) *m*/*z* calcd for C_17_H_25_NNaO_3_ [M + Na]^+^ 314.1727, found 314.1727.

### Preparation of Acid Derivative **4**. (5*S*,6*R*)-8-Methyl-1-oxo-4,6-diphenyl-2-oxa-3-azaspiro[4.4]nona-3,7-diene-7-carboxylic Acid

To compound **3a** (64.0 mg, 0.19 mmol) in acetone (7 mL) and DMSO (2 mL) was dropwise added a mixture of NaClO_2_ (86.0 mg, 0.95 mmol) and KH_2_PO_4_ (129.0 mg, 0.95 mmol) dissolved in H_2_O (6 mL). After the mixture was stirred for 3 h, the solvent was removed under reduced pressure, and the residue was extracted with ethyl acetate (4 × 25 mL). The combined organic layer was washed with H_2_O (1 × 50 mL) and a brine solution (1 × 50 mL). The organic layer was dried over anhydrous Na_2_SO_4_, and the solvent was removed *invacuo*. The crude product was purified by silica gel flash chromatography in a 20:1 DCM/MeOH eluent to give **4** as a white solid (58 mg, 88% yield): mp 195.7 °C (ethyl acetate/*n*-heptane); ^1^H NMR (600 MHz, CDCl_3_) δ 7.76–7.73 (m, 2H), 7.58–7.55 (m, 1H), 7.52–7.49 (m, 2H), 7.30–7.26 (m, 3H), 7.05–7.02 (m, 2H), 4.95 (s, 1H), 3.27 (d, *J* = 19.2 Hz, 1H), 3.19 (d, *J* = 19.2 Hz, 1H), 2.34 (s, 3H); ^13^C{^1^H} NMR (151 MHz, CDCl_3_) δ 178.5, 168.9, 167.3, 156.9, 136.3, 132.2, 129.71 (2C), 128.75 (2C), 128.5, 127.9 (2C), 127.1, 126.9, 126.6 (2C), 61.3, 56.3, 47.8, 16.6; FT-IR (KBr) ν 3034, 2923, 2660, 1793, 1649, 1428, 1362, 1266, 1048 cm^–1^; HRMS (ESI) *m*/*z* calcd for C_21_H_17_NNaO_4_ [M + Na]^+^ 370.1050, found 370.1046; [α]_D_^rt^ = −92.3° (*c* = 1.92° in MeOH); HPLC analysis ee 88% (Daicel Chiralpak IG column, 70:30 heptane/*iso*-propanol, 1.0 mL/min, λ = 198 nm; retention times, *t*_major_ = 5.9 min, *t*_minor_ = 9.0 min) at 25 °C.

### Preparation of Derivative **5**. (4*R*,5*R*)-4-Benzoyl-5-(4-bromophenyl)-2-methylcyclopent-1-ene-1-carbaldehyde

A solution of Mo(CO)_6_ (63.4 mg, 0.24 mmol) in CH_3_CN (3 mL) was refluxed using an oil bath for 3 h. Then, after the mixture was cooled to room temperature, a mixture of the diastereoisomers of **3b** (50.0 mg, 0.12 mmol) and H_2_O (0.09 mL, 4.8 mmol) was added. The reaction mixture was stirred overnight at the same temperature. The solvent was then removed *in vacuo*, and the crude product was purified by silica gel flash chromatography with a 3:1 *n*-hexane/ethyl acetate eluent to give **5** as a single diastereoisomer as a yellow semisolid (26 mg, 59% yield): ^1^H NMR (600 MHz, CDCl_3_) δ 9.89 (s, 1H), 7.80–7.79 (m, 2H), 7.58–7.54 (m, 1H), 7.44–7.39 (m, 4H), 7.04–7.02 (m, 2H), 4.38 (s, 1H), 3.91 (dt, *J* = 8.5 Hz, *J*′ = 4.2 Hz, 1H), 3.15–3.05 (m, 2H), 2.30 (s, 3H); ^13^C{^1^H} NMR (151 MHz, CDCl_3_) δ 199.2, 187.0, 161.2, 142.7, 138.5, 135.5, 133.6, 131.9 (2C), 129.2 (2C), 128.9 (2C), 128.9 (2C), 120.8, 53.2, 51.9, 42.2, 14.5; FT-IR (KBr) ν 3055, 2920, 1664, 1580, 1449, 1335, 1186 cm^–1^; HRMS (ESI) *m*/*z* calcd for C_20_H_17_BrNaO_2_ [M + Na]^+^ 391.0304, found 391.0303; [α]_D_^rt^ = −159.6° (*c* = 1.20 in CHCl_3_); HPLC analysis ee 84% (Daicel Chiralpak IA column, 95:5 heptane/*iso*-propanol, 1.0 mL/min, λ = 190 nm; retention times, *t*_minor_ = 19.8 min, *t*_major_ = 21.3 min) at 25 °C.

### One-Pot Preparation of Derivative **5**. (4*R*,5*R*)-4-Benzoyl-5-(4-bromophenyl)-2-methylcyclopent-1-ene-1-carbaldehyde

To a screw cap septum vial containing EtOAc (2 mL) was added (*S*)-DPP-TMS **I** (0.048 mmol, 0.20 equiv), followed by the addition of *trans*-4-bromocinnamaldehyde **2b** (51 mg, 0.24 mmol, 1.0 equiv) and isoxazolone derivative **3a** (72 mg, 0.36 mmol, 1.5 equiv). Then, Pd_2_(dba)_3_ (11 mg, 0.012 mmol, 0.05 equiv) was added. After full conversion was reached, the solvent was removed and to the crude reaction mixture in MeCN (2 mL) were added Mo(CO)_6_ (190 mg, 0.72 mmol, 3 equiv) and H_2_O (0.17 mL, 9.6 mmol). The reaction mixture was refluxed using an oil bath for 3 h and then stirred at room temperature for an additional 17 h. Then, the solvent was removed *in vacuo*, and the crude product was purified by silica gel flash chromatography with a 3:1 *n*-hexane/ethyl acetate eluent to give **5** as one diastereoisomer as a yellow semisolid (36 mg, 41% yield): [α]_D_^rt^ = −170.6° (*c* = 1.15 in CHCl_3_); HPLC analysis ee 84% (Daicel Chiralpak IA column, 95:5 heptane/*iso*-propanol, 1.0 mL/min, λ = 190 nm; retention times, *t*_minor_ = 19.8 min, *t*_major_ = 21.3 min) at 25 °C.

### Preparation of Derivative **6**. (4*R*,5*R*)-4-Benzoyl-5-(4-bromophenyl)-2-methylcyclopent-1-ene-1-carboxylic Acid

The title compound was prepared according to the procedure for the preparation of compound **4**. The crude product was purified by silica gel flash chromatography in a 20:1 DCM/MeOH eluent to give **6** as a yellow semisolid (38 mg, 73% yield): ^1^H NMR (600 MHz, CDCl_3_) δ 7.79–7.77 (m, 2H), 7.57–7.53 (m, 1H), 7.44–7.38 (m, 4H), 7.03–7.00 (m, 2H), 4.42 (s, 1H), 3.82 (dt, *J* = 8.8 Hz, *J*′ = 4.6 Hz, 1H), 3.06–2.95 (m, 1H), 2.23 (s, 3H); ^13^C{^1^H} NMR (151 MHz, CDCl_3_) δ 199.3, 169.8, 158.5, 143.4, 135.7, 133.5, 131.9 (2C), 129.2 (2C), 128.9 (2C), 128.8 (2C), 128.3, 120.7, 54.8, 52.0, 42.7, 16.7; FT-IR (KBr) ν 3058, 3019, 2642, 1676, 1643, 1577, 1443, 1344, 1242 cm^–1^; HRMS (ESI) *m*/*z* calcd for C_20_H_17_BrNaO_3_ [M + Na]^+^ 409.0238, found 409.0261; [α]_D_^rt^ = −161.0° (*c* = 1.00 in CHCl_3_); HPLC analysis ee 84% (Daicel Chiralpak IG column, 70:30 heptane/*iso*-propanol, 1.0 mL/min, λ = 200 nm; retention times, *t*_minor_ = 13.2 min, *t*_major_ = 15.6 min) at 25 °C.

### Scale-up Asymmetric Preparation of **3a**

To a screw cap septum vial containing EtOAc (6 mL) was added catalyst (*S*)-DPP-TMS **I** (0.024 mmol, 0.20 equiv), followed by addition of (*E*)-cinnamaldehyde **2a** (132 mg, 1.0 mmol, 1.0 equiv) and isoxazolone derivative **1a** (318 mg, 1.5 mmol, 1.5 equiv). Then, Pd_2_(dba)_3_ (46 mg, 0.05 mmol, 0.05 equiv) was added. After full conversion was reached (TLC monitoring), the solvent was removed *invacuo* and the crude reaction mixture (dr 2:1) was purified through silica gel flash chromatography (*n*-Hex/EtOAc), affording the desired spirocyclic compound as two separable diastereoisomers: **3a-major**: 149 mg, 45% yield, 86% ee. **3a-minor**: 89 mg, 27% yield, 99% ee.

## Data Availability

The data underlying this study are available in the published article and its Supporting Information.

## Supporting Information Available

X-ray data for compounds **3e-major**, **3h-major**, **3r-major**, and **6** and chiral HPLC chromatograms of **3a**–**t** and **4**–**6** (PDF)
FAIR data, including the primary NMR FID files, for compounds **1a**–**c**, **2b**–**n**, **3a**–**t**, and **4**–**6** (ZIP)
