# Supplementary material for: Enantioselective Synthesis of Spirocyclic Isoxazolones Using a Conia-Ene Type Reaction
Source: J Org Chem. 2025 Mar 5;90(10):3615–27. doi: 10.1021/acs.joc.4c02921 (PMC11915384; doi:10.1021/acs.joc.4c02921)
Supplement: Supplementary file 1 — jo4c02921_si_001.pdf [file jo4c02921_si_001.pdf]

Enantioselective Synthesis of Spirocyclic Isoxazolones Using Conia-Ene Type Reaction

Martin Kamlar,[a] Salil Putatunda,[a] Ivana Císařová,[b] and Jan Veselý[a]*

[a] Department of Organic Chemistry, Faculty of Science, Charles University, Hlavova 2030, 128 43 Praha 2, Czech Republic.

[b] Department of Inorganic Chemistry, Faculty of Science, Charles University, Hlavova 2030, 128 43 Praha 2, Czech Republic.

E-mail: jxvesely@natur.cuni.cz

Table of Content

General Information 2

Preparation of the Starting Materials 3

Preparation of isoxazolones 3

General procedure for the synthesis of (*E*)-α,β-Unsaturated Aldehydes 3

NMR Spectral Data 5

X-Ray section 47

HPLC Data 81

General Information

Chemicals and solvents were either purchased (puriss p.A.) from commercial suppliers or purified by standard techniques. For thin-layer chromatography (TLC), silica gel plates Merck 60 F254 were used, and compounds were visualized by irradiation with UV light and/or by treatment with a solution of phosphomolybdenic acid (25 g), $Ce(SO_4)_2{\cdot}H_2O$ (10 g), conc. H_2SO_4 (60 mL), and H_2O (940 mL) followed by heating. Column chromatography was performed using silica gel Merck 60 (particle size 0.040–0.063 mm). ^{1}H NMR, ^{13}C NMR, 2D NMR were recorded with a Bruker DPX600 NMR. Chemical shifts (δ) are reported in ppm relative to residual solvent signals ($CHCl_3$, 7.26 ppm for ^{1}H NMR; $CDCl_3$, 77.2 ppm for ^{13}C NMR). High-resolution mass spectra were recorded on an LCQ Fleet spectrometer using a Bruker Compact QTOF-MS controlled by the Compass 1.9 Control software to measure the ESI high-resolution mass spectrums. The monoisotopic mass values were calculated using Data analysis software v 4.4. The analysis was conducted in the positive ion mode at a scan range from m/z 50 to 1000, and nitrogen was used as nebulizer gas at a pressure of 4 psi and flow of 3 l/min for the dry gas. The capillary voltage and temperature were set at 4500 V and 220 °C, respectively. Optical rotations were performed on an AU-Tomatica polarimeter, Autopol III. IR DRIFT spectras were recorded with Nicolet AVATAR 370 FT-IR in cm^{-1}. The HPLC analysis were performed on a LC20AD Shimadzu liquid chromatograph with SPDM20A diode array detector with columns Daicel Chiralpak.

Preparation of the Starting Materials

Preparation of isoxazolones

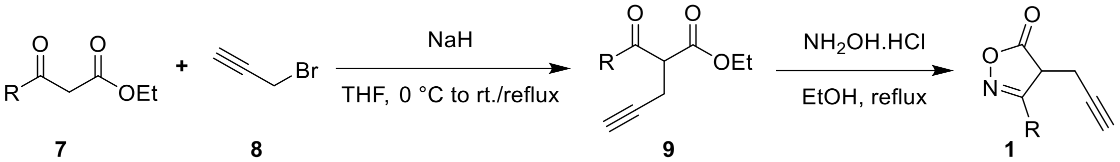

7 **8** **9** **1**

Isoxazolones **1a-c** were prepared according to the published two-step procedure.[1]
Spectral data of **1a** is consistent with data published in the literature.[1b] **1a:** keto form: ^{1}H NMR (600 MHz, $CDCl_3$) δ 7.70 – 7.66 (m, 2H), 7.58 – 7.48 (m, 3H), 3.98 (t, *J* = 5.4 Hz, 1H), 3.01 (ddd, *J* = 17.2 Hz, *J′* = 5.4 Hz, *J′′* = 2.7 Hz, 1H), 2.85 (ddd, *J* = 17.2 Hz, *J′* = 5.4 Hz, *J′′* = 2.7 Hz, 1H), 2.05 (t, *J* = 2.7 Hz, 1H) ppm; ^{13}C {1H} NMR (151 MHz, $CDCl_3$) δ 176.6, 165.2, 132.2, 129.5 (2C), 127.3, 127.1 (2C), 76.4, 72.9, 44.4, 19.2 ppm. enol form: ^{1}H NMR (600 MHz, $CDCl_3$) δ 7.70 – 7.66 (m, 2H), 7.58 – 7.48 (m, 3H), 3.31 (d, *J* = 2.7 Hz, 2H), 2.06 (t, *J* = 2.7 Hz, 1H) ppm; ^{13}C {1H} NMR (151 MHz, $CDCl_3$) δ 172.3, 163.2, 132.0, 129.6 (2C), 127.9 (2C), 126.9, 99.5, 80.2, 69.6, 12.8 ppm. MS (ESI) m/z calcd for $C_{12}H_{10}NO_2$ $[M+H]^+$ = 200.1, found: 200.1.
Spectral data of **1b** is consistent with data published in the literature.[2] **1b:** keto form: ^{1}H NMR (600 MHz, $CDCl_3$) δ 3.44 (t, *J* = 5.9 Hz, 1H), 2.79 (dd, *J* = 5.9 Hz, *J′* = 2.7 Hz, 2H), 2.18 (s, 3H), 2.12 (t, *J* = 2.7 Hz, 1H) ppm; ^{13}C {1H} NMR (151 MHz, $CDCl_3$) δ 176.57, 165.43, 76.87, 72.54, 46.42, 17.41, 13.84 ppm. enol form: ^{1}H NMR (600 MHz, $CDCl_3$) δ 3.16 (d, *J* = 2.7 Hz, 2H), 2.29 (s, 3H), 2.03 (t, *J* = 2.7 Hz, 1H) ppm; ^{13}C {1H} NMR (151 MHz, $CDCl_3$) δ 172.8, 162.0, 96.0, 79.9, 69.4, 11.7, 11.0 ppm; MS (ESI) m/z calcd for $C_7H_8NO_2$ $[M+H]^+$ = 138.1, found: 138.1.
3-(tert-Butyl)-4-(prop-2-yn-1-yl)isoxazol-5(*4H*)-one (1c) was prepared according to the same two-step procedure.[1] The crude product was purified by silica gel flash chromatography with n-hexane/ethyl acetate (2:1) as eluent to give **1c** as pale red solid in 21% yield (0.9 g).^{1}H NMR (600 MHz, $CDCl_3$) δ = 3.51 (t, *J* = 4.7 Hz, 1H), 2.91 (qdd, *J* = 17.4 Hz, *J´* = 4.7 Hz, *J´´* = 2.6 Hz, 2H), 2.12 (t, *J* = 2.6 Hz, 1H), 1.32 (s, 9H) ppm; ^{13}C {1H} NMR (151 MHz, $CDCl_3$) δ = 177.4, 173.8, 77.2, 73.0, 45.4, 35.5, 28.2, 19.5 ppm; HRMS (ESI) m/z calcd for $C_{10}H_{13}NO_2Na$ [M+Na] = 202.0838, found: 202.0839.

General procedure for the synthesis of (*E*)-α,β-Unsaturated Aldehydes

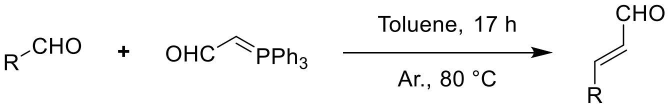

Enals were prepared following the reported procedure.[3] Spectral data of enal **2b** are consistent with data published in the literature.[4] **2b:** **^{1}H** NMR (600 MHz, $CDCl_3$) δ 9.69 (d, *J* = 7.6 Hz, 1H), 7.55 (d, *J* = 8.5 Hz, 2H), 7.43 – 7.38 (m, 3H), 6.68 (dd, *J* = 16.0, 7.6 Hz, 1H) ppm. ^{13}C {1H} NMR (151 MHz, $CDCl_3$) δ 193.7, 151.4, 133.2, 132.7 (2C), 130.1 (2C), 129.3, 126.0 ppm; MS (ESI) m/z calcd for C_9H_7BrNaO $[M+Na]^+$ = 232.9, found: 232.9.

---

[1] (a); Chang, M. Y.; Yu-Chieh Cheng, Y. Ch.; Lu, Y. J. *Org. Lett*. **2015**, *17*, 1264-1267; (b)Galenko, E. E.; Novikov, M. S.; Shakirova, F. M.; Shakirova, J. R.; Kornyakov, I. V.; Bodunov, V. A.; Khlebnikov, A. F. *J. Org. Chem*. **2019**, *84*, 3524–3536.
[2] Krogsgaard-Larsen, P.; Christensen, S. B.; Hjeds, H. *Acta Chem. Scand*. **1973**, *27*, 2802-2812.
[3] F. Hirayama, H. Koshio, N. Katayama, H. Kurihara, Y. Taniuchi, K. Sato, N. Hisamichi, Y. Sakai-Moritani, T. Kawasaki, Y. Matsumoto, and I. Yanagisawa, *Bioorg. Med. Chem.* **2002**, *10*, 1509-1523.
[4] H. Huang, C. Yu, X. Li, Y. Zhang, Y. Zhang, X. Chen, P. S. Mariano, H. Xie and W. Wang, *Angew.Chem.Int.Ed.* **2017**, *56*, 8201-8205.

Sprectral data of **2c-g** are consistent with data published in the literature.[5]

2c: ^{1}H NMR (600 MHz, $CDCl_3$) δ 9.76 (d, *J* = 7.5 Hz, 1H), 7.73 (d, *J* = 8.4 Hz, 2H), 7.66 (d, *J* = 8.4 Hz, 2H), 7.48 (d, *J* = 16.1 Hz, 1H), 6.77 (dd, *J* = 16.0 Hz, *J´* = 7.5 Hz, 1H) ppm; ^{13}C {1H} NMR (151 MHz, $CDCl_3$) δ = 193.0, 149.6, 138.3, 133.0 (2C), 131.3, 128.9 (2C), 118.3, 114.4 ppm; ; MS (EI) m/z calcd for $C_{10}H_7NO$ $[M]^+$ = 157.1, found: 157.1.

2e: ^{1}H NMR (600 MHz, $CDCl_3$) δ 9.68 (d, *J* = 7.8 Hz, 1H), 7.48 – 7.41 (m, 3H), 7.23 (d, *J* = 8.3 Hz, 2H), 6.68 (dd, *J* = 15.9 Hz, *J´* = 7.7 Hz, 1H), 2.39 (s, 3H) ppm; ^{13}C {1H} NMR (151 MHz, $CDCl_3$) δ 194.1, 153.3, 142.3, 131.6, 130.2 (2C), 128.9 (2C), 128.0, 21.9 ppm; MS (ESI) m/z calcd for $C_{10}H_{10}NaO$ $[M+Na]^+$ = 169.1, found: 169.1.

2f: ^{1}H NMR (600 MHz, $CDCl_3$) δ 9.69 (d, *J* = 7.7 Hz, 1H), 7.44 (d, *J* = 15.9 Hz, 1H), 7.38 – 7.36 (m, 2H), 7.32 (t, *J* = 7.8 Hz, 1H), 7.26 – 7.24 (m, 1H), 6.71 (dd, *J* = 15.9 Hz, *J´* = 7.7 Hz, 1H), 2.39 (s, 3H) ppm. ^{13}C {1H} NMR (151 MHz, $CDCl_3$) δ 194.1, 153.4, 139.2, 134.3, 132.5, 129.4, 129.3, 128.8, 126.1, 21.6 ppm; MS (ESI) m/z calcd for $C_{10}H_{11}O$ $[M+H]^+$ = 147.1, found: 147.1.

2g: ^{1}H NMR (600 MHz, $CDCl_3$) δ 9.74 (d, *J* = 7.7 Hz, 1H), 7.78 (d, *J* = 15.8 Hz, 1H), 7.59 (d, *J* = 7.5 Hz, 1H), 7.34 (td, *J* = 7.3 Hz, *J´* = 1.4 Hz, 1H), 7.27 – 7.25 (m, 2H), 6.67 (dd, *J* = 15.8 Hz, *J´* = 7.7 Hz, 1H), 2.49 (s, 3H) ppm; ^{13}C {1H} NMR (151 MHz, $CDCl_3$) δ 193.9, 150.3, 138.0 132.90, 131.2, 131.1, 129.7, 126.9, 126.7, 19.8 ppm; MS (ESI) m/z calcd for $C_{10}H_{11}O$ $[M+H]^+$ = 147.1, found: 147.1.

Spectral data of **2m** are consistent with data published in the literature.[6] ^{1}H NMR (600 MHz, $CDCl_3$) δ δ 9.49 (d, *J* = 7.9 Hz, 1H), 6.83 (dt, *J* = 15.7 Hz, *J´* =6.7 Hz, 1H), 6.12 (dd, *J* = 15.7 Hz, *J´* = 7.9 Hz, 1H), 5.82 – 5.75 (m, 1H), 5.08 – 5.01 (m, 2H), 2.46 – 2.41 (m, 2H), 2.29 – 2.24 (m, 2H) ppm; ^{13}C {1H} NMR (151 MHz, $CDCl_3$) δ 194.1, 157.8, 136.7, 133.4, 116.1, 31.9, 31.9 ppm; MS (EI) m/z calcd for $C_7H_{10}O$ $[M]^+$ = 110.1, found: 110.1.

Sprectral data of **2n** are consistent with data published in the literature.[7] ^{1}H NMR (600 MHz, $CDCl_3$) δ δ 9.72 (d, *J* = 7.7 Hz, 1H), 6.91 (dd, *J* = 16.0 Hz, *J´* = 7.6 Hz, 1H), 6.69 (d, *J* = 15.9 Hz, 1H), 4.24 (q, *J* = 7.2 Hz, 2H), 1.28 (t, *J* = 7.2 Hz, 3H) ppm; ^{13}C {1H} NMR (151 MHz, $CDCl_3$) δ 192.6, 164.9, 140.3, 139.5, 61.7, 14.1 ppm; MS (EI) m/z calcd for $C_6H_8O_3$ $[M]^+$ = 128.0, found: 128.0.

[5] Battistuzzi, G.; Cacchi, S.; Fabrizi, G. *Org. Lett.* **2003**, *5*, 777-780.

[6] I. Coldham, A. J. M. Burrell, H. D. S. Guerrand, and N. Oram, *Org. Lett.* **2011**, *13*, 1267-1269.

[7] S. Putatunda, J. V. Alegre-Requena, M. Meazza, M. Franc, D. Rohal’ová, P. Vemuri, I. Císařová, R. P. Herrera, R. Rios, and J. Veselý, *Chem.Sci*. **2019**, *10*, 4107–411.

NMR Spectral Data

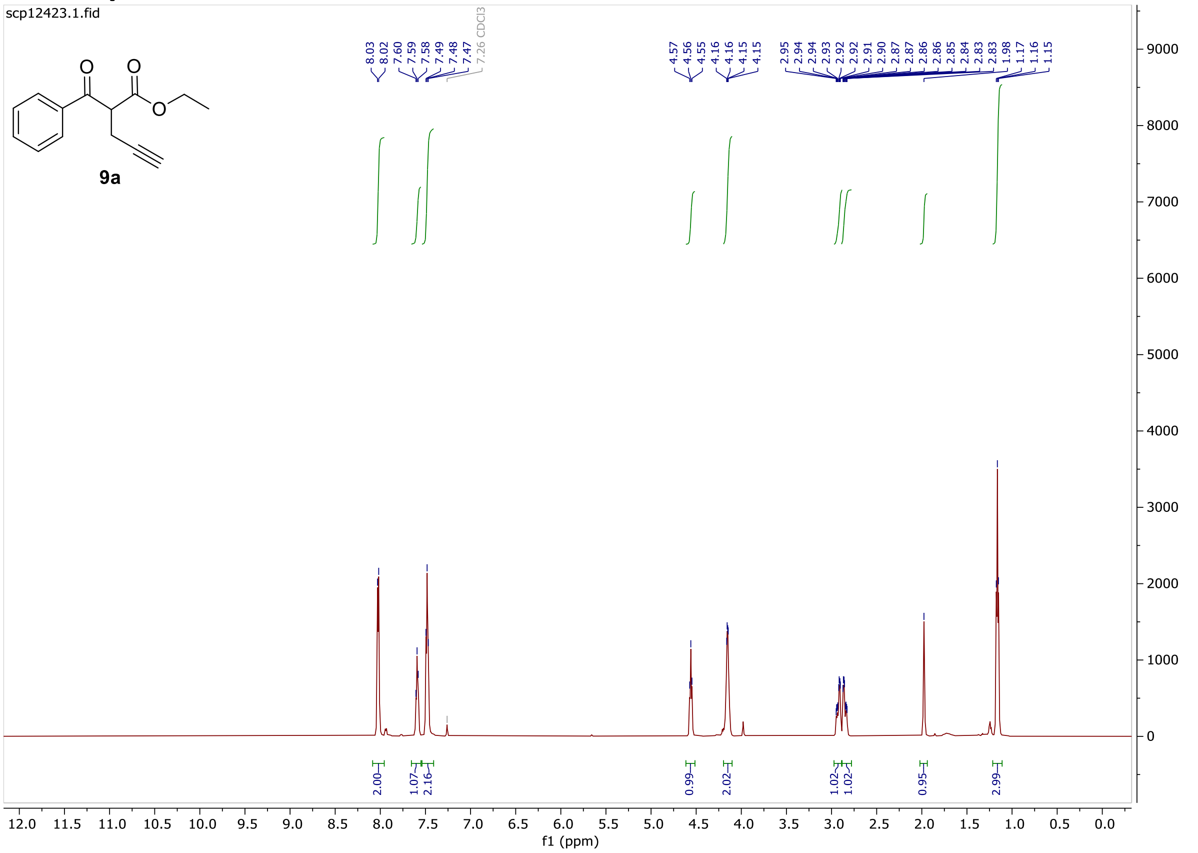

^{1}H NMR spectrum of **9a** ($CDCl_3$, 600 MHz).

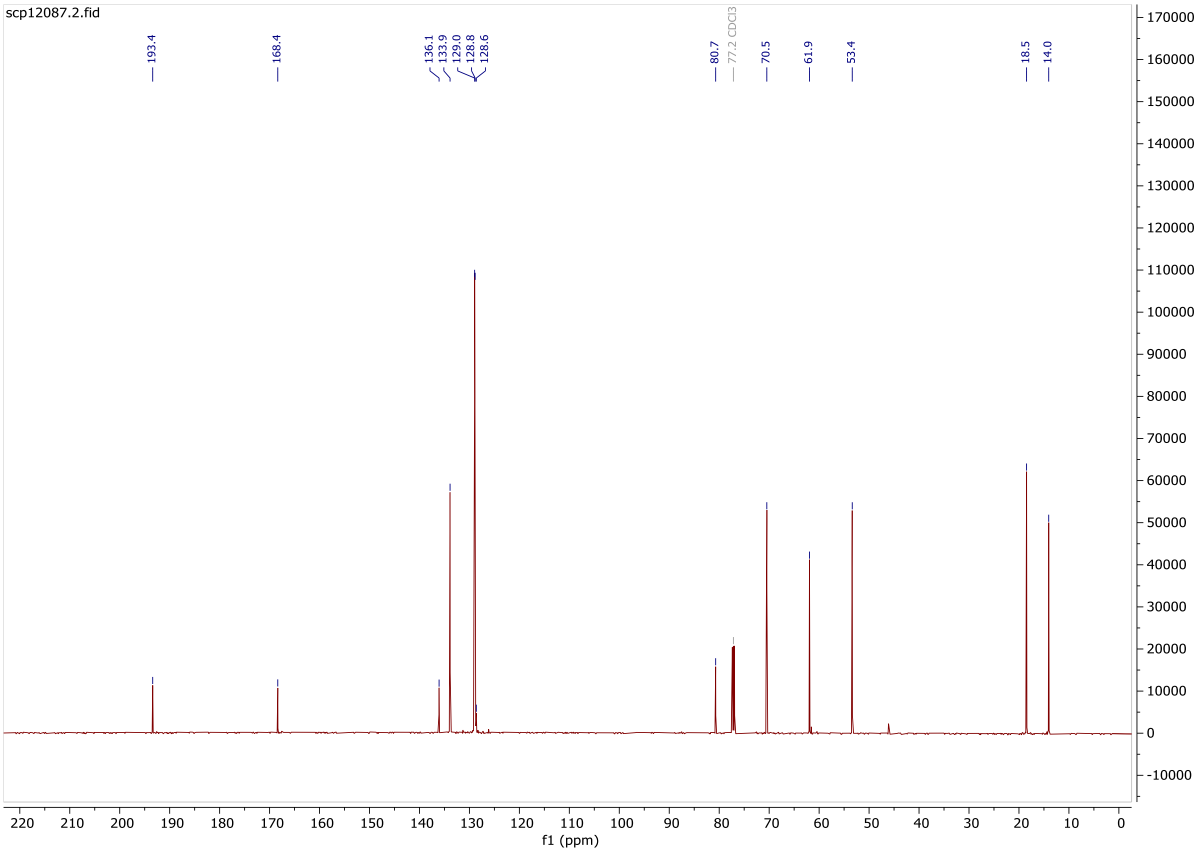

^{13}C {1H} NMR spectrum of **9a** ($CDCl_3$, 151 MHz).

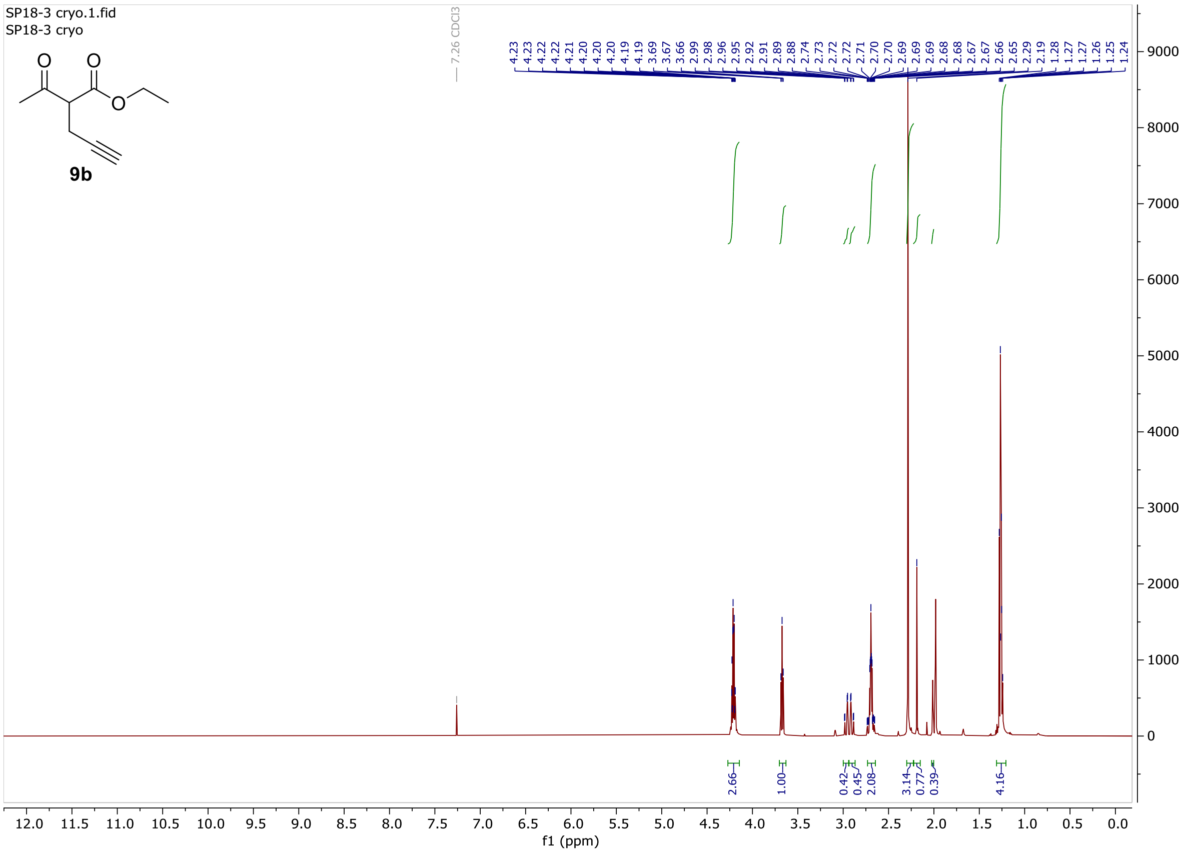

^{1}H NMR spectrum of **9b** ($CDCl_3$, 600 MHz).

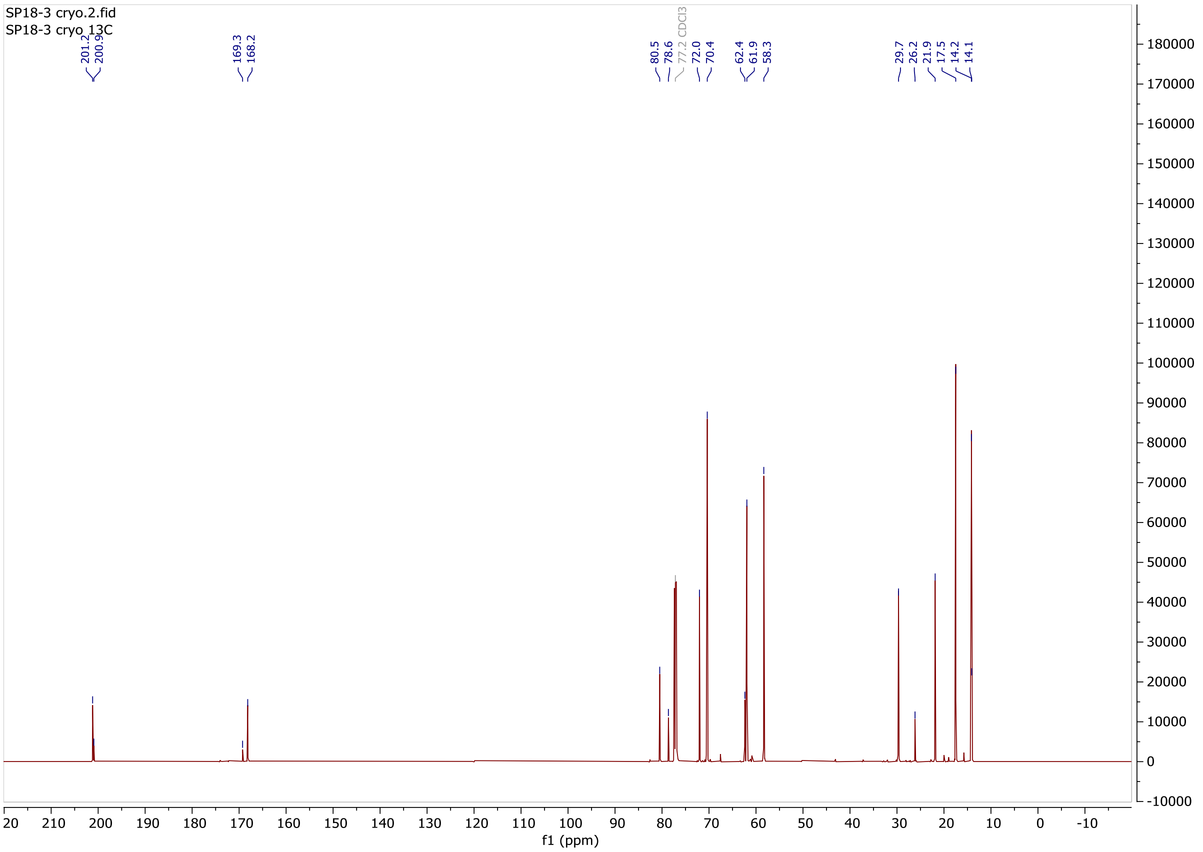

^{13}C {1H} NMR spectrum of **9b** ($CDCl_3$, 151 MHz).

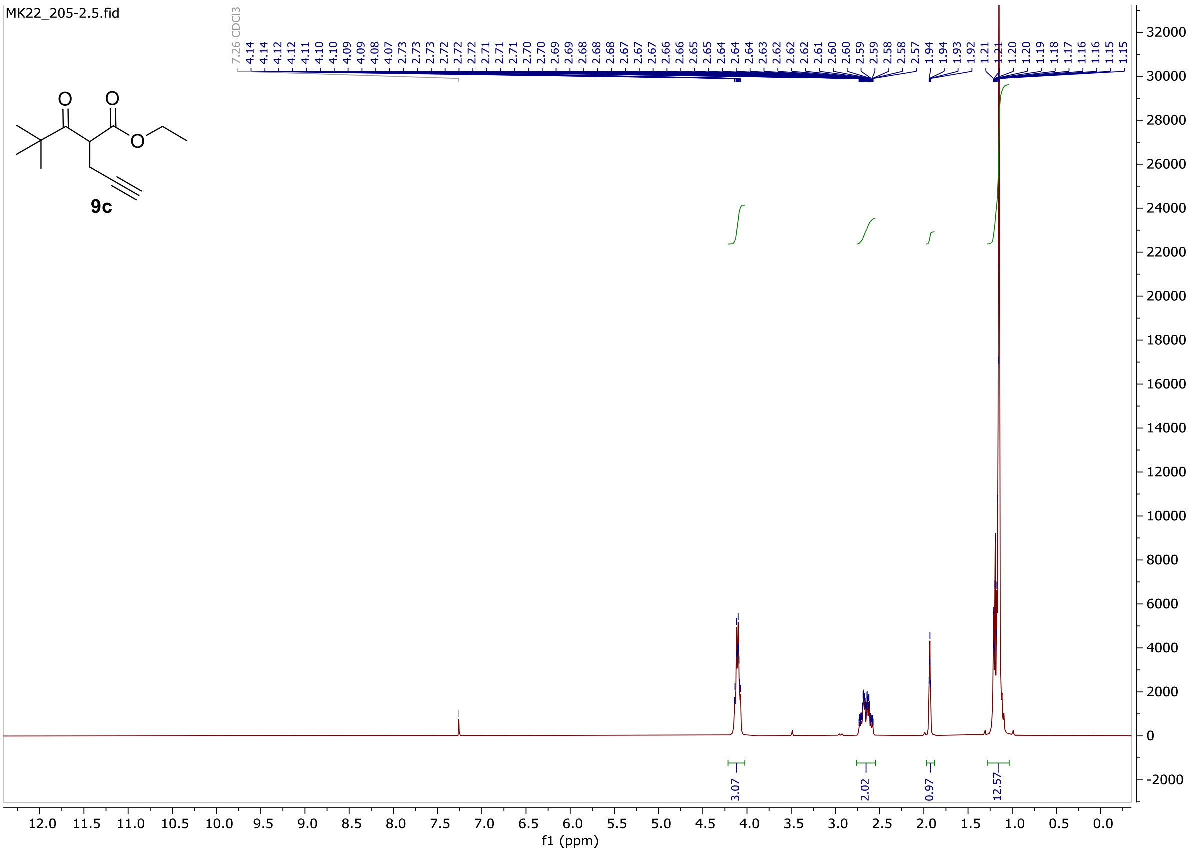

^{1}H NMR spectrum of **9c** ($CDCl_3$, 600 MHz).

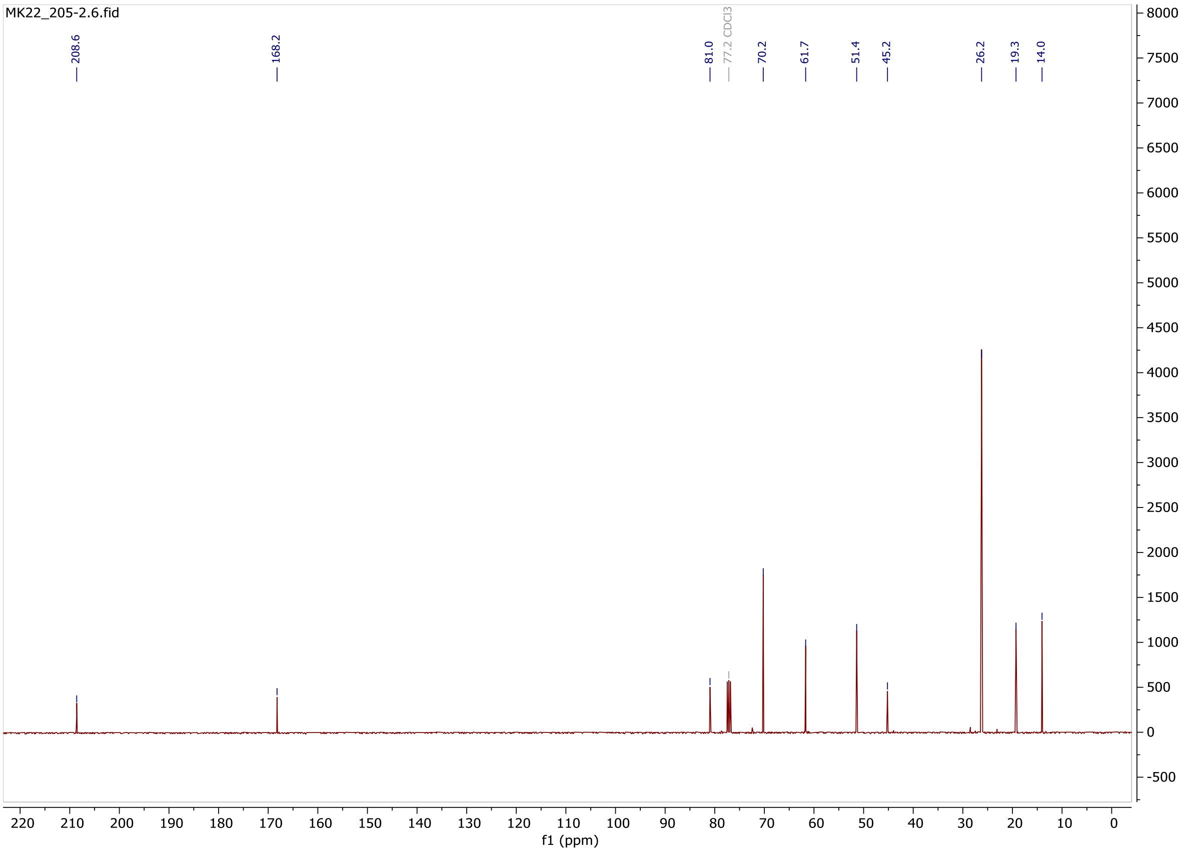

^{13}C {1H} NMR spectrum of **9c** ($CDCl_3$, 151 MHz).

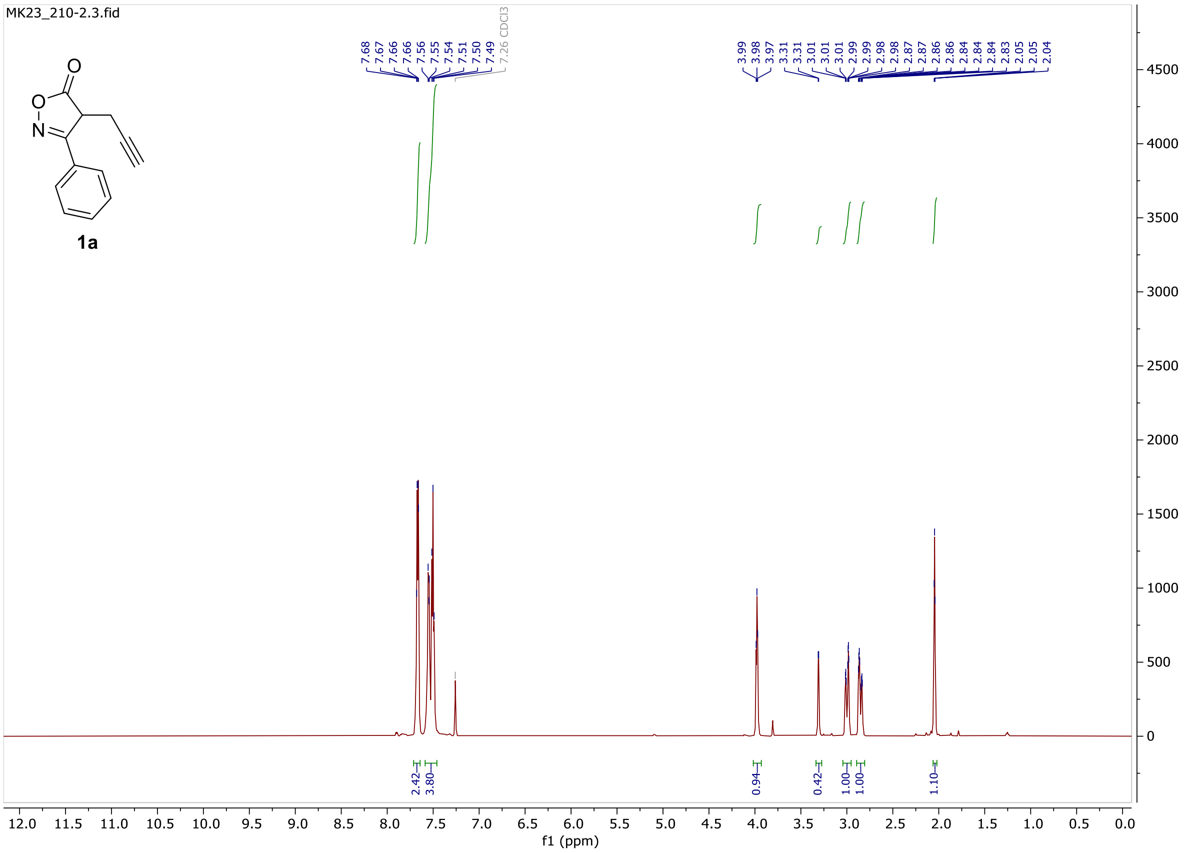

^{1}H NMR spectrum of **1a** ($CDCl_3$, 600 MHz).

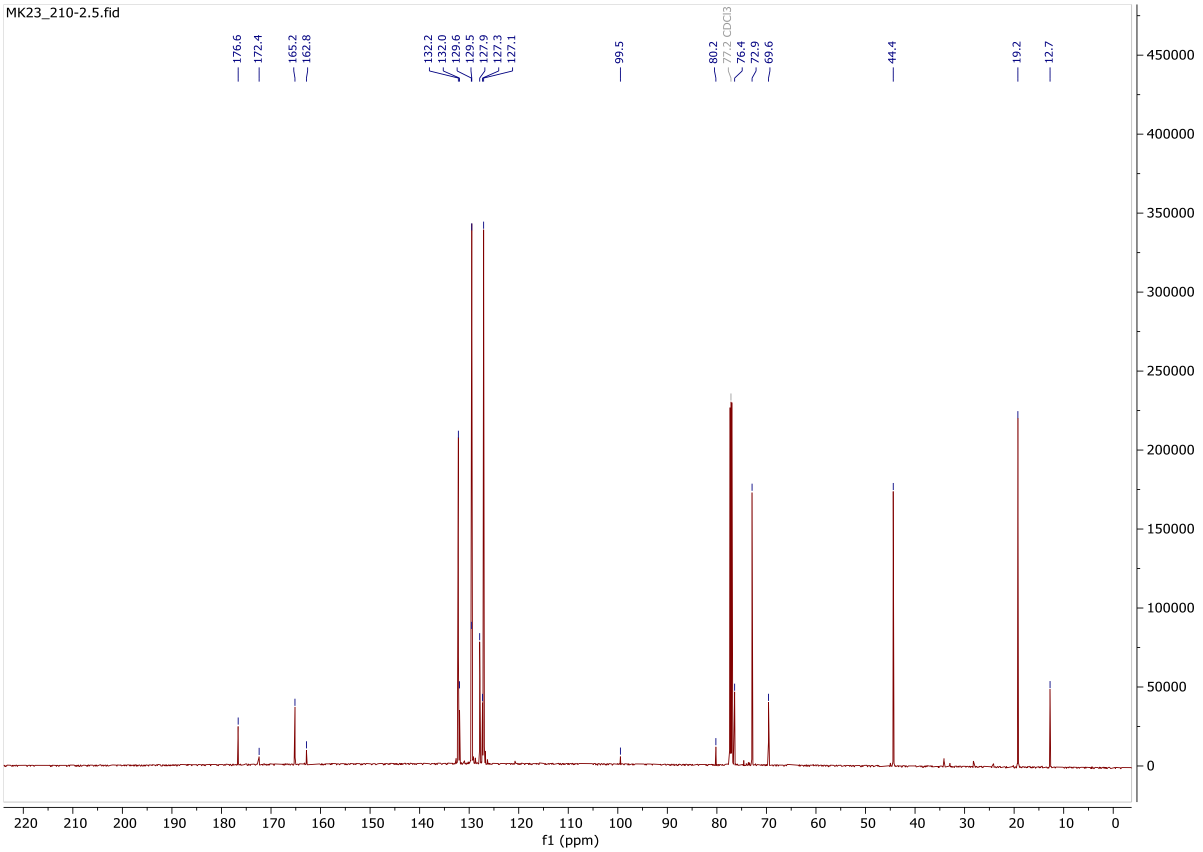

^{13}C {1H} NMR spectrum of **1a** ($CDCl_3$, 151 MHz).

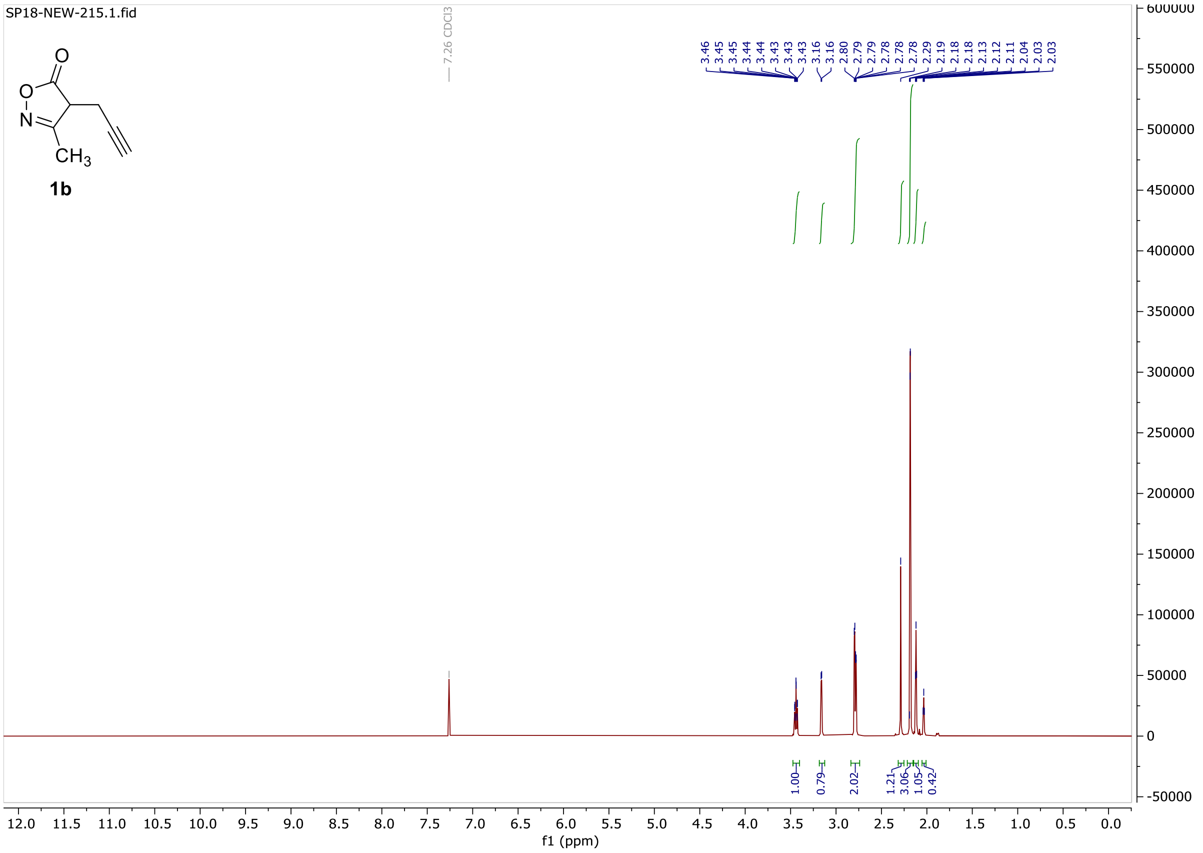

^{1}H NMR spectrum of **1b** ($CDCl_3$, 600 MHz).

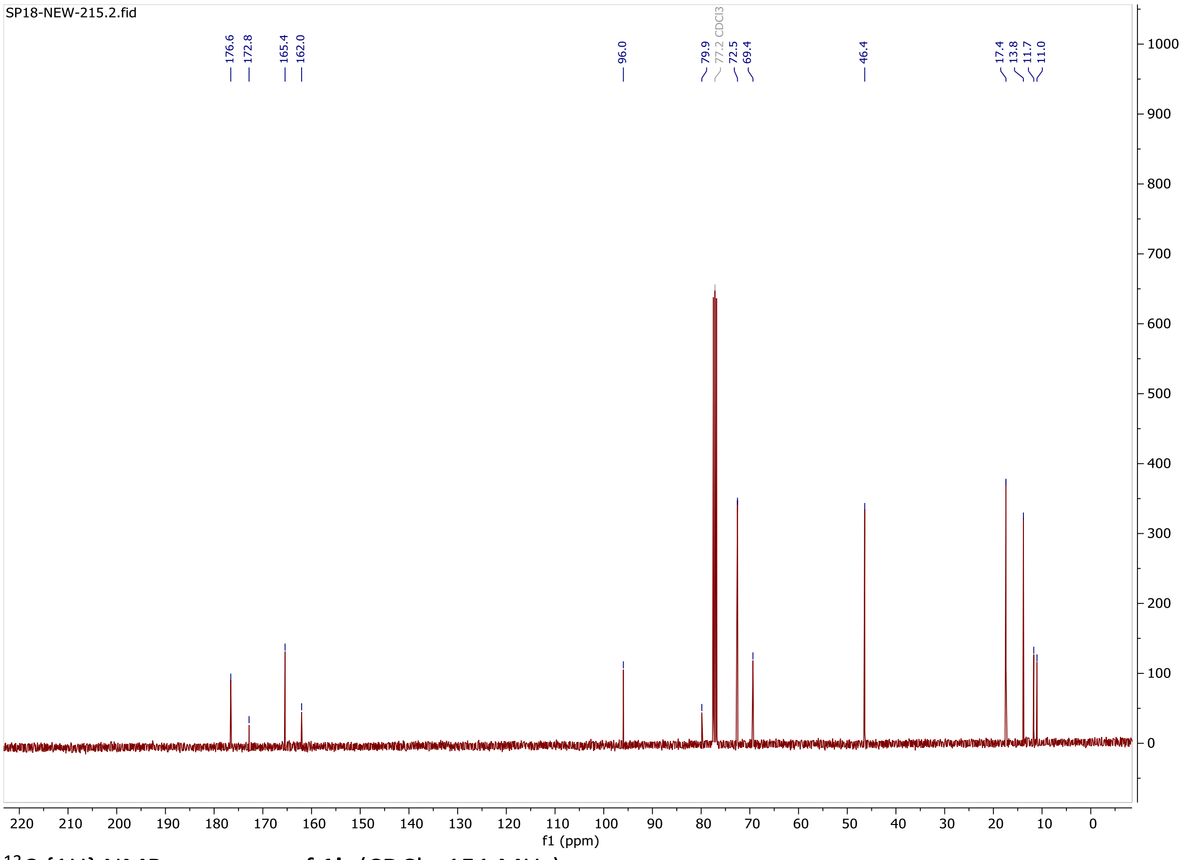

^{13}C {1H} NMR spectrum of **1b** ($CDCl_3$, 151 MHz).

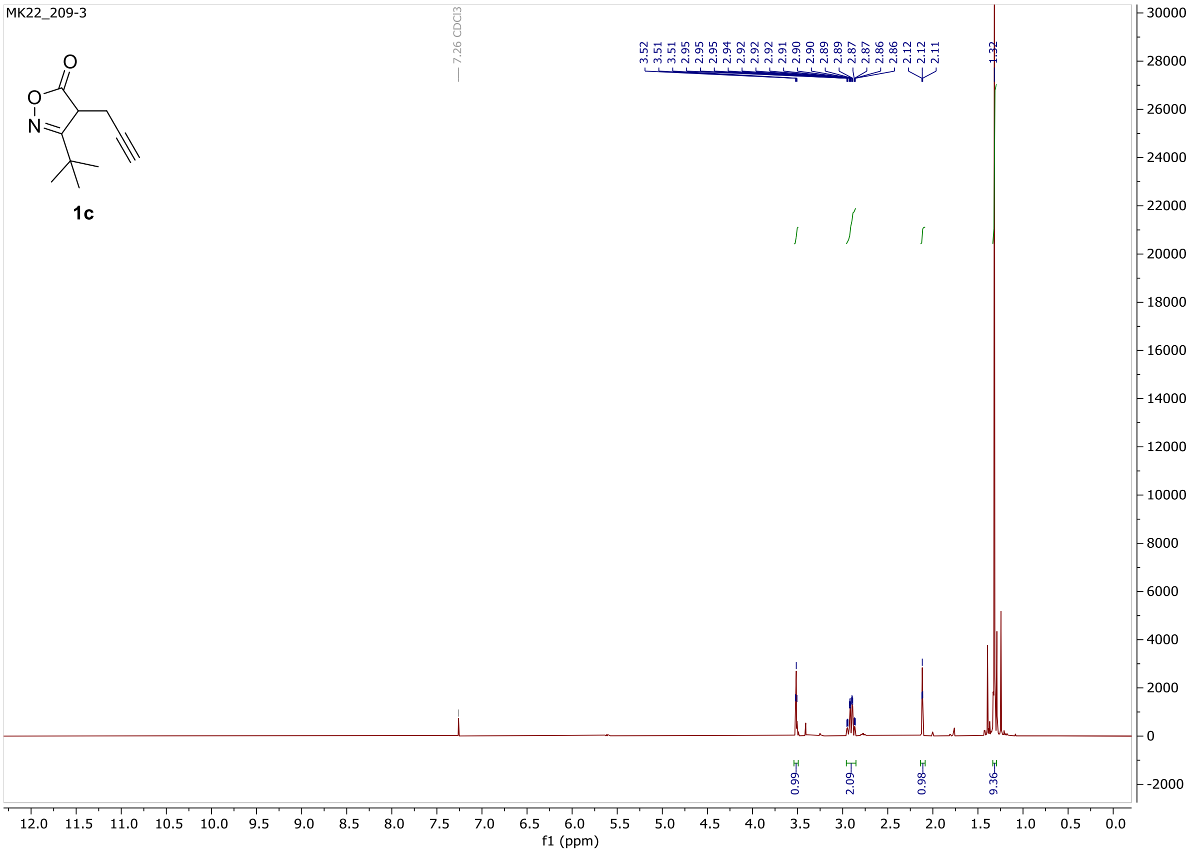

^{1}H NMR spectrum of **1c** ($CDCl_3$, 600 MHz).

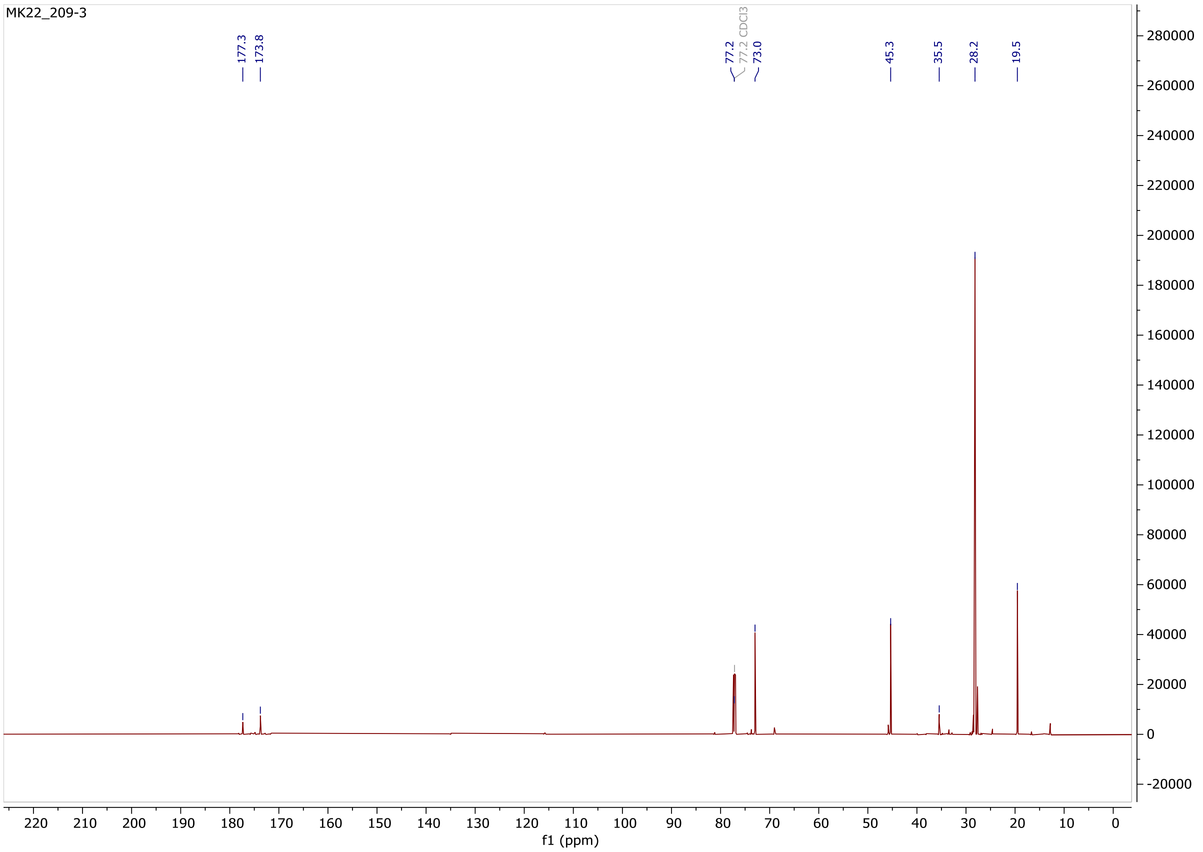

^{13}C {1H} NMR spectrum of **1c** ($CDCl_3$, 151 MHz).

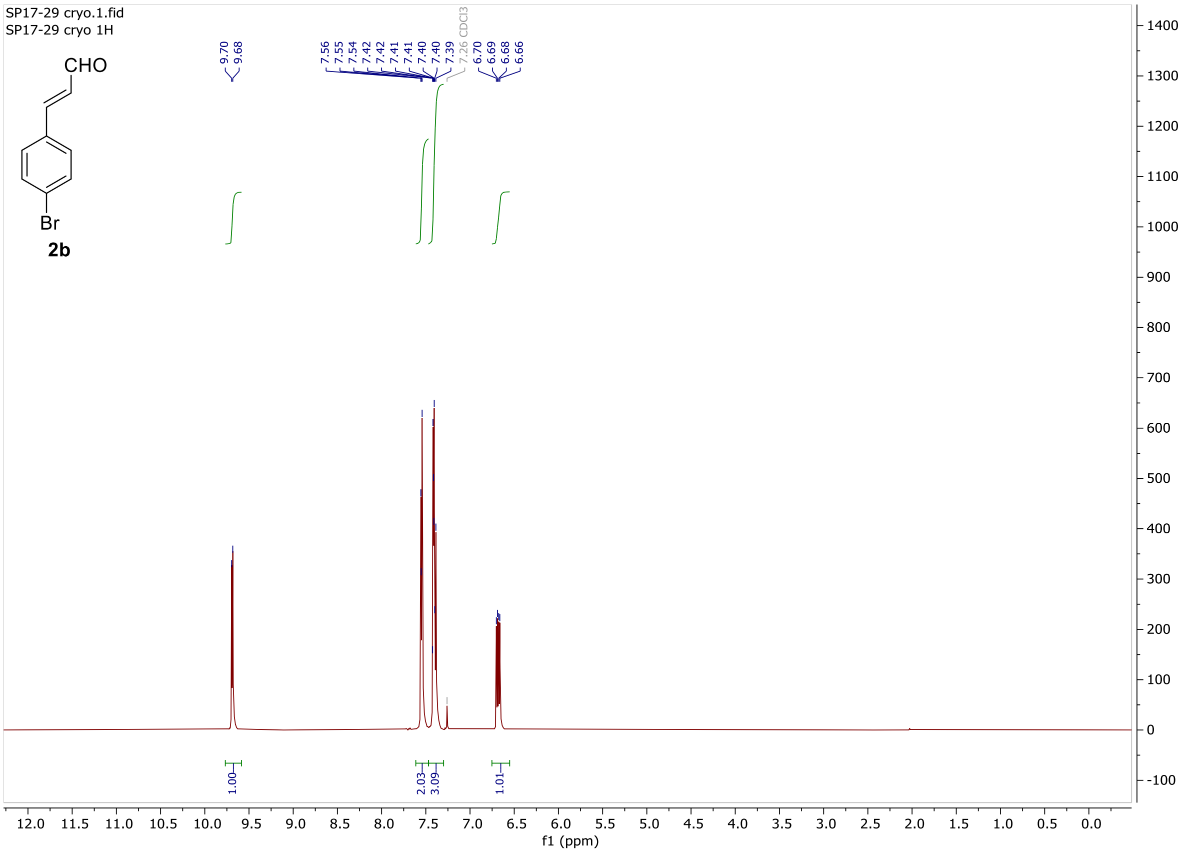

^{1}H NMR spectrum of **2b** ($CDCl_3$, 600 MHz).

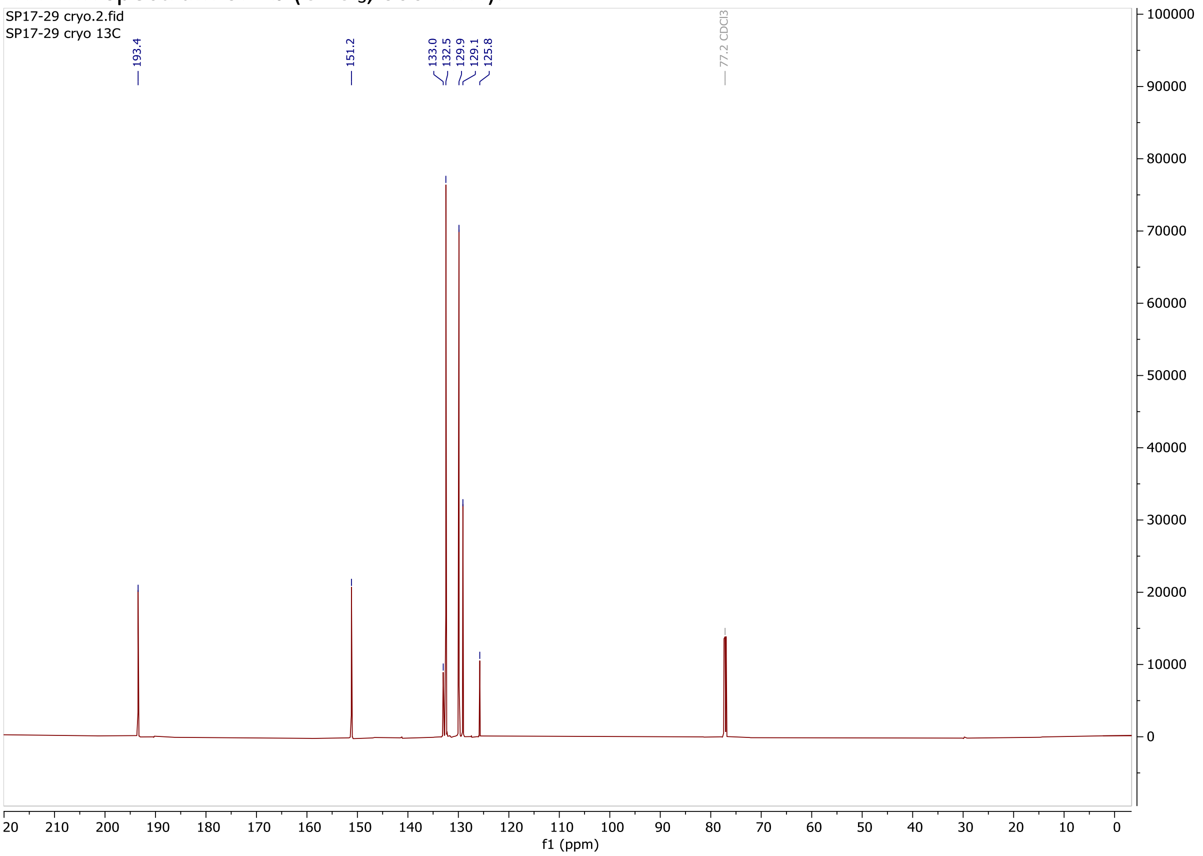

^{13}C {1H} NMR spectrum of **2b** ($CDCl_3$, 151 MHz).

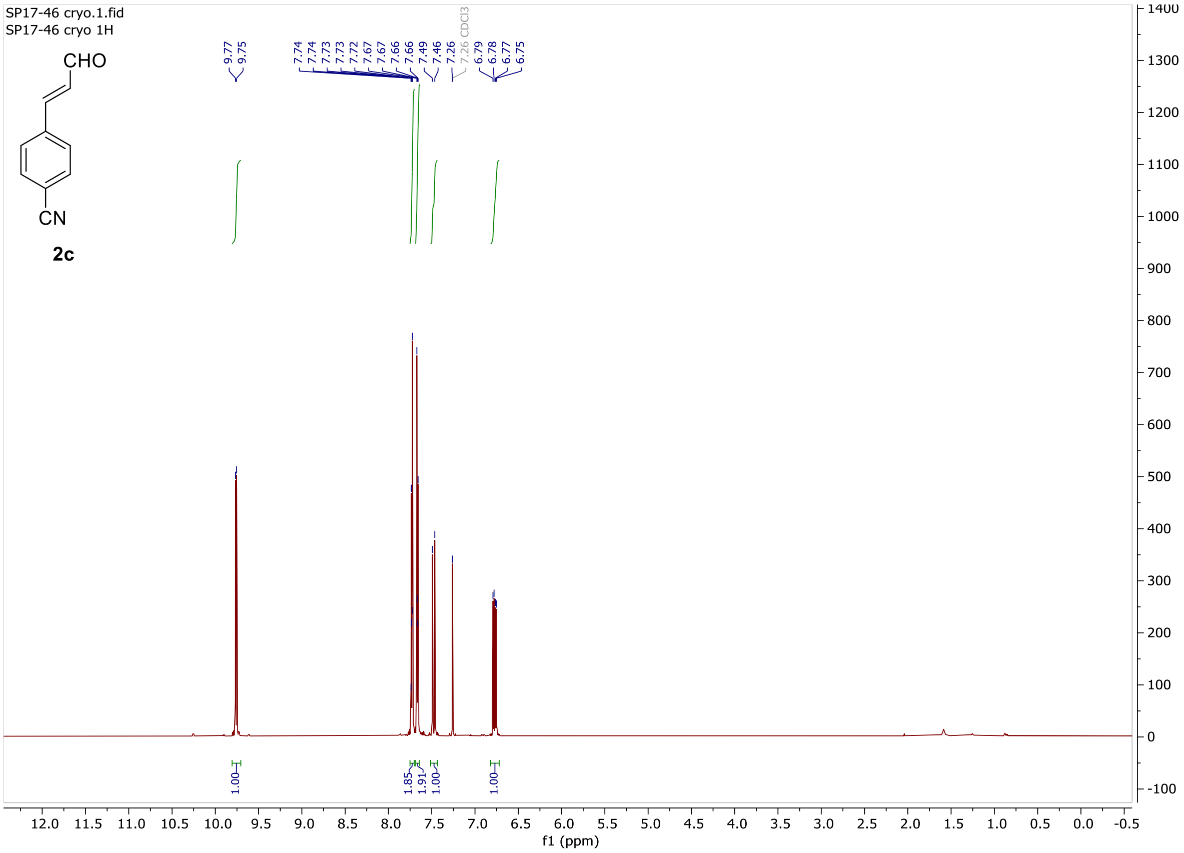

^{1}H NMR spectrum of **2c** ($CDCl_3$, 600 MHz).

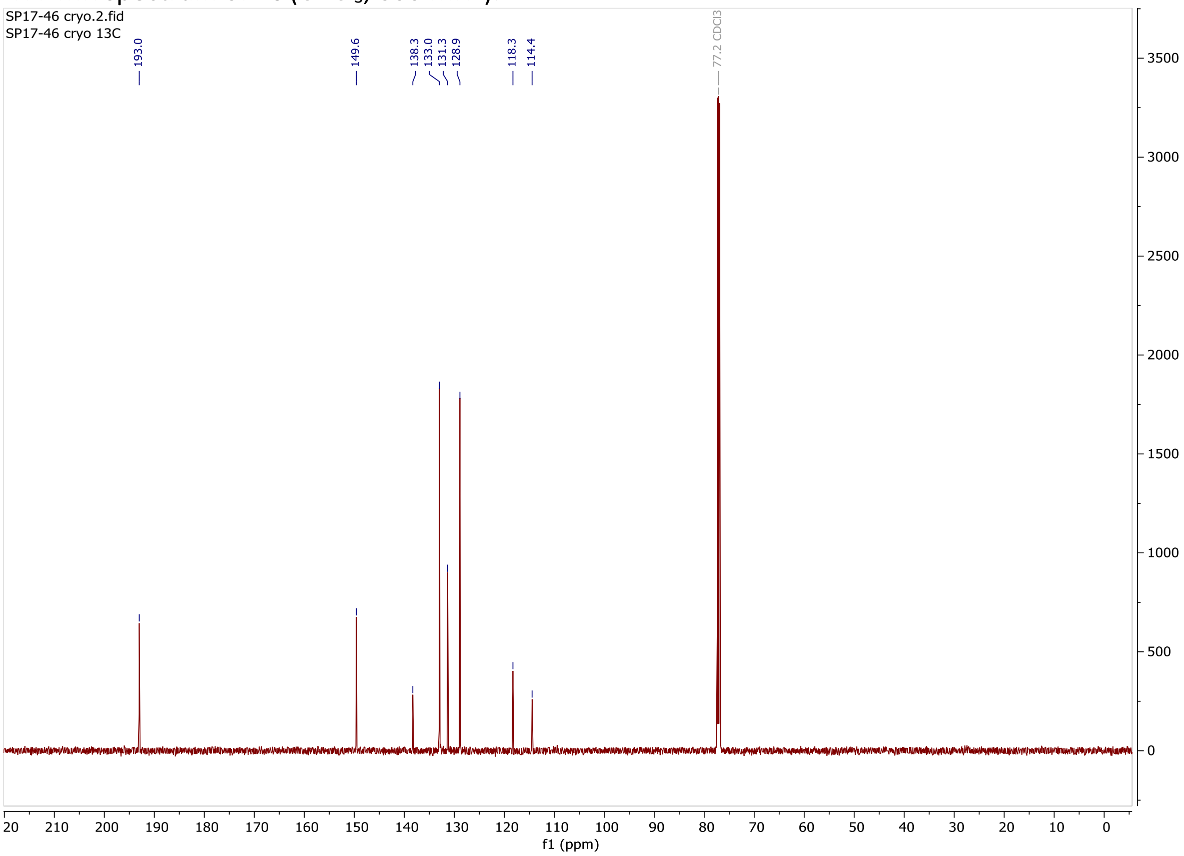

^{13}C {1H} NMR spectrum of **2c** ($CDCl_3$, 151 MHz).

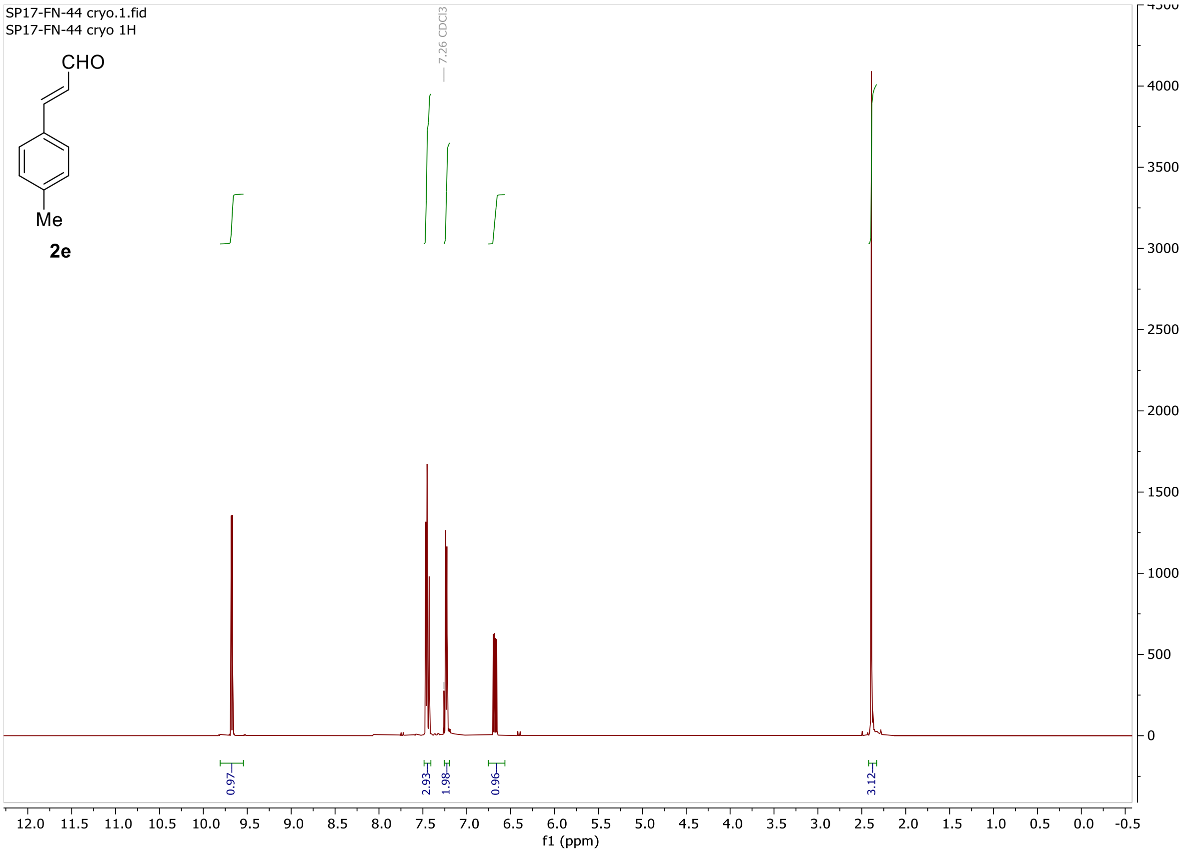

^{1}H NMR spectrum of **2e** ($CDCl_3$, 600 MHz).

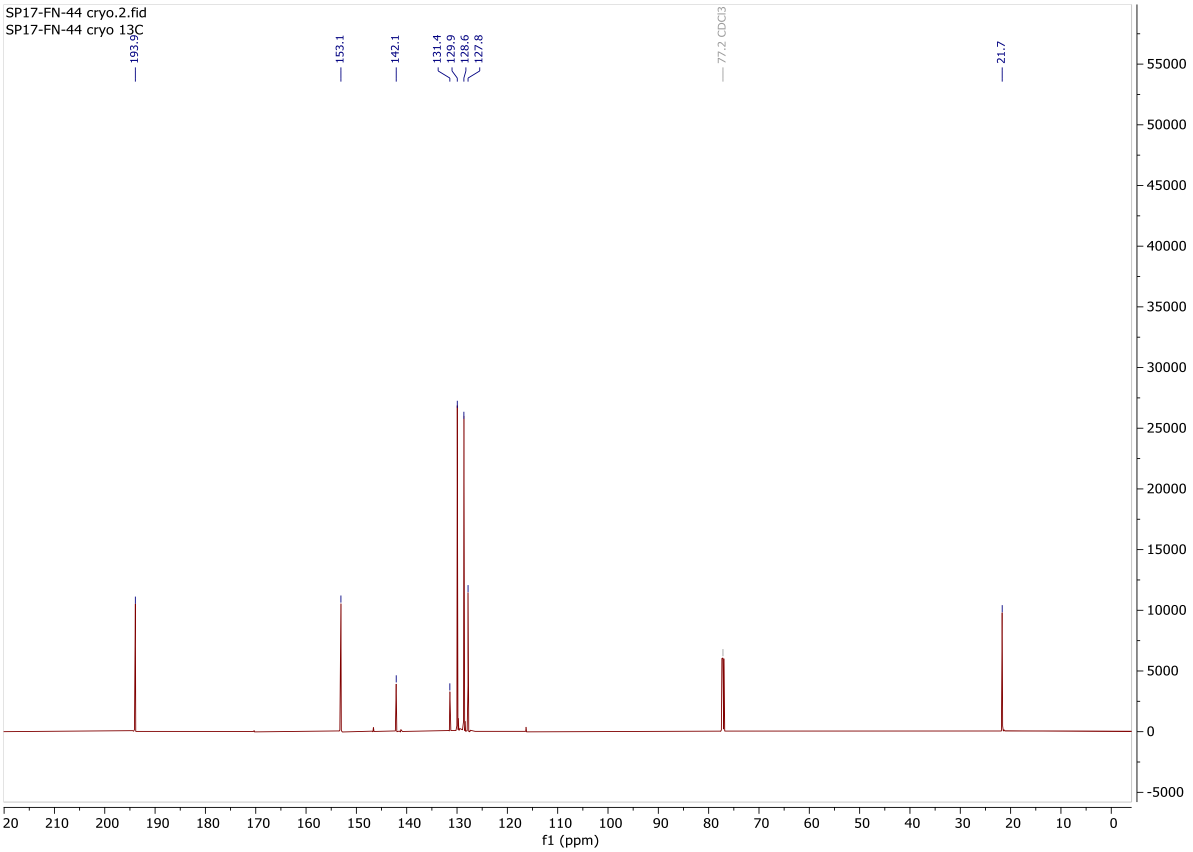

^{13}C {1H} NMR spectrum of **2e** ($CDCl_3$, 151 MHz).

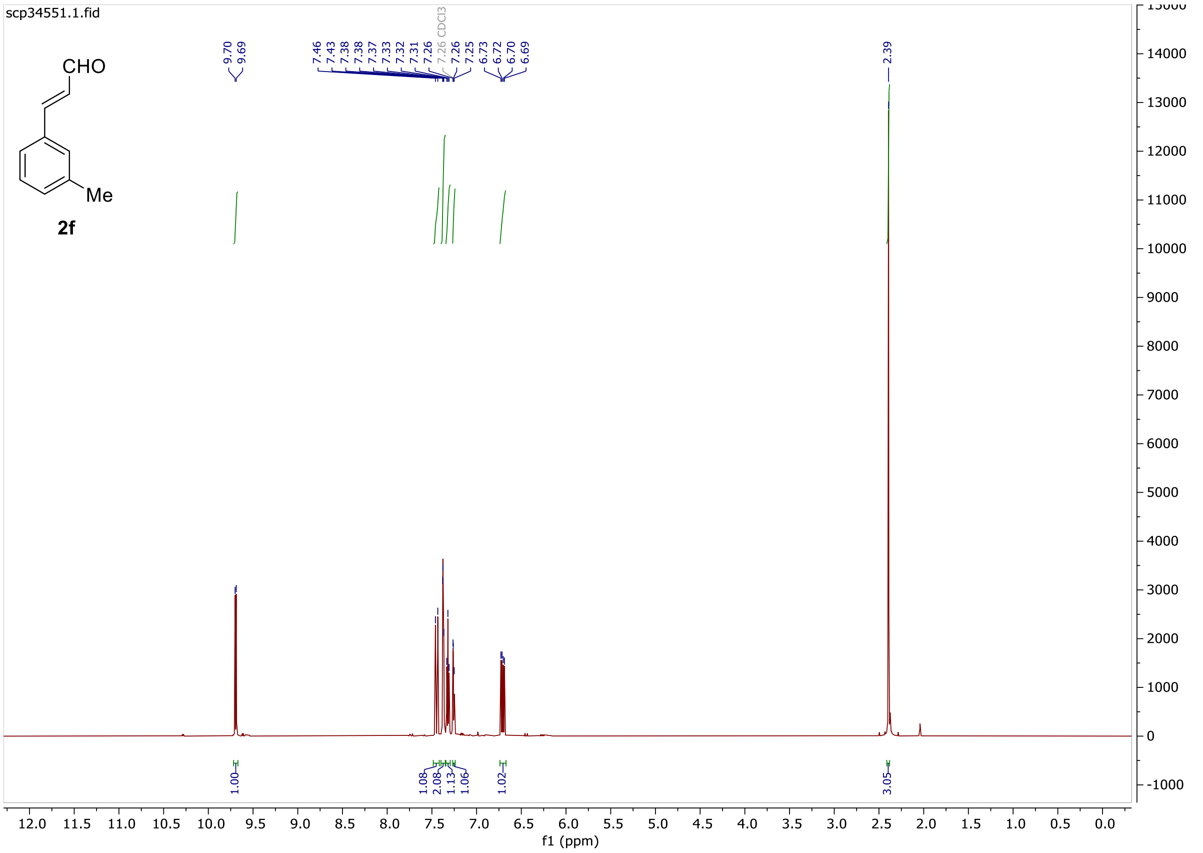

^{1}H NMR spectrum of **2f** ($CDCl_3$, 600 MHz).

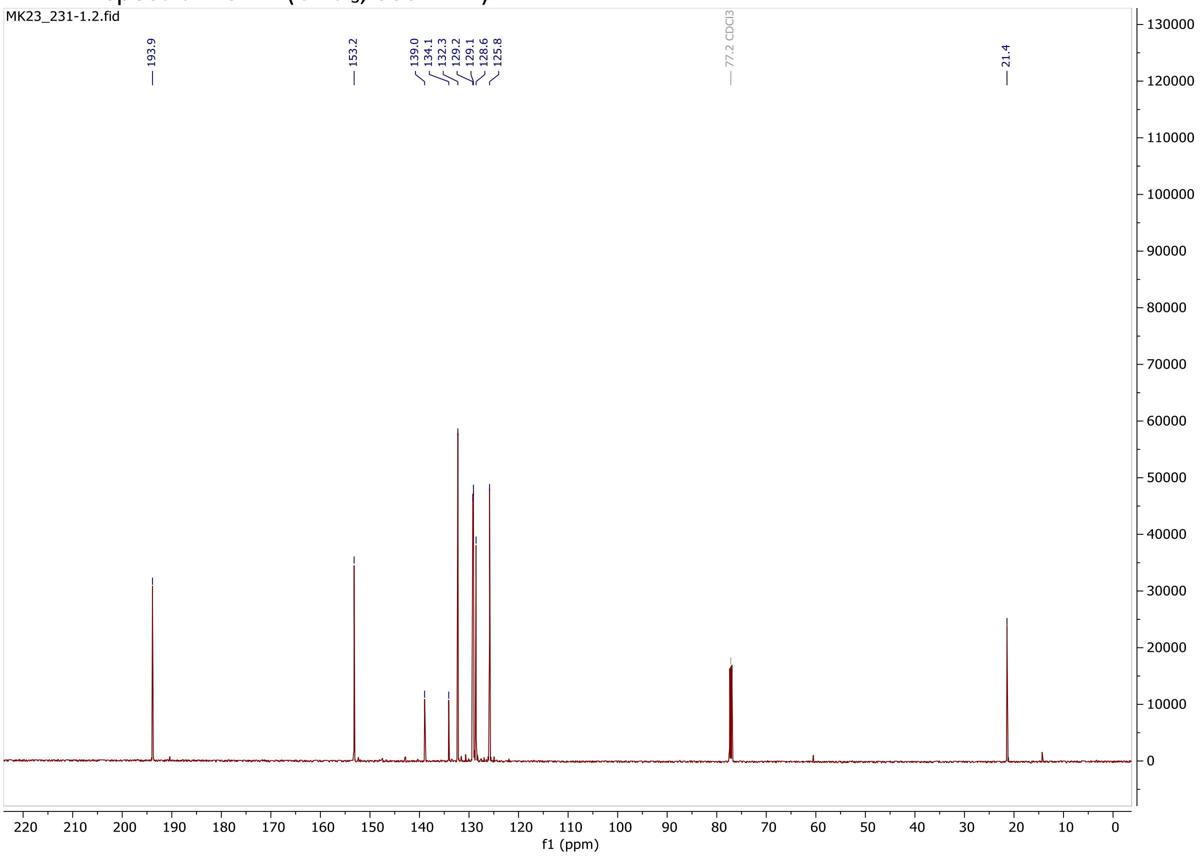

^{13}C {1H} NMR spectrum of **2f** ($CDCl_3$, 151 MHz).

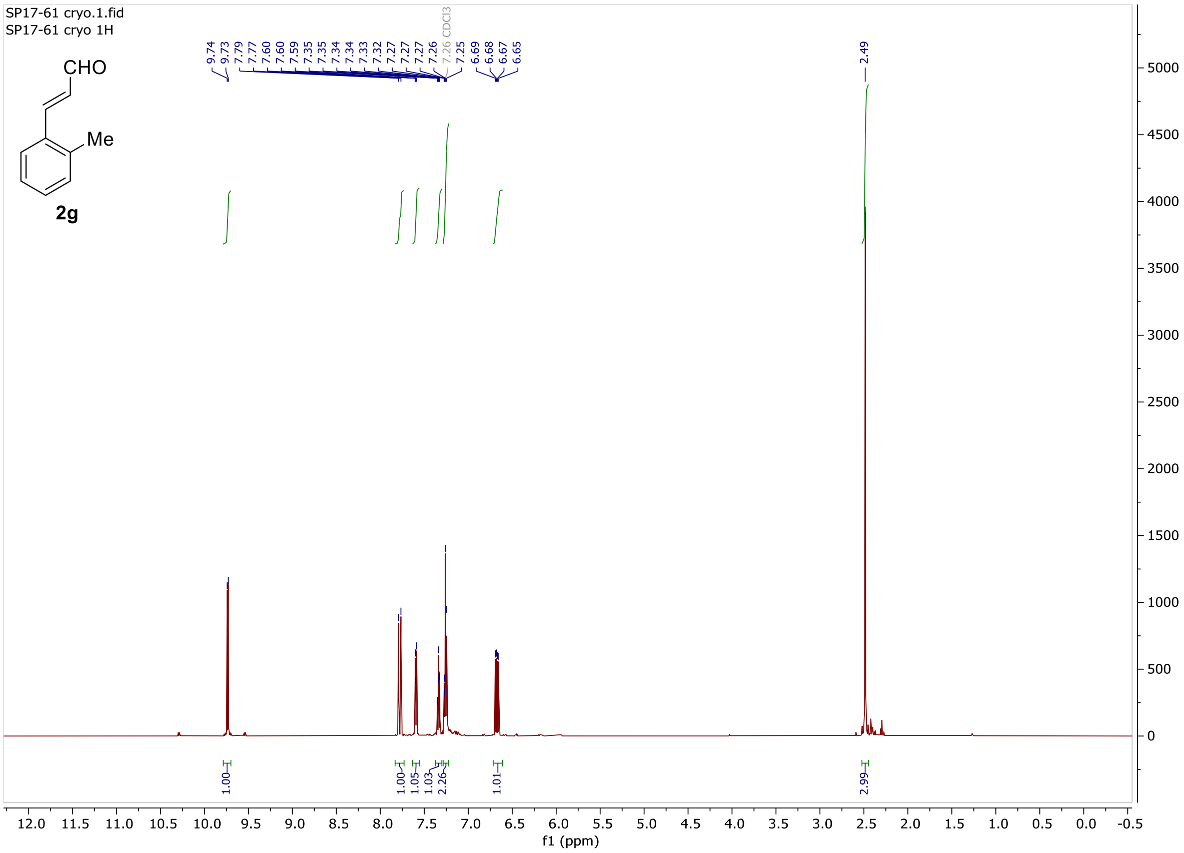

^{1}H NMR spectrum of **2g** ($CDCl_3$, 600 MHz).

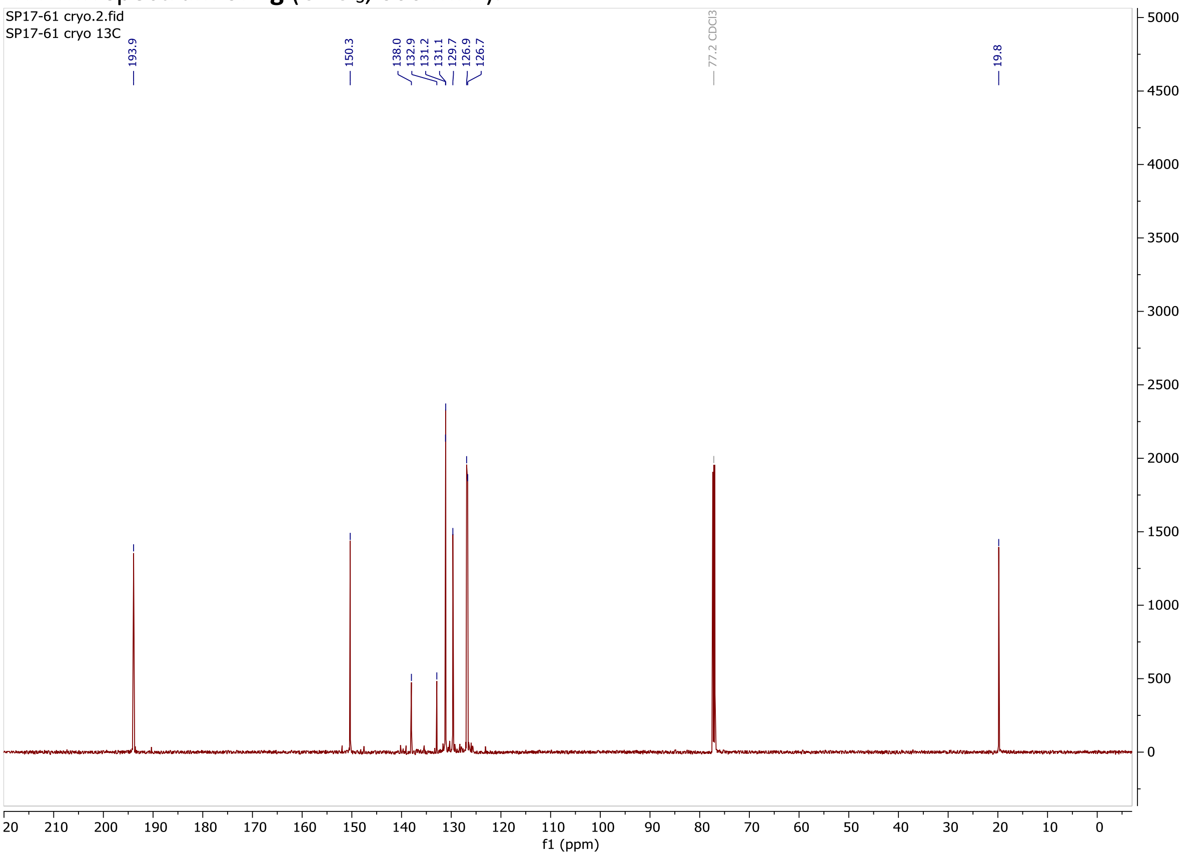

^{13}C {1H} NMR spectrum of **2g** ($CDCl_3$, 151 MHz).

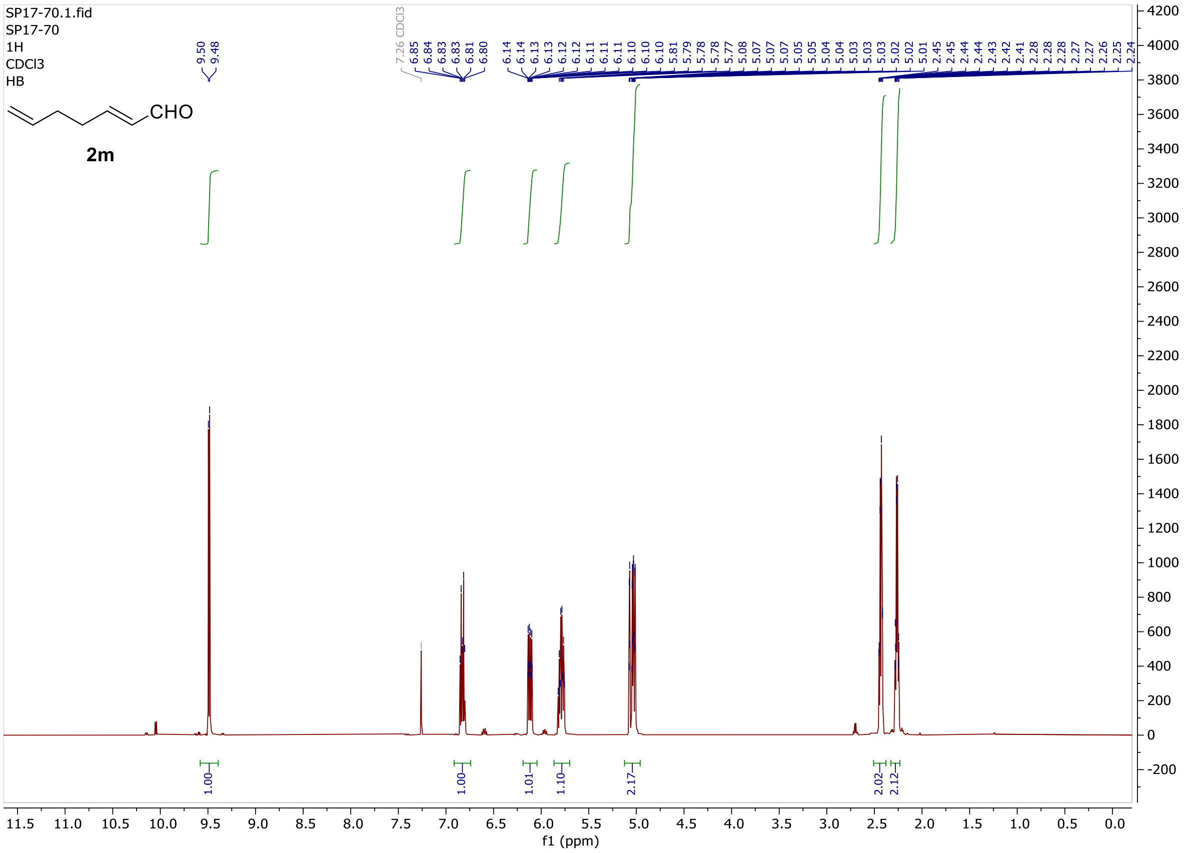

^{1}H NMR spectrum of **2m** ($CDCl_3$, 600 MHz).

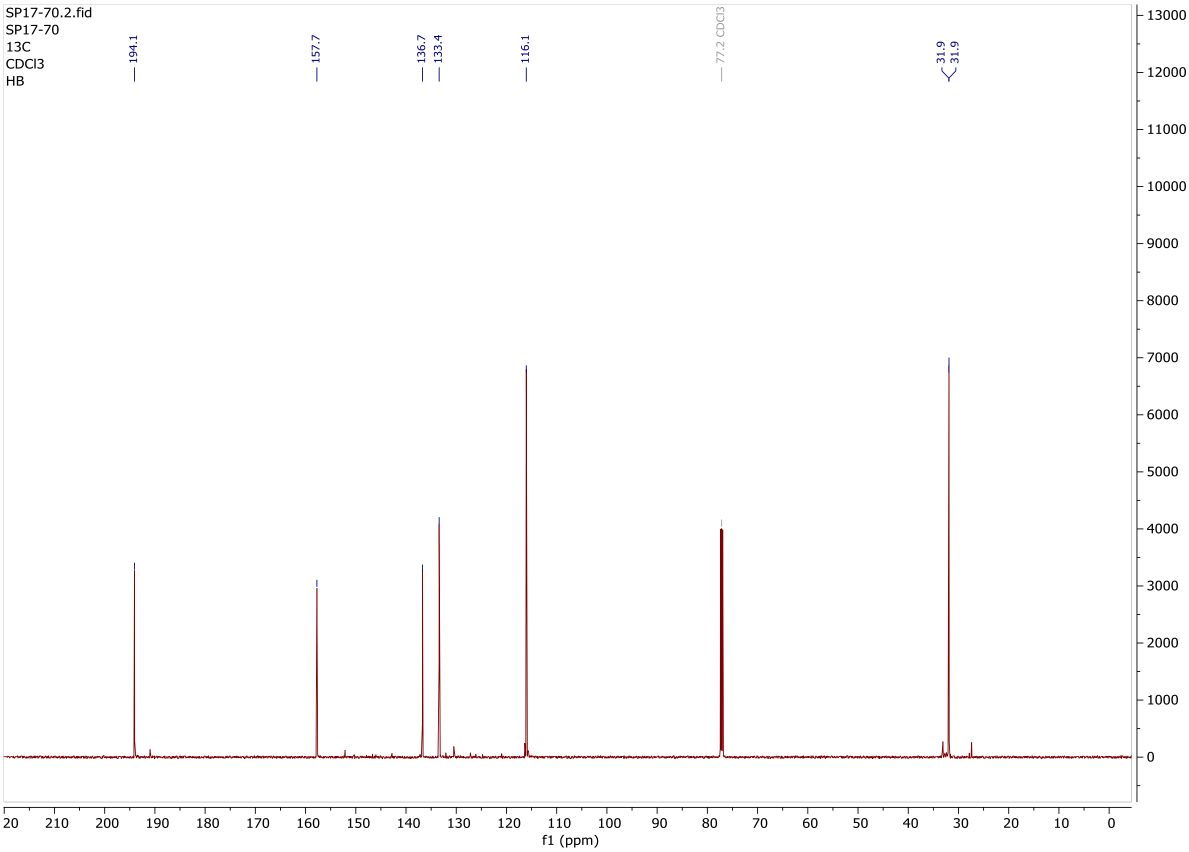

^{13}C {1H} NMR spectrum of **2m** ($CDCl_3$, 151 MHz).

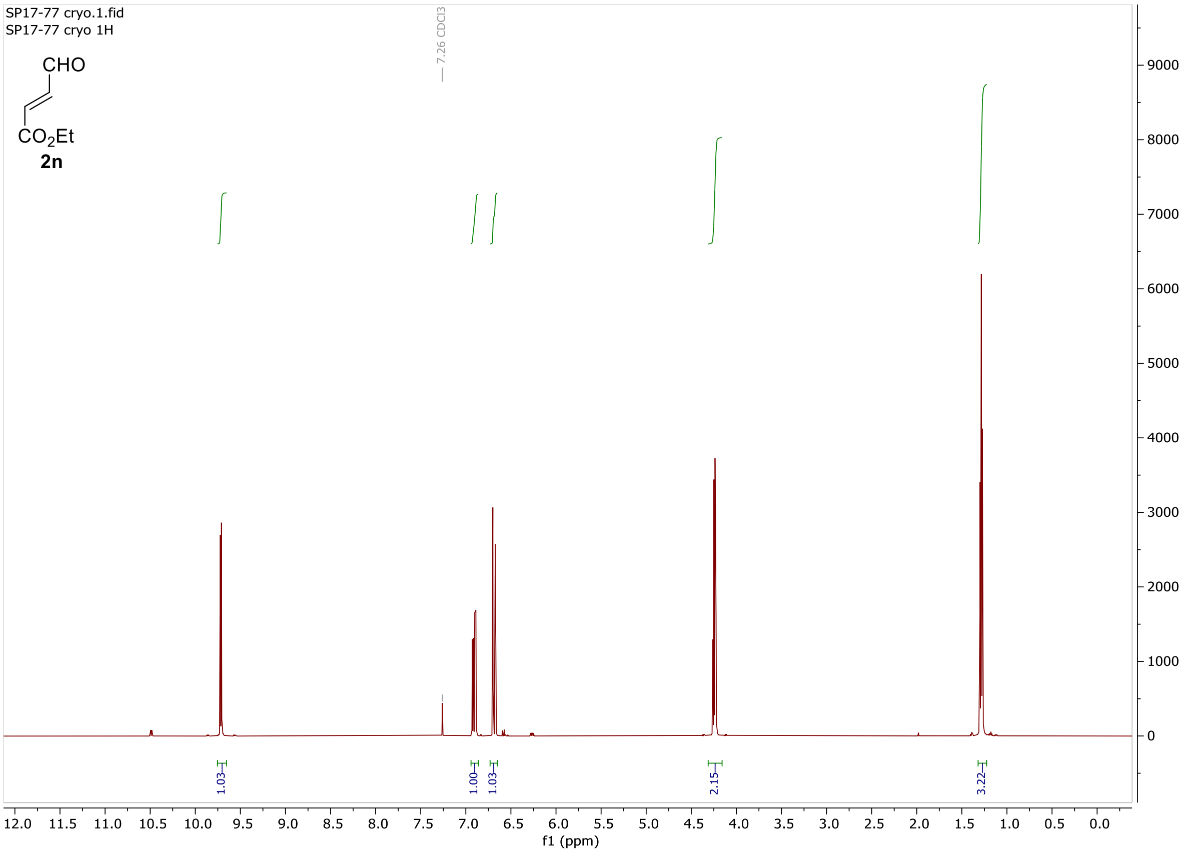

^{1}H NMR spectrum of **2n** ($CDCl_3$, 600 MHz).

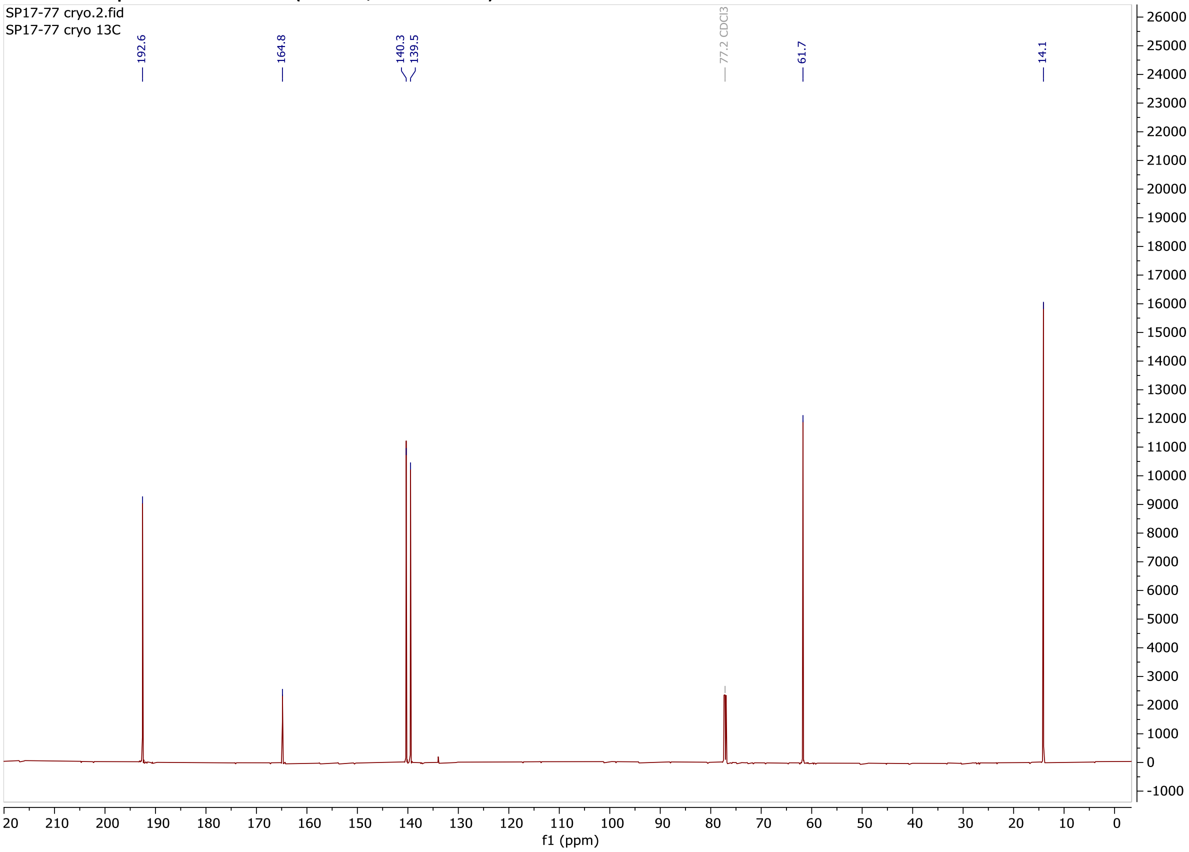

^{13}C {1H} NMR spectrum of **2n** ($CDCl_3$, 151 MHz).

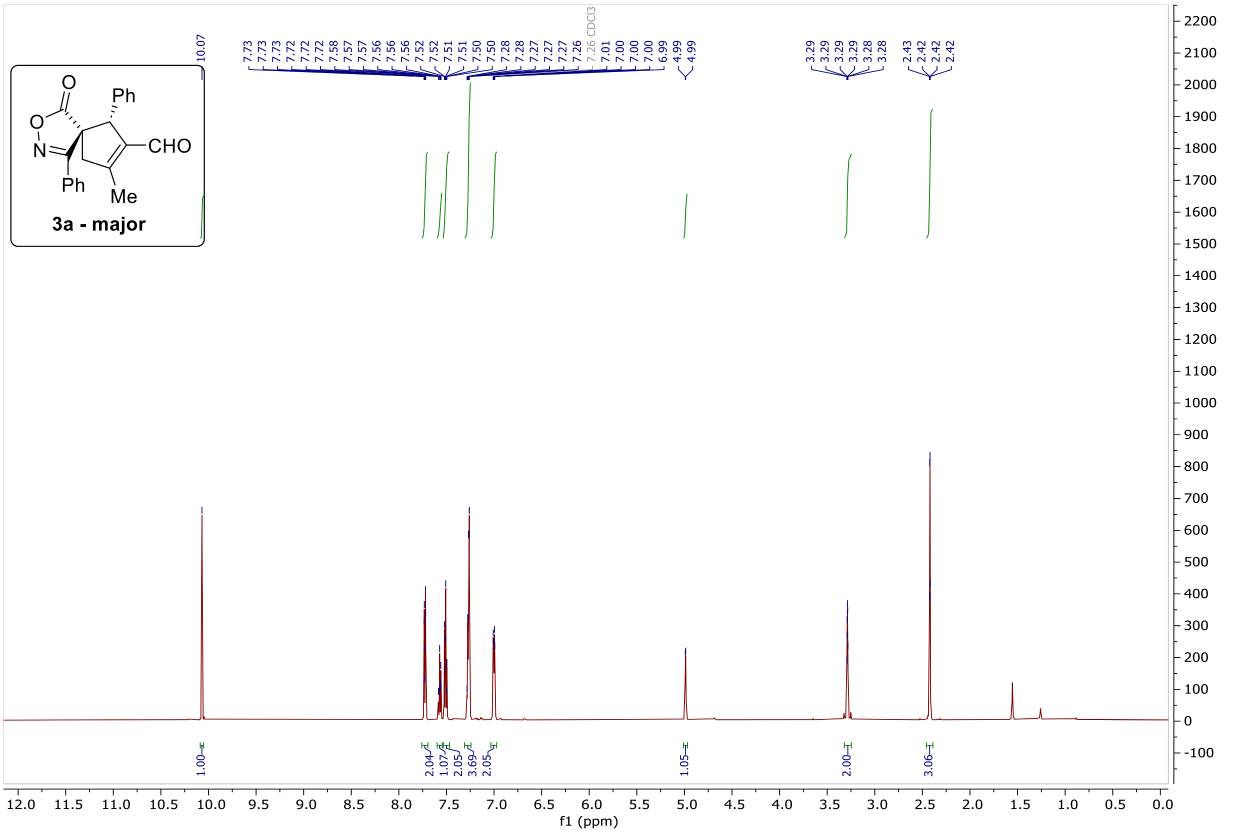

^{1}H NMR spectrum of **3a – major** ($CDCl_3$, 600 MHz).

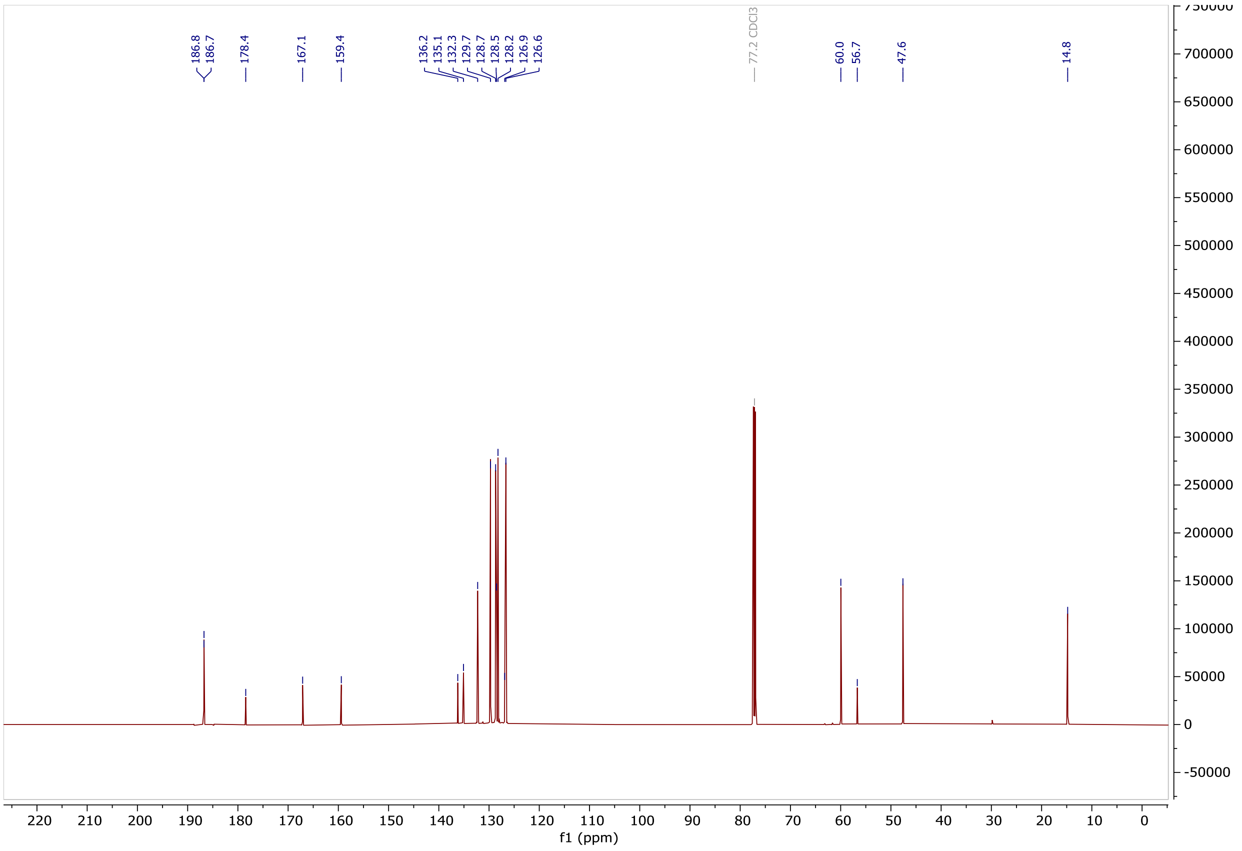

^{13}C {1H} NMR spectrum of **3a - major** ($CDCl_3$, 151 MHz).

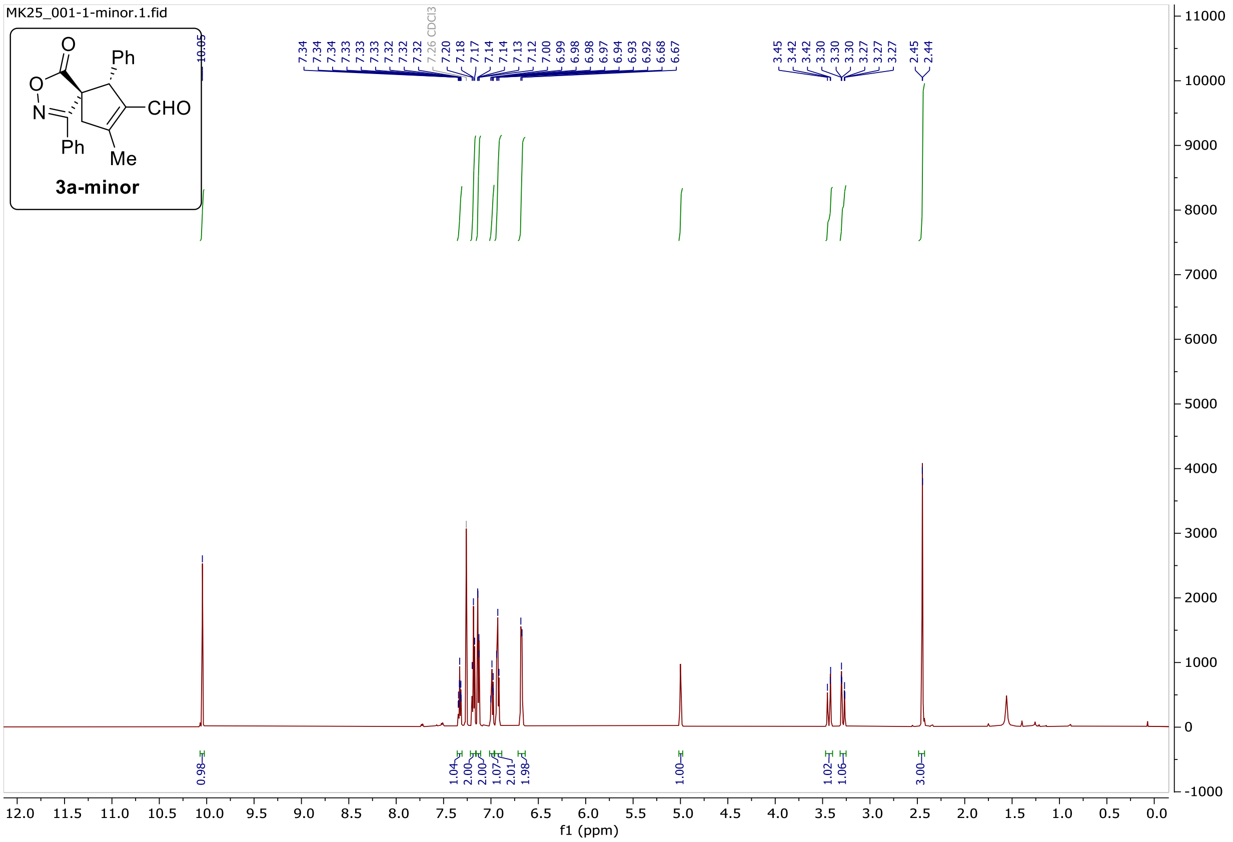

^{1}H NMR spectrum of **3a – minor** ($CDCl_3$, 600 MHz).

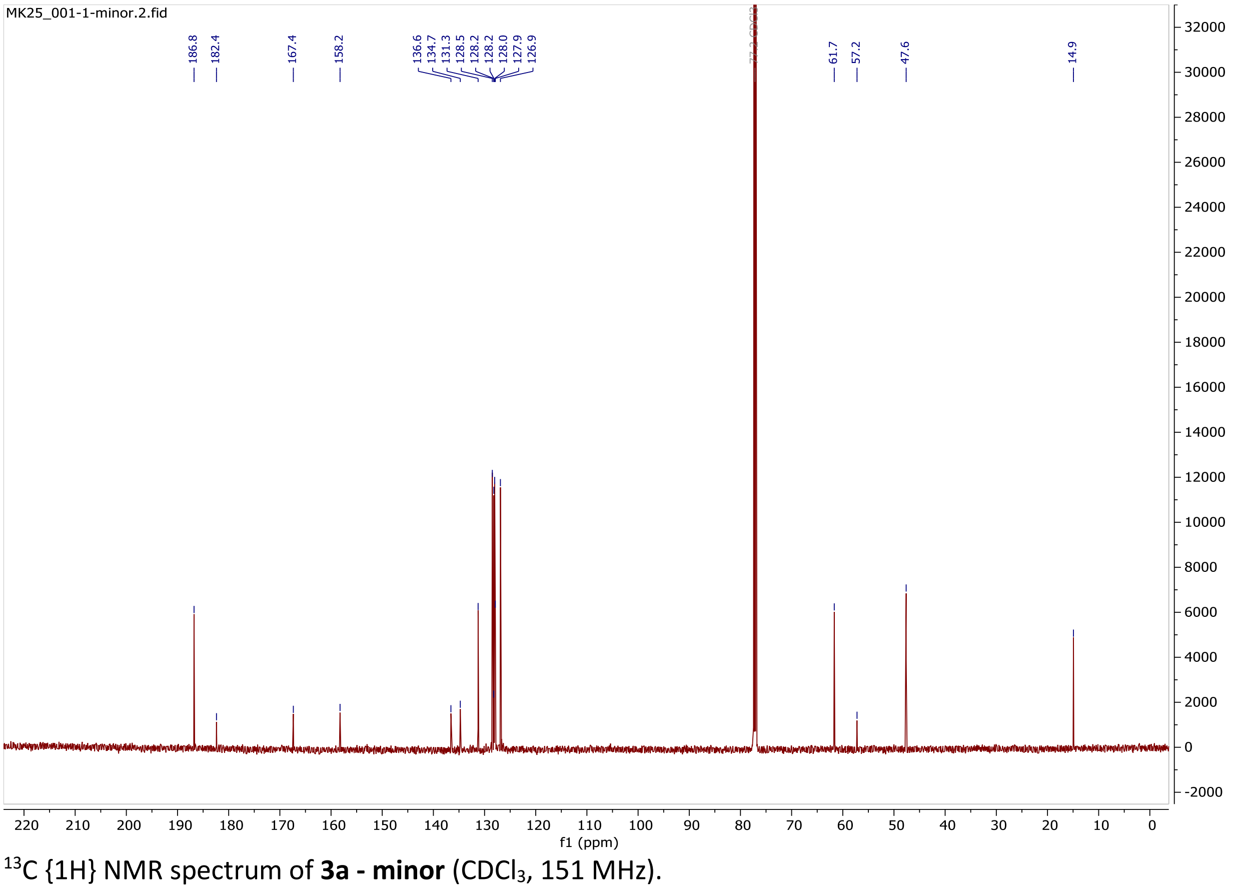

^{13}C {1H} NMR spectrum of **3a - minor** ($CDCl_3$, 151 MHz).

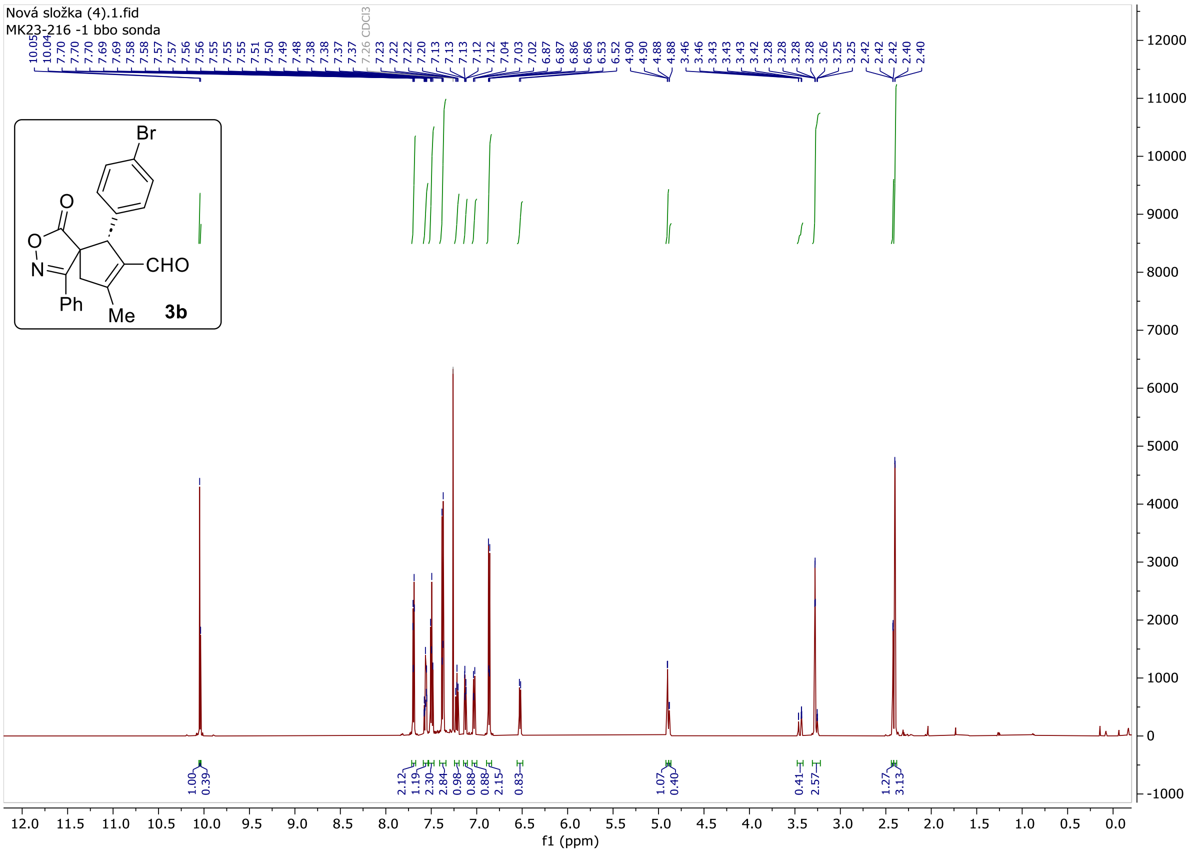

^{1}H NMR spectrum of **3b** ($CDCl_3$, 600 MHz).

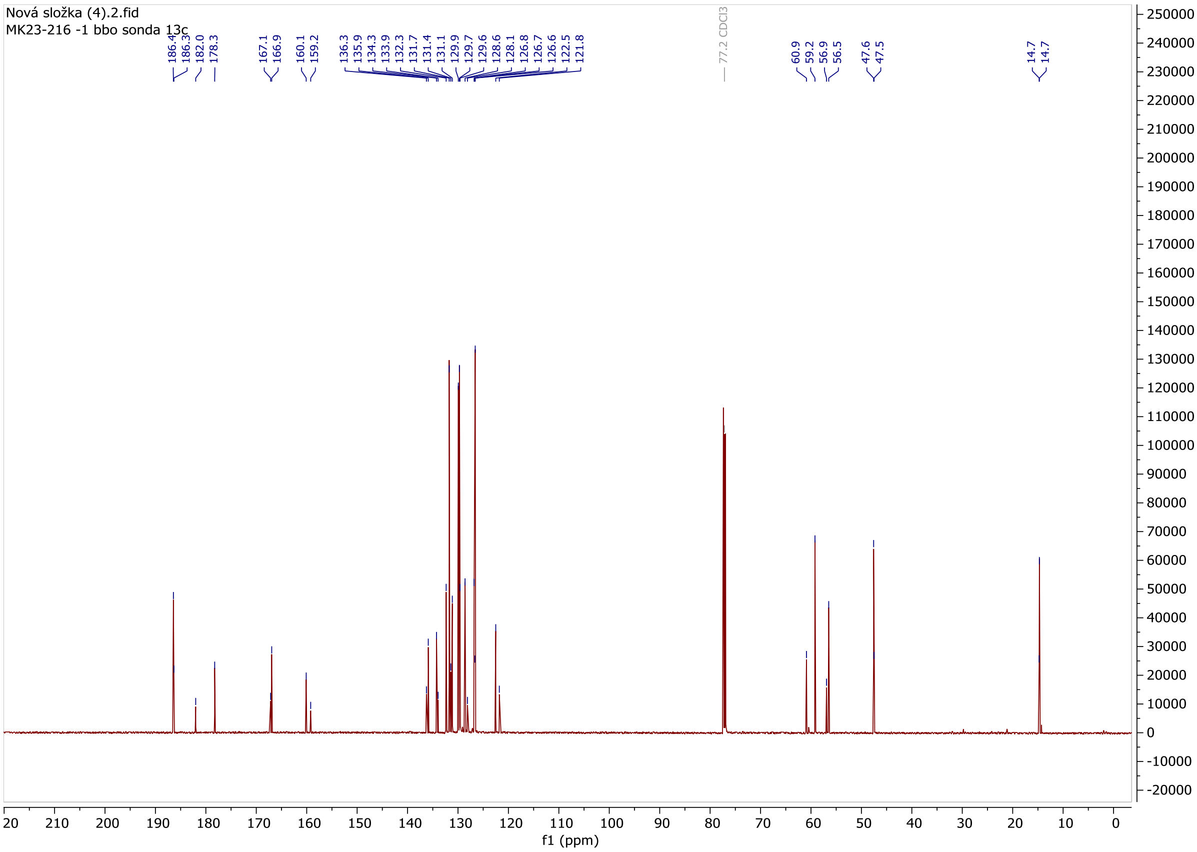

^{13}C {1H} NMR spectrum of **3b** ($CDCl_3$, 151 MHz).

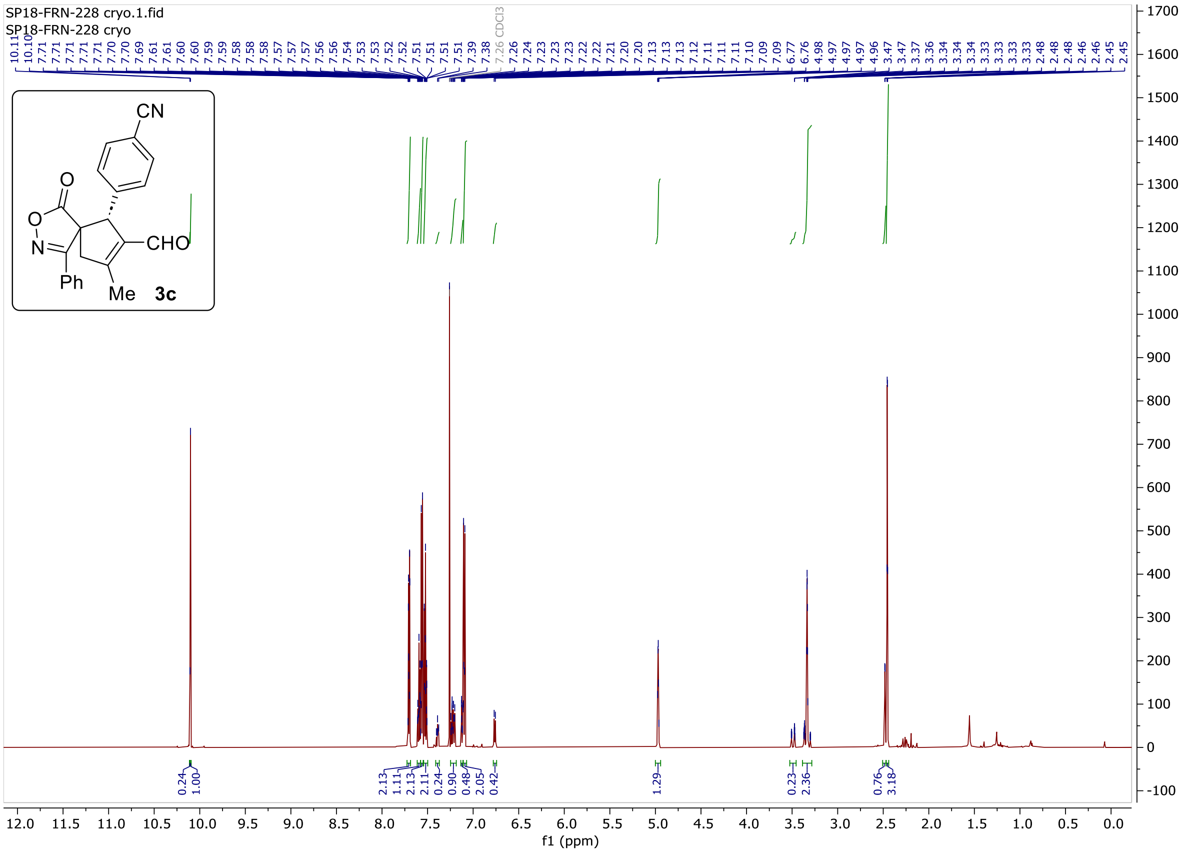

^{1}H NMR spectrum of **3c** ($CDCl_3$, 600 MHz).

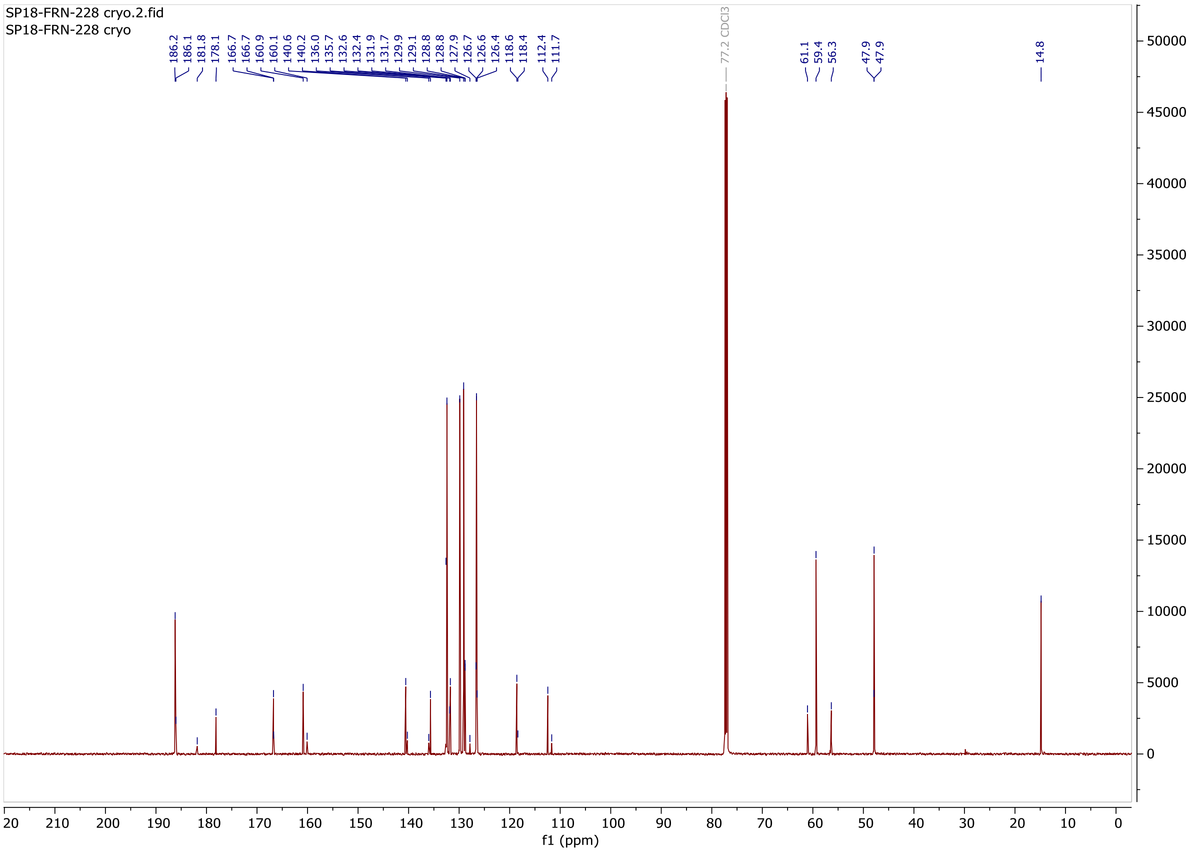

^{13}C {1H} NMR spectrum of **3c** ($CDCl_3$, 151 MHz).

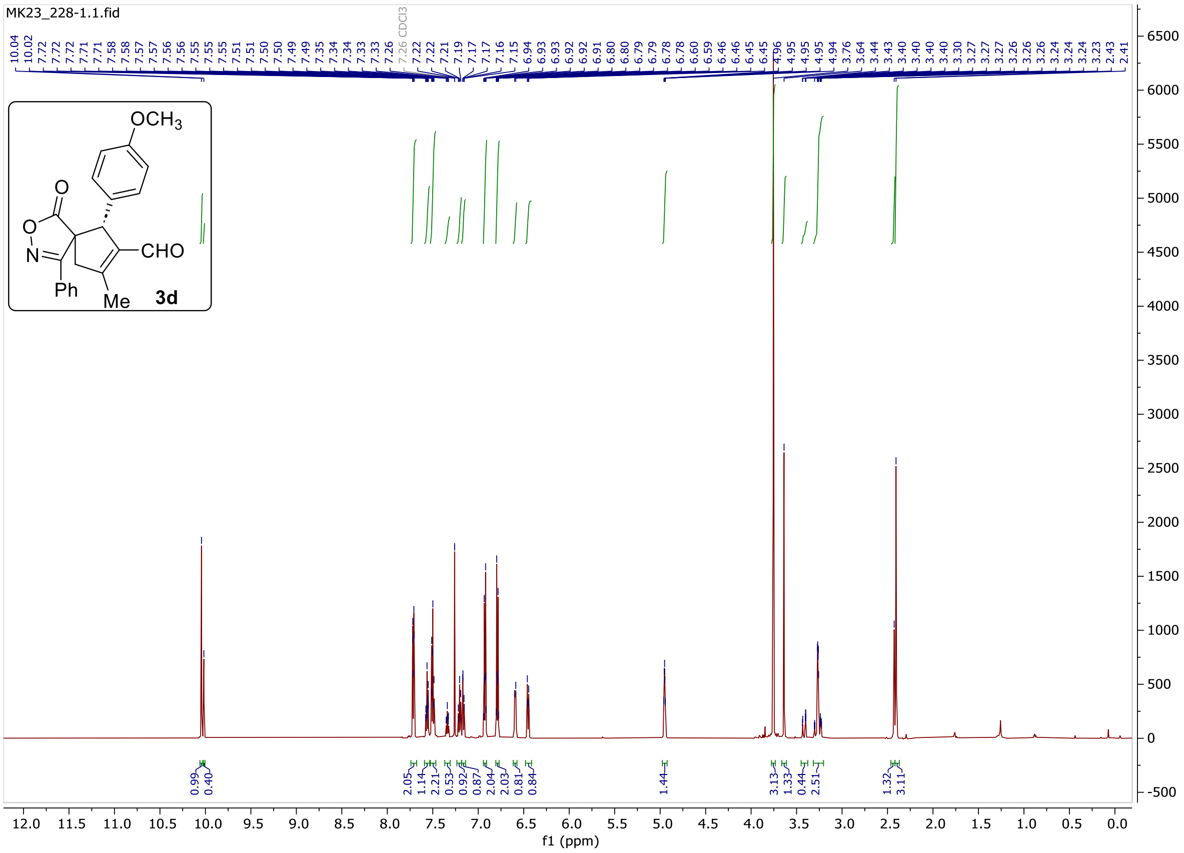

^{1}H NMR spectrum of **3d** ($CDCl_3$, 600 MHz).

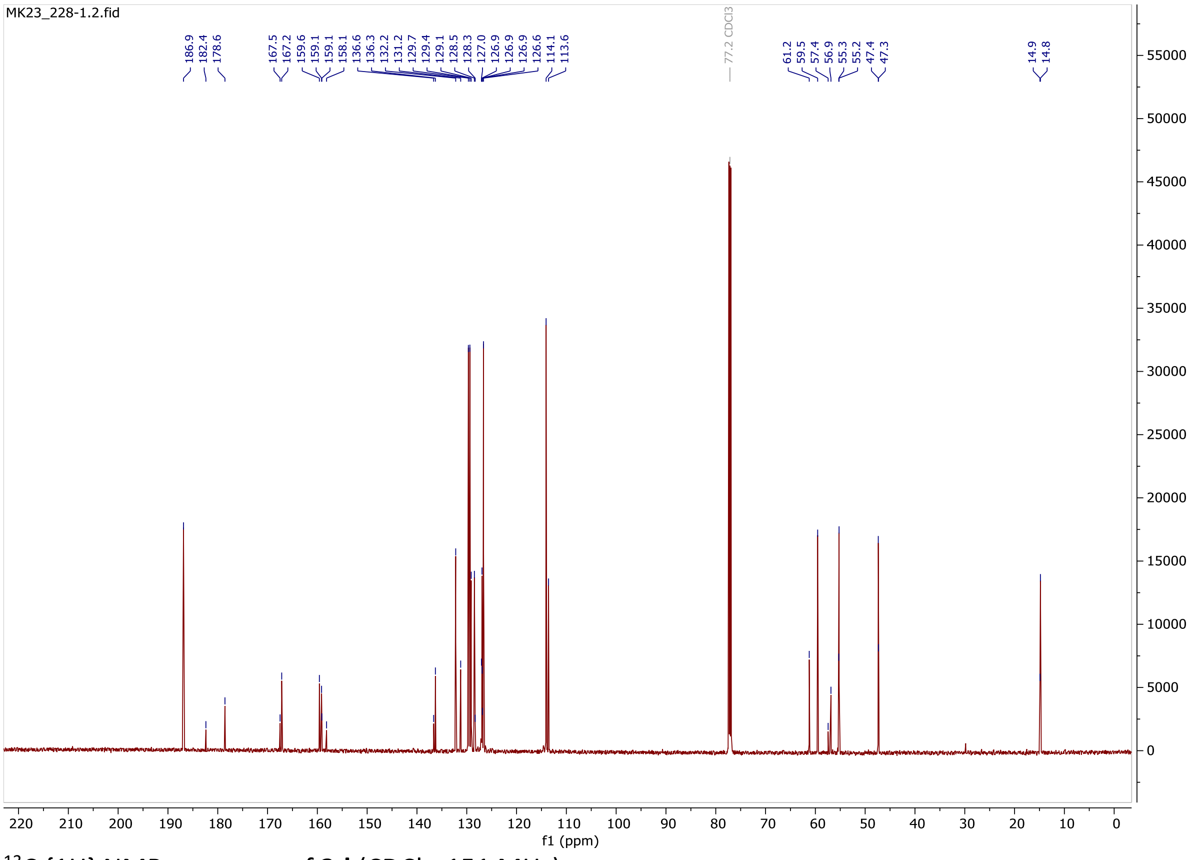

^{13}C {1H} NMR spectrum of **3d** ($CDCl_3$, 151 MHz).

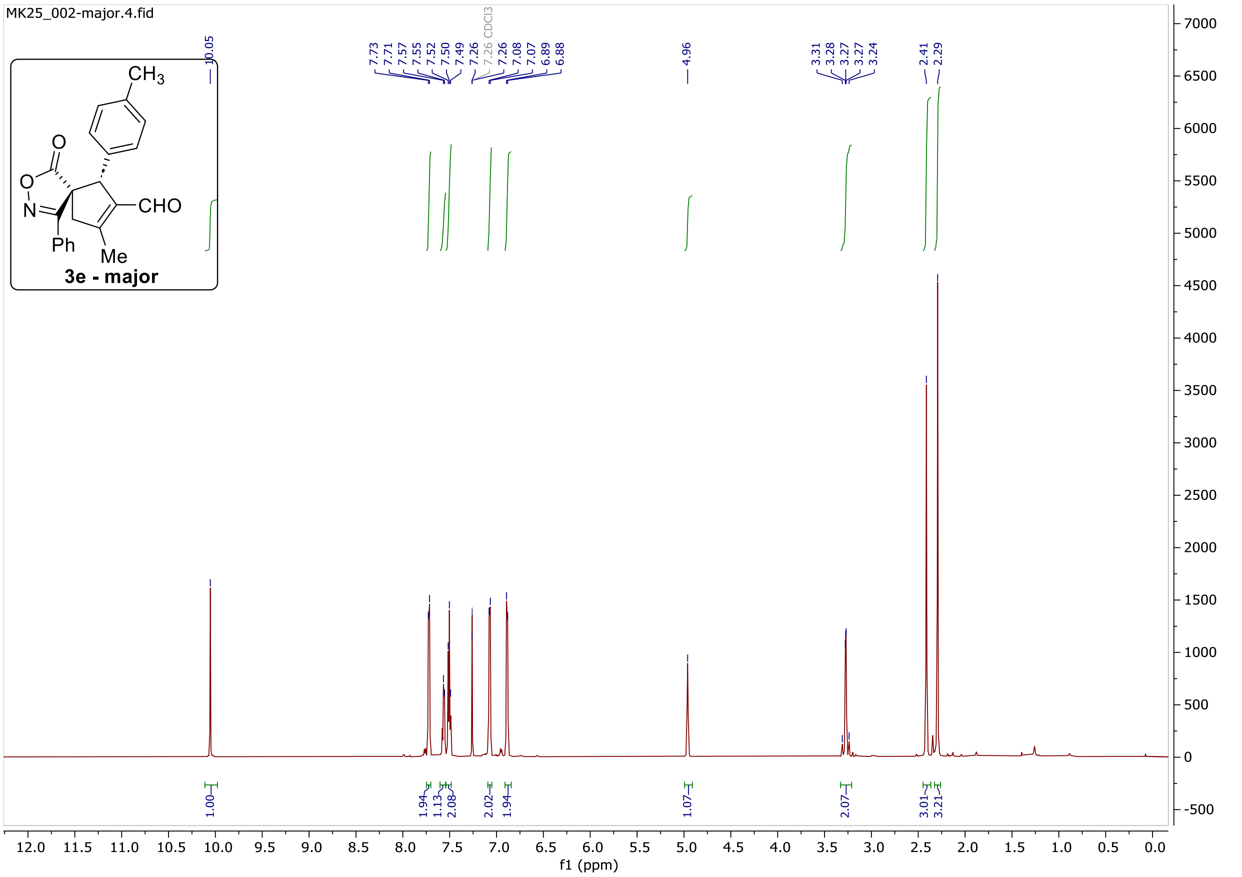

^{1}H NMR spectrum of **3e - major** ($CDCl_3$, 600 MHz).

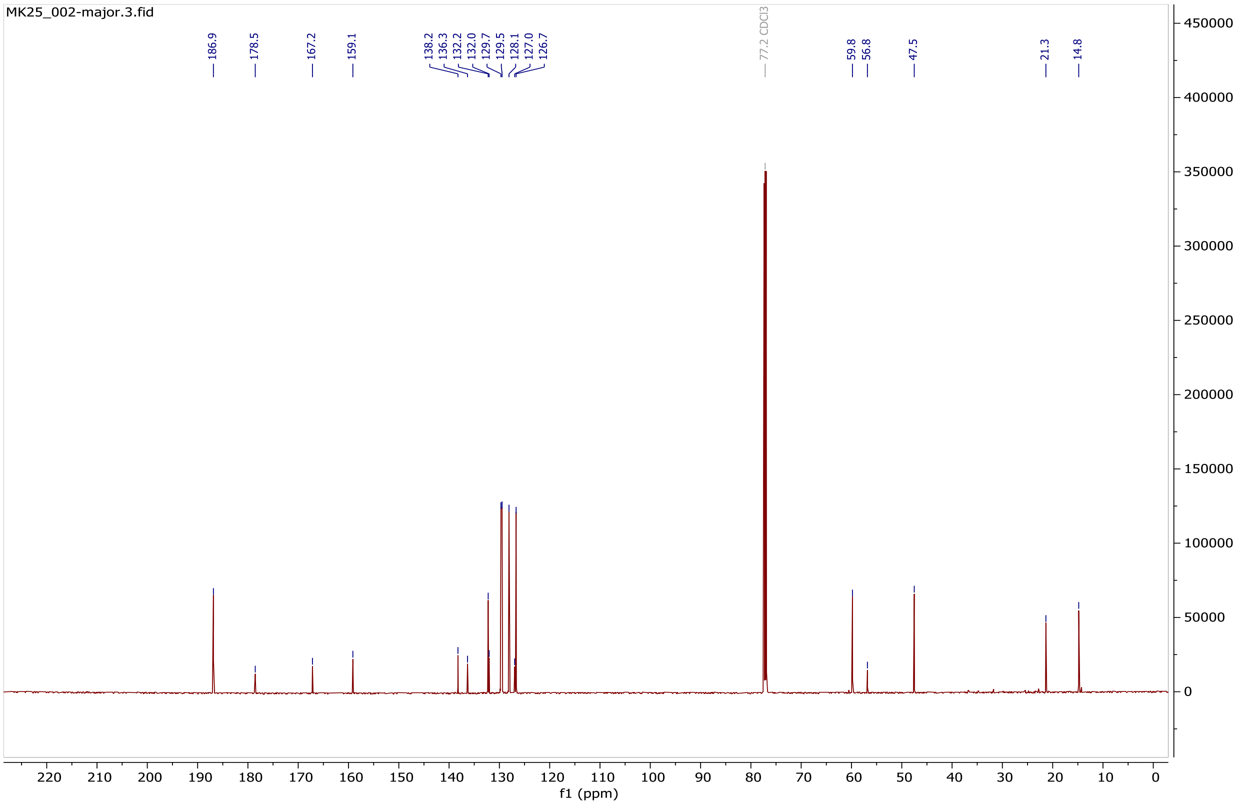

^{13}C {1H} NMR spectrum of **3e - major** ($CDCl_3$, 151 MHz).

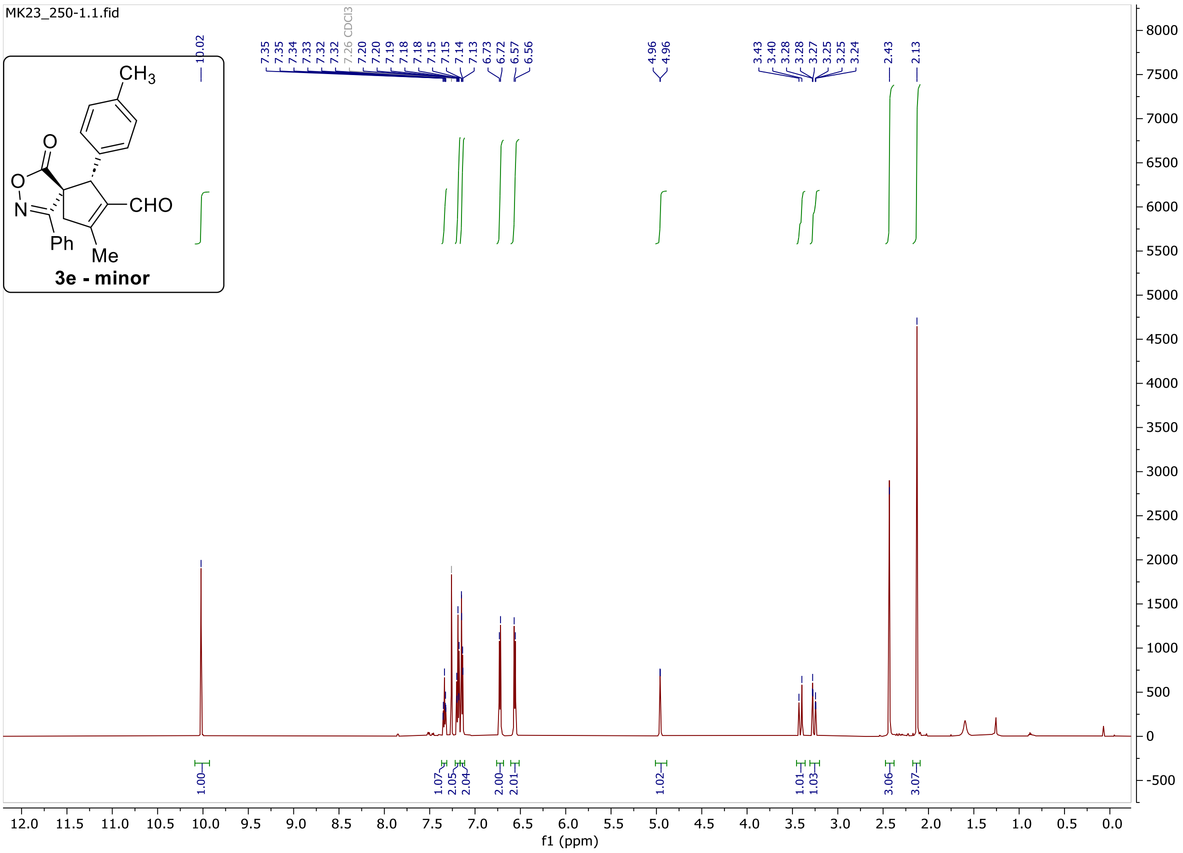

^{1}H NMR spectrum of **3e - minor** ($CDCl_3$, 600 MHz).

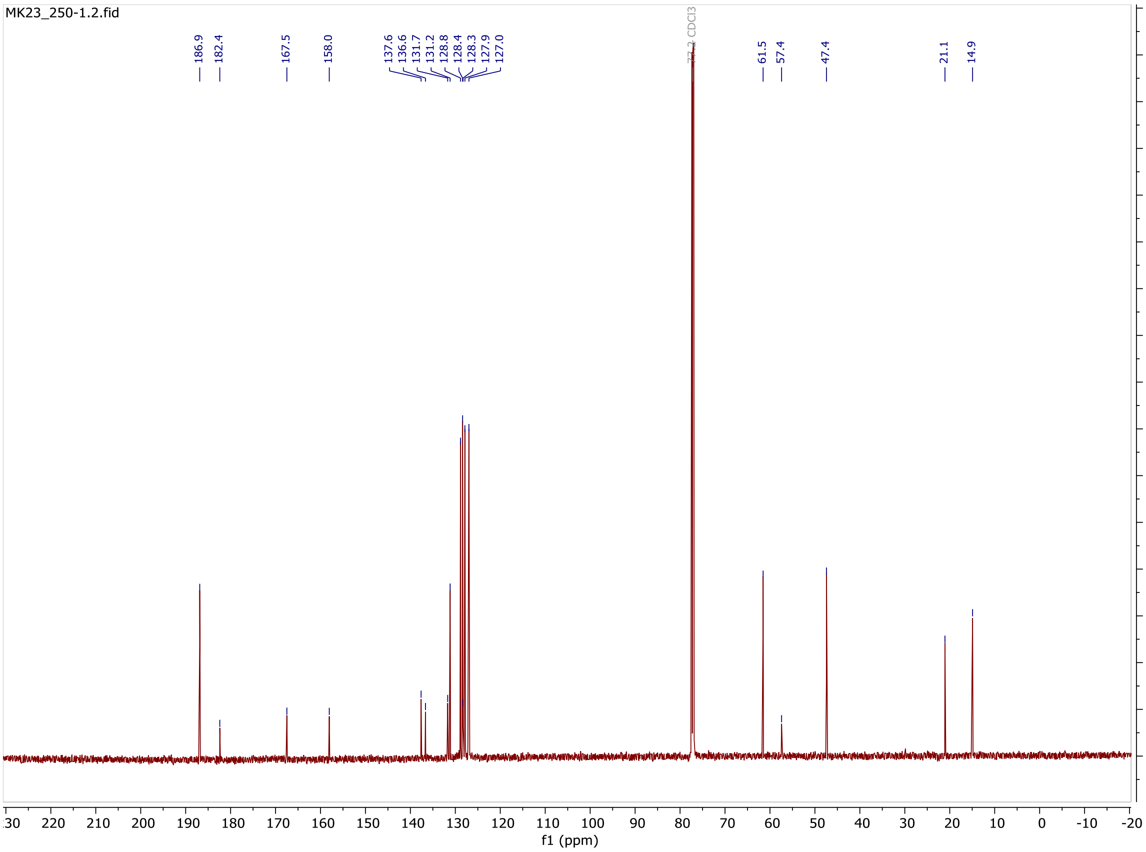

^{13}C {1H} NMR spectrum of **3e - minor** ($CDCl_3$, 151 MHz).

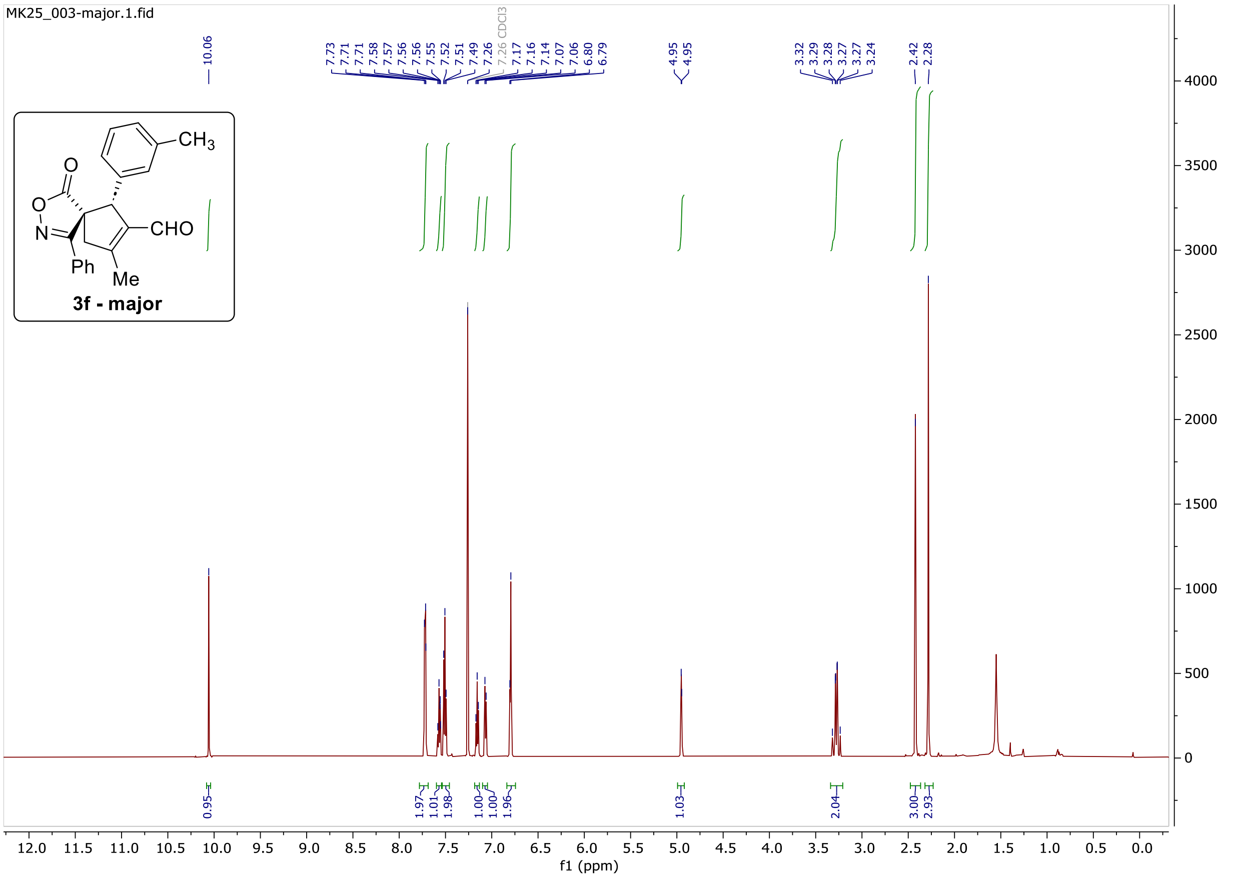

^{1}H NMR spectrum of **3f - major** ($CDCl_3$, 600 MHz).

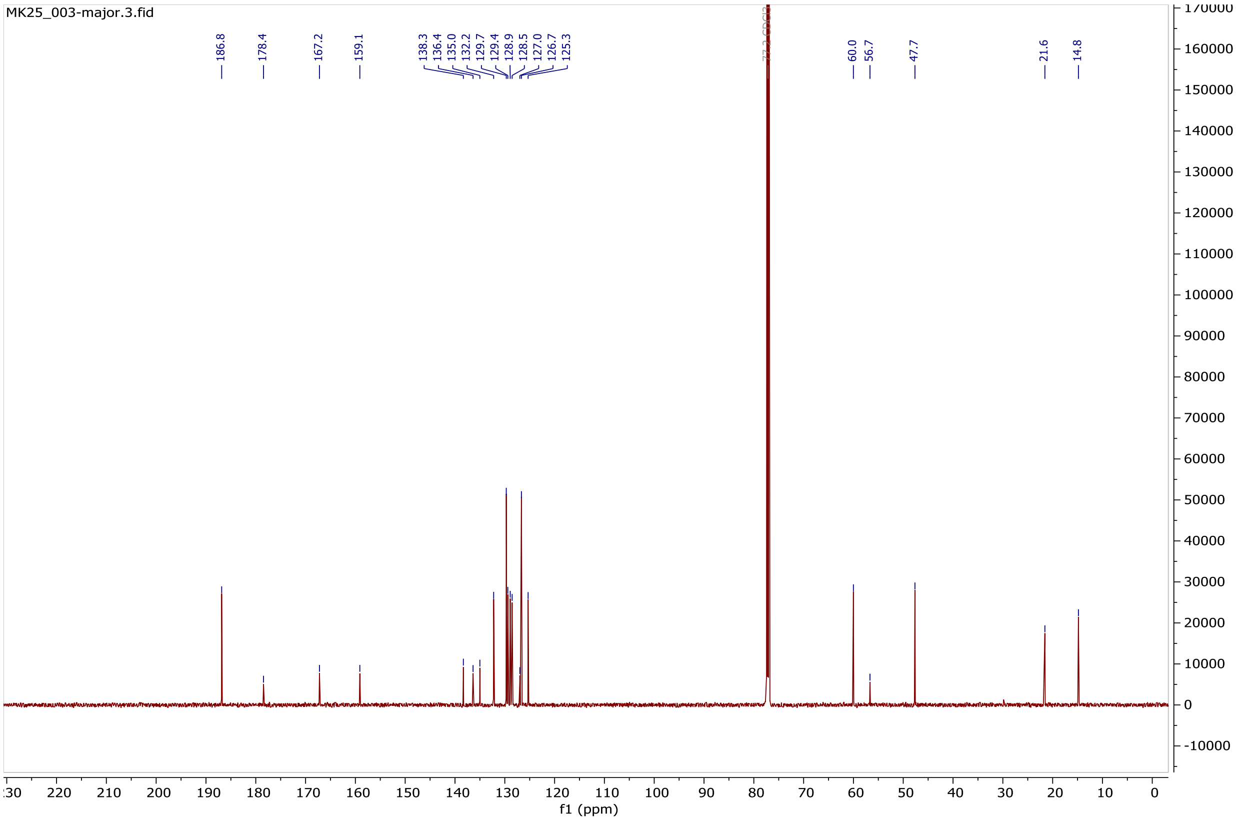

^{13}C {1H} NMR spectrum of **3f - major** ($CDCl_3$, 151 MHz).

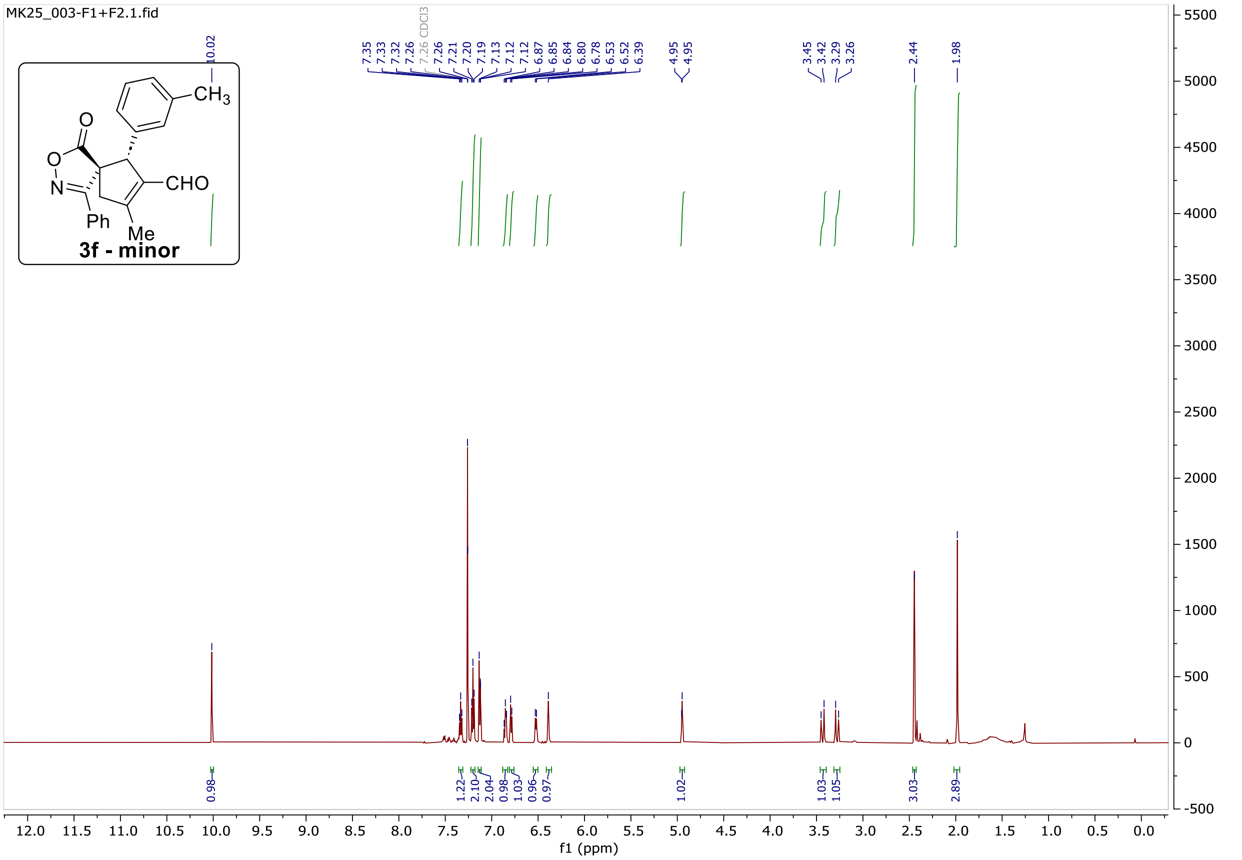

^{1}H NMR spectrum of **3f - minor** ($CDCl_3$, 600 MHz).

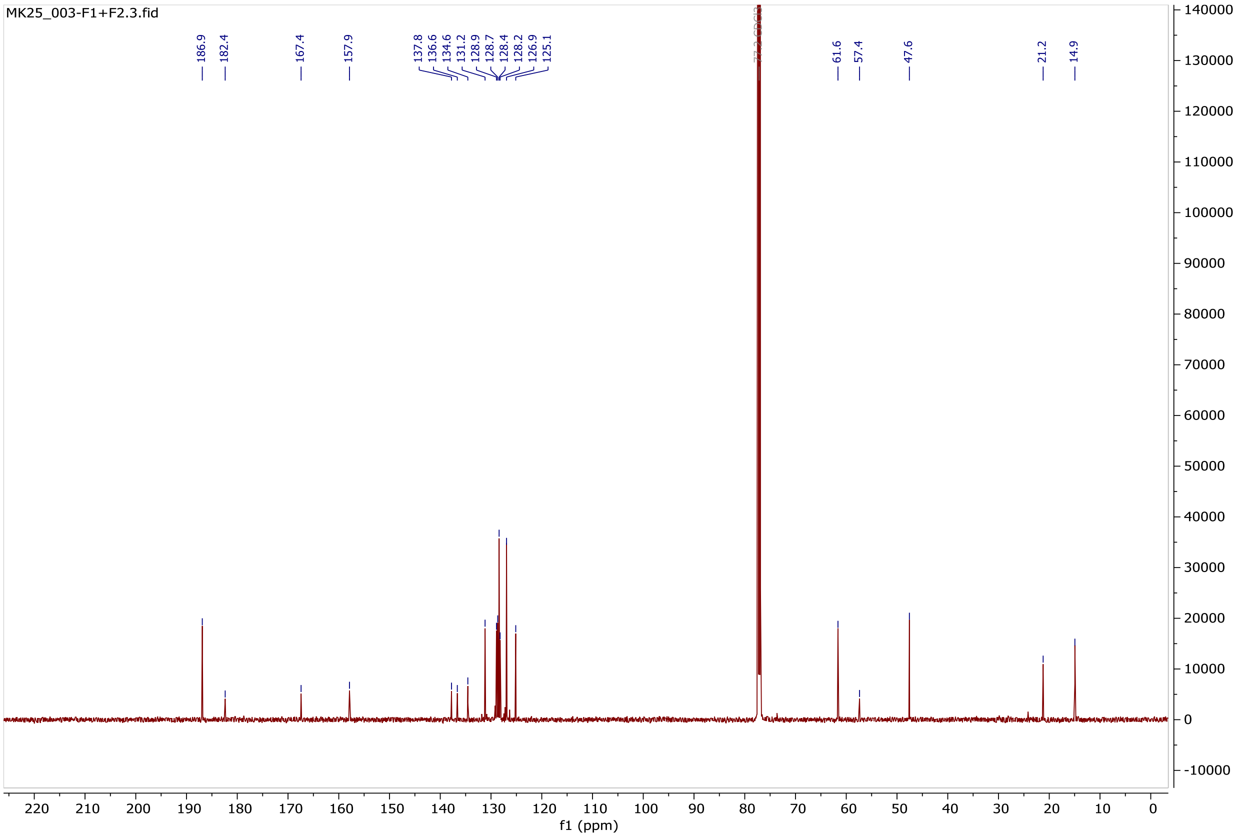

^{13}C {1H} NMR spectrum of **3f - minor** ($CDCl_3$, 151 MHz).

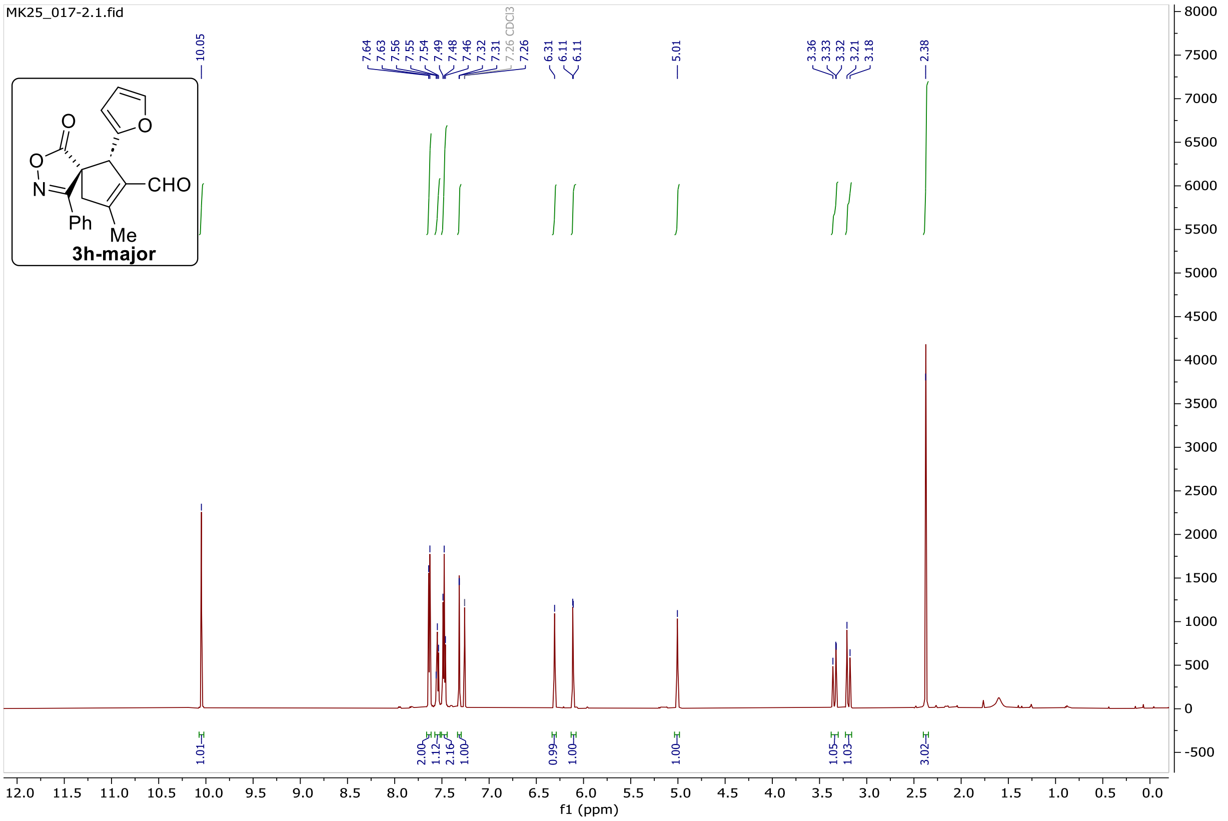

^{1}H NMR spectrum of **3h - major** ($CDCl_3$, 600 MHz).

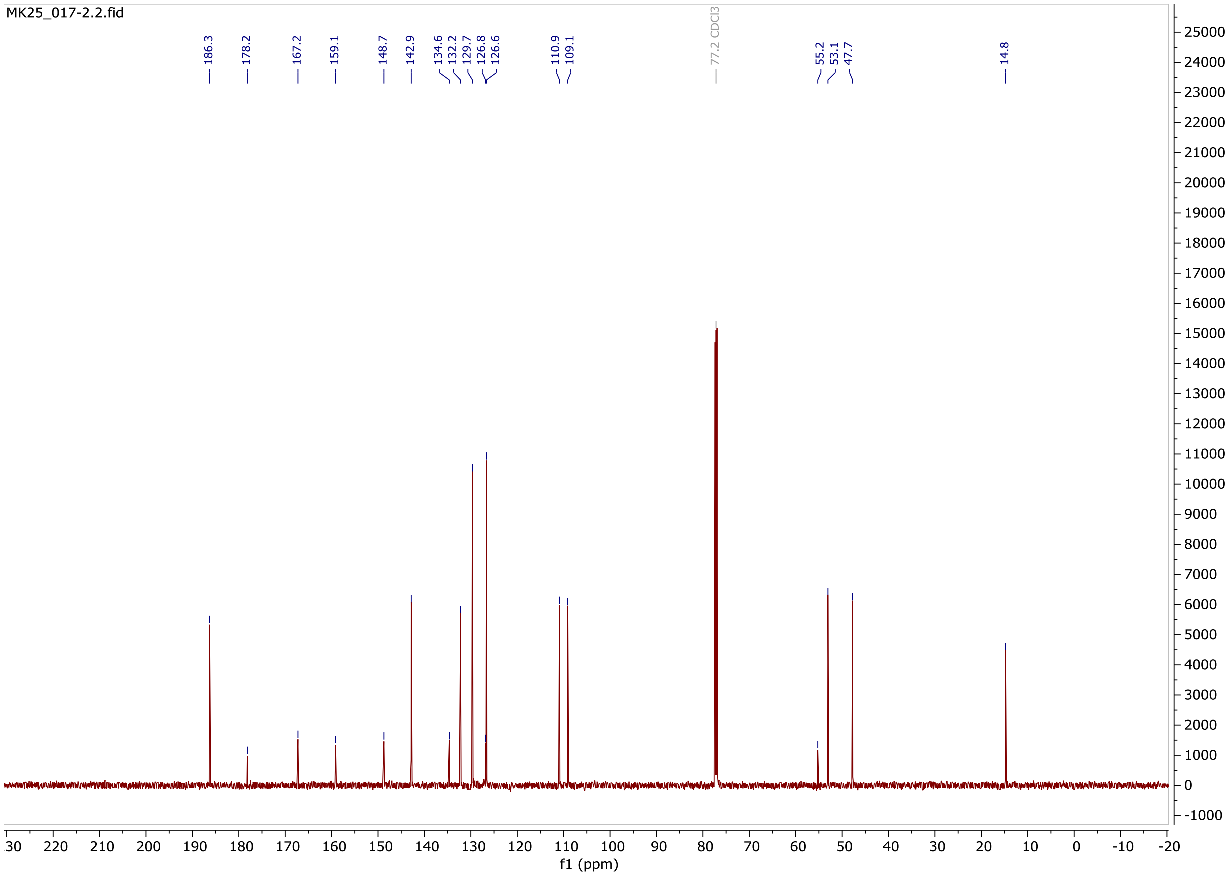

^{13}C {1H} NMR spectrum of **3h - major** ($CDCl_3$, 151 MHz).

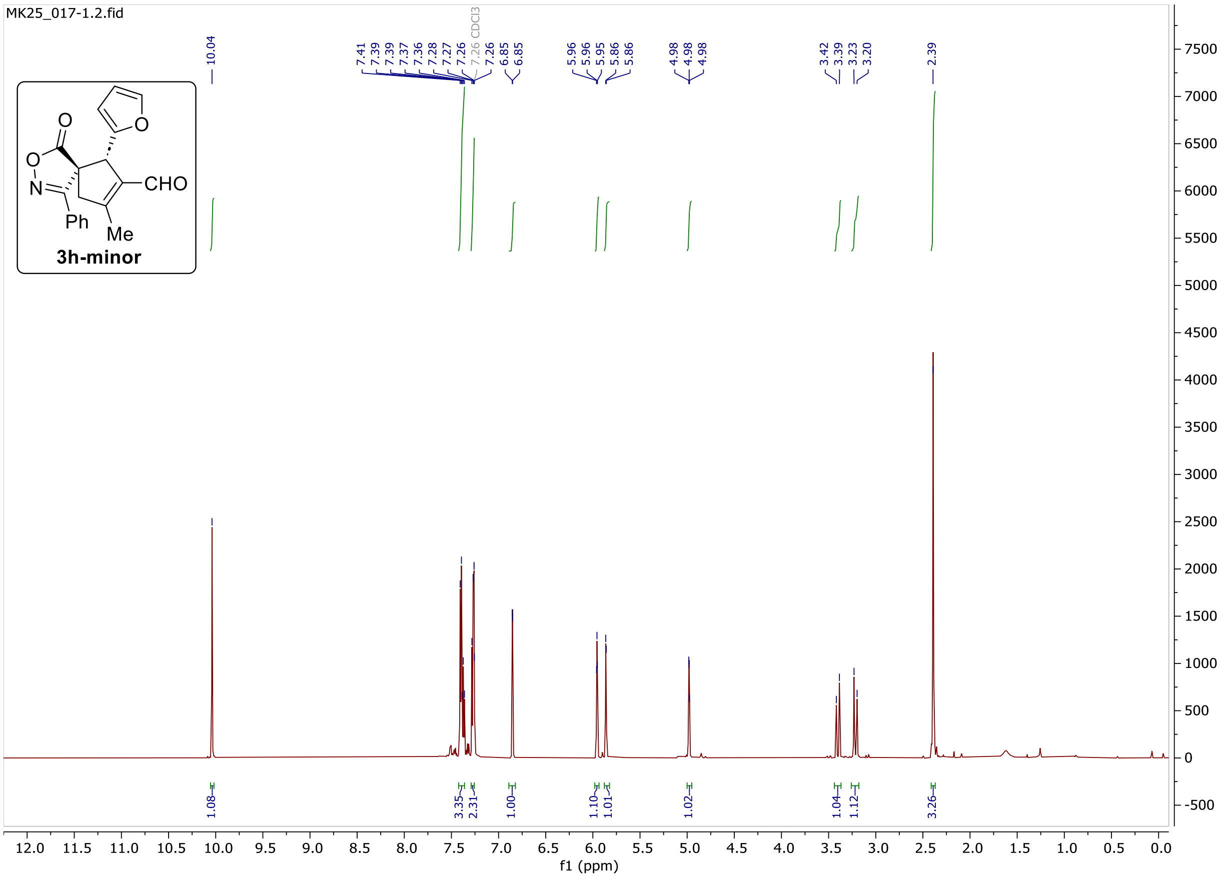

^{1}H NMR spectrum of **3h - minor** ($CDCl_3$, 600 MHz).

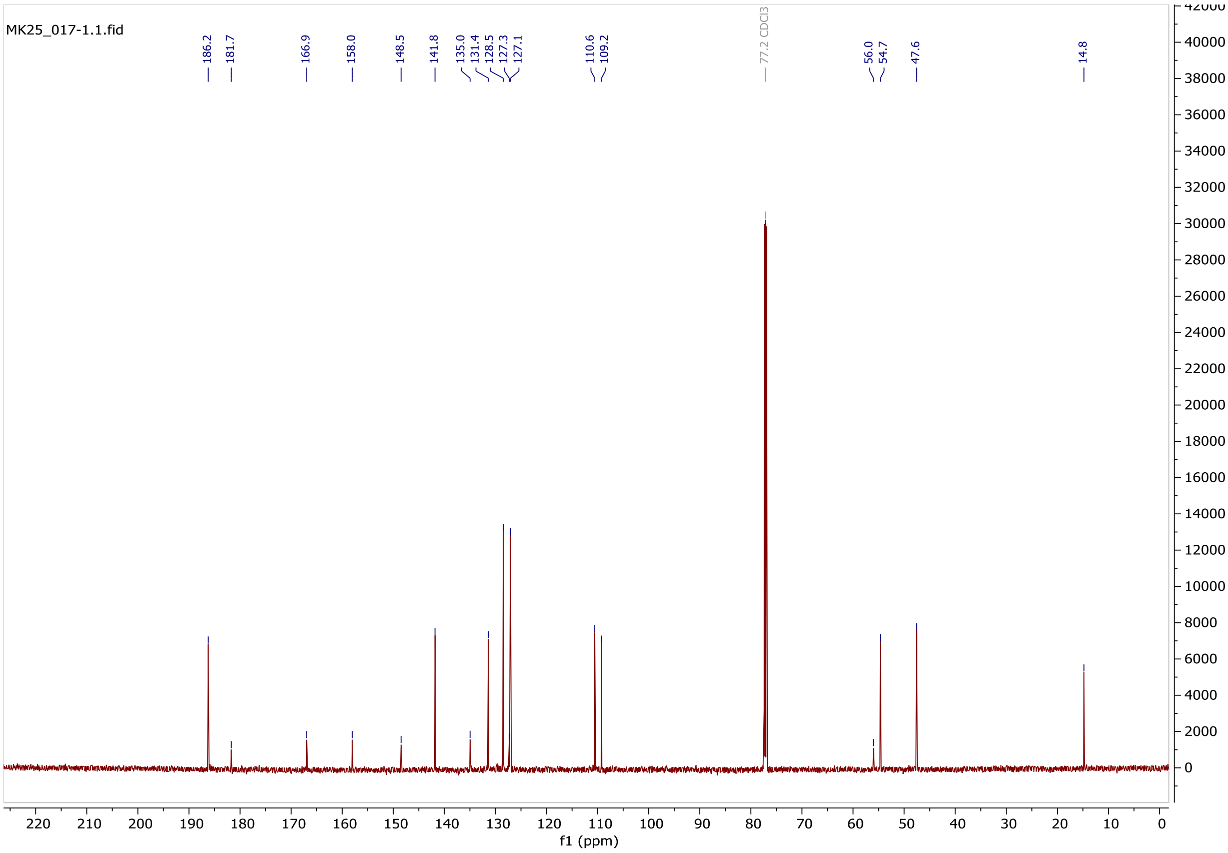

^{13}C {1H} NMR spectrum of **3h - minor** ($CDCl_3$, 151 MHz).

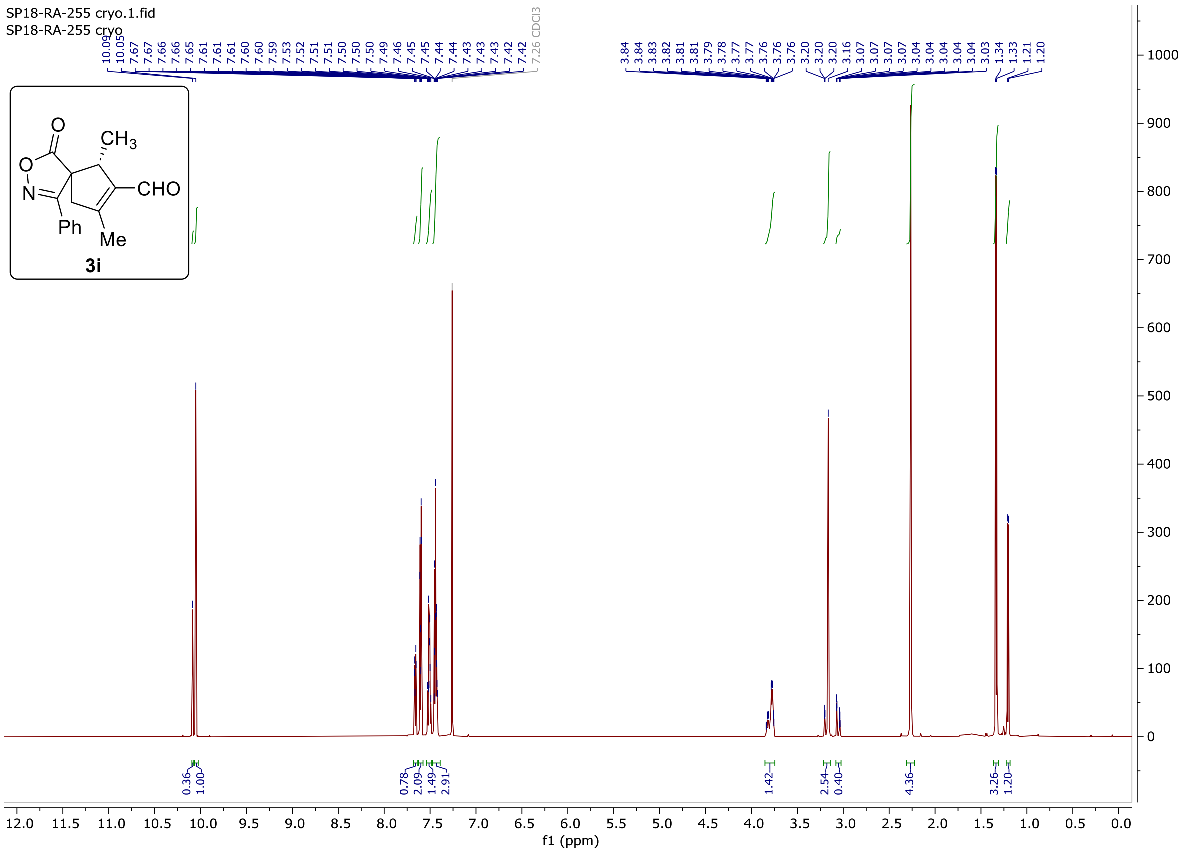

^{1}H NMR spectrum of **3i** ($CDCl_3$, 600 MHz).

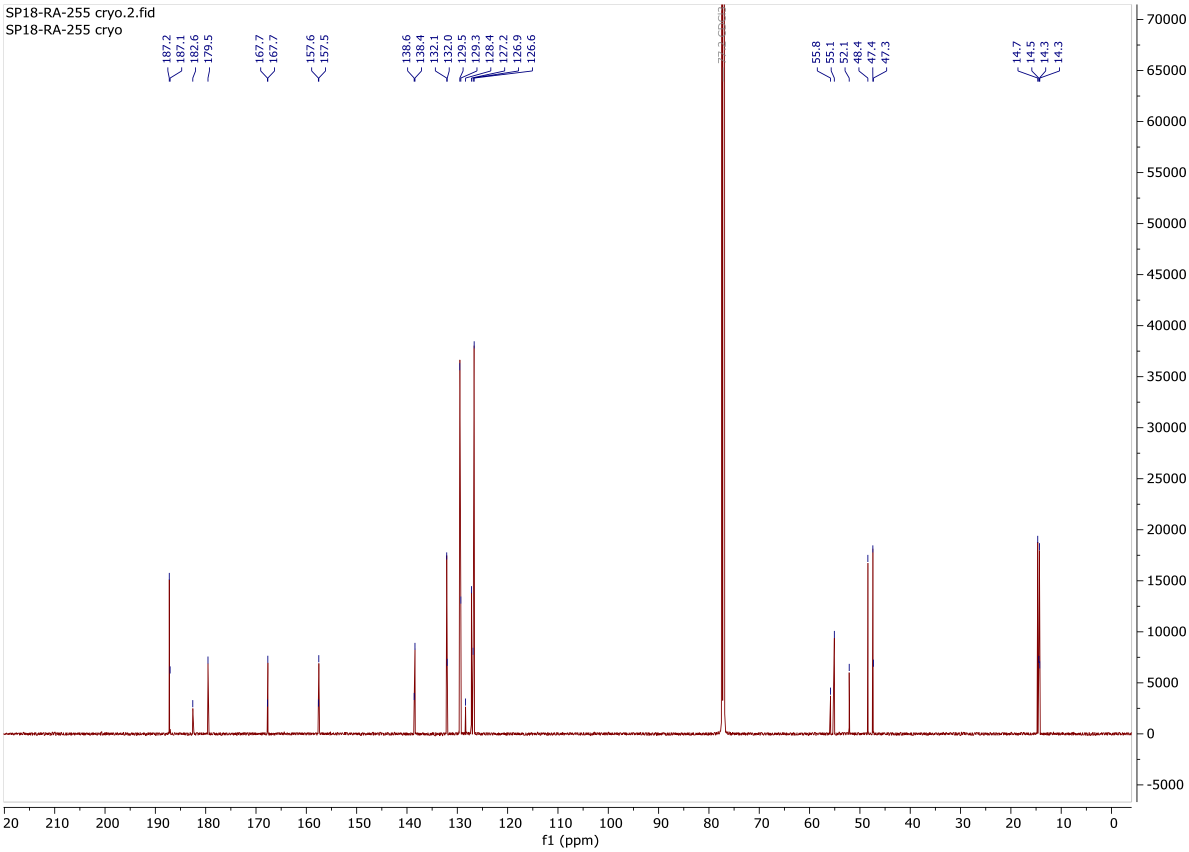

^{13}C {1H} NMR spectrum of **3i** ($CDCl_3$, 151 MHz).

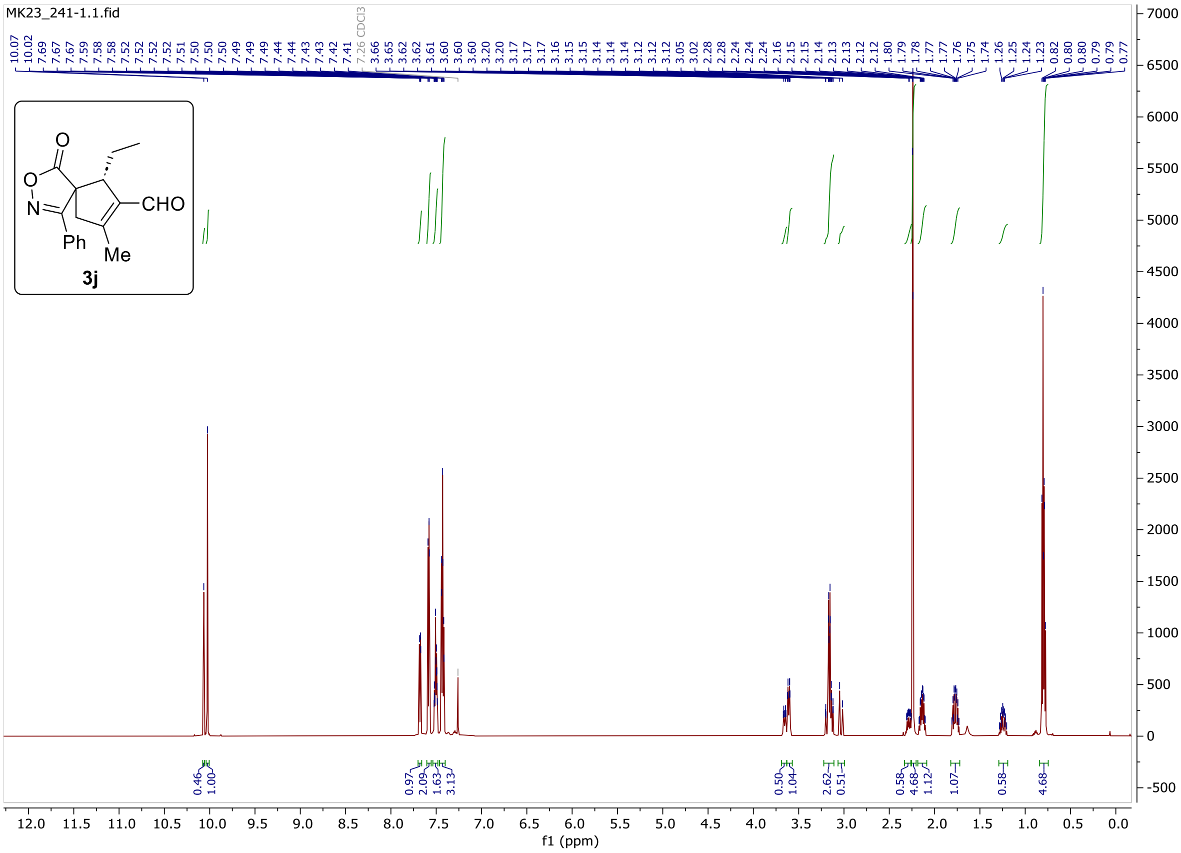

^{1}H NMR spectrum of **3j** ($CDCl_3$, 600 MHz).

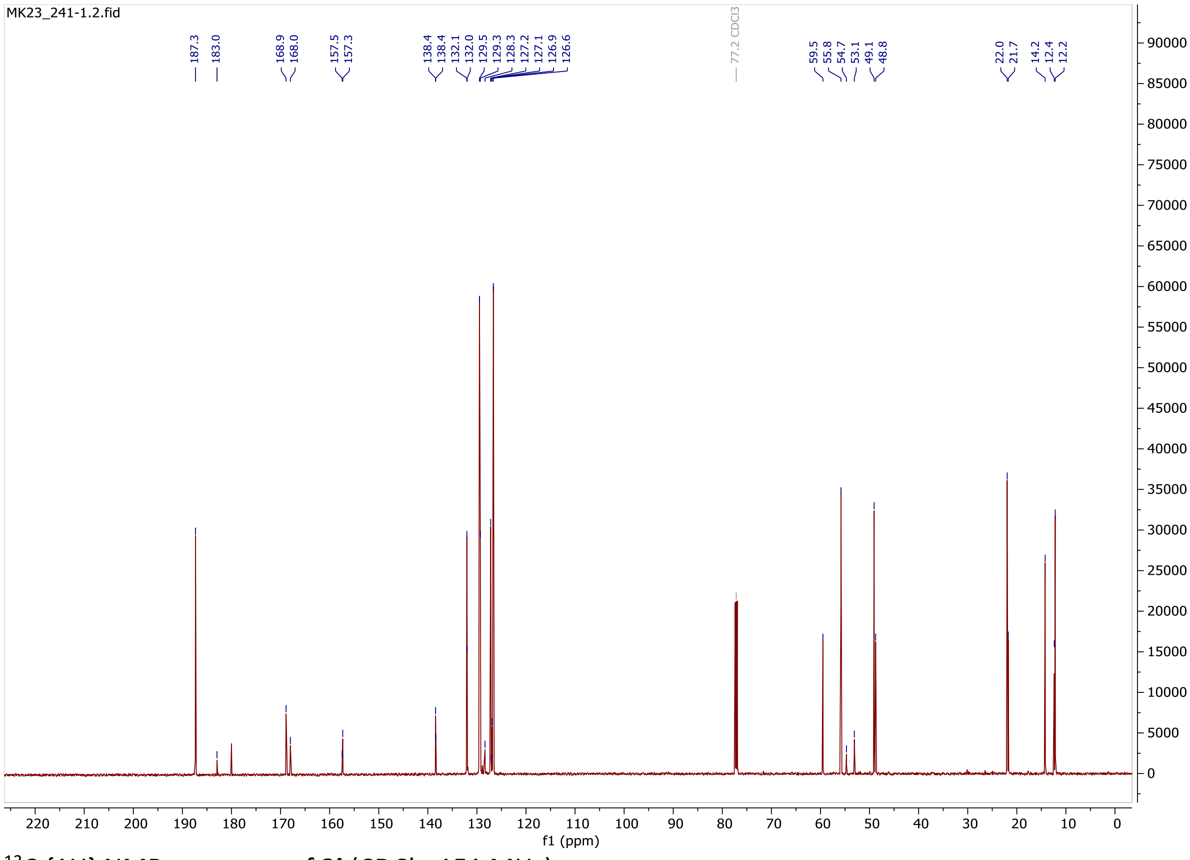

^{13}C {1H} NMR spectrum of **3j** ($CDCl_3$, 151 MHz).

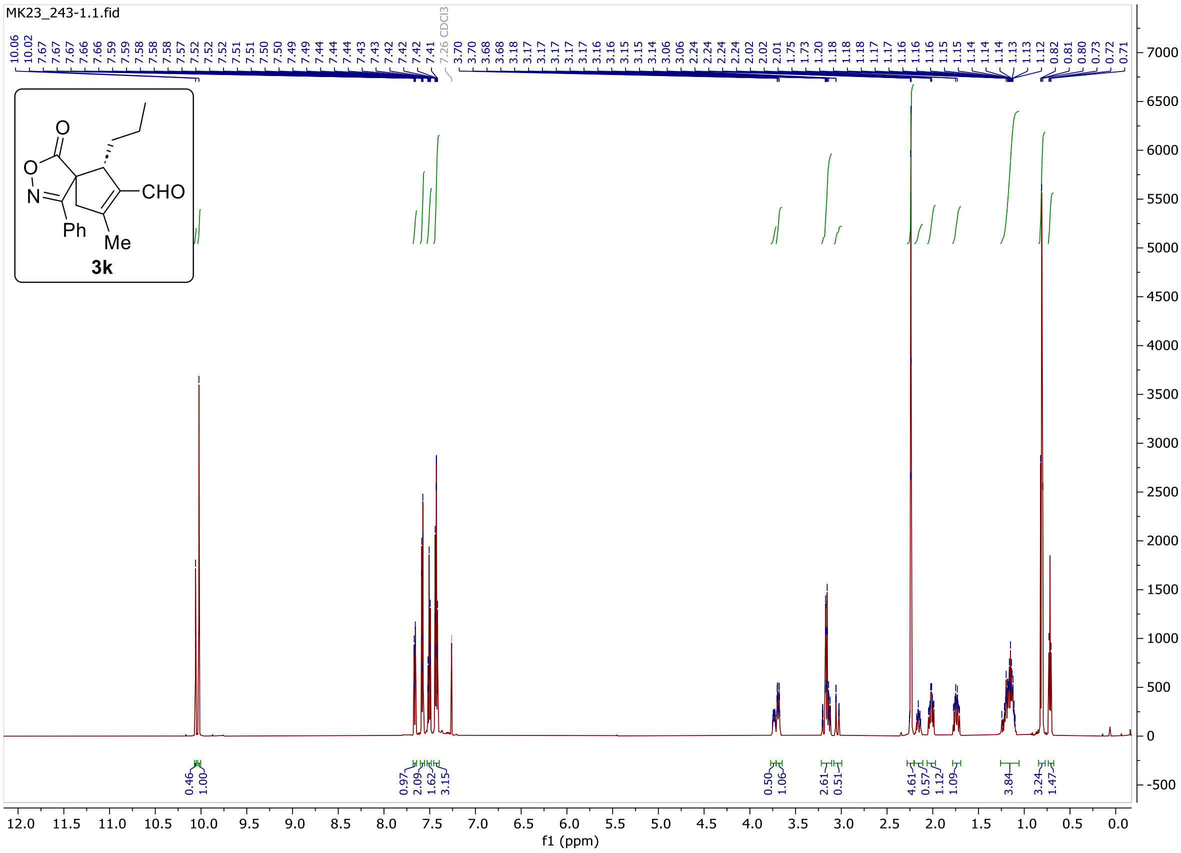

^{1}H NMR spectrum of **3k** ($CDCl_3$, 600 MHz).

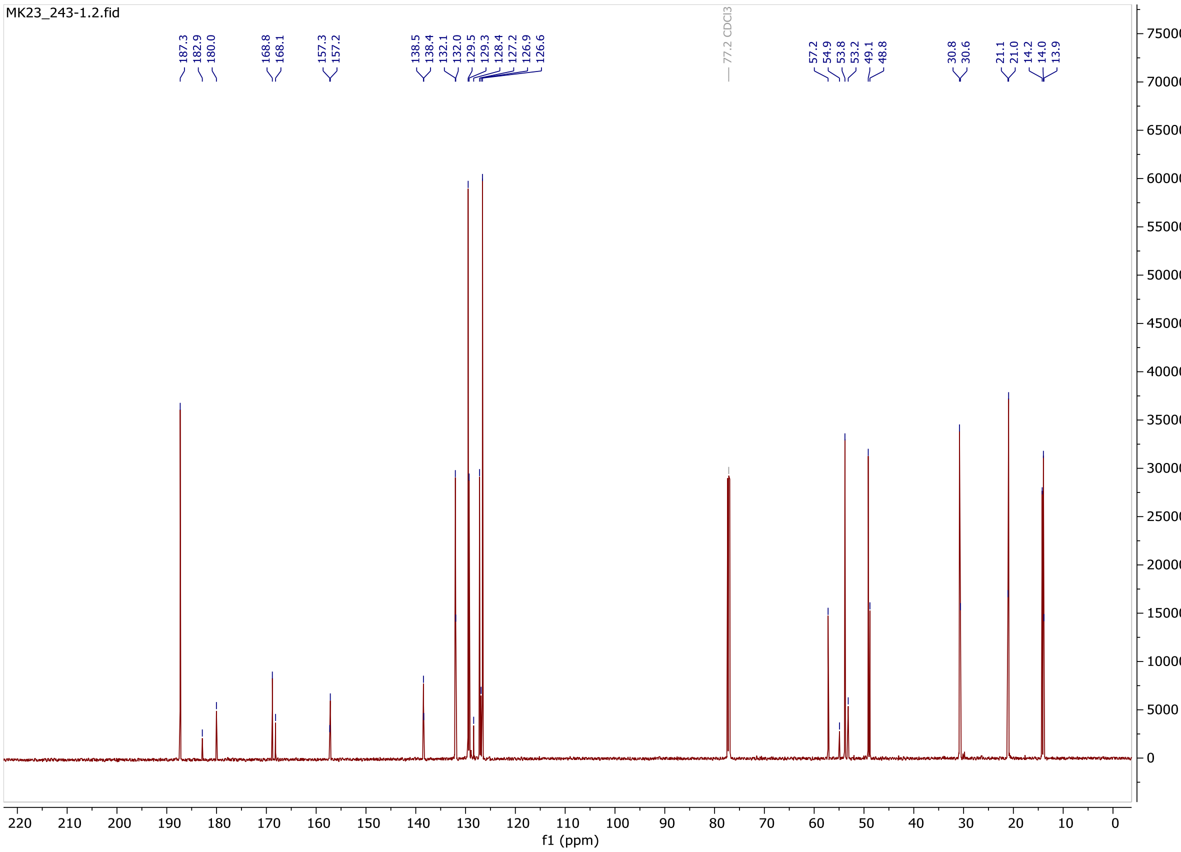

^{13}C {1H} NMR spectrum of **3k** ($CDCl_3$, 151 MHz).

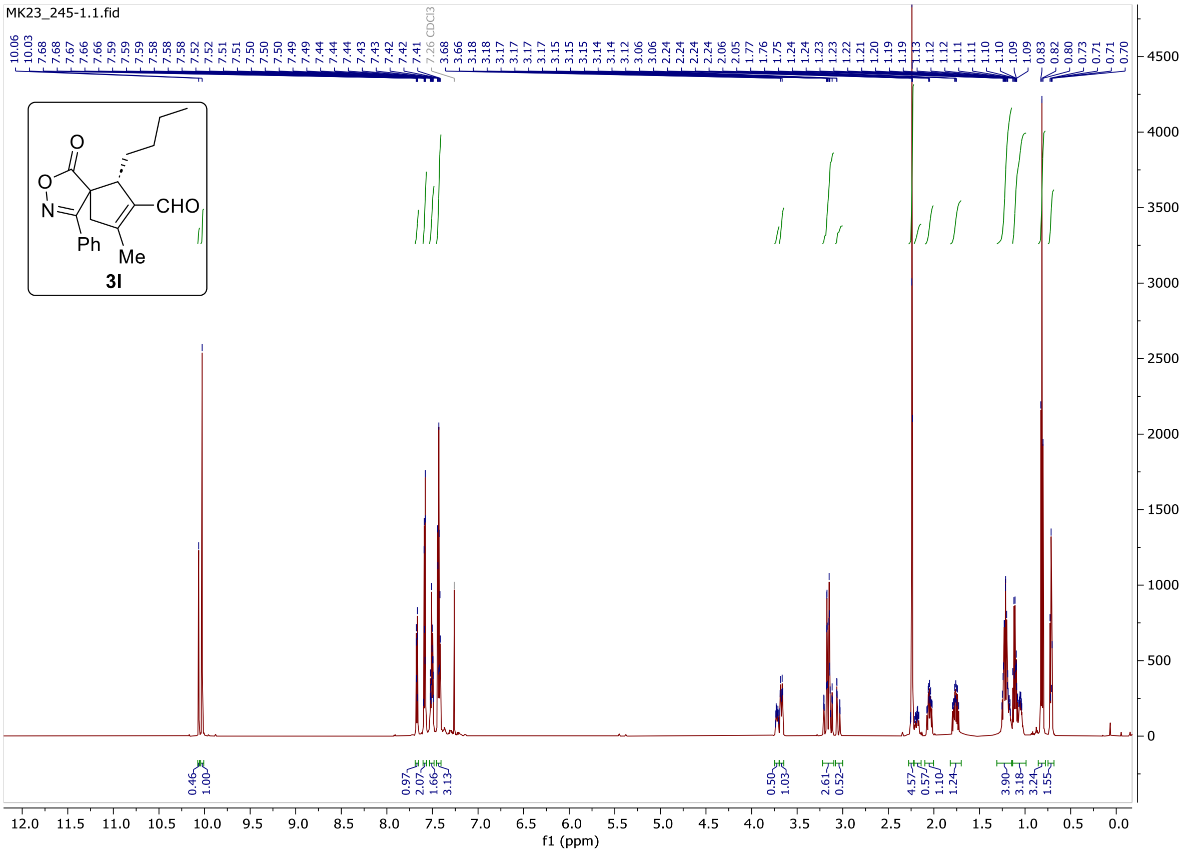

^{1}H NMR spectrum of **3l** ($CDCl_3$, 600 MHz).

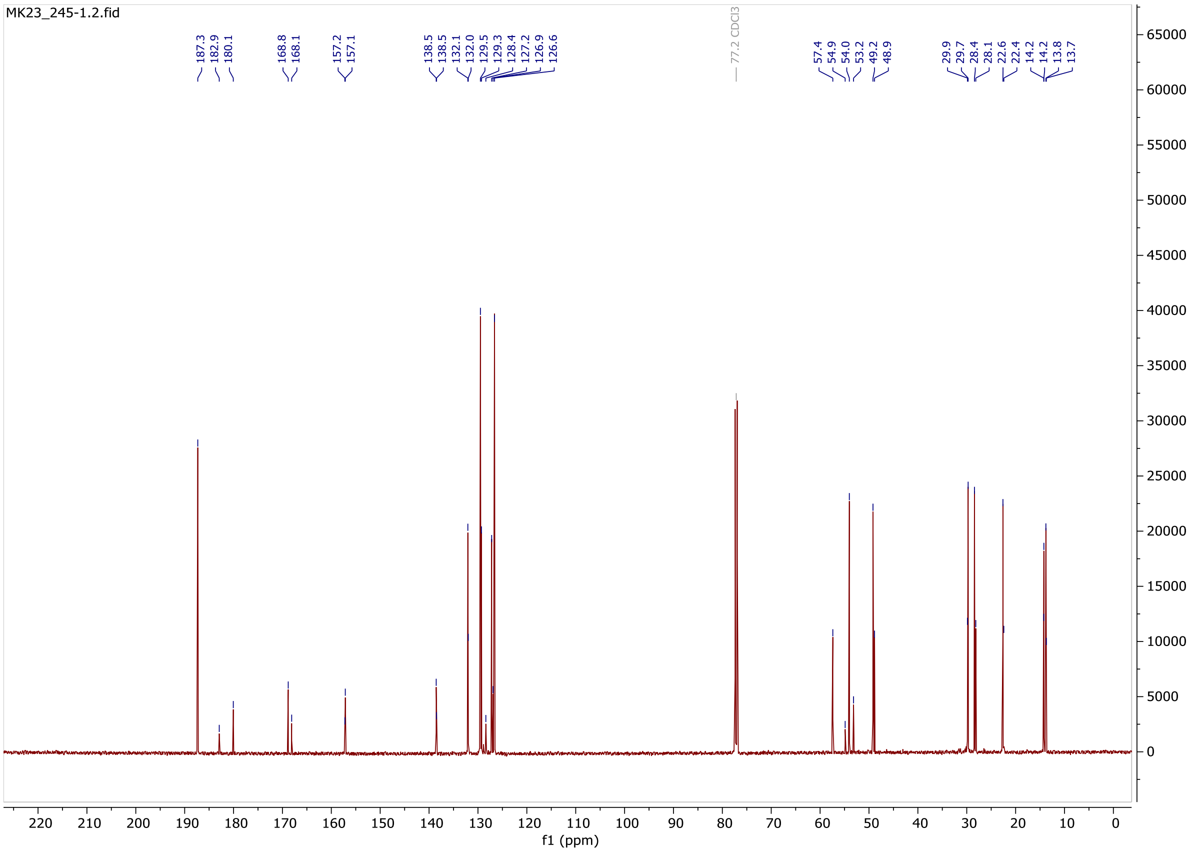

^{13}C {1H} NMR spectrum of **3l** ($CDCl_3$, 151 MHz).

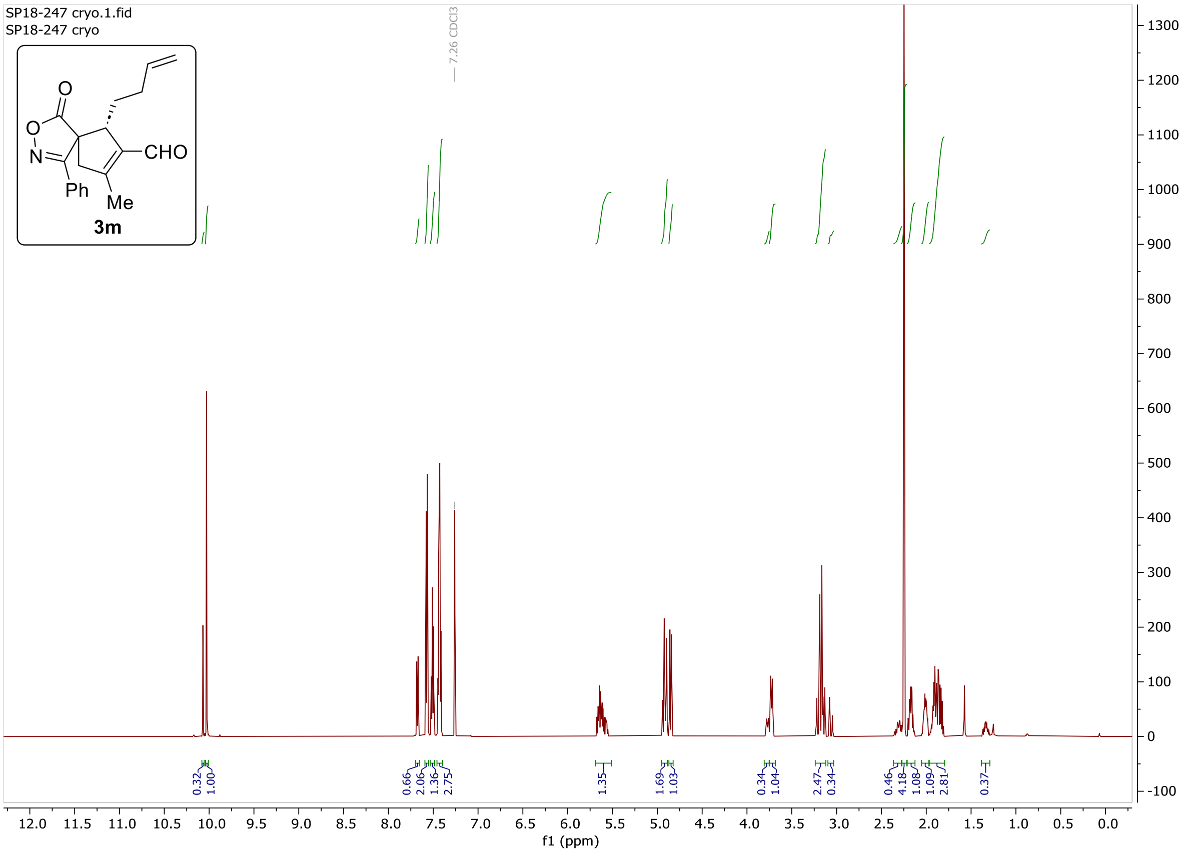

^{1}H NMR spectrum of **3m** ($CDCl_3$, 600 MHz).

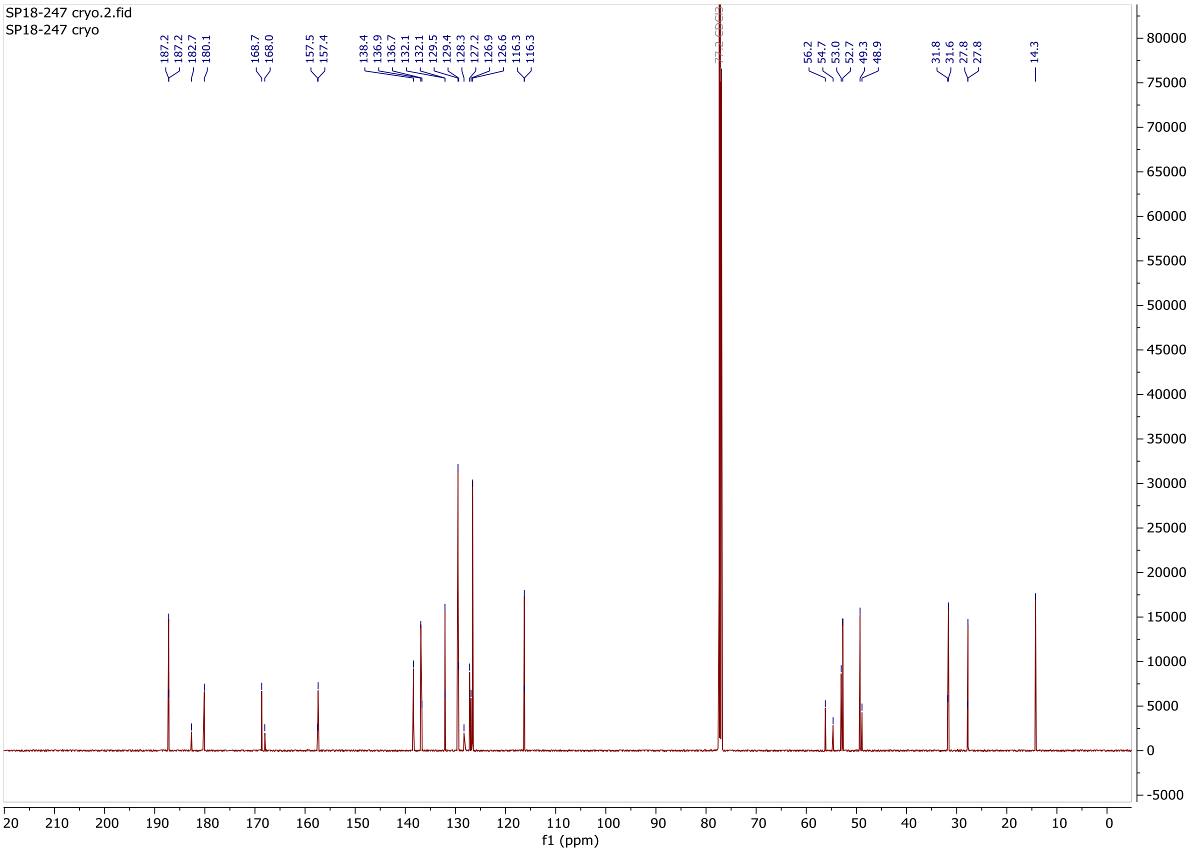

^{13}C {1H} NMR spectrum of **3m** ($CDCl_3$, 151 MHz).

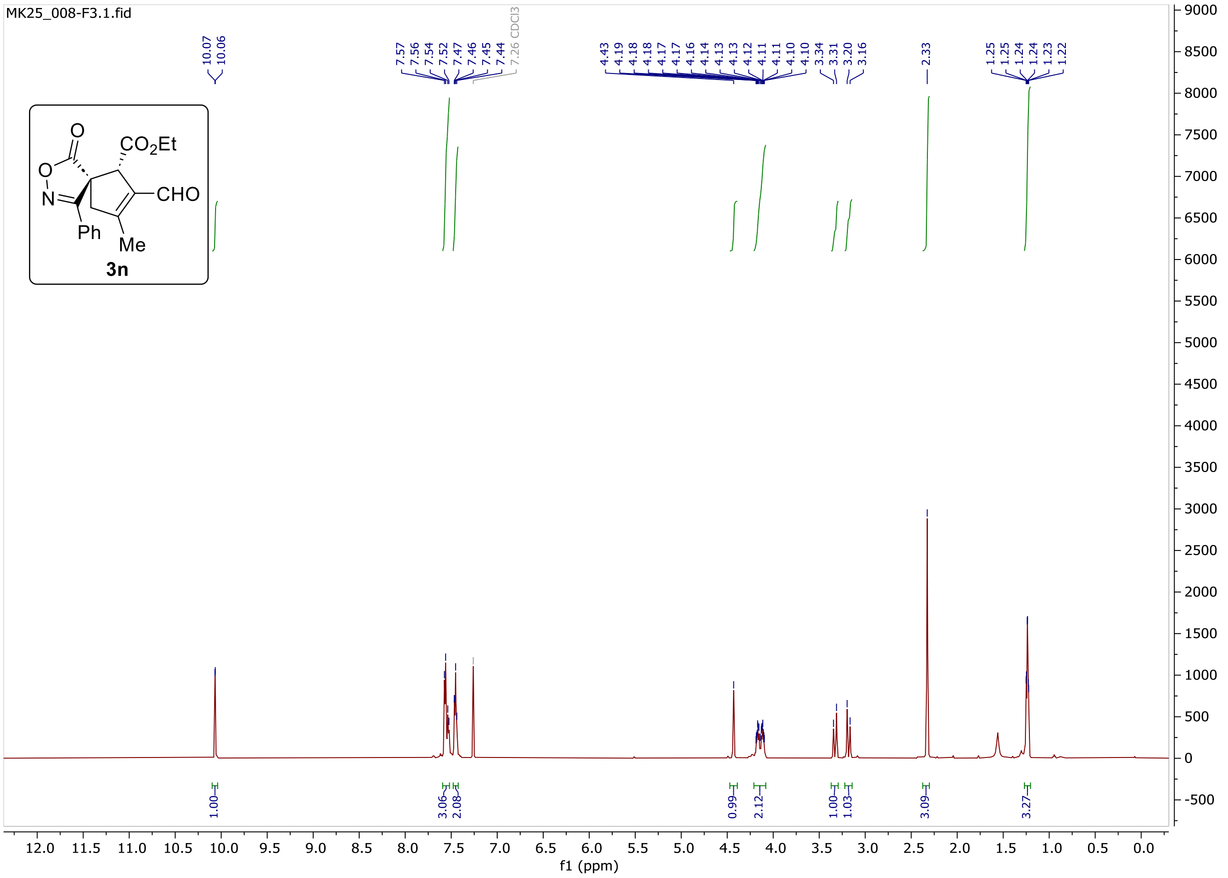

^{1}H NMR spectrum of **3n - major** ($CDCl_3$, 600 MHz).

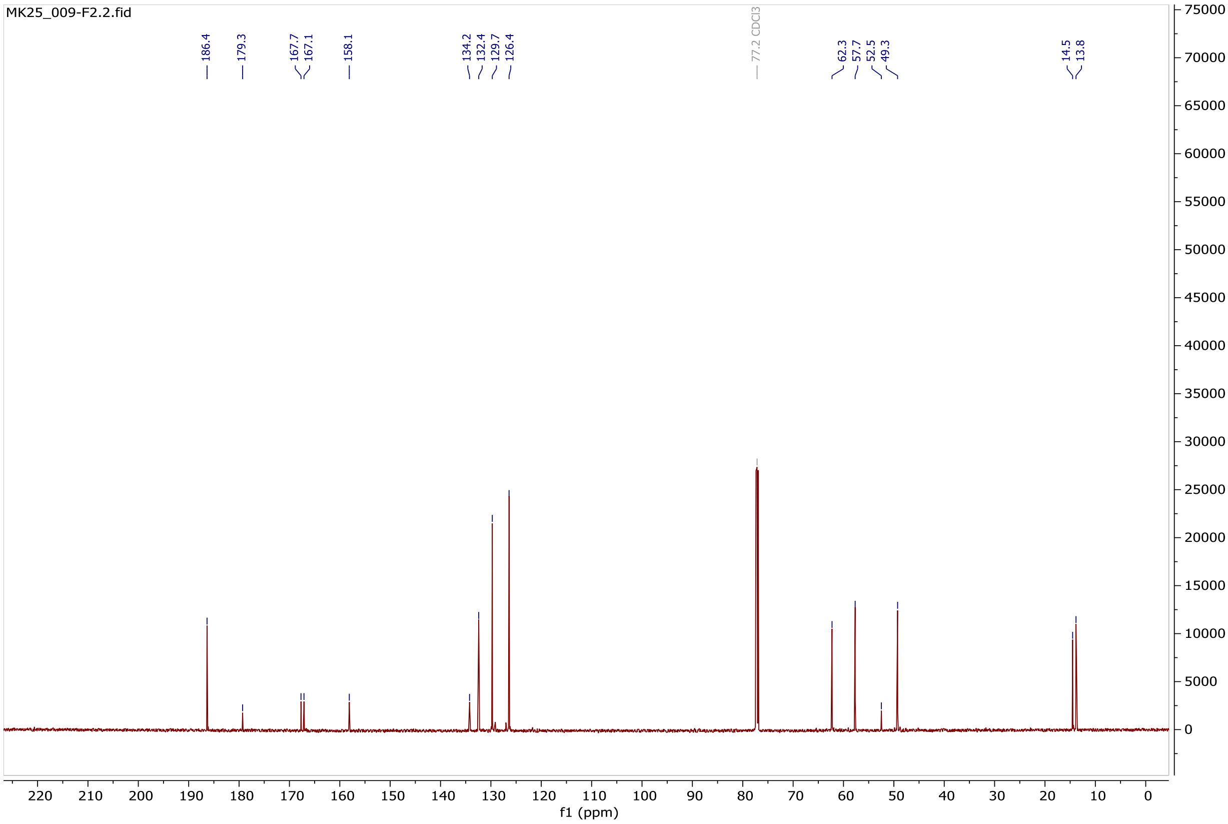

^{13}C {1H} NMR spectrum of **3n – major** ($CDCl_3$, 151 MHz).

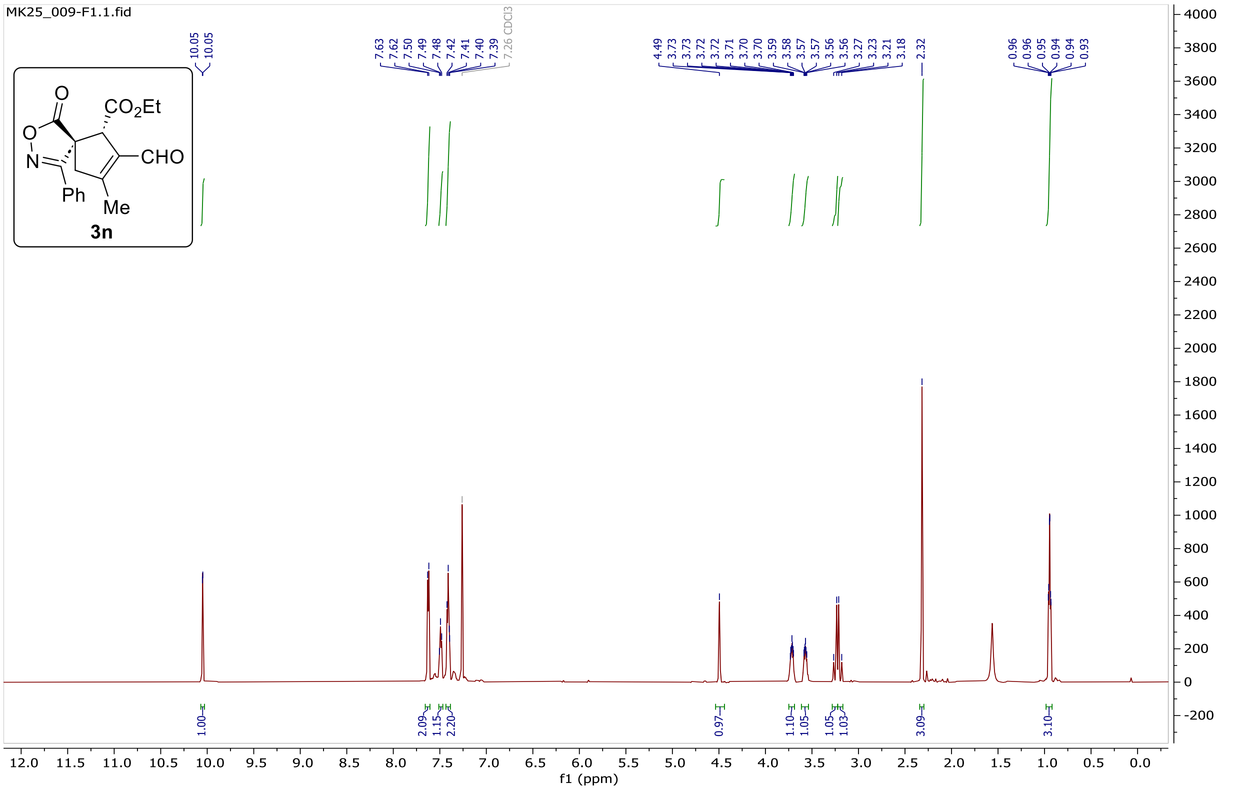

^{1}H NMR spectrum of **3n - minor** ($CDCl_3$, 600 MHz).

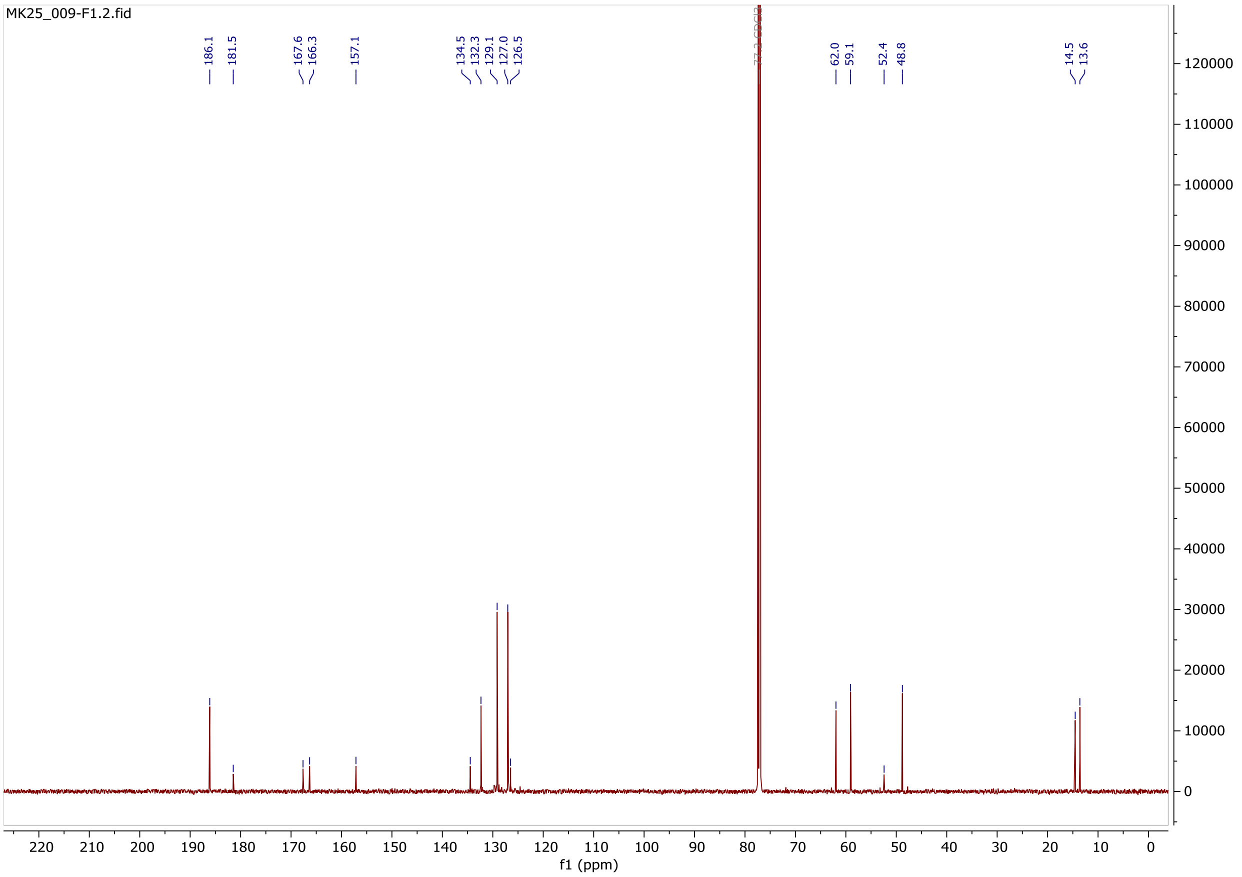

^{13}C {1H} NMR spectrum of **3n - minor** ($CDCl_3$, 151 MHz).

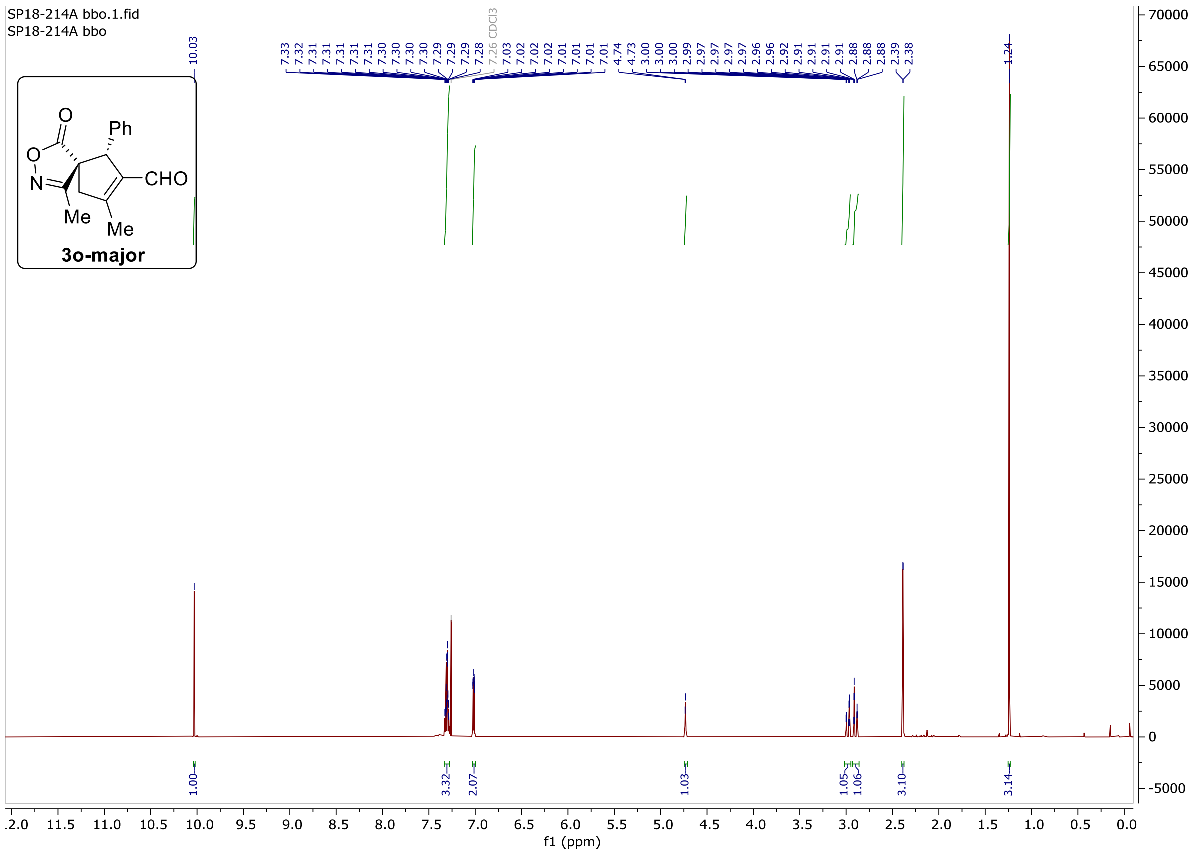

^{1}H NMR spectrum of **3o - major** ($CDCl_3$, 600 MHz).

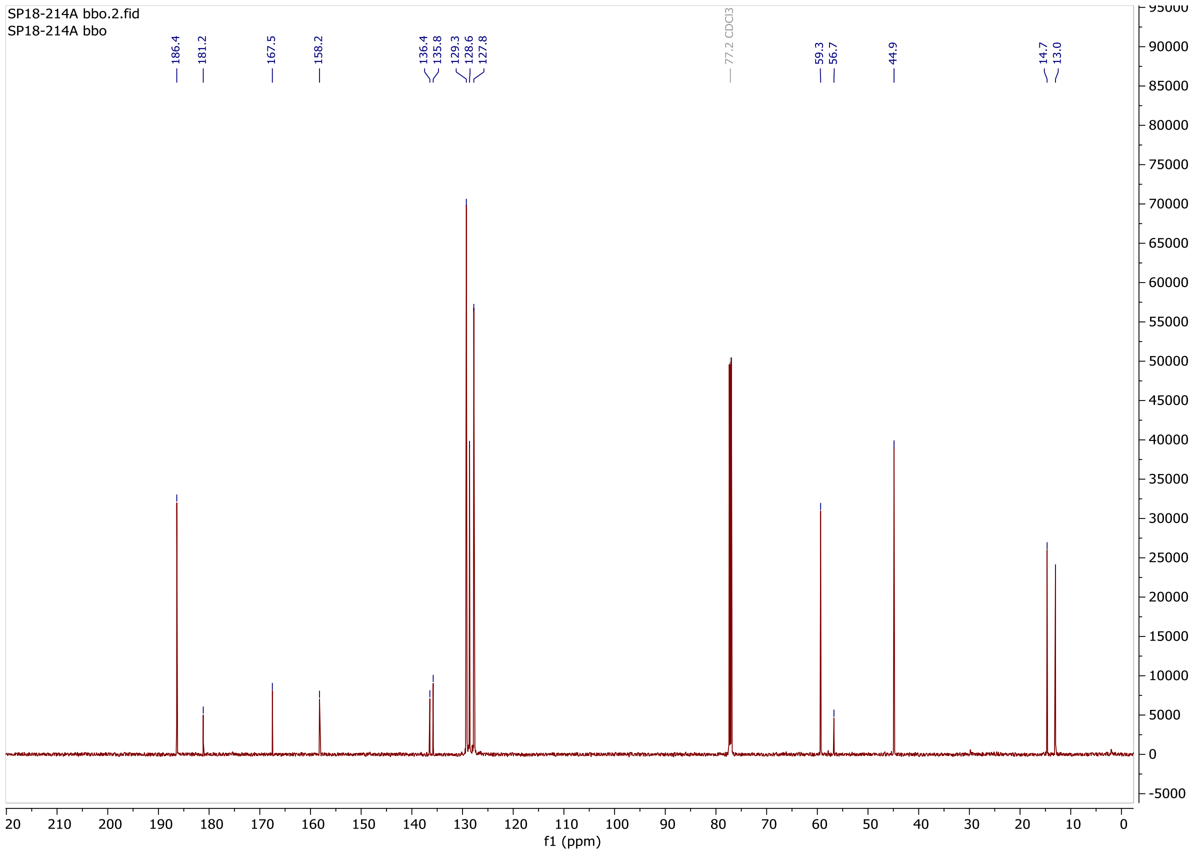

^{13}C {1H} NMR spectrum of **3o - major** ($CDCl_3$, 151 MHz).

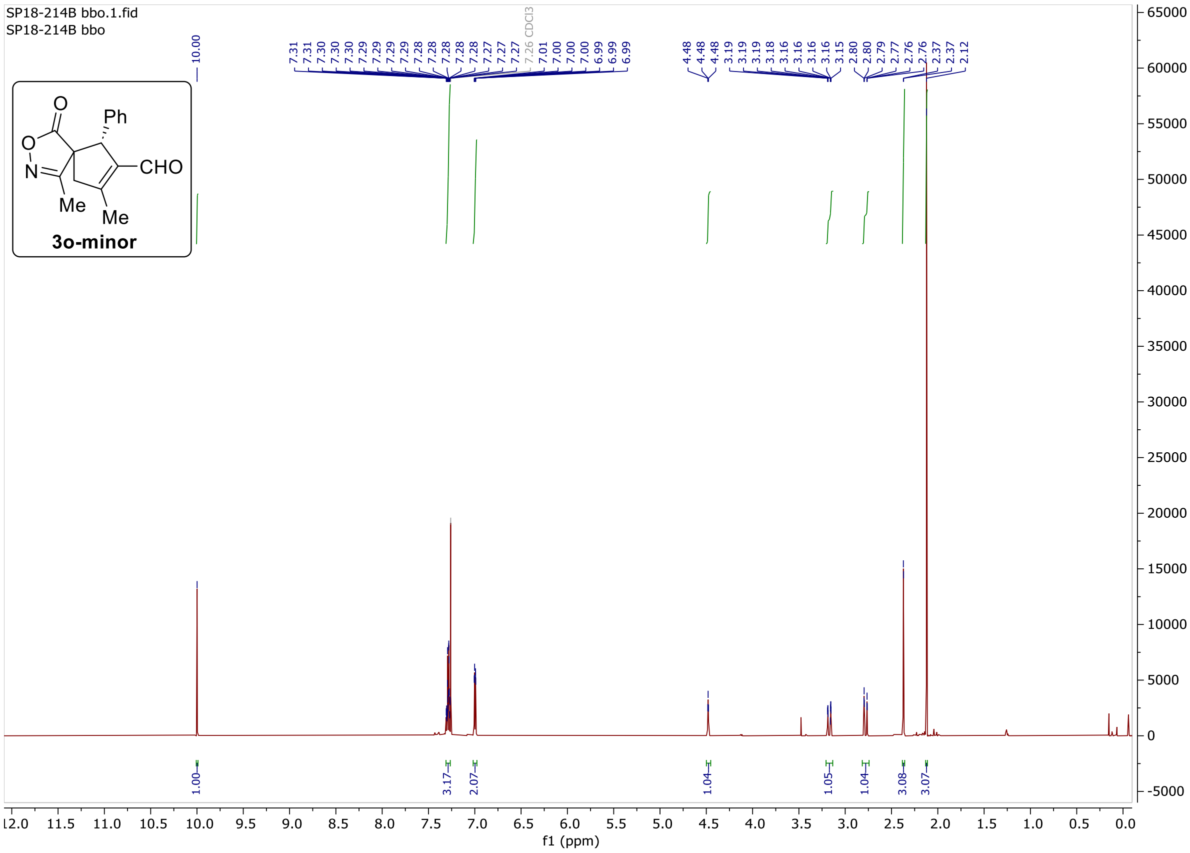

^{1}H NMR spectrum of **3o - minor** ($CDCl_3$, 600 MHz).

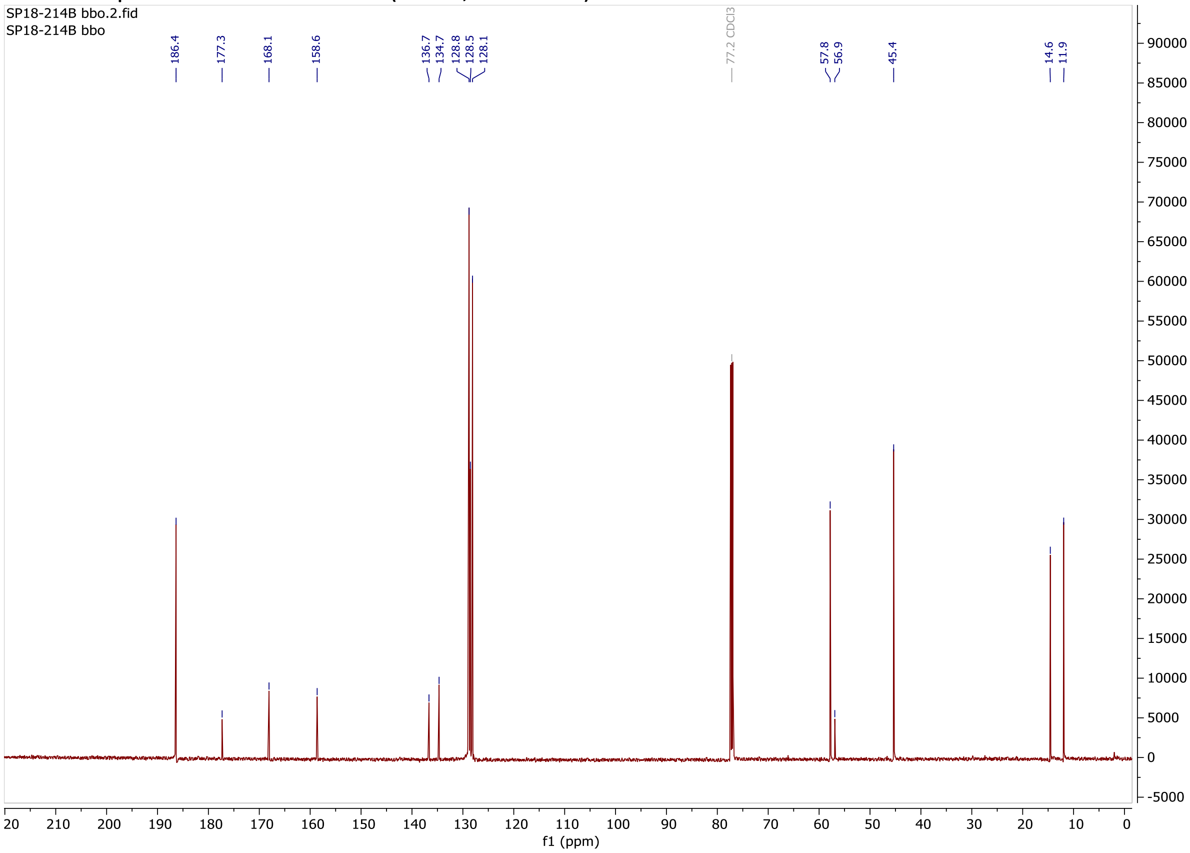

^{13}C {1H} NMR spectrum of **3o - minor** ($CDCl_3$, 151 MHz).

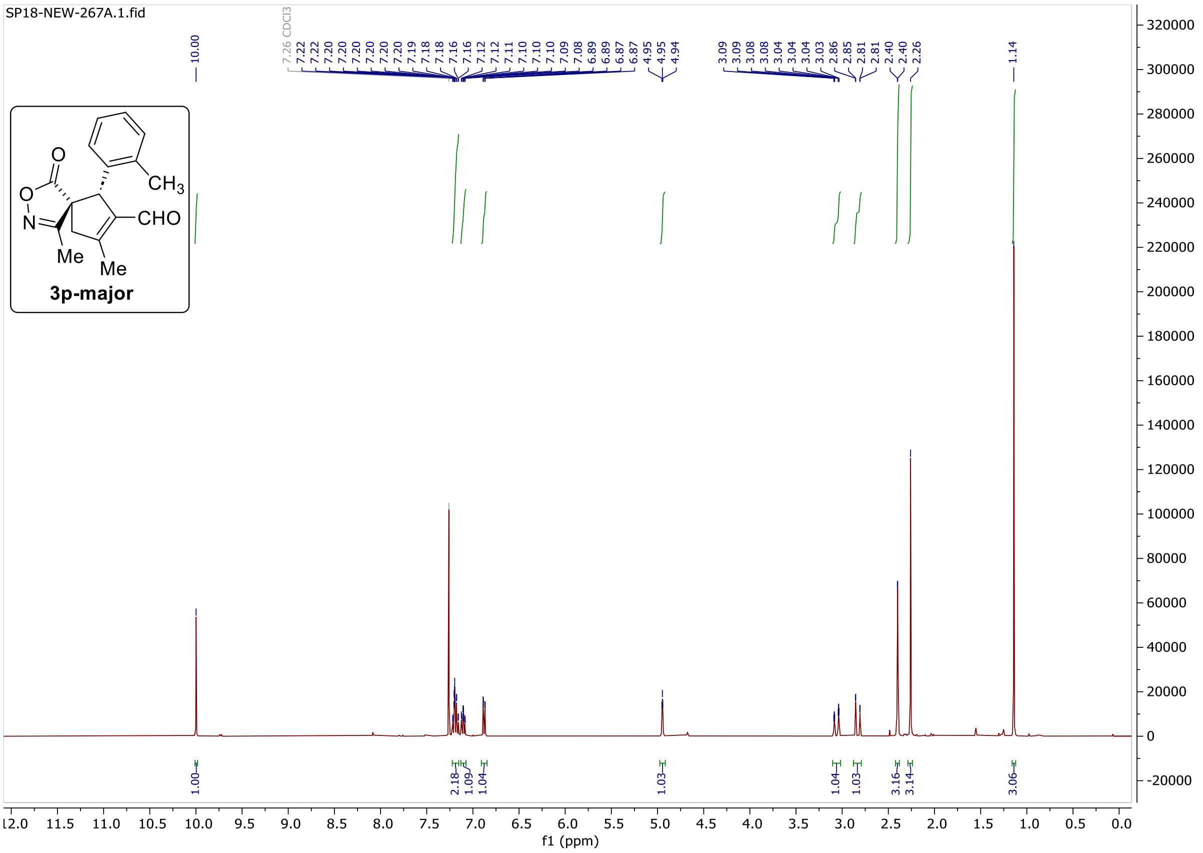

^{1}H NMR spectrum of **3p - major** ($CDCl_3$, 600 MHz).

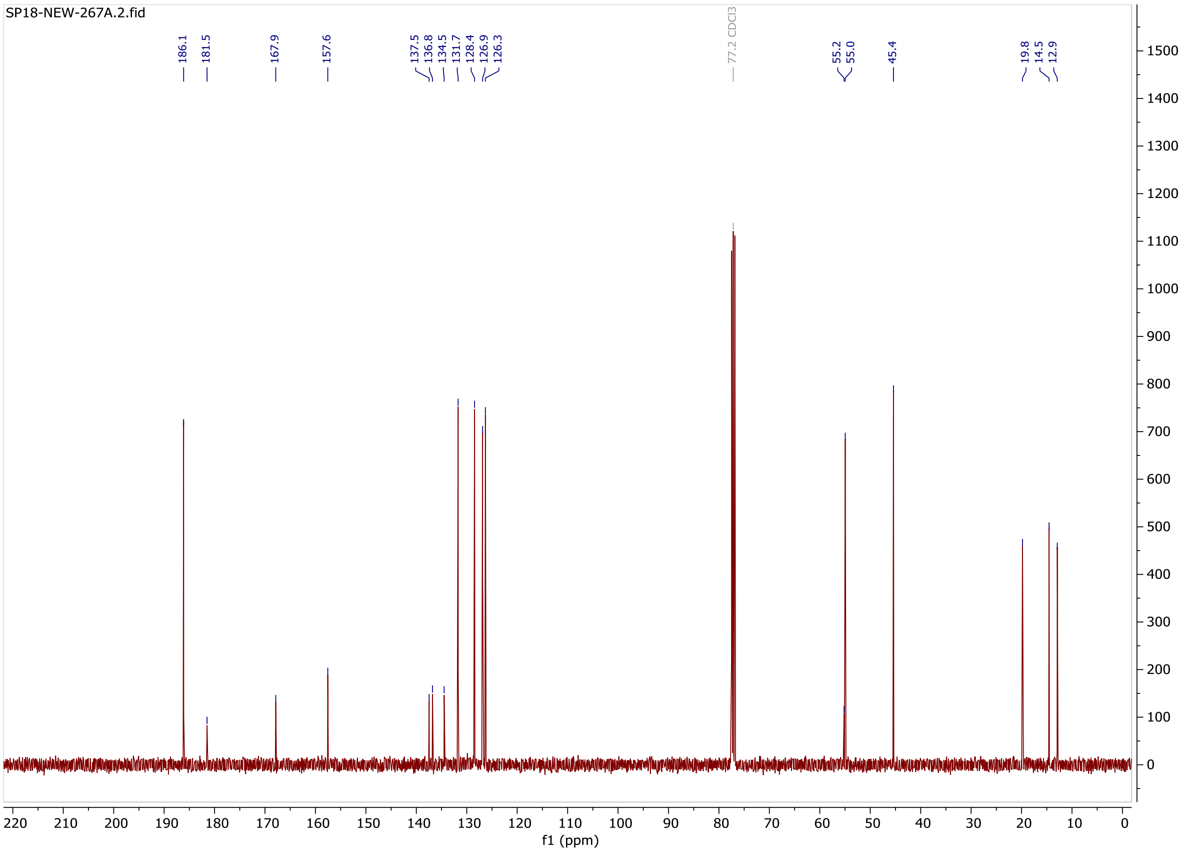

^{13}C {1H} NMR spectrum of **3p - major** ($CDCl_3$, 151 MHz).

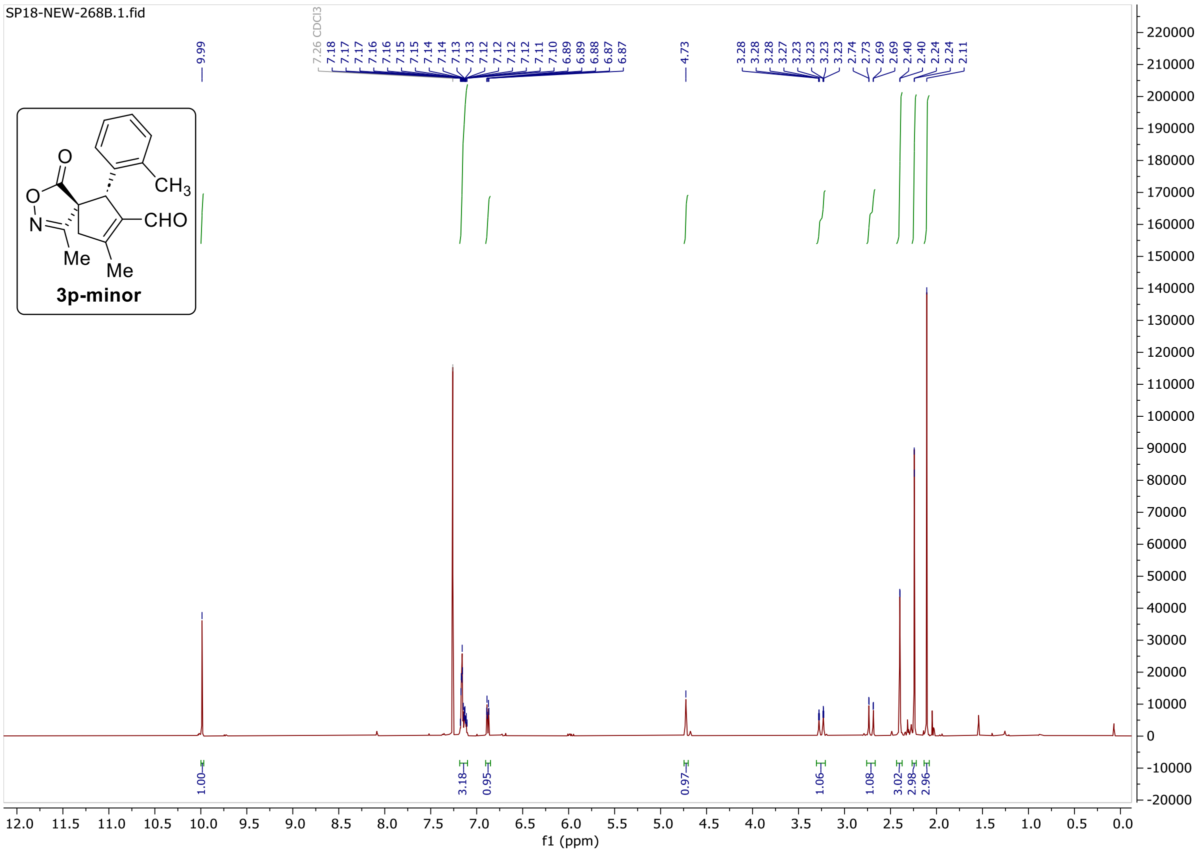

^{1}H NMR spectrum of **3p - minor** ($CDCl_3$, 600 MHz).

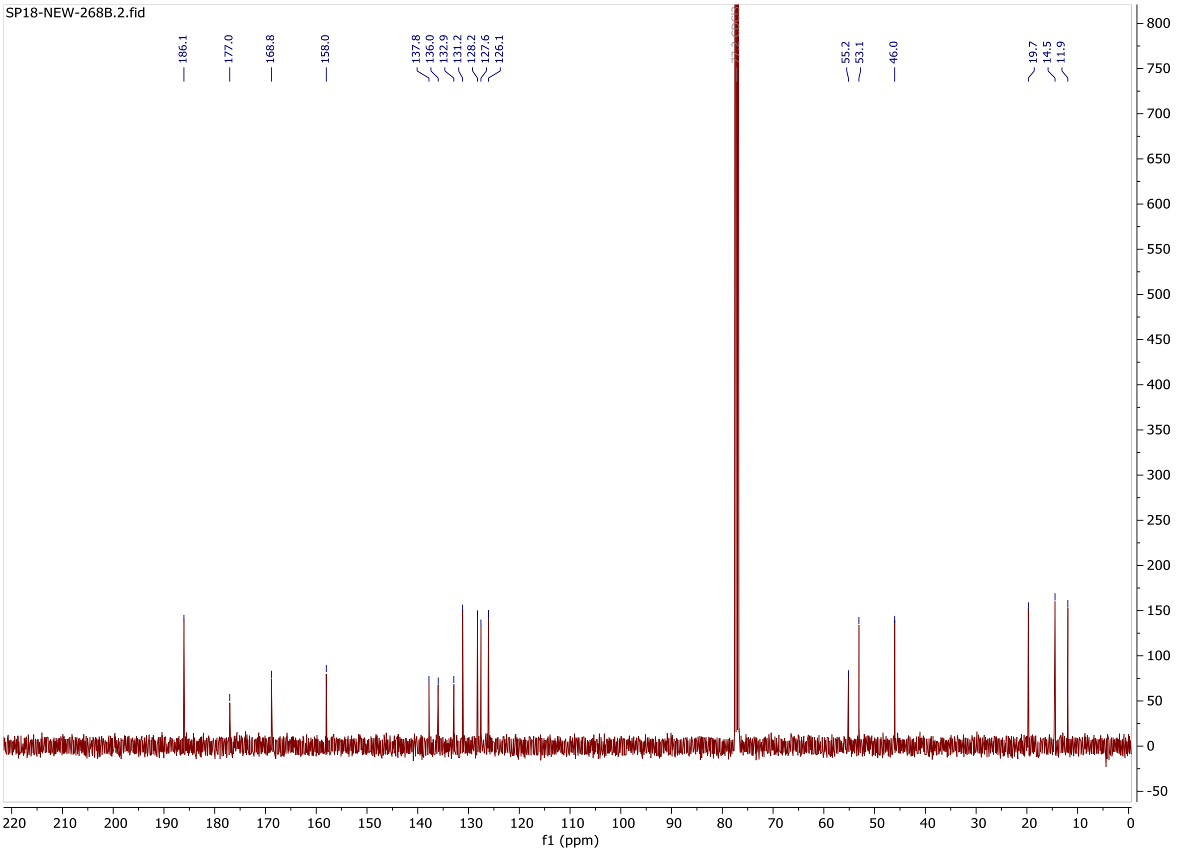

^{13}C {1H} NMR spectrum of **3p - minor** ($CDCl_3$, 151 MHz).

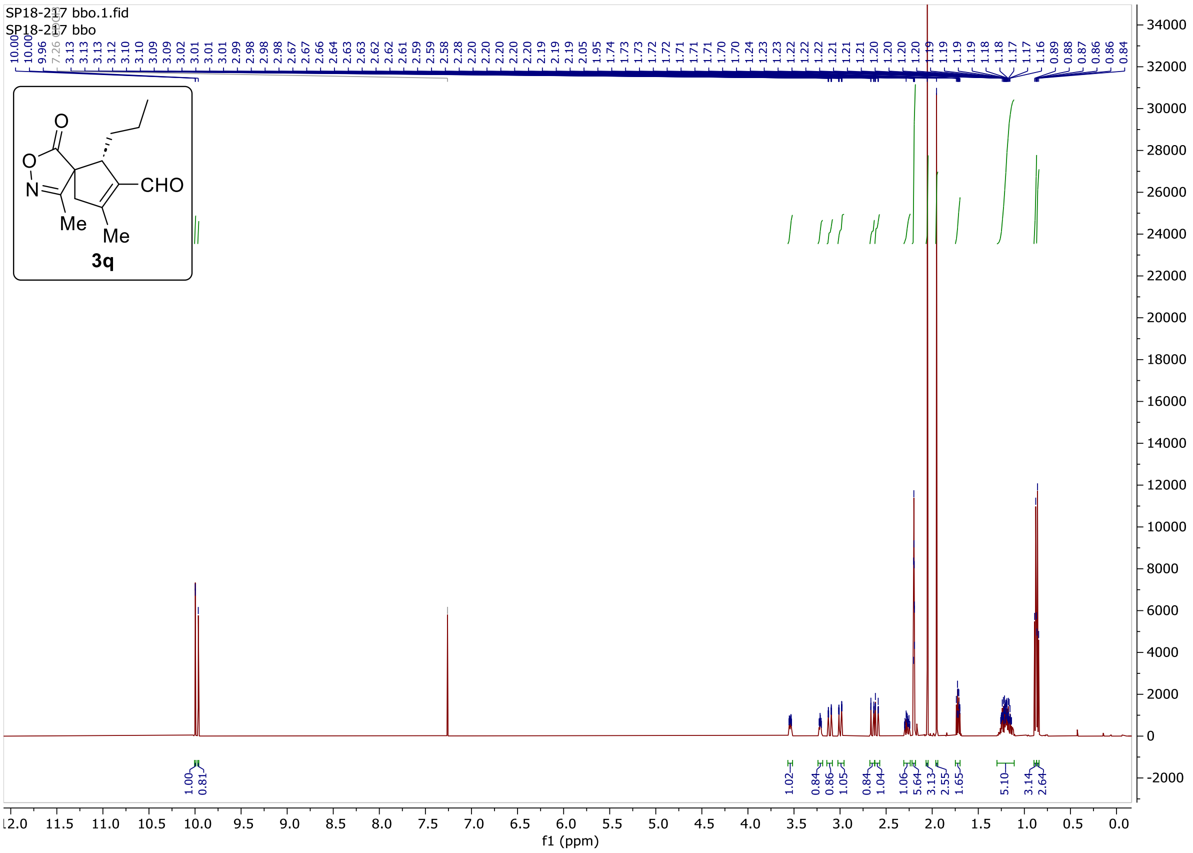

^{1}H NMR spectrum of **3q** ($CDCl_3$, 600 MHz).

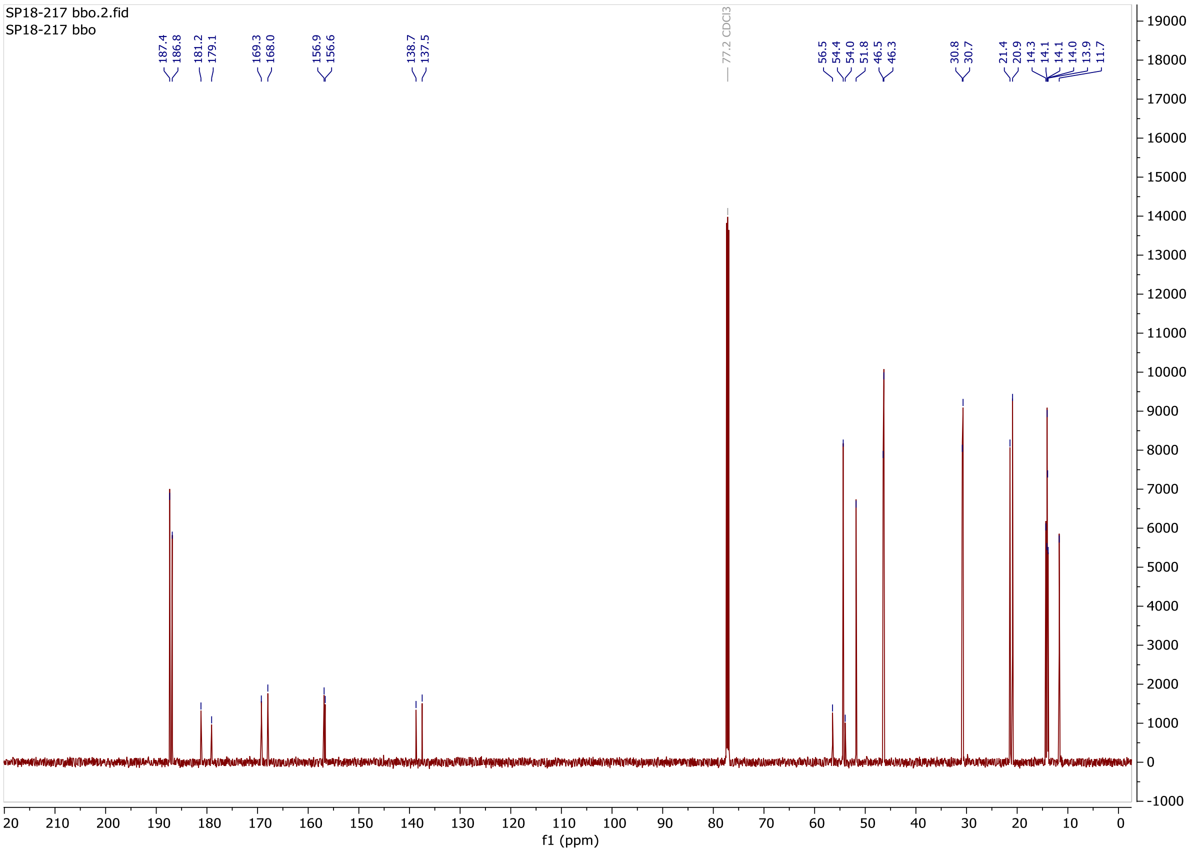

^{13}C {1H} NMR spectrum of **3q** ($CDCl_3$, 151 MHz).

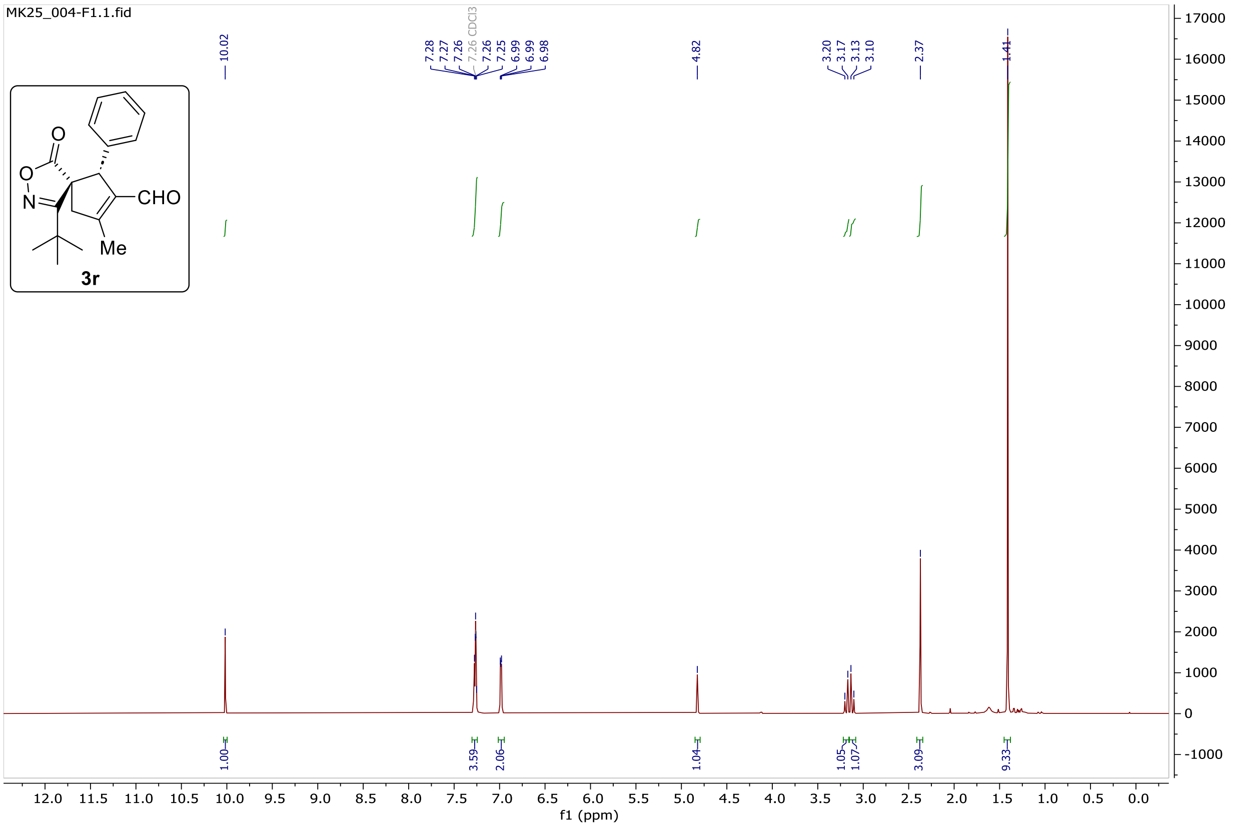

^{1}H NMR spectrum of **3r** ($CDCl_3$, 600 MHz).

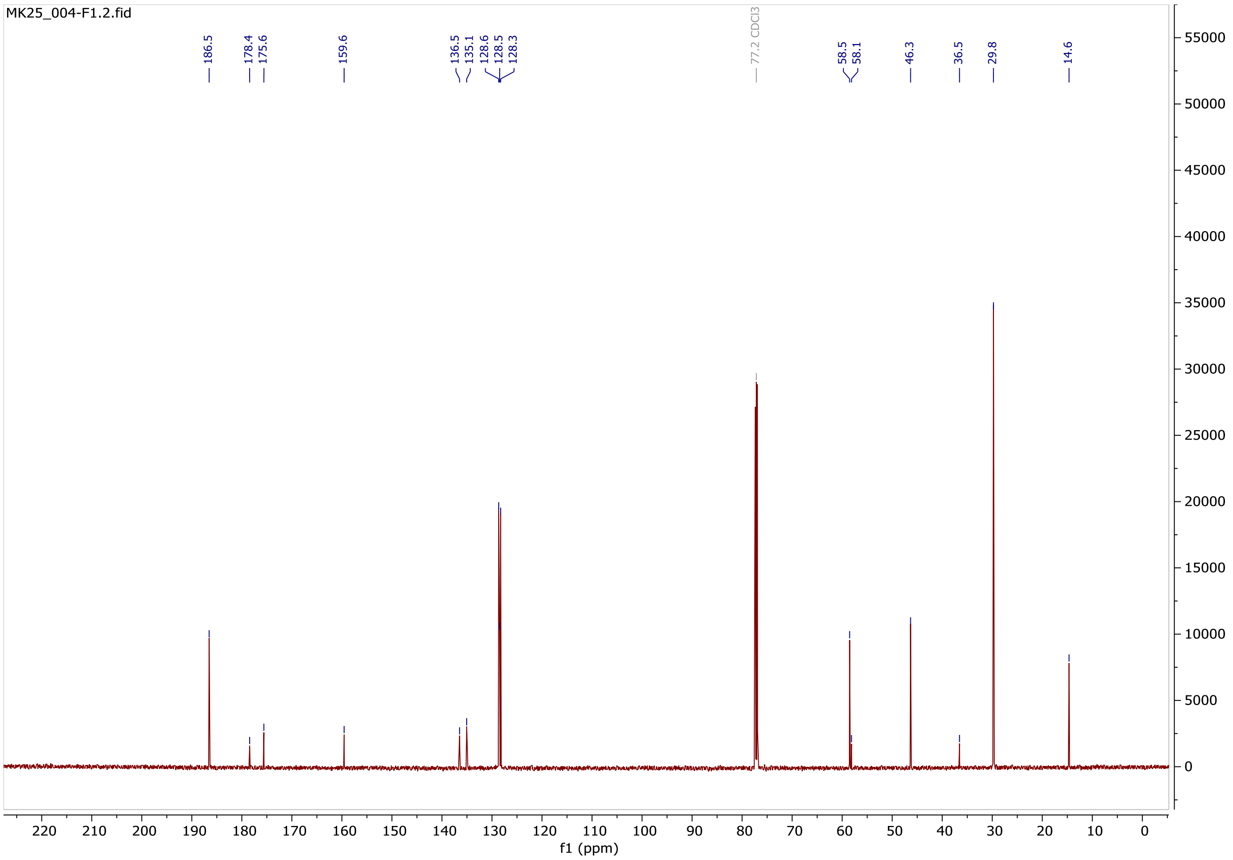

^{13}C {1H} NMR spectrum of **3r** ($CDCl_3$, 151 MHz).

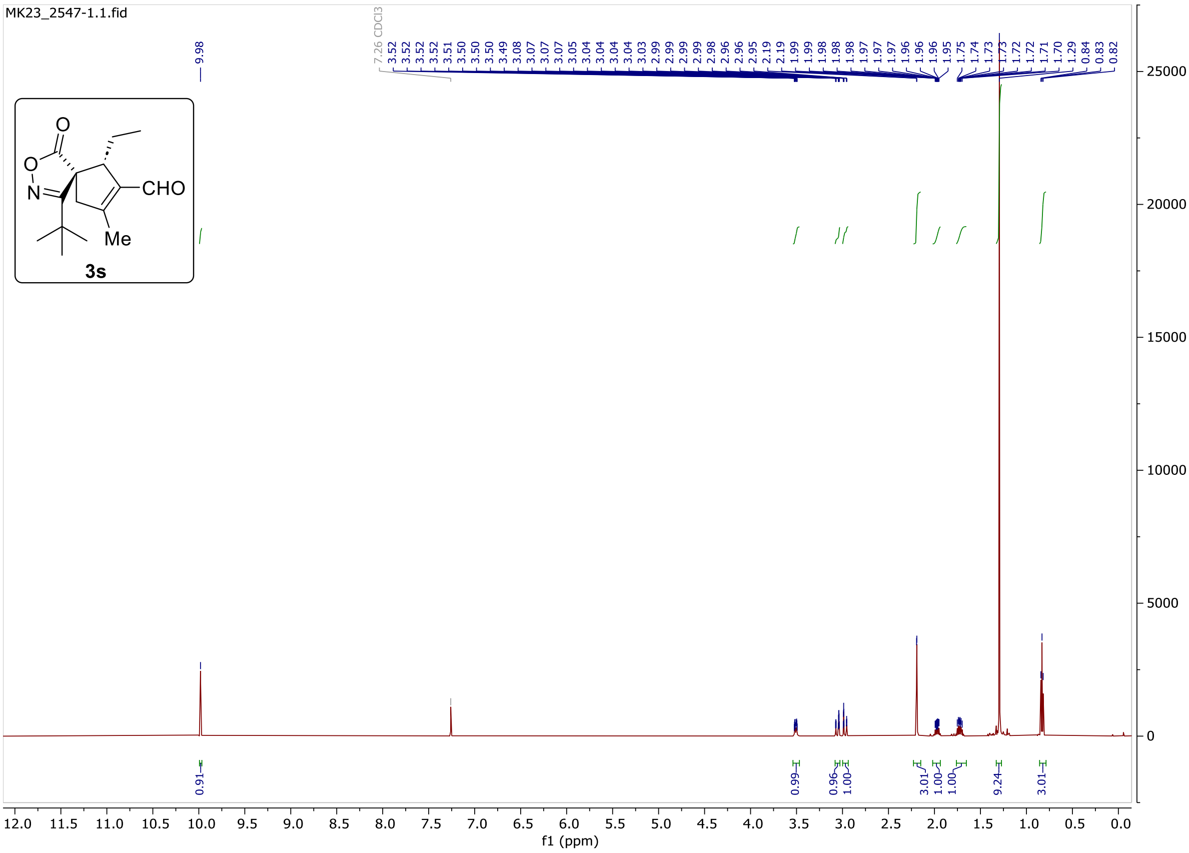

^{1}H NMR spectrum of **3s** ($CDCl_3$, 600 MHz).

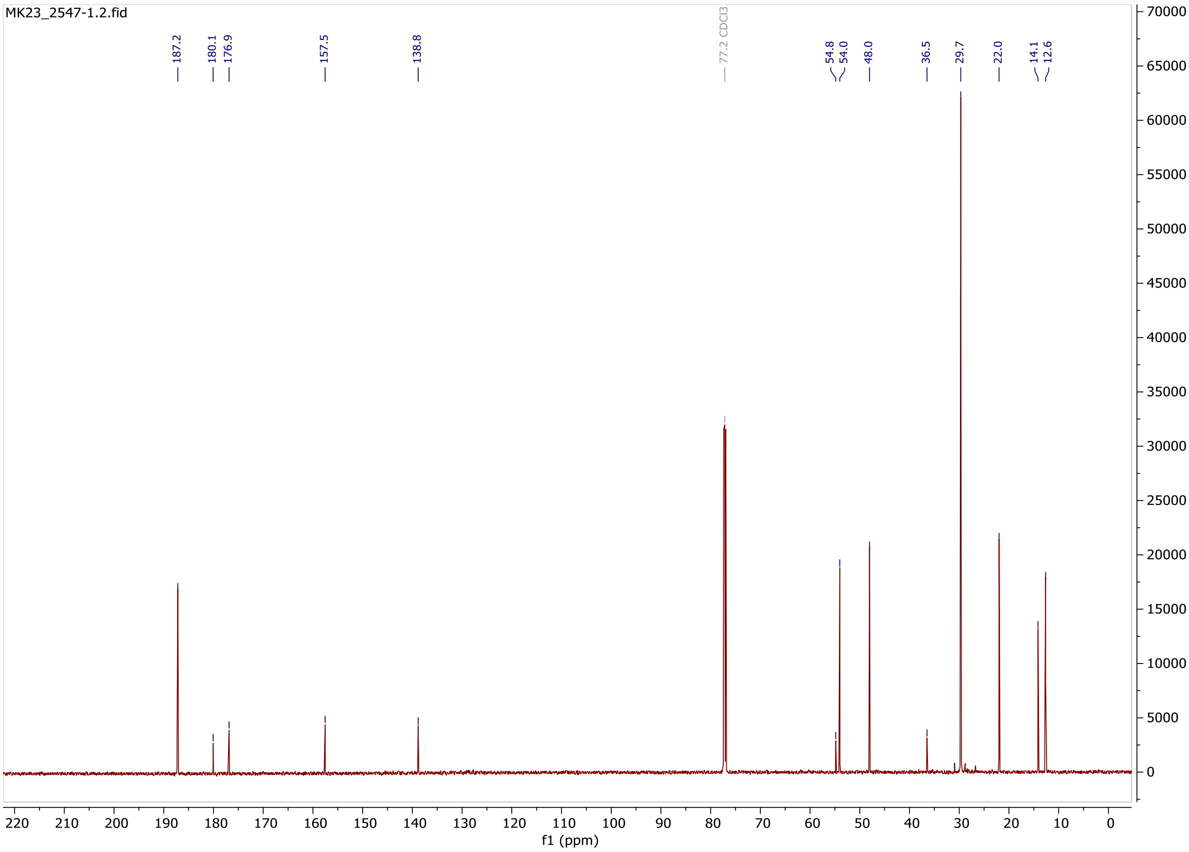

^{13}C {1H} NMR spectrum of **3s** ($CDCl_3$, 151 MHz).

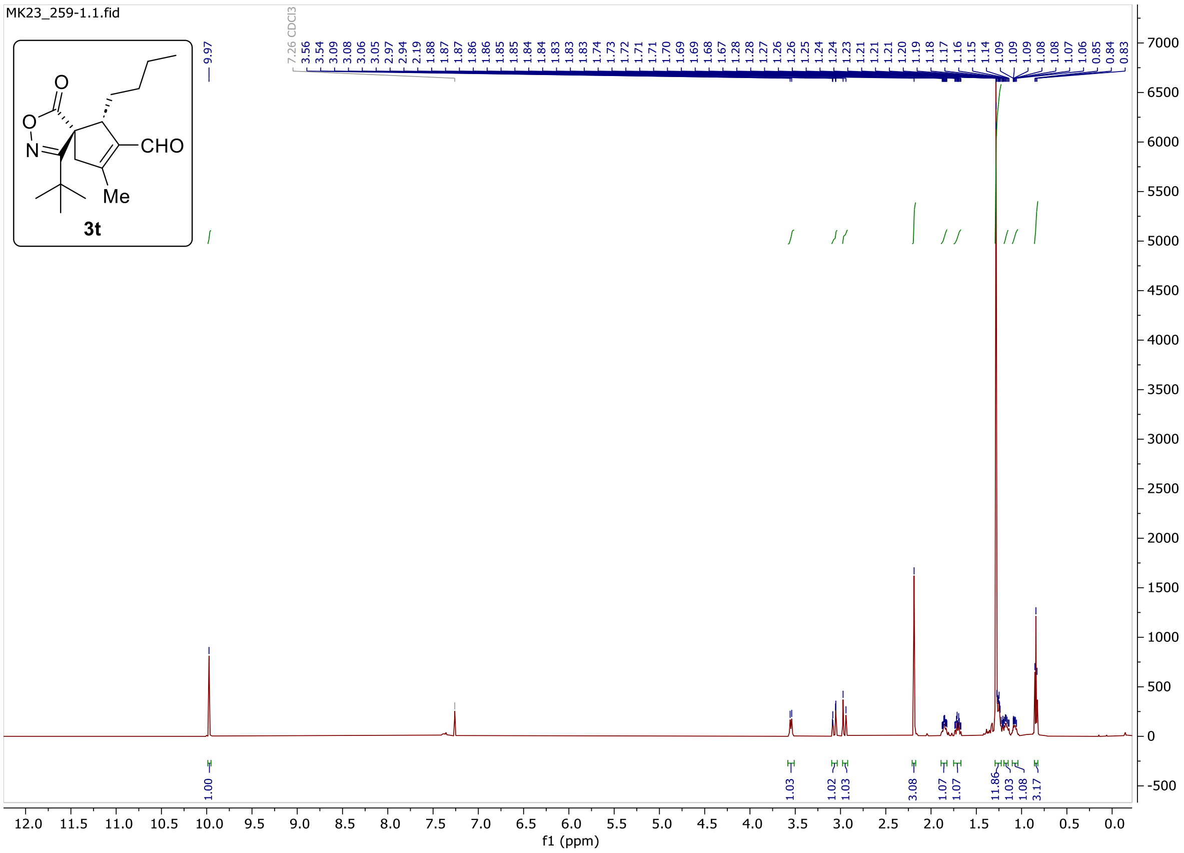

^{1}H NMR spectrum of **3t** ($CDCl_3$, 600 MHz).

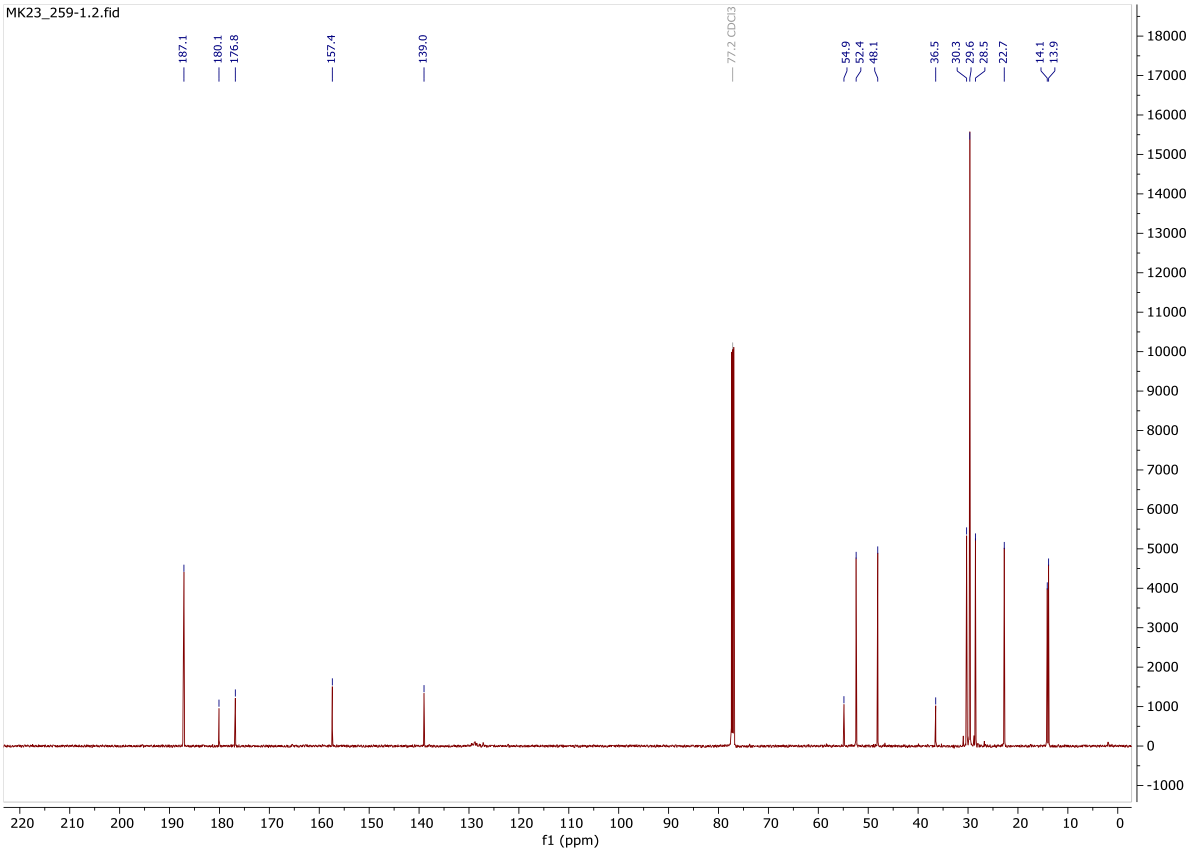

^{13}C {1H} NMR spectrum of **3t** ($CDCl_3$, 151 MHz).

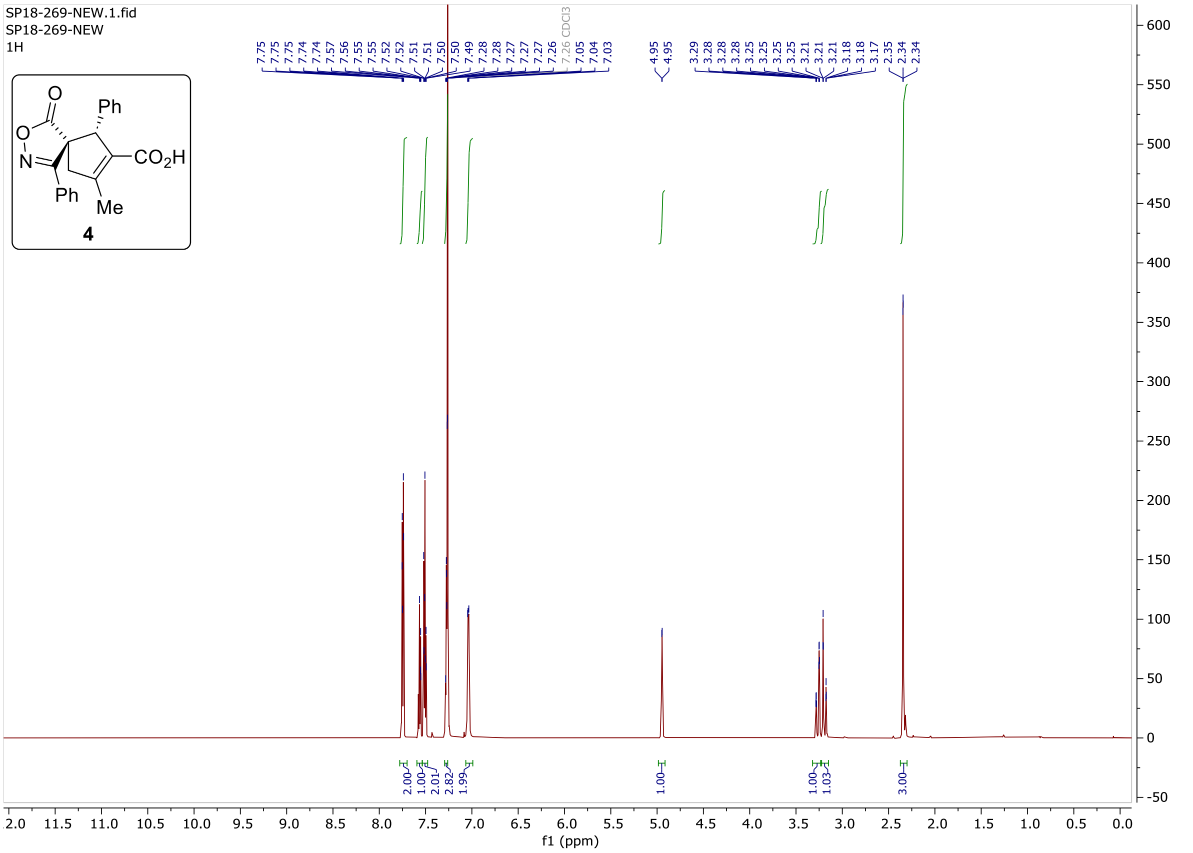

^{1}H NMR spectrum of **4** ($CDCl_3$, 600 MHz).

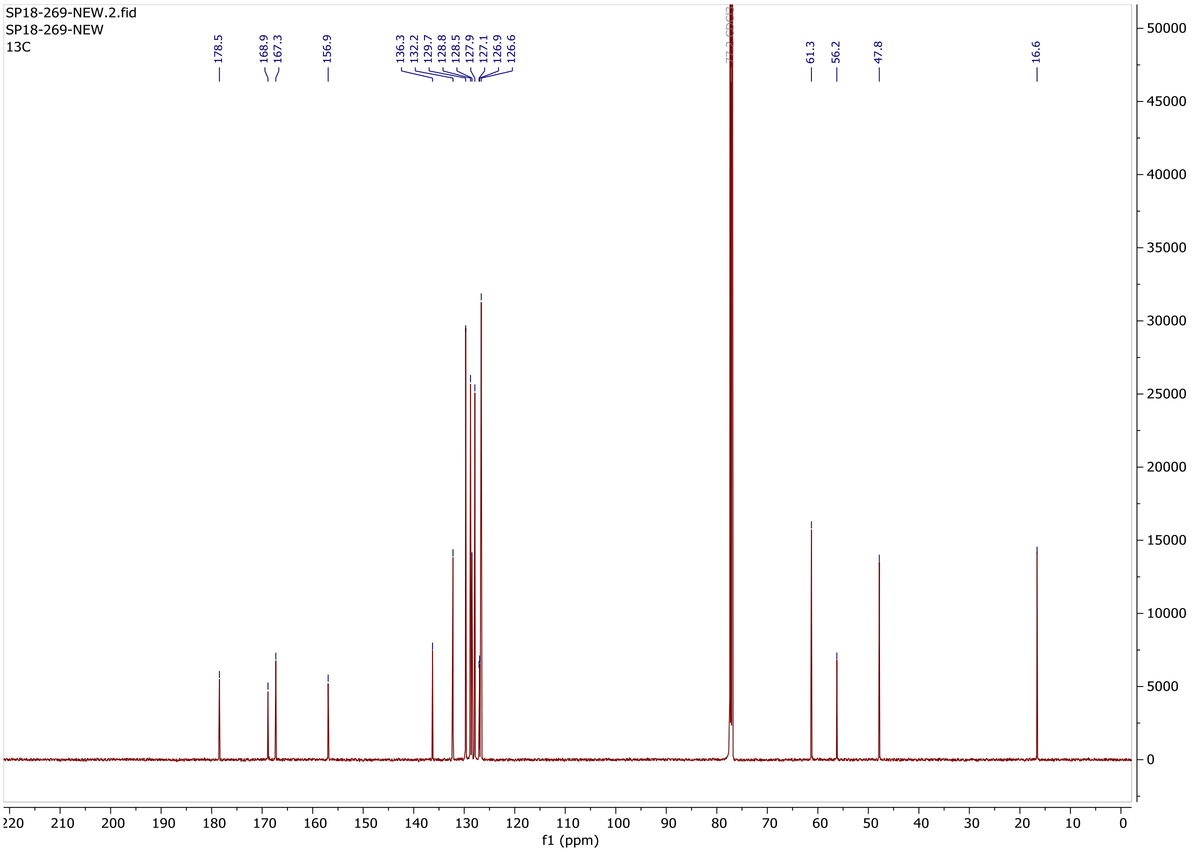

^{13}C {1H} NMR spectrum of **4** ($CDCl_3$, 151 MHz).

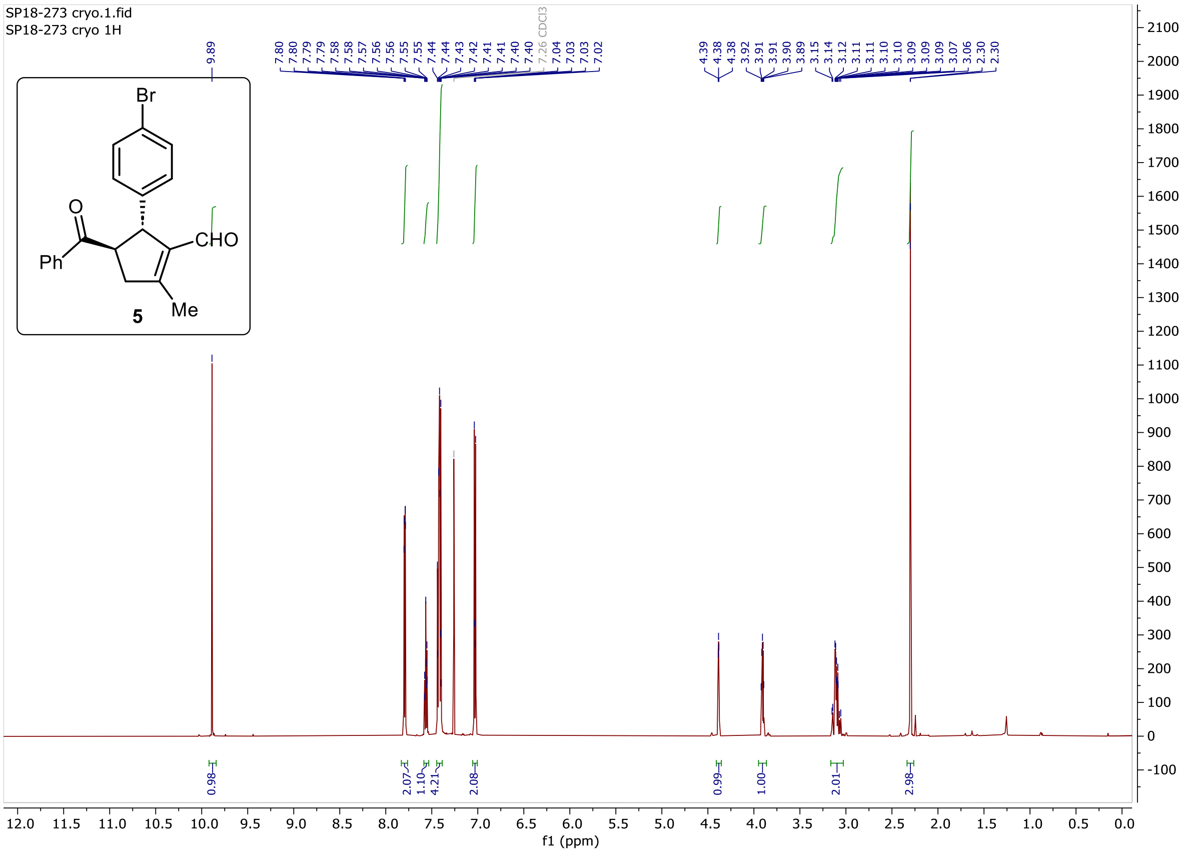

^{1}H NMR spectrum of **5** ($CDCl_3$, 600 MHz).

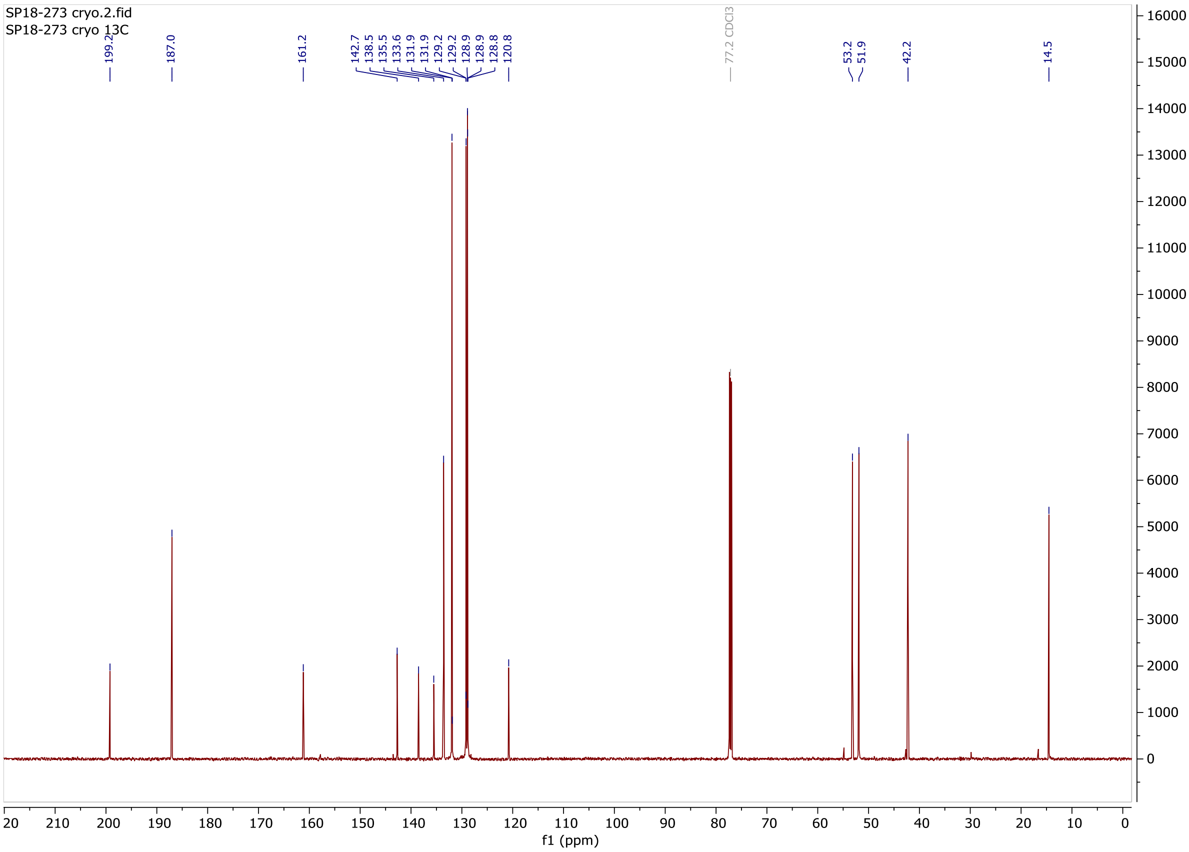

^{13}C {1H} NMR spectrum of **5** ($CDCl_3$, 151 MHz).

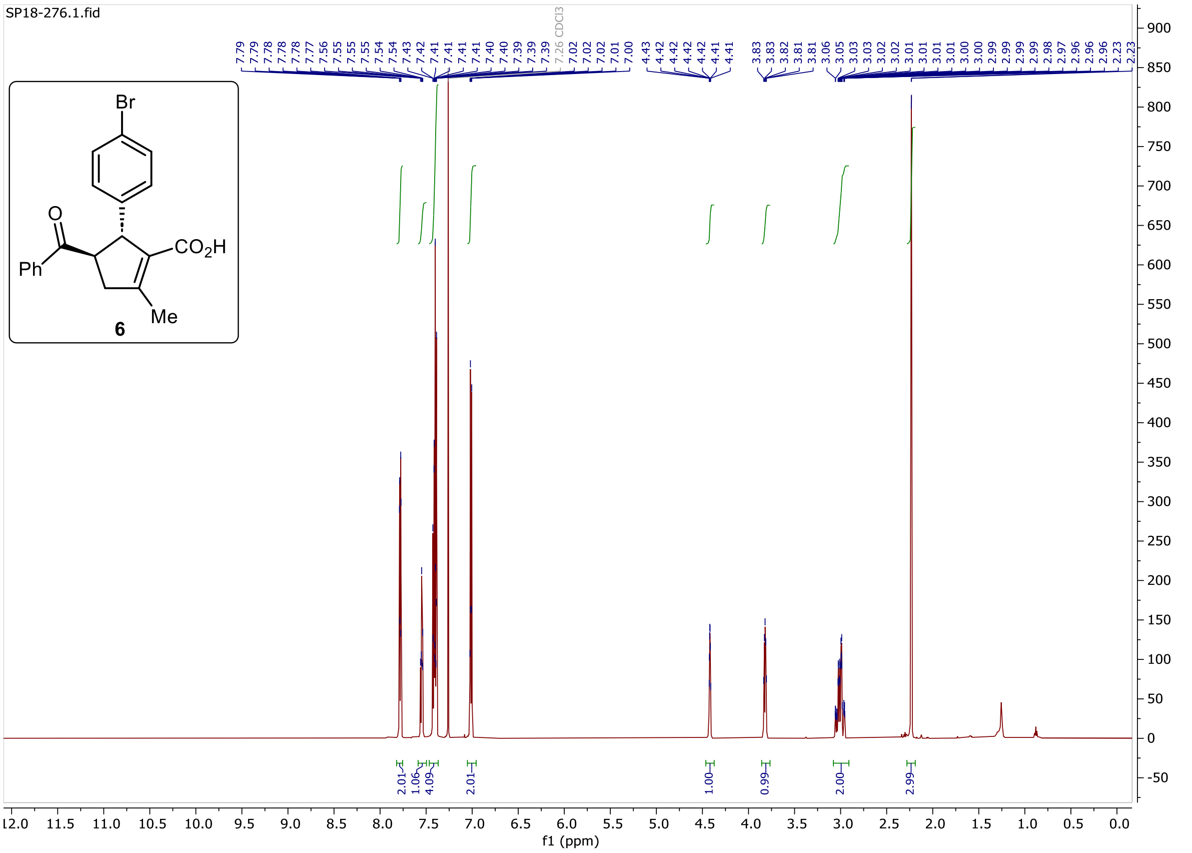

^{1}H NMR spectrum of **6** ($CDCl_3$, 600 MHz).

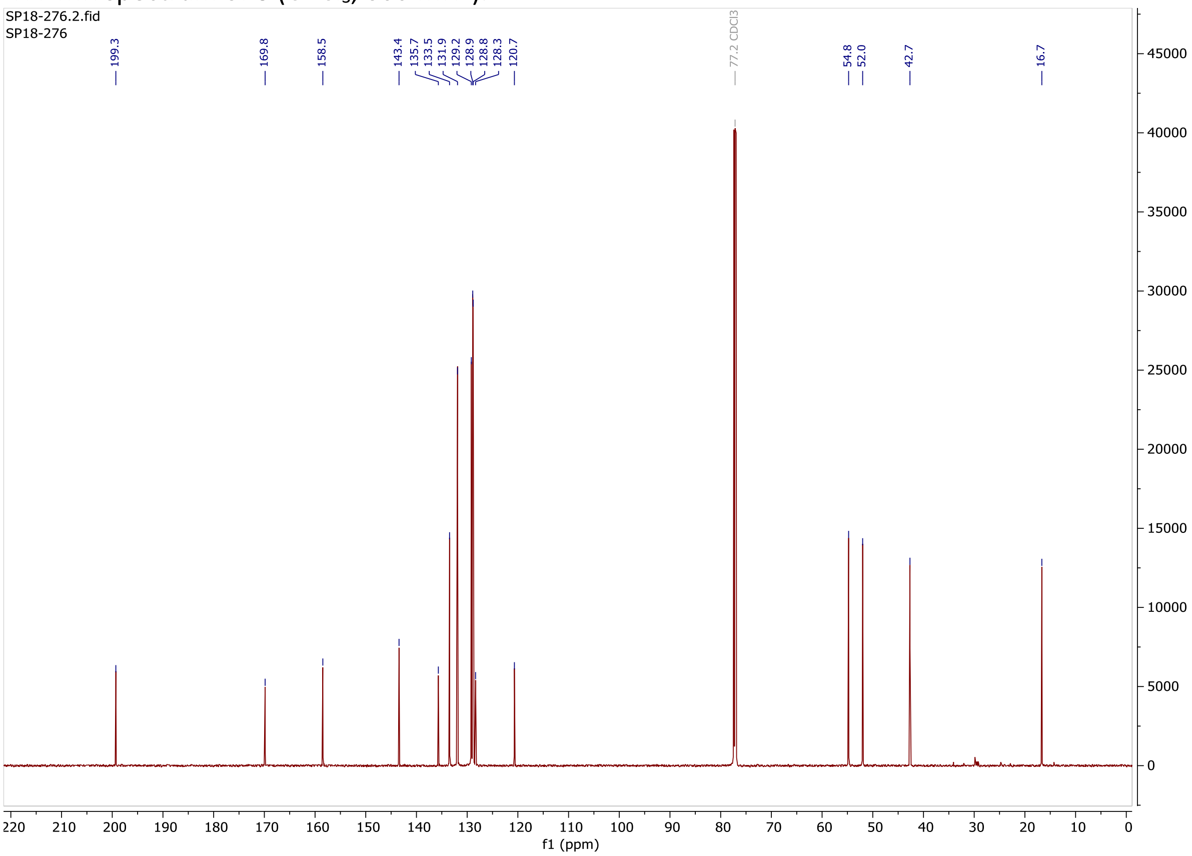

^{13}C {1H} NMR spectrum of **6** ($CDCl_3$, 151 MHz).

X-Ray section

The crystallographic data for samples **3e–major** (mk23_250_2), **3h–major** (mk23_247_2), **3r–major** (mk23_225_1) and **6** (sp18_276) were obtained from measurement on Bruker D8 VENTURE Kappa Duo diffractometer with PHOTON III detector, using x-ray source IμS micro-focus sealed tube either MoKα or CuKα radiation at a temperature 120(2) K. The structures were solved by direct methods (XT)[8] and refined by full matrix least squares based on F2 (SHELXL2018).[9] The hydrogen atoms on carbon were fixed into idealized positions (riding model) and assigned temperature factors either Hiso(H) = 1.2 Ueq (pivot atom) or Hiso(H) = 1.5 Ueq (pivot atom) for methyl moiety. The determination of absolute structure[10]40 of the crystals was based on anomalous scattering of Br (**6** sp18_276); and N,O for **3r–major** (mk23_225_1), **3h–major** (mk23_247_2).

X-ray crystallographic data have been deposited with the Cambridge Crystallographic Data Centre (CCDC) under deposition number **2363085** for **3e-major** (mk23_250-2), **2363084** for **3h-major** (mk23_247-2), **2363083** for **3r–major** (mk23_225_1) and **2363082** for **6** (sp18_276) and can be obtained free of charge from the Centre via its website (https://www.ccdc.cam.ac.uk/structures/?).

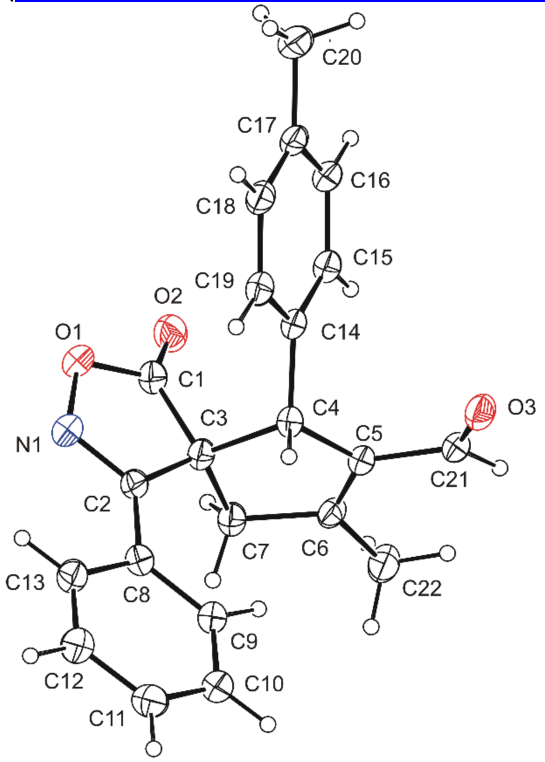

Fig. 1. View on the molecule of **3e – major** (mk23_250_2) with atom numbering scheme. The displacement ellipsoids are drawn of 30% probability level. The chirality descriptors for one molecule of racemic crystal are for C(3), C(4) : *S*, *R* respectively.

---

[8] SHELXT: G.M. Sheldrick, *Acta Cryst*. **2015**, *A71*, 3-8

[9] SHELXL: G.M. Sheldrick, *Acta Cryst*. **2015**, *C71*, 3-8.

[10] S. Parsons, H. D. Flack, T. Wagner, *Acta Cryst*. **2013**, *B69*, 249-259.

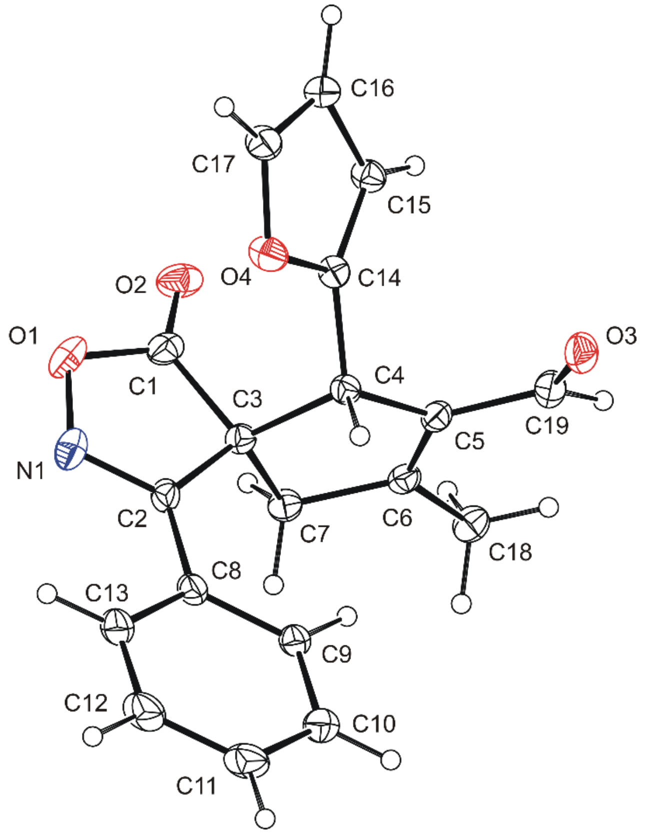

Fig. 2. View on the molecule of **3h – major** (mk23_247_2) with atom numbering scheme. The displacement ellipsoids are drawn of 30% probability level. The chirality descriptors for C(3), C(4) and are *S*, *R* respectively.

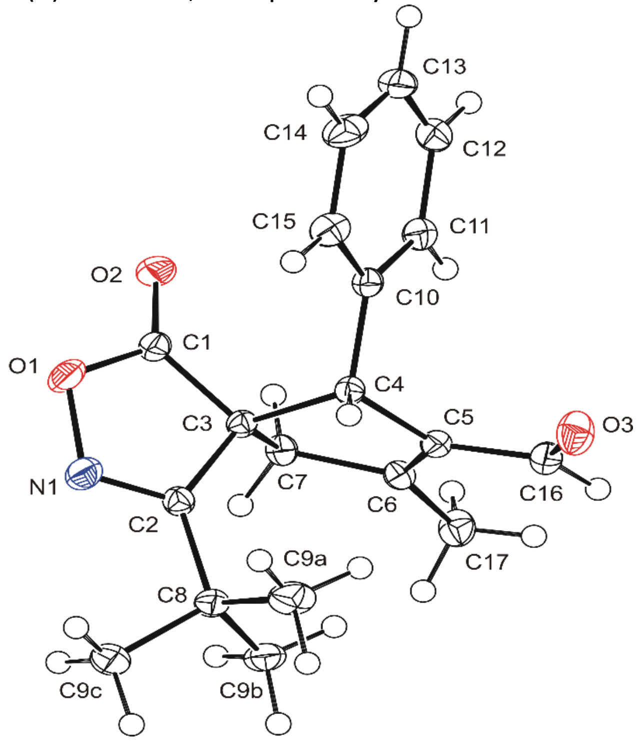

Fig. 3. View on the molecule of **3r–major** (mk23_225_1) with atom numbering scheme. The displacement ellipsoids are drawn of 30% probability level. The chirality descriptors for C(3), C(4) and are *S*, *R* respectively.

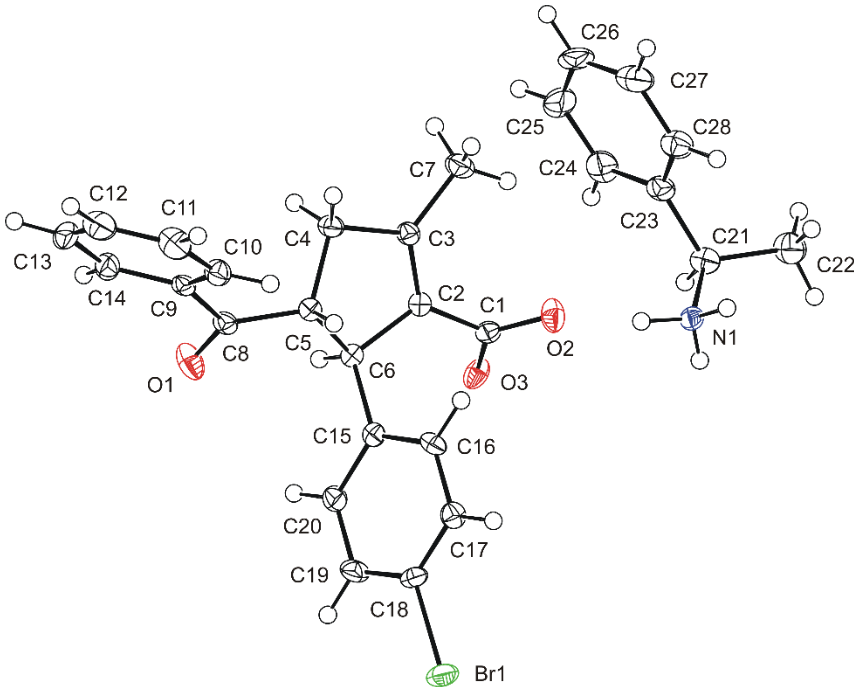

Fig. 4. View on the molecule of **6** (sp18_276) with atom numbering scheme. The displacement ellipsoids are drawn of 30% probability level. The chirality descriptors for C(5), C(6) and C(21) are *R*, *R*, *R* respectively.

Table S4. Crystal data, data collection, and refinement parameters for **6, 3r–major, 3h-major**

**Compound**	**6** **(sp18_276)**	**3r–major** **(mk23_225_1)**	**3h-major** **(mk23_247_2)**
CCDC	2363082	2363083	2363084
Formula	$C_{20}H_{16}BrO_3{\cdot}C_8H_{12}N$	$C_{19}H_{21}NO_3$	$C_{19}H_{15}NO_4$
M.w.	506.42	311.37	321.32
Crystal system	Orthorhombic	Monoclinic	Orthorhombic
Space group	$P2_12_12_1$ (No.19)	$P2_1$ (No. 4)	$P2_12_12_1$ (No.19)
a [Å]	6.1946 (3)	8.7310 (11)	7.3465 (2)
b [Å]	17.9333 (9)	7.1858 (9)	10.7682 (3)
c [Å]	22.1148 (11)	13.1687 (17)	19.3679 (5)
α [°]			
β [°]		91.407 (3)	
γ [°]			
Z	4	2	4
V[Å³]	2456.7 (2)	825.94 (18)	1532.17 (7)
Temperature	120	120	120
D_x [g cm^{-3}]	1.369	1.252	1.393
Wavelength, Å	0.71073	1.54178	1.54178
Crystal size [mm]	0.59 × 0.09 × 0.04	0.36 × 0.17 × 0.16	0.28 × 0.15 × 0.06
Crystal color, shape	Needle, colourless	Prism, colourless	Plate, brown
μ [mm^{-1}]	1.70	0.68	0.81
T_{min},T_{max}	0.77, 0.94	0.79, 0.90	0.79, 0.95
Measured reflections	19966	21538	23169
Independent diffractions (R_{int}^{a})	5605, (0.050)	3395, (0.025)	3238, (0.049)

Observed diffract. [I>2σ(I)]	4574	3384	3088
No. of parameters	303	212	218
R^b	0.036	0.027	0.038
$wR(F^2)$ for all data	0.071	0.071	0.103
GOF^c	1.02	1.07	1.07
Residual electron density [e/Å^3]	0.24, −0.40	0.20, −0.14	0.22, −0.14
Absolute structure parameter	-0.009(4)	-0.03(5)	-0.06(10)

$^a R_{int}$ = □□F_o^2−$F_{o,mean}^2$□/□F_o^2; $^b R(F)$ = □□□F_o□−□F_c□□/□□F_o□; $wR(F^2)$ = [□(w(F_o^2−F_c^2)^2)/(□w(F_o^2)^2)]^{½};
^{c}GOF = [□(w(F_o^2−F_c^2)^2)/(N_{diffrs}−N_{params})]^{½}

Table S5. Crystal data, data collection, and refinement parameters for **3e-major**

**Compound**	**3e-major** **(mk23_250_2)**
CCDC	2363085
Formula	$C_{22}H_{19}NO_3$
M.w.	345.38
Crystal system	Monoclinic
Space group	$P2_1/n$ (No.14)
a [Å]	16.2629 (9)
b [Å]	6.7976 (4)
c [Å]	17.0226 (8)
α [°]	
β [°]	112.103 (4)
γ [°]	
Z	4
V[Å^3]	1743.53 (17)
Temperature [K]	120
D_x [g cm^{-3}]	1.316
Wavelength [Å]	1.54178
Crystal size [mm]	0.11 × 0.11 × 0.04
Crystal color, shape	bar, colourless
μ [mm^{-1}]	0.71
T_{min},T_{max}	0.72, 0.98
Measured reflections	15627

Independent diffractions *(R_{int}[a])*	2956, (0.163)
Observed diffract. [I>2σ(I)]	1718
No. of parameters	237
R^b	0.063
$wR(F^2)$ for all data	0.165
GOF[c]	1.01
Residual electron density [e/Å^3]	0.18, −0.25

[a]R_{int} = □□F_o^2−$F_{o,mean}^2$□/□F_o^2; [b]$R(F)$ = □□□F_o□−□F_c□□/□□F_o□; $wR(F^2)$ = [□(w(F_o^2−F_c^2)^2)/(□w(F_o^2)^2)]^{½};
[c]GOF = [□(w(F_o^2−F_c^2)^2)/(N_{diffrs}−N_{params})]^{½}

Crystallization

Single crystal of product **3e-major** (mk23_250_2) was prepared by volatilization using a methanol as a solvent. Suitable crystals were selected and collected on a Bruker D8 VENTURE Kappa Duo PHOTONIII instrument by an IμS microfocus sealed tube with Mo Kα (λ = 0.71073) radiation at a low temperature of 120 K.

Refinement

Crystal data, data collection and structure refinement details are summarized in Table 1.

Results and discussion

Table 1
Experimental details

Crystal data	
Chemical formula	$C_{22}H_{19}NO_3$
M_r	345.38
Crystal system, space group	Monoclinic, $P2_1/n$
Temperature (K)	120
a, *b*, *c* (Å)	16.2629 (9), 6.7976 (4), 17.0226 (8)
□ (°)	112.103 (4)
V (Å^3)	1743.53 (17)
Z	4
Radiation type	Cu *K*□
□ (mm^{-1})	0.71

Crystal size (mm)	0.11 × 0.11 × 0.04
Data collection	
Diffractometer	Bruker D8 VENTURE Kappa Duo PHOTONIII CMOS
Absorption correction	Multi-scan<br>Krause, L., Herbst-Irmer, R., Sheldrick, G. M., Stalke, D. (2015). "Comparison of silver and molybdenum microfocus X-ray sources for single-crystal structure determination" J. Appl. Cryst. 48, 3-10.<br>doi:10.1107/S1600576714022985
T_{min}, T_{max}	0.72, 0.98
No. of measured, independent and observed [I > 2□□(I)] reflections	15627, 2956, 1718
R_{int}	0.163
(sin □/□)$_{max}$ (Å^{-1})	0.590
Refinement	
R[F^2 > 2□(F^2)], wR(F^2), S	0.063, 0.165, 1.01
No. of reflections	2956
No. of parameters	237
H-atom treatment	H-atom parameters constrained
□□$_{max}$, □□$_{min}$ (e Å^{-3})	0.18, -0.25

Computer programs: Bruker Instrument Service v8.5.1.2 Release, *SAINT* V8.40B (Bruker AXS LLC, 2019), SHELXT 2018/2 (Sheldrick, 2018), *SHELXL2019*/2 (Sheldrick, 2019).

References

NOT FOUND

Document origin: *publCIF* [Westrip, S. P. (2010). *J. Apply. Cryst.*, **43**, 920-925].

Computing details

Data collection: Bruker Instrument Service v8.5.1.2 Release; cell refinement: *SAINT* V8.40B (Bruker AXS LLC, 2019); data reduction: *SAINT* V8.40B (Bruker AXS LLC, 2019); program(s) used to solve structure: SHELXT 2018/2 (Sheldrick, 2018); program(s) used to refine structure: *SHELXL2019*/2 (Sheldrick, 2019).

(cu_mk23_250_2_vesely)

Crystal data

$C_{22}H_{19}NO_3$	F(000) = 728
M_r = 345.38	D_x = 1.316 Mg m^{-3}
Monoclinic, $P2_1/n$	Cu K□ radiation, □ = 1.54178 Å

a = 16.2629 (9) Å	Cell parameters from 3614 reflections
b = 6.7976 (4) Å	[illegible] = 3.2–65.1°
c = 17.0226 (8) Å	[illegible] = 0.71 mm^{-1}
[illegible] = 112.103 (4)°	*T* = 120 K
V = 1743.53 (17) $Å^3$	Bar, colourless
Z = 4	0.11 × 0.11 × 0.04 mm

Data collection

Bruker D8 VENTURE Kappa Duo PHOTONIII CMOS<br>diffractometer	2956 independent reflections
Radiation source: I[illegible]S micro-focus sealed tube	1718 reflections with $I > 2$[illegible](I)
Helios Cu multilayer optic monochromator	R_{int} = 0.163
[illegible] and [illegible] scans	[illegible]$_{max}$ = 65.4°, [illegible]$_{min}$ = 3.2°
Absorption correction: multi-scan<br>Krause, L., Herbst-Irmer, R., Sheldrick, G. M., Stalke, D. (2015). "Comparison of silver and molybdenum microfocus X-ray sources for single-crystal structure determination" J. Appl. Cryst. 48, 3-10.<br>doi:10.1107/S1600576714022985	*h* = -19[illegible]19
T_{min} = 0.72, T_{max} = 0.98	*k* = -7[illegible]7
15627 measured reflections	*l* = -20[illegible]20

Refinement

Refinement on F^2	Primary atom site location: structure-invariant direct methods
Least-squares matrix: full	Secondary atom site location: difference Fourier map
$R[F^2 > 2$[illegible]$(F^2)]$ = 0.063	Hydrogen site location: mixed
$wR(F^2)$ = 0.165	H-atom parameters constrained
S = 1.01	$w = 1/[$[illegible]$^2(F_o^2) + (0.0651P)^2]$<br>where $P = (F_o^2 + 2F_c^2)/3$
2956 reflections	([illegible]/[illegible])$_{max}$ < 0.001
237 parameters	[illegible]$_{max}$ = 0.18 e $Å^{-3}$
0 restraints	[illegible]$_{min}$ = -0.25 e $Å^{-3}$

Special details

Geometry. All esds (except the esd in the dihedral angle between two l.s. planes) are estimated using the full covariance matrix. The cell esds are taken into account individually in the estimation of esds in distances, angles and torsion angles; correlations between esds in cell parameters are only used when they are defined by crystal symmetry. An approximate (isotropic) treatment of cell esds is used for estimating esds involving l.s. planes.

Fractional atomic coordinates and isotropic or equivalent isotropic displacement parameters (Å^2) for (cu_mk23_250_2_vesely)

	x	*y*	*z*	U_{iso}*/U_{eq}
C2	0.5192 (2)	0.3377 (5)	0.4112 (2)	0.0390 (8)
O3	0.70907 (18)	0.3470 (4)	0.21502 (14)	0.0531 (7)
C3	0.5766 (2)	0.4884 (5)	0.3928 (2)	0.0393 (8)
C1	0.6347 (3)	0.5493 (5)	0.4816 (2)	0.0433 (9)
N1	0.5448 (2)	0.2974 (5)	0.49160 (17)	0.0470 (8)
O1	0.61882 (17)	0.4230 (4)	0.53742 (14)	0.0482 (7)
O2	0.68751 (18)	0.6796 (4)	0.50600 (15)	0.0533 (7)
C4	0.6344 (2)	0.3864 (5)	0.3452 (2)	0.0381 (8)
H4	0.596453	0.260178	0.315525	0.046*
C5	0.6292 (2)	0.5373 (5)	0.27844 (19)	0.0377 (8)
C6	0.5749 (2)	0.6871 (5)	0.27383 (19)	0.0386 (8)
C7	0.5332 (3)	0.6707 (5)	0.3392 (2)	0.0442 (9)
H7A	0.468148	0.653054	0.311416	0.053*
H7B	0.545272	0.790179	0.374991	0.053*
C8	0.4451 (2)	0.2279 (5)	0.3506 (2)	0.0399 (8)
C9	0.4174 (2)	0.2572 (5)	0.2631 (2)	0.0441 (9)
H9	0.445669	0.354795	0.242155	0.053*
C10	0.3499 (3)	0.1467 (6)	0.2071 (2)	0.0491 (10)
H10	0.332625	0.166620	0.147776	0.059*
C11	0.3072 (3)	0.0072 (6)	0.2368 (2)	0.0520 (10)
H11	0.260392	-0.068682	0.198072	0.062*
C12	0.3328 (3)	-0.0219 (6)	0.3231 (2)	0.0524 (10)
H12	0.302960	-0.116928	0.343620	0.063*
C13	0.4008 (3)	0.0857 (5)	0.3791 (2)	0.0463 (9)
H13	0.418192	0.063219	0.438189	0.056*
C14	0.7258 (2)	0.3265 (5)	0.40361 (19)	0.0363 (8)
C15	0.7970 (2)	0.4543 (5)	0.4258 (2)	0.0414 (9)
H15	0.788822	0.582724	0.402105	0.050*
C16	0.8798 (3)	0.3980 (6)	0.4818 (2)	0.0442 (9)
H16	0.927510	0.489020	0.496247	0.053*
C17	0.8950 (3)	0.2110 (6)	0.5178 (2)	0.0441 (9)
C18	0.8242 (3)	0.0820 (5)	0.4958 (2)	0.0447 (9)
H18	0.832962	-0.046617	0.519403	0.054*
C19	0.7401 (3)	0.1377 (5)	0.4396 (2)	0.0434 (9)
H19	0.692149	0.047224	0.425551	0.052*
C20	0.9861 (3)	0.1541 (6)	0.5782 (2)	0.0581 (11)

H20A	0.984981	0.018441	0.597278	0.087*
H20B	1.004571	0.242550	0.627312	0.087*
H20C	1.028088	0.164236	0.549586	0.087*
C21	0.6732 (2)	0.5003 (6)	0.2180 (2)	0.0408 (8)
H21	0.673065	0.602129	0.179755	0.049*
C22	0.5519 (3)	0.8571 (5)	0.2139 (2)	0.0524 (10)
H22A	0.569370	0.980048	0.245944	0.079*
H22B	0.487779	0.858232	0.181172	0.079*
H22C	0.583455	0.844684	0.175107	0.079*

Atomic displacement parameters (Å^2) for (cu_mk23_250_2_vesely)

	U^{11}	U^{22}	U^{33}	U^{12}	U^{13}	U^{23}
C2	0.036 (2)	0.0506 (19)	0.0329 (18)	0.0107 (17)	0.0160 (16)	0.0040 (15)
O3	0.0582 (18)	0.0691 (17)	0.0355 (13)	0.0150 (15)	0.0216 (13)	0.0017 (12)
C3	0.041 (2)	0.0492 (19)	0.0308 (17)	0.0055 (17)	0.0170 (16)	0.0018 (15)
C1	0.043 (2)	0.054 (2)	0.0369 (19)	0.0047 (19)	0.0198 (17)	0.0008 (17)
N1	0.0460 (19)	0.0612 (18)	0.0328 (16)	0.0006 (16)	0.0136 (14)	0.0025 (14)
O1	0.0471 (16)	0.0646 (16)	0.0302 (12)	0.0014 (13)	0.0112 (12)	0.0020 (11)
O2	0.0521 (18)	0.0648 (16)	0.0464 (15)	-0.0065 (15)	0.0224 (13)	-0.0127 (13)
C4	0.039 (2)	0.0475 (19)	0.0311 (17)	0.0013 (16)	0.0172 (16)	-0.0014 (15)
C5	0.036 (2)	0.0500 (19)	0.0275 (16)	0.0018 (17)	0.0129 (15)	-0.0006 (15)
C6	0.040 (2)	0.0488 (19)	0.0251 (16)	-0.0050 (17)	0.0104 (15)	-0.0017 (14)
C7	0.046 (2)	0.054 (2)	0.0370 (18)	0.0057 (18)	0.0211 (18)	0.0026 (16)
C8	0.037 (2)	0.0488 (19)	0.0372 (18)	0.0033 (17)	0.0174 (16)	0.0007 (16)
C9	0.040 (2)	0.059 (2)	0.0356 (18)	0.0005 (18)	0.0166 (17)	0.0016 (17)
C10	0.042 (2)	0.066 (2)	0.038 (2)	0.004 (2)	0.0150 (19)	0.0011 (18)
C11	0.043 (2)	0.059 (2)	0.052 (2)	0.001 (2)	0.0149 (19)	-0.0011 (19)
C12	0.051 (3)	0.060 (2)	0.047 (2)	0.000 (2)	0.020 (2)	0.0071 (19)
C13	0.047 (2)	0.053 (2)	0.040 (2)	0.0062 (19)	0.0191 (19)	0.0071 (17)
C14	0.038 (2)	0.0470 (19)	0.0255 (16)	0.0013 (17)	0.0143 (15)	0.0000 (14)
C15	0.044 (2)	0.051 (2)	0.0315 (18)	0.0027 (18)	0.0168 (17)	0.0007 (15)
C16	0.041 (2)	0.062 (2)	0.0283 (17)	0.0020 (18)	0.0113 (17)	-0.0022 (16)
C17	0.043 (2)	0.063 (2)	0.0257 (17)	0.010 (2)	0.0130 (16)	0.0000 (16)
C18	0.051 (3)	0.052 (2)	0.0335 (18)	0.0118 (19)	0.0189 (18)	0.0047 (16)
C19	0.047 (2)	0.054 (2)	0.0337 (18)	-0.0010 (18)	0.0193 (18)	-0.0025 (16)
C20	0.051 (3)	0.082 (3)	0.037 (2)	0.012 (2)	0.0112 (19)	-0.0011 (19)
C21	0.038 (2)	0.056 (2)	0.0275 (17)	-0.0010 (19)	0.0119 (15)	-0.0034 (16)
C22	0.064 (3)	0.055 (2)	0.042 (2)	0.011 (2)	0.025 (2)	0.0064 (17)

Geometric parameters (Å, º) for (cu_mk23_250_2_vesely)

C2—N1	1.301 (4)	C10—H10	0.9500
C2—C8	1.462 (5)	C11—C12	1.382 (5)
C2—C3	1.495 (5)	C11—H11	0.9500
O3—C21	1.205 (4)	C12—C13	1.369 (5)
C3—C1	1.507 (5)	C12—H12	0.9500
C3—C7	1.544 (5)	C13—H13	0.9500
C3—C4	1.611 (4)	C14—C15	1.382 (5)
C1—O2	1.194 (4)	C14—C19	1.403 (5)
C1—O1	1.375 (4)	C15—C16	1.379 (5)
N1—O1	1.442 (4)	C15—H15	0.9500
C4—C14	1.501 (5)	C16—C17	1.392 (5)
C4—C5	1.510 (4)	C16—H16	0.9500
C4—H4	1.0662	C17—C18	1.383 (5)
C5—C6	1.331 (5)	C17—C20	1.501 (5)
C5—C21	1.478 (4)	C18—C19	1.395 (5)
C6—C22	1.493 (5)	C18—H18	0.9500
C6—C7	1.509 (4)	C19—H19	0.9500
C7—H7A	0.9900	C20—H20A	0.9800
C7—H7B	0.9900	C20—H20B	0.9800
C8—C13	1.396 (5)	C20—H20C	0.9800
C8—C9	1.398 (5)	C21—H21	0.9500
C9—C10	1.376 (5)	C22—H22A	0.9800
C9—H9	0.9500	C22—H22B	0.9800
C10—C11	1.379 (5)	C22—H22C	0.9800
N1—C2—C8	119.2 (3)	C10—C11—H11	120.1
N1—C2—C3	112.7 (3)	C12—C11—H11	120.1
C8—C2—C3	128.0 (3)	C13—C12—C11	120.3 (4)
C2—C3—C1	100.6 (3)	C13—C12—H12	119.8
C2—C3—C7	119.4 (3)	C11—C12—H12	119.8
C1—C3—C7	110.3 (3)	C12—C13—C8	121.0 (3)
C2—C3—C4	109.8 (3)	C12—C13—H13	119.5
C1—C3—C4	111.0 (3)	C8—C13—H13	119.5
C7—C3—C4	105.7 (2)	C15—C14—C19	118.1 (3)
O2—C1—O1	121.3 (3)	C15—C14—C4	122.0 (3)
O2—C1—C3	130.6 (3)	C19—C14—C4	119.9 (3)
O1—C1—C3	108.1 (3)	C16—C15—C14	121.0 (3)

C2—N1—O1	108.4 (3)	C16—C15—H15	119.5
C1—O1—N1	109.4 (3)	C14—C15—H15	119.5
C14—C4—C5	116.3 (3)	C15—C16—C17	121.6 (4)
C14—C4—C3	113.8 (2)	C15—C16—H16	119.2
C5—C4—C3	102.1 (3)	C17—C16—H16	119.2
C14—C4—H4	110.3	C18—C17—C16	117.9 (3)
C5—C4—H4	109.4	C18—C17—C20	121.9 (3)
C3—C4—H4	104.0	C16—C17—C20	120.2 (4)
C6—C5—C21	125.0 (3)	C17—C18—C19	121.0 (3)
C6—C5—C4	114.3 (3)	C17—C18—H18	119.5
C21—C5—C4	120.3 (3)	C19—C18—H18	119.5
C5—C6—C22	128.5 (3)	C18—C19—C14	120.4 (4)
C5—C6—C7	111.9 (3)	C18—C19—H19	119.8
C22—C6—C7	119.6 (3)	C14—C19—H19	119.8
C6—C7—C3	105.3 (3)	C17—C20—H20A	109.5
C6—C7—H7A	110.7	C17—C20—H20B	109.5
C3—C7—H7A	110.7	H20A—C20—H20B	109.5
C6—C7—H7B	110.7	C17—C20—H20C	109.5
C3—C7—H7B	110.7	H20A—C20—H20C	109.5
H7A—C7—H7B	108.8	H20B—C20—H20C	109.5
C13—C8—C9	117.8 (3)	O3—C21—C5	123.0 (3)
C13—C8—C2	120.3 (3)	O3—C21—H21	118.5
C9—C8—C2	121.8 (3)	C5—C21—H21	118.5
C10—C9—C8	120.9 (3)	C6—C22—H22A	109.5
C10—C9—H9	119.5	C6—C22—H22B	109.5
C8—C9—H9	119.5	H22A—C22—H22B	109.5
C9—C10—C11	120.1 (3)	C6—C22—H22C	109.5
C9—C10—H10	120.0	H22A—C22—H22C	109.5
C11—C10—H10	120.0	H22B—C22—H22C	109.5
C10—C11—C12	119.8 (4)		

Document origin: *publCIF* [Westrip, S. P. (2010). *J. Apply. Cryst.*, **43**, 920-925].

Crystallization

Single crystal of product **3h-major** (mk23_247_2) was prepared by volatilization using a methanol as a solvent. Suitable crystals were selected and collected on a Bruker D8 VENTURE Kappa Duo PHOTONIII instrument by an IμS microfocus sealed tube with Mo Kα (λ = 0.71073) radiation at a low temperature of 120 K.

Refinement

Crystal data, data collection and structure refinement details are summarized in Table 1.

Results and discussion

Table 1
Experimental details

Crystal data	
Chemical formula	$C_{19}H_{15}NO_4$
M_r	321.32
Crystal system, space group	Orthorhombic, $P2_12_12_1$
Temperature (K)	120
a, *b*, *c* (Å)	7.3465 (2), 10.7682 (3), 19.3679 (5)
V (Å^3)	1532.17 (7)
Z	4
Radiation type	Cu *K*[illegible]
[illegible] (mm^{-1})	0.81
Crystal size (mm)	0.28 × 0.15 × 0.06
Data collection	
Diffractometer	Bruker D8 VENTURE Kappa Duo PHOTONIII CMOS
Absorption correction	Multi-scan<br>Krause, L., Herbst-Irmer, R., Sheldrick, G. M., Stalke, D. (2015). "Comparison of silver and molybdenum microfocus X-ray sources for single-crystal structure determination" J. Appl. Cryst. 48, 3-10.<br>doi:10.1107/S1600576714022985
T_{min}, T_{max}	0.79, 0.95
No. of measured, independent and observed [I > 2[illegible](I)] reflections	23169, 3238, 3088
R_{int}	0.049
(sin [illegible]/[illegible])$_{max}$ (Å^{-1})	0.633
Refinement	
R[F^2 > 2[illegible](F^2)], wR(F^2), S	0.038, 0.103, 1.07
No. of reflections	3238

No. of parameters	218
H-atom treatment	H-atom parameters constrained
[illegible]$_{max}$, [illegible]$_{min}$ (e Å^{-3})	0.22, -0.14
Absolute structure	Flack x determined using 1238 quotients [(I+)-(I-)]/[(I+)+(I-)] (Parsons, Flack and Wagner, Acta Cryst. B69 (2013) 249-259).
Absolute structure parameter	-0.06 (10)

Computer programs: Bruker Instrument Service v8.5.1.2 Release, *SAINT* V8.40B (Bruker AXS LLC, 2019), SHELXT 2018/2 (Sheldrick, 2018), *SHELXL2019*/2 (Sheldrick, 2019).

References

NOT FOUND

Document origin: *publCIF* [Westrip, S. P. (2010). *J. Apply. Cryst.*, **43**, 920-925].

Computing details

Data collection: Bruker Instrument Service v8.5.1.2 Release; cell refinement: *SAINT* V8.40B (Bruker AXS LLC, 2019); data reduction: *SAINT* V8.40B (Bruker AXS LLC, 2019); program(s) used to solve structure: SHELXT 2018/2 (Sheldrick, 2018); program(s) used to refine structure: *SHELXL2019*/2 (Sheldrick, 2019).

(cu_mk23_247_2_vesely)

Crystal data

$C_{19}H_{15}NO_4$	D_x = 1.393 Mg m^{-3}
M_r = 321.32	Cu *K*[illegible] radiation, [illegible] = 1.54178 Å
Orthorhombic, $P2_12_12_1$	Cell parameters from 9934 reflections
a = 7.3465 (2) Å	[illegible] = 4.6–77.0°
b = 10.7682 (3) Å	[illegible] = 0.81 mm^{-1}
c = 19.3679 (5) Å	*T* = 120 K
V = 1532.17 (7) Å^3	Plate, brown
Z = 4	0.28 × 0.15 × 0.06 mm
F(000) = 672	

Data collection

Bruker D8 VENTURE Kappa Duo PHOTONIII CMOS<br>diffractometer	3238 independent reflections
Radiation source: I[illegible]S micro-focus sealed tube	3088 reflections with *I* > 2[illegible](*I*)
Helios Cu multilayer optic monochromator	R_{int} = 0.049
[illegible] and [illegible] scans	[illegible]$_{max}$ = 77.4°, [illegible]$_{min}$ = 4.6°
Absorption correction: multi-scan<br>Krause, L., Herbst-Irmer, R., Sheldrick, G. M., Stalke, D. (2015). "Comparison of silver and molybdenum microfocus X-ray sources for single-crystal structure determination" J. Appl. Cryst. 48, 3-10.<br>doi:10.1107/S1600576714022985	*h* = -9[illegible]9
T_{min} = 0.79, T_{max} = 0.95	*k* = -13[illegible]13
23169 measured reflections	*l* = -22[illegible]24

Refinement

Refinement on F^2	Secondary atom site location: difference Fourier map
Least-squares matrix: full	Hydrogen site location: mixed
$R[F^2 > 2$[illegible]$(F^2)]$ = 0.038	H-atom parameters constrained
$wR(F^2)$ = 0.103	$w = 1/[$[illegible]$^2(F_o^2) + (0.0616P)^2 + 0.2868P]$<br>where $P = (F_o^2 + 2F_c^2)/3$
S = 1.07	([illegible]/[illegible])$_{max}$ < 0.001
3238 reflections	[illegible]$_{max}$ = 0.22 e Å^{-3}

218 parameters	[illegible]$_{min}$ = -0.14 e Å^{-3}
0 restraints	Absolute structure: Flack x determined using 1238 quotients [(I+)-(I-)]/[(I+)+(I-)] (Parsons, Flack and Wagner, Acta Cryst. B69 (2013) 249-259).
Primary atom site location: structure-invariant direct methods	Absolute structure parameter: -0.06 (10)

Special details

Geometry. All esds (except the esd in the dihedral angle between two l.s. planes) are estimated using the full covariance matrix. The cell esds are taken into account individually in the estimation of esds in distances, angles and torsion angles; correlations between esds in cell parameters are only used when they are defined by crystal symmetry. An approximate (isotropic) treatment of cell esds is used for estimating esds involving l.s. planes.

Fractional atomic coordinates and isotropic or equivalent isotropic displacement parameters (Å^2) for (cu_mk23_247_2_vesely)

	x	*y*	*z*	U_{iso}*/U_{eq}
N1	0.8888 (3)	0.67268 (19)	0.67631 (11)	0.0379 (5)
O1	0.8743 (3)	0.60858 (18)	0.74085 (9)	0.0417 (4)
O2	0.8379 (3)	0.4118 (2)	0.77552 (9)	0.0424 (5)
O3	0.3839 (3)	0.23604 (17)	0.56801 (10)	0.0400 (4)
O4	0.4388 (2)	0.55032 (14)	0.68994 (10)	0.0353 (4)
C1	0.8467 (3)	0.4841 (2)	0.72931 (12)	0.0322 (5)
C2	0.8704 (3)	0.59334 (19)	0.62694 (12)	0.0273 (4)
C3	0.8365 (3)	0.46345 (19)	0.65187 (11)	0.0250 (4)
C4	0.6413 (3)	0.41371 (18)	0.62956 (11)	0.0243 (4)
H4	0.607967	0.457690	0.585880	0.029*
C5	0.6801 (3)	0.28135 (19)	0.60844 (10)	0.0265 (4)
C6	0.8581 (3)	0.25284 (19)	0.60871 (10)	0.0265 (4)
C7	0.9736 (3)	0.36030 (19)	0.63064 (12)	0.0281 (5)
H7A	1.051715	0.388734	0.592113	0.034*
H7B	1.052098	0.337045	0.670121	0.034*
C8	0.8707 (3)	0.63597 (19)	0.55492 (12)	0.0281 (5)
C9	0.8484 (3)	0.5525 (2)	0.50019 (11)	0.0278 (5)
H9	0.838243	0.466160	0.509468	0.033*
C10	0.8411 (4)	0.5943 (2)	0.43275 (13)	0.0346 (5)
H10	0.826850	0.536581	0.396069	0.042*
C11	0.8544 (4)	0.7198 (2)	0.41845 (14)	0.0433 (6)
H11	0.847235	0.748600	0.372154	0.052*
C12	0.8783 (5)	0.8035 (2)	0.47227 (15)	0.0457 (7)

H12	0.889115	0.889689	0.462538	0.055*
C13	0.8864 (4)	0.7627 (2)	0.53970 (14)	0.0389 (6)
H13	0.902682	0.820911	0.576045	0.047*
C14	0.4991 (3)	0.43053 (18)	0.68350 (11)	0.0243 (4)
C15	0.4176 (3)	0.3579 (2)	0.73078 (12)	0.0290 (5)
H15	0.436074	0.271362	0.737300	0.035*
C16	0.2978 (3)	0.4362 (2)	0.76931 (12)	0.0326 (5)
H16	0.221236	0.412121	0.806509	0.039*
C17	0.3148 (3)	0.5502 (2)	0.74258 (14)	0.0366 (5)
H17	0.249802	0.621246	0.758033	0.044*
C18	0.9485 (4)	0.1333 (2)	0.58937 (13)	0.0332 (5)
H18A	1.041260	0.149169	0.554081	0.050*
H18B	0.857408	0.075555	0.571081	0.050*
H18C	1.006131	0.096913	0.630276	0.050*
C19	0.5369 (4)	0.2015 (2)	0.58135 (12)	0.0340 (5)
H19	0.565319	0.116570	0.573619	0.041*

Atomic displacement parameters (Å^2) for (cu_mk23_247_2_vesely)

	U^{11}	U^{22}	U^{33}	U^{12}	U^{13}	U^{23}
N1	0.0436 (12)	0.0328 (10)	0.0373 (11)	0.0016 (9)	-0.0034 (9)	-0.0117 (8)
O1	0.0490 (11)	0.0477 (10)	0.0283 (8)	0.0083 (9)	-0.0043 (8)	-0.0147 (7)
O2	0.0336 (10)	0.0680 (12)	0.0257 (8)	0.0022 (9)	-0.0009 (7)	0.0066 (8)
O3	0.0323 (9)	0.0458 (10)	0.0419 (9)	-0.0059 (8)	-0.0043 (7)	-0.0106 (8)
O4	0.0362 (9)	0.0218 (7)	0.0478 (9)	0.0034 (6)	0.0121 (8)	0.0016 (7)
C1	0.0202 (11)	0.0478 (13)	0.0286 (11)	0.0044 (9)	0.0003 (8)	-0.0063 (10)
C2	0.0253 (10)	0.0235 (9)	0.0332 (11)	-0.0006 (8)	0.0025 (9)	-0.0080 (8)
C3	0.0234 (10)	0.0269 (10)	0.0249 (9)	0.0000 (8)	0.0012 (8)	-0.0023 (8)
C4	0.0247 (10)	0.0242 (9)	0.0238 (9)	-0.0011 (8)	0.0002 (8)	0.0010 (7)
C5	0.0324 (11)	0.0257 (10)	0.0215 (9)	-0.0007 (8)	0.0008 (8)	-0.0019 (8)
C6	0.0318 (11)	0.0271 (10)	0.0206 (9)	0.0010 (9)	0.0022 (8)	0.0004 (7)
C7	0.0259 (10)	0.0283 (10)	0.0302 (11)	0.0032 (8)	0.0018 (9)	-0.0015 (8)
C8	0.0280 (11)	0.0224 (9)	0.0338 (11)	-0.0043 (9)	0.0054 (9)	-0.0024 (8)
C9	0.0309 (12)	0.0226 (9)	0.0299 (10)	-0.0022 (8)	0.0045 (8)	-0.0008 (8)
C10	0.0380 (13)	0.0328 (11)	0.0332 (12)	-0.0022 (10)	0.0049 (10)	-0.0006 (9)
C11	0.0515 (16)	0.0378 (12)	0.0404 (13)	-0.0017 (12)	0.0112 (12)	0.0118 (10)
C12	0.0570 (17)	0.0264 (11)	0.0538 (16)	-0.0073 (12)	0.0132 (14)	0.0086 (11)
C13	0.0472 (15)	0.0242 (10)	0.0454 (13)	-0.0086 (10)	0.0114 (11)	-0.0056 (9)
C14	0.0231 (10)	0.0213 (9)	0.0285 (10)	0.0031 (7)	-0.0007 (8)	0.0005 (8)
C15	0.0287 (11)	0.0255 (10)	0.0328 (11)	0.0011 (8)	0.0030 (9)	0.0030 (8)

C16	0.0309 (12)	0.0368 (12)	0.0299 (11)	-0.0021 (9)	0.0057 (9)	-0.0013 (9)
C17	0.0314 (12)	0.0296 (10)	0.0488 (14)	0.0010 (10)	0.0129 (11)	-0.0092 (10)
C18	0.0364 (12)	0.0298 (10)	0.0335 (11)	0.0060 (9)	0.0010 (10)	-0.0027 (9)
C19	0.0344 (12)	0.0333 (11)	0.0343 (12)	-0.0029 (10)	-0.0014 (10)	-0.0072 (9)

Geometric parameters (Å, º) for (cu_mk23_247_2_vesely)

N1—C2	1.289 (3)	C8—C9	1.399 (3)
N1—O1	1.432 (3)	C8—C13	1.401 (3)
O1—C1	1.374 (3)	C9—C10	1.383 (3)
O2—C1	1.188 (3)	C9—H9	0.9500
O3—C19	1.212 (3)	C10—C11	1.383 (4)
O4—C17	1.367 (3)	C10—H10	0.9500
O4—C14	1.370 (2)	C11—C12	1.389 (4)
C1—C3	1.518 (3)	C11—H11	0.9500
C2—C8	1.469 (3)	C12—C13	1.379 (4)
C2—C3	1.500 (3)	C12—H12	0.9500
C3—C7	1.554 (3)	C13—H13	0.9500
C3—C4	1.591 (3)	C14—C15	1.345 (3)
C4—C14	1.488 (3)	C15—C16	1.429 (3)
C4—C5	1.510 (3)	C15—H15	0.9500
C4—H4	0.9999	C16—C17	1.338 (4)
C5—C6	1.343 (3)	C16—H16	0.9500
C5—C19	1.457 (3)	C17—H17	0.9500
C6—C18	1.496 (3)	C18—H18A	0.9800
C6—C7	1.496 (3)	C18—H18B	0.9800
C7—H7A	0.9900	C18—H18C	0.9800
C7—H7B	0.9900	C19—H19	0.9500
C2—N1—O1	108.67 (19)	C10—C9—C8	120.7 (2)
C1—O1—N1	109.83 (17)	C10—C9—H9	119.6
C17—O4—C14	106.43 (18)	C8—C9—H9	119.6
O2—C1—O1	121.7 (2)	C9—C10—C11	120.3 (2)
O2—C1—C3	130.2 (2)	C9—C10—H10	119.8
O1—C1—C3	108.1 (2)	C11—C10—H10	119.8
N1—C2—C8	119.8 (2)	C10—C11—C12	119.5 (2)
N1—C2—C3	113.4 (2)	C10—C11—H11	120.2
C8—C2—C3	126.67 (18)	C12—C11—H11	120.2
C2—C3—C1	99.97 (18)	C13—C12—C11	120.6 (2)
C2—C3—C7	118.28 (18)	C13—C12—H12	119.7

C1—C3—C7	109.51 (18)	C11—C12—H12	119.7
C2—C3—C4	112.08 (18)	C12—C13—C8	120.4 (2)
C1—C3—C4	111.22 (17)	C12—C13—H13	119.8
C7—C3—C4	105.76 (16)	C8—C13—H13	119.8
C14—C4—C5	115.94 (17)	C15—C14—O4	109.97 (19)
C14—C4—C3	113.65 (17)	C15—C14—C4	136.03 (19)
C5—C4—C3	102.77 (17)	O4—C14—C4	113.93 (18)
C14—C4—H4	111.4	C14—C15—C16	106.65 (19)
C5—C4—H4	105.3	C14—C15—H15	126.7
C3—C4—H4	106.9	C16—C15—H15	126.7
C6—C5—C19	124.7 (2)	C17—C16—C15	106.4 (2)
C6—C5—C4	113.5 (2)	C17—C16—H16	126.8
C19—C5—C4	121.2 (2)	C15—C16—H16	126.8
C5—C6—C18	128.9 (2)	C16—C17—O4	110.6 (2)
C5—C6—C7	112.10 (19)	C16—C17—H17	124.7
C18—C6—C7	119.0 (2)	O4—C17—H17	124.7
C6—C7—C3	105.10 (18)	C6—C18—H18A	109.5
C6—C7—H7A	110.7	C6—C18—H18B	109.5
C3—C7—H7A	110.7	H18A—C18—H18B	109.5
C6—C7—H7B	110.7	C6—C18—H18C	109.5
C3—C7—H7B	110.7	H18A—C18—H18C	109.5
H7A—C7—H7B	108.8	H18B—C18—H18C	109.5
C9—C8—C13	118.4 (2)	O3—C19—C5	124.4 (2)
C9—C8—C2	121.24 (18)	O3—C19—H19	117.8
C13—C8—C2	120.3 (2)	C5—C19—H19	117.8

Document origin: *publCIF* [Westrip, S. P. (2010). *J. Apply. Cryst.*, **43**, 920-925].

Crystallization

Single crystal of product **3r-major** (mk23_225_1) was prepared by volatilization using a methanol as a solvent. Suitable crystals were selected and collected on a Bruker D8 VENTURE Kappa Duo PHOTONIII instrument by an IµS microfocus sealed tube with Mo Kα (λ = 0.71073) radiation at a low temperature of 120 K.

Refinement

Crystal data, data collection and structure refinement details are summarized in Table 1.

Results and discussion

Table 1
Experimental details

Crystal data	
Chemical formula	$C_{19}H_{21}NO_3$
M_r	311.37
Crystal system, space group	Monoclinic, $P2_1$
Temperature (K)	120
a, *b*, *c* (Å)	8.7310 (11), 7.1858 (9), 13.1687 (17)
[illegible] (°)	91.407 (3)
V (Å^3)	825.94 (18)
Z	2
Radiation type	Cu *K*[illegible]
[illegible] (mm^{-1})	0.68
Crystal size (mm)	0.36 × 0.17 × 0.16
Data collection	
Diffractometer	Bruker D8 VENTURE Kappa Duo PHOTONIII CMOS
Absorption correction	Multi-scan<br>Krause, L., Herbst-Irmer, R., Sheldrick, G. M., Stalke, D. (2015). "Comparison of silver and molybdenum microfocus X-ray sources for single-crystal structure determination" J. Appl. Cryst. 48, 3-10.<br>doi:10.1107/S1600576714022985
T_{min}, T_{max}	0.79, 0.90
No. of measured, independent and observed [I > 2[illegible](I)] reflections	21538, 3395, 3384
R_{int}	0.025
(sin [illegible]/[illegible])$_{max}$ (Å^{-1})	0.633
Refinement	
$R[F^2 > 2$[illegible]$(F^2)]$, $wR(F^2)$, S	0.027, 0.071, 1.07

No. of reflections	3395
No. of parameters	212
No. of restraints	1
H-atom treatment	H-atom parameters constrained
□□$_{max}$, □□$_{min}$ (e Å^{-3})	0.20, -0.14
Absolute structure	Flack x determined using 1496 quotients [(I+)-(I-)]/[(I+)+(I-)] (Parsons, Flack and Wagner, Acta Cryst. B69 (2013) 249-259).
Absolute structure parameter	-0.03 (5)

Computer programs: Bruker Instrument Service v8.5.1.2 Release, *SAINT* V8.40B (Bruker AXS LLC, 2019), SHELXT 2018/2 (Sheldrick, 2018), *SHELXL2019*/2 (Sheldrick, 2019).

References

NOT FOUND

Document origin: *publCIF* [Westrip, S. P. (2010). *J. Apply. Cryst.*, **43**, 920-925].

Computing details

Data collection: Bruker Instrument Service v8.5.1.2 Release; cell refinement: *SAINT* V8.40B (Bruker AXS LLC, 2019); data reduction: *SAINT* V8.40B (Bruker AXS LLC, 2019); program(s) used to solve structure: SHELXT 2018/2 (Sheldrick, 2018); program(s) used to refine structure: *SHELXL2019*/2 (Sheldrick, 2019).

(cu_mk23_225_1_vesely)

Crystal data

$C_{19}H_{21}NO_3$	$F(000)$ = 332
M_r = 311.37	D_x = 1.252 Mg m^{-3}
Monoclinic, $P2_1$	Cu *K*□ radiation, □ = 1.54178 Å
a = 8.7310 (11) Å	Cell parameters from 9922 reflections
b = 7.1858 (9) Å	□ = 3.4–77.5°
c = 13.1687 (17) Å	□ = 0.68 mm^{-1}
□ = 91.407 (3)°	*T* = 120 K
V = 825.94 (18) Å^3	Prism, colourless
Z = 2	0.36 × 0.17 × 0.16 mm

Data collection

Bruker D8 VENTURE Kappa Duo PHOTONIII CMOS diffractometer	3395 independent reflections
Radiation source: I□S micro-focus sealed tube	3384 reflections with $I > 2$□(I)
Helios Cu multilayer optic monochromator	R_{int} = 0.025
□ and □ scans	□$_{max}$ = 77.5°, □$_{min}$ = 3.4°
Absorption correction: multi-scan Krause, L., Herbst-Irmer, R., Sheldrick, G. M., Stalke, D. (2015). "Comparison of silver and molybdenum microfocus X-ray sources for single-crystal structure determination" J. Appl. Cryst. 48, 3-10. doi:10.1107/S1600576714022985	*h* = -10□11
T_{min} = 0.79, T_{max} = 0.90	*k* = -8□9
21538 measured reflections	*l* = -16□16

Refinement

Refinement on F^2	Secondary atom site location: difference Fourier map
Least-squares matrix: full	Hydrogen site location: inferred from neighbouring sites
$R[F^2 > 2$□$(F^2)]$ = 0.027	H-atom parameters constrained
$wR(F^2)$ = 0.071	$w = 1/[$□$^2(F_o^2) + (0.0386P)^2 + 0.141P]$ where $P = (F_o^2 + 2F_c^2)/3$
S = 1.07	(□/□)$_{max}$ < 0.001

3395 reflections	□□$_{max}$ = 0.20 e Å^{-3}
212 parameters	□□$_{min}$ = -0.14 e Å^{-3}
1 restraint	Absolute structure: Flack x determined using 1496 quotients [(I+)-(I-)]/[(I+)+(I-)] (Parsons, Flack and Wagner, Acta Cryst. B69 (2013) 249-259).
Primary atom site location: structure-invariant direct methods	Absolute structure parameter: -0.03 (5)

Special details

Geometry. All esds (except the esd in the dihedral angle between two l.s. planes) are estimated using the full covariance matrix. The cell esds are taken into account individually in the estimation of esds in distances, angles and torsion angles; correlations between esds in cell parameters are only used when they are defined by crystal symmetry. An approximate (isotropic) treatment of cell esds is used for estimating esds involving l.s. planes.

Fractional atomic coordinates and isotropic or equivalent isotropic displacement parameters (Å^2) for (cu_mk23_225_1_vesely)

	x	*y*	*z*	U_{iso}*/U_{eq}
O1	0.39531 (12)	0.54382 (19)	0.48623 (8)	0.0282 (3)
O2	0.21928 (12)	0.44932 (17)	0.37198 (8)	0.0255 (3)
O3	0.64929 (15)	0.69316 (17)	0.04521 (9)	0.0297 (3)
C2	0.61631 (16)	0.4938 (2)	0.41003 (10)	0.0169 (3)
N1	0.56169 (15)	0.5462 (2)	0.49432 (10)	0.0258 (3)
C1	0.35109 (16)	0.4788 (2)	0.39344 (11)	0.0184 (3)
C3	0.49228 (15)	0.45009 (19)	0.33027 (10)	0.0151 (3)
C4	0.49678 (16)	0.5819 (2)	0.23312 (10)	0.0159 (3)
H4	0.564893	0.690816	0.248250	0.019*
C5	0.57306 (15)	0.4554 (2)	0.15726 (11)	0.0168 (3)
C6	0.56948 (17)	0.2751 (2)	0.18464 (11)	0.0177 (3)
C7	0.49431 (17)	0.2524 (2)	0.28476 (11)	0.0172 (3)
H7A	0.553702	0.166336	0.329310	0.021*
H7B	0.388906	0.203446	0.275414	0.021*
C8	0.79109 (16)	0.4880 (2)	0.40371 (11)	0.0190 (3)
C9A	0.84547 (19)	0.6562 (3)	0.34203 (14)	0.0289 (4)
H9AA	0.957690	0.660642	0.343925	0.043*
H9AB	0.804933	0.770905	0.371388	0.043*
H9AC	0.808442	0.644365	0.271469	0.043*
C9B	0.84716 (18)	0.3079 (2)	0.35437 (13)	0.0243 (3)
H9BA	0.959374	0.304178	0.357701	0.036*
H9BB	0.811772	0.303922	0.283205	0.036*

H9BC	0.806167	0.200552	0.390692	0.036*
C9C	0.86301 (18)	0.4984 (3)	0.51118 (12)	0.0278 (4)
H9CA	0.974897	0.494949	0.507067	0.042*
H9CB	0.828165	0.392280	0.551273	0.042*
H9CC	0.831865	0.614512	0.543781	0.042*
C10	0.34013 (16)	0.6515 (2)	0.19601 (10)	0.0173 (3)
C11	0.24655 (18)	0.5498 (2)	0.12903 (11)	0.0228 (3)
H11	0.279708	0.432382	0.104809	0.027*
C12	0.10418 (19)	0.6204 (3)	0.09748 (12)	0.0287 (4)
H12	0.040642	0.549905	0.052283	0.034*
C13	0.05462 (19)	0.7920 (3)	0.13137 (13)	0.0299 (4)
H13	-0.041669	0.840006	0.108699	0.036*
C14	0.14655 (19)	0.8936 (2)	0.19871 (14)	0.0290 (4)
H14	0.113113	1.011118	0.222622	0.035*
C15	0.28796 (18)	0.8226 (2)	0.23112 (12)	0.0233 (3)
H15	0.349808	0.891840	0.277896	0.028*
C16	0.64155 (17)	0.5279 (2)	0.06546 (11)	0.0215 (3)
H16	0.682463	0.441462	0.018754	0.026*
C17	0.6276 (2)	0.1073 (2)	0.13177 (12)	0.0242 (3)
H17A	0.543560	0.018516	0.120882	0.036*
H17B	0.708799	0.049130	0.173515	0.036*
H17C	0.668677	0.143622	0.066078	0.036*

Atomic displacement parameters (Å^2) for (cu_mk23_225_1_vesely)

	U^{11}	U^{22}	U^{33}	U^{12}	U^{13}	U^{23}
O1	0.0173 (5)	0.0450 (7)	0.0225 (5)	0.0009 (5)	0.0049 (4)	-0.0099 (5)
O2	0.0148 (5)	0.0343 (7)	0.0274 (6)	-0.0020 (5)	0.0022 (4)	0.0055 (5)
O3	0.0392 (7)	0.0247 (6)	0.0255 (6)	-0.0009 (5)	0.0080 (5)	0.0061 (5)
C2	0.0167 (6)	0.0179 (6)	0.0160 (6)	-0.0010 (5)	0.0000 (5)	0.0001 (5)
N1	0.0177 (6)	0.0388 (8)	0.0210 (6)	-0.0009 (6)	0.0010 (5)	-0.0074 (6)
C1	0.0172 (6)	0.0199 (7)	0.0180 (6)	0.0008 (6)	0.0025 (5)	0.0019 (6)
C3	0.0141 (6)	0.0161 (7)	0.0152 (6)	-0.0013 (5)	0.0010 (5)	0.0002 (5)
C4	0.0160 (6)	0.0172 (7)	0.0145 (6)	-0.0006 (5)	0.0012 (5)	0.0007 (5)
C5	0.0158 (6)	0.0191 (7)	0.0155 (6)	0.0019 (6)	0.0002 (5)	-0.0012 (5)
C6	0.0171 (7)	0.0206 (7)	0.0152 (6)	0.0001 (5)	-0.0013 (5)	-0.0015 (5)
C7	0.0192 (7)	0.0164 (7)	0.0160 (7)	-0.0015 (5)	0.0001 (5)	-0.0009 (5)
C8	0.0141 (6)	0.0239 (7)	0.0190 (6)	-0.0012 (6)	-0.0008 (5)	-0.0010 (6)
C9A	0.0177 (7)	0.0309 (9)	0.0381 (9)	-0.0061 (7)	0.0001 (6)	0.0087 (7)
C9B	0.0159 (7)	0.0296 (9)	0.0274 (8)	0.0028 (6)	-0.0011 (6)	-0.0042 (6)

C9C	0.0188 (7)	0.0412 (10)	0.0230 (7)	-0.0003 (7)	-0.0051 (5)	-0.0043 (7)
C10	0.0170 (7)	0.0187 (7)	0.0164 (6)	0.0004 (6)	0.0022 (5)	0.0028 (5)
C11	0.0209 (7)	0.0293 (8)	0.0183 (6)	0.0013 (6)	0.0001 (5)	-0.0032 (6)
C12	0.0207 (7)	0.0454 (10)	0.0200 (7)	-0.0003 (7)	-0.0016 (6)	0.0005 (7)
C13	0.0200 (7)	0.0429 (10)	0.0271 (8)	0.0082 (7)	0.0026 (6)	0.0127 (7)
C14	0.0261 (8)	0.0236 (8)	0.0377 (9)	0.0082 (7)	0.0085 (7)	0.0069 (7)
C15	0.0229 (7)	0.0191 (8)	0.0281 (8)	0.0006 (6)	0.0039 (6)	0.0018 (6)
C16	0.0224 (7)	0.0243 (8)	0.0178 (6)	0.0026 (6)	0.0032 (5)	0.0018 (6)
C17	0.0315 (8)	0.0202 (7)	0.0210 (7)	0.0040 (6)	0.0021 (6)	-0.0036 (6)

Geometric parameters (Å, º) for (cu_mk23_225_1_vesely)

O1—C1	1.3556 (19)	C9A—H9AB	0.9800
O1—N1	1.4541 (17)	C9A—H9AC	0.9800
O2—C1	1.1971 (19)	C9B—H9BA	0.9800
O3—C16	1.219 (2)	C9B—H9BB	0.9800
C2—N1	1.2758 (19)	C9B—H9BC	0.9800
C2—C3	1.5227 (18)	C9C—H9CA	0.9800
C2—C8	1.5309 (19)	C9C—H9CB	0.9800
C1—C3	1.5180 (19)	C9C—H9CC	0.9800
C3—C7	1.5424 (19)	C10—C11	1.394 (2)
C3—C4	1.5930 (19)	C10—C15	1.394 (2)
C4—C5	1.517 (2)	C11—C12	1.396 (2)
C4—C10	1.5253 (19)	C11—H11	0.9500
C4—H4	1.0000	C12—C13	1.384 (3)
C5—C6	1.345 (2)	C12—H12	0.9500
C5—C16	1.458 (2)	C13—C14	1.389 (3)
C6—C17	1.488 (2)	C13—H13	0.9500
C6—C7	1.496 (2)	C14—C15	1.393 (2)
C7—H7A	0.9900	C14—H14	0.9500
C7—H7B	0.9900	C15—H15	0.9500
C8—C9B	1.534 (2)	C16—H16	0.9500
C8—C9C	1.5361 (19)	C17—H17A	0.9800
C8—C9A	1.538 (2)	C17—H17B	0.9800
C9A—H9AA	0.9800	C17—H17C	0.9800
C1—O1—N1	109.39 (10)	H9AA—C9A—H9AC	109.5
N1—C2—C3	112.73 (12)	H9AB—C9A—H9AC	109.5
N1—C2—C8	116.73 (12)	C8—C9B—H9BA	109.5
C3—C2—C8	130.53 (12)	C8—C9B—H9BB	109.5

C2—N1—O1	109.07 (11)	H9BA—C9B—H9BB	109.5
O2—C1—O1	121.63 (13)	C8—C9B—H9BC	109.5
O2—C1—C3	129.47 (14)	H9BA—C9B—H9BC	109.5
O1—C1—C3	108.89 (12)	H9BB—C9B—H9BC	109.5
C1—C3—C2	99.64 (11)	C8—C9C—H9CA	109.5
C1—C3—C7	110.80 (11)	C8—C9C—H9CB	109.5
C2—C3—C7	116.31 (12)	H9CA—C9C—H9CB	109.5
C1—C3—C4	113.31 (12)	C8—C9C—H9CC	109.5
C2—C3—C4	113.59 (11)	H9CA—C9C—H9CC	109.5
C7—C3—C4	103.59 (11)	H9CB—C9C—H9CC	109.5
C5—C4—C10	112.96 (11)	C11—C10—C15	118.73 (14)
C5—C4—C3	101.10 (11)	C11—C10—C4	122.56 (13)
C10—C4—C3	114.37 (11)	C15—C10—C4	118.71 (13)
C5—C4—H4	109.4	C10—C11—C12	120.11 (16)
C10—C4—H4	109.4	C10—C11—H11	119.9
C3—C4—H4	109.4	C12—C11—H11	119.9
C6—C5—C16	125.38 (13)	C13—C12—C11	120.70 (16)
C6—C5—C4	112.77 (13)	C13—C12—H12	119.6
C16—C5—C4	121.84 (13)	C11—C12—H12	119.6
C5—C6—C17	130.14 (14)	C12—C13—C14	119.58 (16)
C5—C6—C7	110.81 (13)	C12—C13—H13	120.2
C17—C6—C7	119.05 (13)	C14—C13—H13	120.2
C6—C7—C3	104.54 (11)	C13—C14—C15	119.82 (16)
C6—C7—H7A	110.8	C13—C14—H14	120.1
C3—C7—H7A	110.8	C15—C14—H14	120.1
C6—C7—H7B	110.8	C14—C15—C10	121.04 (16)
C3—C7—H7B	110.8	C14—C15—H15	119.5
H7A—C7—H7B	108.9	C10—C15—H15	119.5
C2—C8—C9B	112.02 (12)	O3—C16—C5	123.73 (14)
C2—C8—C9C	109.52 (12)	O3—C16—H16	118.1
C9B—C8—C9C	107.68 (13)	C5—C16—H16	118.1
C2—C8—C9A	109.17 (12)	C6—C17—H17A	109.5
C9B—C8—C9A	109.49 (13)	C6—C17—H17B	109.5
C9C—C8—C9A	108.90 (13)	H17A—C17—H17B	109.5
C8—C9A—H9AA	109.5	C6—C17—H17C	109.5
C8—C9A—H9AB	109.5	H17A—C17—H17C	109.5
H9AA—C9A—H9AB	109.5	H17B—C17—H17C	109.5
C8—C9A—H9AC	109.5		

Document origin: *publCIF* [Westrip, S. P. (2010). *J. Apply. Cryst.*, **43**, 920-925].

Refinement

Crystal data, data collection and structure refinement details are summarized in Table 1.

Results and discussion

Table 1
Experimental details

Crystal data	
Chemical formula	$C_{20}H_{16}BrO_3 \cdot C_8H_{12}N$
M_r	506.42
Crystal system, space group	Orthorhombic, $P2_12_12_1$
Temperature (K)	120
a, *b*, *c* (Å)	6.1946 (3), 17.9333 (9), 22.1148 (11)
V (Å^3)	2456.7 (2)
Z	4
Radiation type	Mo *K*□
□ (mm^{-1})	1.70
Crystal size (mm)	0.59 × 0.09 × 0.04
Data collection	
Diffractometer	Bruker D8 VENTURE Kappa Duo PHOTONIII CMOS
Absorption correction	Multi-scan<br>*SADABS2016*/2 - Bruker AXS area detector scaling and absorption correction
T_{min}, T_{max}	0.77, 0.94
No. of measured, independent and observed [*I* > 2□(*I*)] reflections	19966, 5605, 4574
R_{int}	0.050
(sin □/□)$_{max}$ (Å^{-1})	0.650
Refinement	
R[F^2 > 2□(F^2)], *wR*(F^2), *S*	0.036, 0.071, 1.02
No. of reflections	5605
No. of parameters	303
H-atom treatment	H atoms treated by a mixture of independent and constrained refinement
□□$_{max}$, □□$_{min}$ (e Å^{-3})	0.24, -0.40
Absolute structure	Flack x determined using 1633 quotients [(I+)-(I-)]/[(I+)+(I-)] (Parsons, Flack and Wagner, Acta Cryst. B69 (2013) 249-259).

Absolute structure parameter	-0.009 (4)

Computer programs: Bruker Instrument Service vV6.2.9, *SAINT* V8.38A (Bruker AXS Inc., 2017), SHELXT 2014/5 (Sheldrick, 2014), *SHELXL2019*/2 (Sheldrick, 2019).

Table 2
Hydrogen-bond geometry (Å, º) for (jv_sp18_276_vesely)

D—H···*A*	*D*—H	H···*A*	*D*···*A*	*D*—H···*A*
N1—H1*C*···O3^{i}	0.97	1.78	2.742 (4)	173
N1—H1*B*···O3^{ii}	0.84	1.90	2.737 (3)	174
N1—H1*A*···O2	0.95	1.73	2.676 (4)	172

Symmetry codes: (i) *x*+1, *y*, *z*; (ii) *x*+1/2, -*y*+3/2, -*z*+1.

References

NOT FOUND

Document origin: *publCIF* [Westrip, S. P. (2010). *J. Apply. Cryst.*, **43**, 920-925].

Computing details

Data collection: Bruker Instrument Service vV6.2.9; cell refinement: *SAINT* V8.38A (Bruker AXS Inc., 2017); data reduction: *SAINT* V8.38A (Bruker AXS Inc., 2017); program(s) used to solve structure: SHELXT 2014/5 (Sheldrick, 2014); program(s) used to refine structure: *SHELXL2019*/2 (Sheldrick, 2019).

(jv_sp18_276_vesely)

Crystal data

$C_{20}H_{16}BrO_3{\cdot}C_8H_{12}N$	D_x = 1.369 Mg m^{-3}
M_r = 506.42	Mo *K*[illegible] radiation, [illegible] = 0.71073 Å
Orthorhombic, $P2_12_12_1$	Cell parameters from 9894 reflections
a = 6.1946 (3) Å	[illegible] = 2.3–27.4°
b = 17.9333 (9) Å	[illegible] = 1.70 mm^{-1}
c = 22.1148 (11) Å	*T* = 120 K
V = 2456.7 (2) Å^3	Needle, colourless
Z = 4	0.59 × 0.09 × 0.04 mm
F(000) = 1048	

Data collection

Bruker D8 VENTURE Kappa Duo PHOTONIII CMOS diffractometer	5605 independent reflections
Radiation source: I[illegible]S micro-focus sealed tube	4574 reflections with *I* > 2[illegible](*I*)
Helios Cu multilayer optic monochromator	R_{int} = 0.050
[illegible] and [illegible] scans	[illegible]$_{max}$ = 27.5°, [illegible]$_{min}$ = 2.2°
Absorption correction: multi-scan *SADABS2016*/2 - Bruker AXS area detector scaling and absorption correction	*h* = -6[illegible]8
T_{min} = 0.77, T_{max} = 0.94	*k* = -23[illegible]19
19966 measured reflections	*l* = -24[illegible]28

Refinement

Refinement on F^2	Hydrogen site location: mixed
Least-squares matrix: full	H atoms treated by a mixture of independent and constrained refinement
$R[F^2 > 2$[illegible]$(F^2)]$ = 0.036	$w = 1/[$[illegible]$^2(F_o^2) + (0.0331P)^2]$ where $P = (F_o^2 + 2F_c^2)/3$
$wR(F^2)$ = 0.071	([illegible]/[illegible])$_{max}$ = 0.001
S = 1.02	[illegible]$_{max}$ = 0.24 e Å^{-3}
5605 reflections	[illegible]$_{min}$ = -0.40 e Å^{-3}
303 parameters	Absolute structure: Flack x determined using 1633 quotients [(I+)-(I-)]/[(I+)+(I-)] (Parsons, Flack and Wagner, Acta Cryst. B69 (2013) 249-

	259).
0 restraints	Absolute structure parameter: -0.009 (4)

Special details

Geometry. All esds (except the esd in the dihedral angle between two l.s. planes) are estimated using the full covariance matrix. The cell esds are taken into account individually in the estimation of esds in distances, angles and torsion angles; correlations between esds in cell parameters are only used when they are defined by crystal symmetry. An approximate (isotropic) treatment of cell esds is used for estimating esds involving l.s. planes.

Fractional atomic coordinates and isotropic or equivalent isotropic displacement parameters (Å^2) for (jv_sp18_276_vesely)

	x	*y*	*z*	U_{iso}*/U_{eq}
Br1	0.21186 (6)	0.38567 (2)	0.59832 (2)	0.02839 (11)
O1	-0.3478 (4)	0.67991 (14)	0.76110 (11)	0.0314 (6)
N1	0.8209 (5)	0.80388 (13)	0.53410 (12)	0.0181 (6)
H1C	0.961845	0.791852	0.550167	0.070 (16)*
H1B	0.793045	0.781552	0.501767	0.048 (13)*
H1A	0.711945	0.788752	0.561667	0.031 (10)*
C1	0.3369 (5)	0.76216 (16)	0.62176 (15)	0.0176 (7)
O2	0.5383 (4)	0.76573 (14)	0.62022 (11)	0.0276 (6)
C2	0.2261 (5)	0.75912 (16)	0.68137 (13)	0.0155 (7)
C3	0.2664 (5)	0.80225 (15)	0.72929 (14)	0.0178 (7)
O3	0.2186 (4)	0.75936 (12)	0.57481 (10)	0.0241 (5)
C4	0.1136 (6)	0.78527 (16)	0.78007 (15)	0.0193 (7)
H4A	-0.005707	0.821934	0.781695	0.023*
H4AB	0.189246	0.784649	0.819511	0.023*
C5	0.0304 (5)	0.70704 (17)	0.76302 (14)	0.0160 (7)
H5	0.137744	0.669911	0.778323	0.019*
C6	0.0404 (5)	0.70564 (16)	0.69305 (14)	0.0154 (7)
H6	-0.096226	0.726362	0.675801	0.019*
C9	-0.1983 (6)	0.68215 (16)	0.85864 (14)	0.0179 (7)
C8	-0.1856 (6)	0.68817 (16)	0.79102 (15)	0.0180 (7)
C7	0.4301 (6)	0.86338 (18)	0.73537 (17)	0.0278 (9)
H7A	0.526932	0.852248	0.769235	0.042*
H7B	0.356186	0.910798	0.742875	0.042*
H7C	0.514286	0.867056	0.697962	0.042*
C10	-0.0260 (5)	0.65597 (17)	0.89306 (16)	0.0229 (8)
H10	0.108718	0.645485	0.874401	0.027*
C11	-0.0523 (7)	0.6452 (2)	0.95490 (17)	0.0322 (10)

H11	0.063999	0.626291	0.978284	0.039*
C12	-0.2443 (7)	0.6618 (2)	0.98232 (16)	0.0358 (10)
H12	-0.261144	0.653671	1.024511	0.043*
C13	-0.4141 (7)	0.6902 (2)	0.94893 (18)	0.0316 (10)
H13	-0.545184	0.703523	0.968438	0.038*
C14	-0.3929 (6)	0.69934 (18)	0.88696 (17)	0.0244 (8)
H14	-0.511128	0.717328	0.863810	0.029*
C15	0.0793 (5)	0.62744 (17)	0.66867 (14)	0.0161 (7)
C16	0.2813 (6)	0.59537 (16)	0.67382 (15)	0.0203 (7)
H16	0.395671	0.623148	0.691497	0.024*
C17	0.3204 (6)	0.52306 (17)	0.65356 (15)	0.0210 (8)
H17	0.458879	0.501046	0.658222	0.025*
C18	0.1560 (6)	0.48433 (16)	0.62687 (15)	0.0201 (8)
C19	-0.0471 (6)	0.51411 (19)	0.62063 (16)	0.0248 (8)
H19	-0.159841	0.486187	0.602293	0.030*
C20	-0.0844 (6)	0.58653 (18)	0.64189 (16)	0.0219 (8)
H20	-0.223960	0.607896	0.637880	0.026*
C21	0.8207 (6)	0.88486 (19)	0.51738 (14)	0.0226 (7)
H21	0.702851	0.892998	0.487192	0.027*
C22	1.0331 (7)	0.9043 (2)	0.48724 (18)	0.0341 (10)
H22A	1.032372	0.956995	0.475570	0.051*
H22B	1.052308	0.873278	0.451141	0.051*
H22C	1.152011	0.895109	0.515509	0.051*
C23	0.7704 (6)	0.93220 (16)	0.57279 (15)	0.0232 (8)
C24	0.5808 (6)	0.97306 (19)	0.57409 (19)	0.0316 (9)
H24	0.486782	0.971591	0.540175	0.038*
C25	0.5256 (8)	1.0158 (2)	0.6235 (2)	0.0401 (12)
H25	0.394172	1.043103	0.623826	0.048*
C26	0.6626 (8)	1.01846 (19)	0.6722 (2)	0.0409 (12)
H26	0.626036	1.047962	0.706390	0.049*
C27	0.8544 (7)	0.9783 (2)	0.67191 (18)	0.0370 (11)
H27	0.949169	0.980675	0.705626	0.044*
C28	0.9073 (7)	0.93453 (19)	0.62197 (17)	0.0292 (9)
H28	1.037267	0.906392	0.621805	0.035*

Atomic displacement parameters (Å^2) for (jv_sp18_276_vesely)

	U^{11}	U^{22}	U^{33}	U^{12}	U^{13}	U^{23}
Br1	0.0431 (2)	0.01794 (15)	0.02411 (18)	-0.00377 (17)	0.00733 (18)	-0.00562 (15)

O1	0.0212 (14)	0.0457 (16)	0.0273 (15)	-0.0097 (12)	-0.0047 (12)	0.0093 (13)
N1	0.0188 (17)	0.0211 (14)	0.0142 (14)	-0.0005 (12)	0.0001 (13)	-0.0016 (12)
C1	0.020 (2)	0.0117 (15)	0.0208 (18)	0.0024 (13)	-0.0001 (16)	0.0004 (13)
O2	0.0203 (14)	0.0421 (14)	0.0203 (14)	-0.0034 (12)	0.0030 (11)	0.0033 (11)
C2	0.0163 (17)	0.0159 (14)	0.0144 (16)	0.0016 (14)	-0.0012 (15)	0.0033 (12)
C3	0.0195 (19)	0.0159 (14)	0.0180 (16)	-0.0022 (14)	0.0013 (15)	0.0012 (13)
O3	0.0228 (13)	0.0362 (13)	0.0133 (11)	0.0069 (12)	-0.0001 (12)	-0.0026 (10)
C4	0.0232 (18)	0.0180 (16)	0.0167 (18)	-0.0036 (14)	-0.0005 (15)	-0.0010 (14)
C5	0.0162 (18)	0.0152 (15)	0.0166 (17)	0.0007 (13)	-0.0013 (15)	0.0017 (13)
C6	0.0163 (17)	0.0155 (15)	0.0144 (17)	0.0014 (13)	-0.0009 (14)	0.0016 (13)
C9	0.0231 (18)	0.0128 (14)	0.0176 (16)	-0.0055 (15)	0.0031 (16)	0.0006 (12)
C8	0.0195 (19)	0.0129 (14)	0.0214 (17)	-0.0019 (14)	0.0002 (17)	0.0023 (13)
C7	0.033 (2)	0.0236 (18)	0.027 (2)	-0.0116 (16)	0.0038 (18)	-0.0036 (15)
C10	0.0240 (19)	0.0242 (17)	0.020 (2)	-0.0033 (15)	0.0006 (17)	0.0007 (15)
C11	0.038 (3)	0.040 (2)	0.018 (2)	-0.0112 (19)	-0.0022 (19)	0.0040 (17)
C12	0.049 (3)	0.042 (2)	0.0164 (18)	-0.020 (2)	0.006 (2)	-0.0033 (16)
C13	0.037 (2)	0.027 (2)	0.031 (2)	-0.0075 (18)	0.018 (2)	-0.0069 (18)
C14	0.0240 (19)	0.0186 (16)	0.031 (2)	-0.0010 (15)	0.0074 (17)	0.0009 (15)
C15	0.0190 (18)	0.0188 (17)	0.0105 (16)	-0.0015 (14)	0.0010 (14)	0.0028 (13)
C16	0.0199 (17)	0.0193 (16)	0.0216 (17)	-0.0061 (15)	-0.0036 (17)	-0.0012 (13)
C17	0.0191 (19)	0.0212 (16)	0.0226 (18)	0.0011 (15)	-0.0010 (16)	0.0017 (14)
C18	0.032 (2)	0.0133 (15)	0.0147 (17)	-0.0025 (14)	0.0054 (16)	-0.0016 (13)
C19	0.026 (2)	0.0265 (18)	0.022 (2)	-0.0083 (16)	-0.0037 (16)	-0.0066 (15)
C20	0.0166 (18)	0.0272 (18)	0.0219 (19)	-0.0010 (15)	-0.0015 (16)	-0.0036 (15)
C21	0.0284 (19)	0.0220 (15)	0.0172 (16)	-0.0014 (18)	-0.0036 (15)	0.0031 (15)
C22	0.045 (3)	0.032 (2)	0.025 (2)	-0.0102 (18)	0.0069 (19)	0.0033 (16)
C23	0.029 (2)	0.0165 (15)	0.0246 (18)	-0.0036 (16)	0.0015 (18)	0.0017 (13)
C24	0.032 (2)	0.0212 (18)	0.042 (2)	0.0017 (17)	0.0014 (19)	0.0057 (18)
C25	0.046 (3)	0.0166 (18)	0.057 (3)	0.0013 (19)	0.018 (2)	-0.0049 (19)
C26	0.065 (3)	0.0140 (17)	0.044 (3)	-0.014 (2)	0.023 (3)	-0.0106 (18)
C27	0.055 (3)	0.030 (2)	0.026 (2)	-0.015 (2)	0.001 (2)	-0.0031 (18)
C28	0.035 (2)	0.0253 (18)	0.027 (2)	-0.0041 (17)	-0.0017 (19)	0.0008 (16)

Geometric parameters (Å, º) for (jv_sp18_276_vesely)

Br1—C18	1.910 (3)	C12—H12	0.9500
O1—C8	1.212 (4)	C13—C14	1.386 (5)
N1—C21	1.499 (4)	C13—H13	0.9500
N1—H1C	0.9670	C14—H14	0.9500
N1—H1B	0.8374	C15—C16	1.382 (5)

N1—H1A	0.9491	C15—C20	1.384 (5)
C1—O2	1.250 (4)	C16—C17	1.393 (4)
C1—O3	1.272 (4)	C16—H16	0.9500
C1—C2	1.487 (4)	C17—C18	1.367 (5)
C2—C3	1.336 (4)	C17—H17	0.9500
C2—C6	1.520 (4)	C18—C19	1.374 (5)
C3—C7	1.500 (4)	C19—C20	1.400 (5)
C3—C4	1.500 (5)	C19—H19	0.9500
C4—C5	1.542 (4)	C20—H20	0.9500
C4—H4A	0.9900	C21—C22	1.516 (5)
C4—H4AB	0.9900	C21—C23	1.523 (5)
C5—C8	1.513 (5)	C21—H21	1.0000
C5—C6	1.549 (4)	C22—H22A	0.9800
C5—H5	1.0000	C22—H22B	0.9800
C6—C15	1.522 (4)	C22—H22C	0.9800
C6—H6	1.0000	C23—C28	1.380 (5)
C9—C10	1.392 (5)	C23—C24	1.385 (5)
C9—C14	1.393 (5)	C24—C25	1.378 (6)
C9—C8	1.501 (4)	C24—H24	0.9500
C7—H7A	0.9800	C25—C26	1.373 (6)
C7—H7B	0.9800	C25—H25	0.9500
C7—H7C	0.9800	C26—C27	1.389 (6)
C10—C11	1.391 (5)	C26—H26	0.9500
C10—H10	0.9500	C27—C28	1.394 (5)
C11—C12	1.367 (6)	C27—H27	0.9500
C11—H11	0.9500	C28—H28	0.9500
C12—C13	1.383 (6)		
C21—N1—H1C	107.9	C12—C13—C14	120.0 (4)
C21—N1—H1B	104.6	C12—C13—H13	120.0
H1C—N1—H1B	113.2	C14—C13—H13	120.0
C21—N1—H1A	115.8	C13—C14—C9	120.0 (4)
H1C—N1—H1A	110.0	C13—C14—H14	120.0
H1B—N1—H1A	105.4	C9—C14—H14	120.0
O2—C1—O3	123.7 (3)	C16—C15—C20	118.5 (3)
O2—C1—C2	119.1 (3)	C16—C15—C6	119.8 (3)
O3—C1—C2	117.1 (3)	C20—C15—C6	121.6 (3)
C3—C2—C1	126.6 (3)	C15—C16—C17	121.2 (3)

C3—C2—C6	111.8 (3)	C15—C16—H16	119.4
C1—C2—C6	121.5 (3)	C17—C16—H16	119.4
C2—C3—C7	128.4 (3)	C18—C17—C16	118.9 (3)
C2—C3—C4	111.0 (3)	C18—C17—H17	120.6
C7—C3—C4	120.5 (3)	C16—C17—H17	120.6
C3—C4—C5	102.3 (3)	C17—C18—C19	121.9 (3)
C3—C4—H4A	111.3	C17—C18—Br1	118.6 (3)
C5—C4—H4A	111.3	C19—C18—Br1	119.5 (3)
C3—C4—H4AB	111.3	C18—C19—C20	118.5 (3)
C5—C4—H4AB	111.3	C18—C19—H19	120.7
H4A—C4—H4AB	109.2	C20—C19—H19	120.7
C8—C5—C4	113.5 (3)	C15—C20—C19	121.0 (3)
C8—C5—C6	116.2 (3)	C15—C20—H20	119.5
C4—C5—C6	104.2 (2)	C19—C20—H20	119.5
C8—C5—H5	107.5	N1—C21—C22	109.3 (3)
C4—C5—H5	107.5	N1—C21—C23	110.0 (3)
C6—C5—H5	107.5	C22—C21—C23	113.8 (3)
C2—C6—C15	113.7 (3)	N1—C21—H21	107.9
C2—C6—C5	100.9 (2)	C22—C21—H21	107.9
C15—C6—C5	112.0 (2)	C23—C21—H21	107.9
C2—C6—H6	110.0	C21—C22—H22A	109.5
C15—C6—H6	110.0	C21—C22—H22B	109.5
C5—C6—H6	110.0	H22A—C22—H22B	109.5
C10—C9—C14	119.5 (3)	C21—C22—H22C	109.5
C10—C9—C8	121.9 (3)	H22A—C22—H22C	109.5
C14—C9—C8	118.5 (3)	H22B—C22—H22C	109.5
O1—C8—C9	119.4 (3)	C28—C23—C24	119.3 (3)
O1—C8—C5	122.5 (3)	C28—C23—C21	121.7 (3)
C9—C8—C5	118.1 (3)	C24—C23—C21	119.0 (3)
C3—C7—H7A	109.5	C25—C24—C23	121.4 (4)
C3—C7—H7B	109.5	C25—C24—H24	119.3
H7A—C7—H7B	109.5	C23—C24—H24	119.3
C3—C7—H7C	109.5	C26—C25—C24	119.2 (4)
H7A—C7—H7C	109.5	C26—C25—H25	120.4
H7B—C7—H7C	109.5	C24—C25—H25	120.4
C11—C10—C9	119.6 (4)	C25—C26—C27	120.5 (4)
C11—C10—H10	120.2	C25—C26—H26	119.8
C9—C10—H10	120.2	C27—C26—H26	119.8

C12—C11—C10	120.5 (4)	C26—C27—C28	119.8 (4)
C12—C11—H11	119.7	C26—C27—H27	120.1
C10—C11—H11	119.7	C28—C27—H27	120.1
C11—C12—C13	120.3 (4)	C23—C28—C27	119.8 (4)
C11—C12—H12	119.8	C23—C28—H28	120.1
C13—C12—H12	119.8	C27—C28—H28	120.1

Hydrogen-bond geometry (Å, º) for (jv_sp18_276_vesely)

D—H···*A*	*D*—H	H···*A*	*D*···*A*	*D*—H···*A*
N1—H1*C*···O3^{i}	0.97	1.78	2.742 (4)	173
N1—H1*B*···O3^{ii}	0.84	1.90	2.737 (3)	174
N1—H1*A*···O2	0.95	1.73	2.676 (4)	172

Symmetry codes: (i) *x*+1, *y*, *z*; (ii) *x*+1/2, -*y*+3/2, -*z*+1.

Document origin: *publCIF* [Westrip, S. P. (2010). *J. Apply. Cryst.*, **43**, 920-925].

HPLC Data

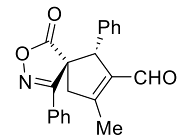

3a - major

Column: IA column
mobile phase: *n*-heptane / propan-2-ol= 90:10
λ= 250 nm, V= 1 ml/min, t= 25 °C
t_R= 11.1 min (major), t_R= 13.1 min (minor), ee= 88 %

major diastereoisomer

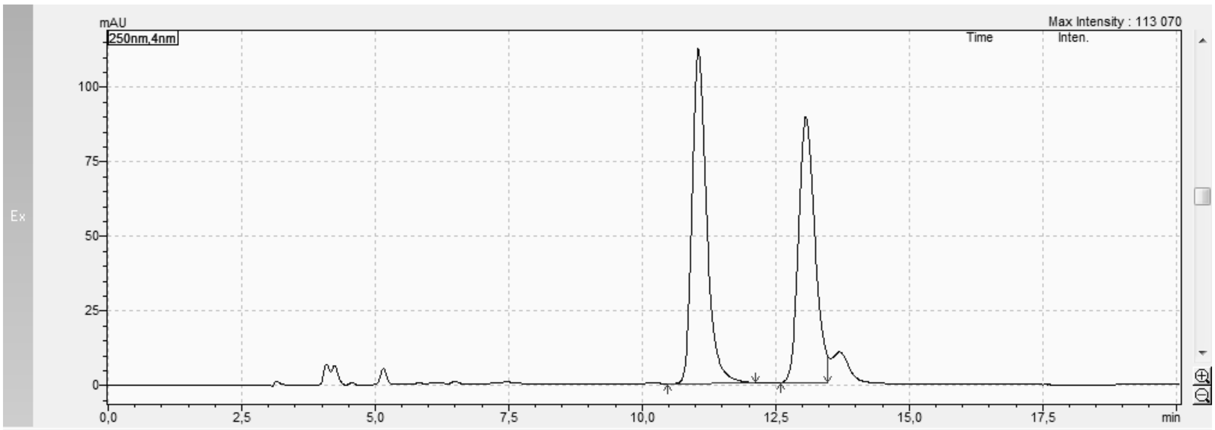

Results View - Peak Table

Peak Table | Compound | Group | Calibration Curve

Peak#	Ret. Time	Conc.	Area	Height	Similarity Index	Mark	Peak Start	Peak End	Area%
1	11,048	52,55190	2120860	112226	0,000000		10,485	12,117	52,552
2	13,062	47,44810	1914884	89330	0,000000	M	12,587	13,483	47,448
Total		100,00000	4035745	201556					100,000

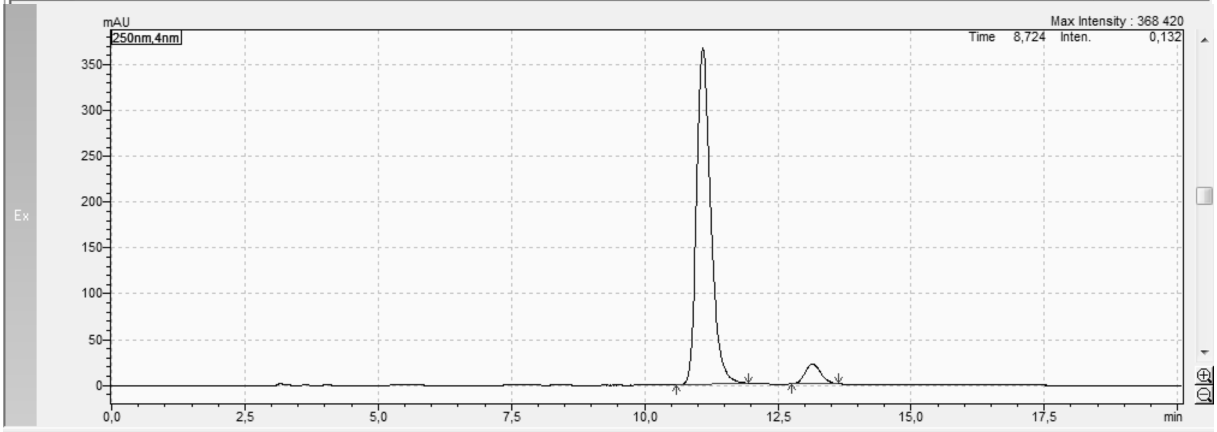

Results View - Peak Table

Peak Table | Compound | Group | Calibration Curve

Peak#	Ret. Time	Conc.	Area	Height	Similarity Index	Mark	Peak Start	Peak End	Area%
1	11,086	93,81687	6812877	367381	0,000000	M	10,603	11,947	93,817
2	13,142	6,18313	449012	22003	0,000000	M	12,768	13,643	6,183
Total		100,00000	7261888	389384					100,000

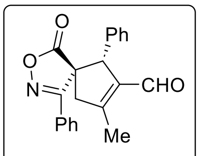

3a-minor

Column: IC column
mobile phase: *n*-heptane / propan-2-ol= 70:30
λ= 243 nm, *V*= 1 ml/min, *t*= 25 °C
t_R= 21.4 min (minor), t_R= 24.8 min (major), ee= 99 %

minor diastereoisomer

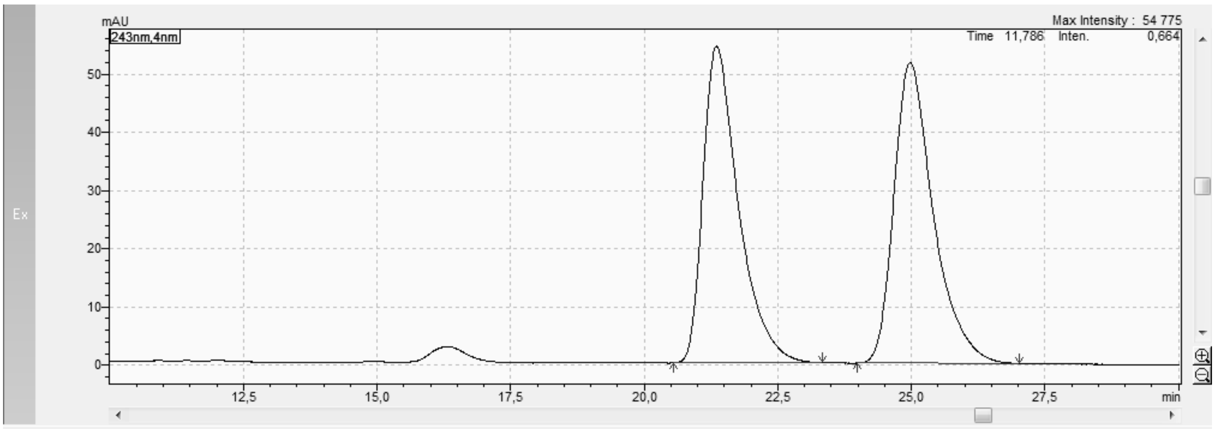

Results View - Peak Table

Peak Table | Compound | Group | Calibration Curve

Peak#	Ret. Time	Conc.	Area	Height	Similarity Index	Mark	Peak Start	Peak End	Area%
1	21,350	48,69614	2515884	54403	0,000000		20,555	23,328	48,696
2	24,973	51,30386	2650612	51611	0,000000		23,989	27,019	51,304
Total		100,00000	5166497	106013					100,000

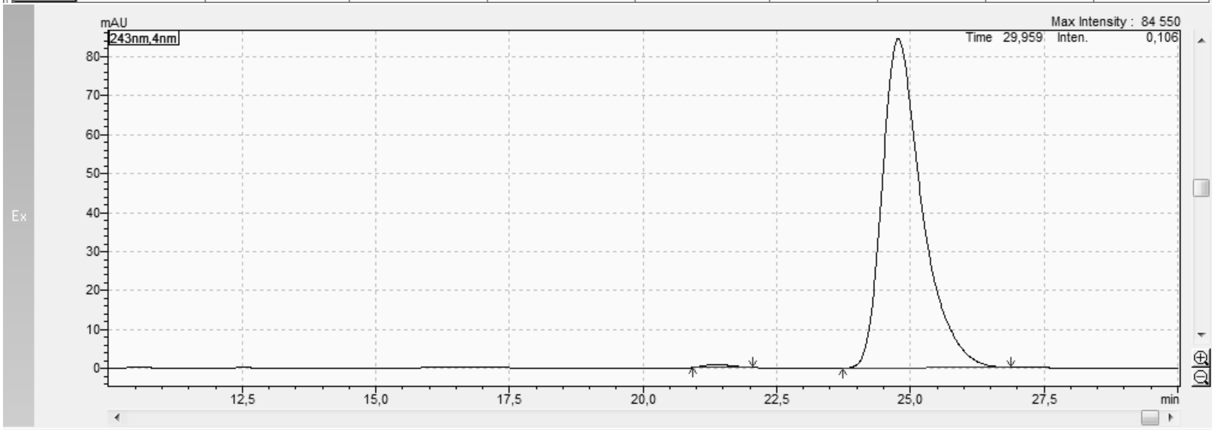

Results View - Peak Table

Peak Table | Compound | Group | Calibration Curve

Peak#	Ret. Time	Conc.	Area	Height	Similarity Index	Mark	Peak Start	Peak End	Area%
1	21,371	0,62131	27239	773	0,000000	M	20,928	22,048	0,621
2	24,769	99,37869	4356917	84323	0,000000		23,744	26,891	99,379
Total		100,00000	4384156	85096					100,000

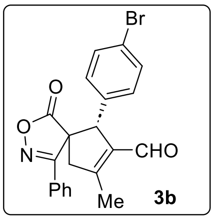

Column: IC column
mobile phase: *n*-heptane / propan-2-ol= 70:30
λ= 190 nm, V= 1 ml/min, t= 25 °C
t_R= 13.9 min (major), t_R= 16.0 min (minor), ee= 88 %

major diastereoisomer

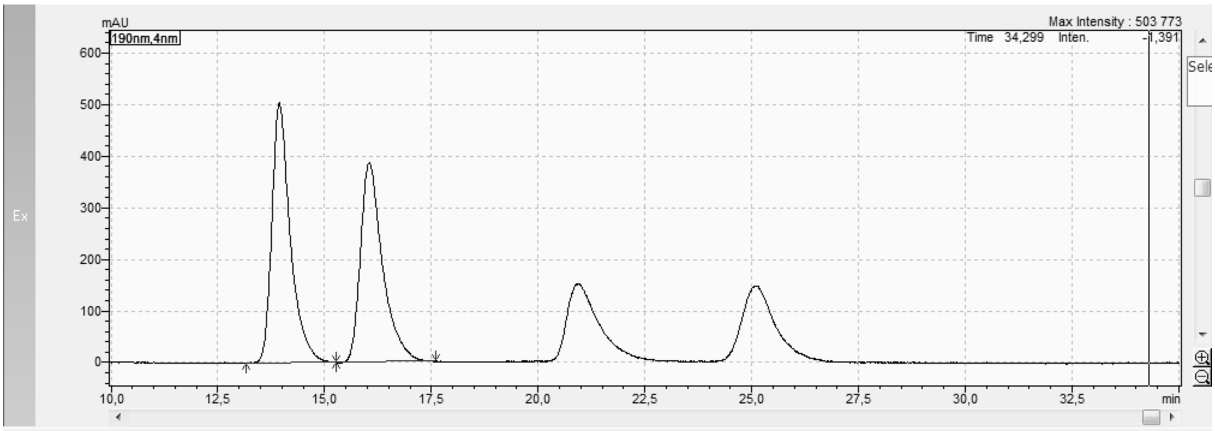

Results View - Peak Table

Peak Table | Compound | Group | Calibration Curve

Peak#	Ret. Time	Conc.	Area	Height	Similarity Index	Mark	Peak Start	Peak End	Area%
1	13,941	52,01469	15130577	504342	0,000000	M	13,163	15,275	52,015
2	16,057	47,98531	13958471	386576	0,000000	M	15,275	17,600	47,985
Total		100,00000	29089048	890918					100,000

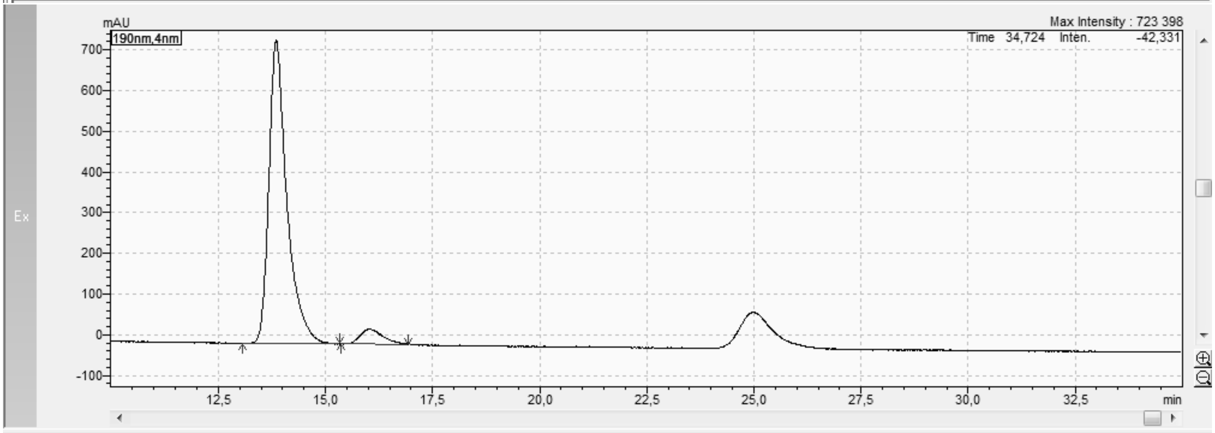

Results View - Peak Table

Peak Table | Compound | Group | Calibration Curve

Peak#	Ret. Time	Conc.	Area	Height	Similarity Index	Mark	Peak Start	Peak End	Area%
1	13,852	94,18124	21722207	744799	0,000000	M	13,067	15,339	94,181
2	16,044	5,81876	1342053	36771	0,000000	M	15,360	16,928	5,819
Total		100,00000	23064260	781570					100,000

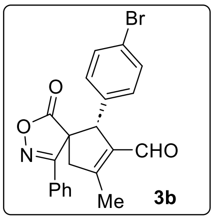

3b

Column: IC column
mobile phase: *n*-heptane / propan-2-ol= 70:30
λ= 190 nm, *V*= 1 ml/min, *t*= 25 °C
t_R= 20.1 min (minor), t_R= 25.0 min (major), ee= 99 %

minor diastereoisomer

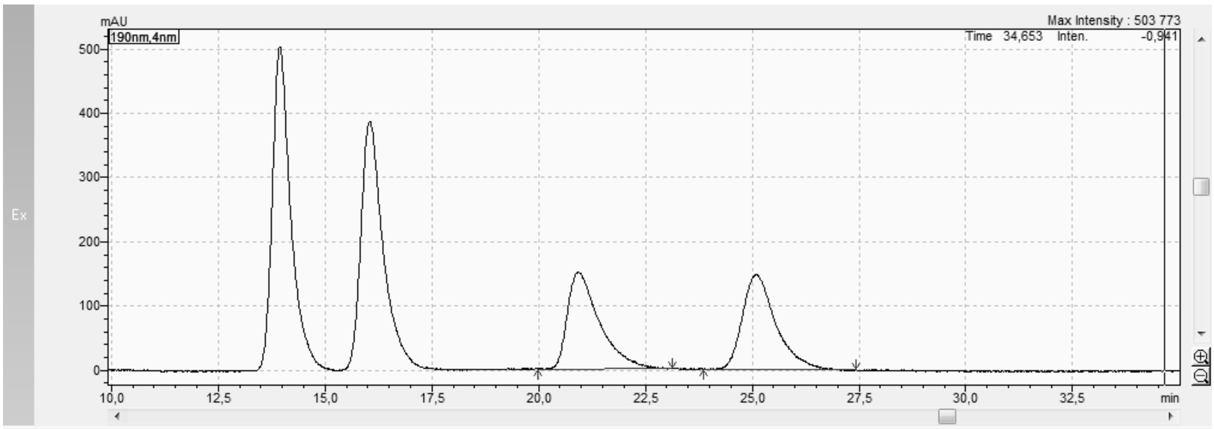

Results View - Peak Table

Peak Table | Compound | Group | Calibration Curve

Peak#	Ret. Time	Conc.	Area	Height	Similarity Index	Mark	Peak Start	Peak End	Area%
1	20,936	49,52446	7801639	150382	0,000000	M	19,979	23,125	49,524
2	25,065	50,47554	7951464	147408	0,000000	M	23,861	27,424	50,476
Total		100,00000	15753103	297790					100,000

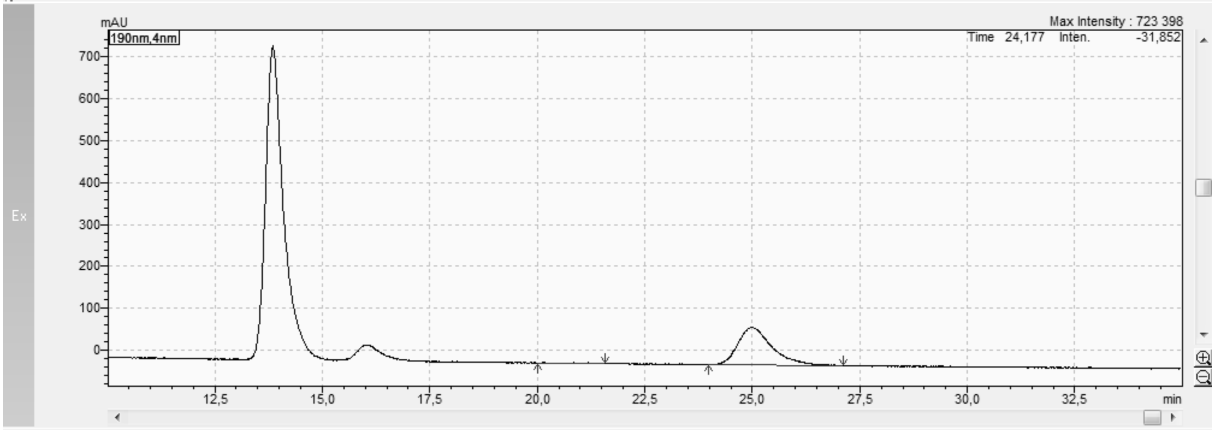

Results View - Peak Table

Peak Table | Compound | Group | Calibration Curve

Peak#	Ret. Time	Conc.	Area	Height	Similarity Index	Mark	Peak Start	Peak End	Area%
1	20,108	0,65497	32764	1062	0,000000	M	20,011	21,579	0,655
2	24,975	99,34503	4969640	90050	0,000000	M	23,979	27,115	99,345
Total		100,00000	5002405	91112					100,000

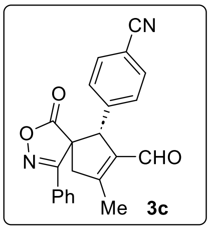

Column: IA column
mobile phase: *n*-heptane / propan-2-ol= 80:20
λ= 197 nm, V= 1 ml/min, t= 25 °C
t_R= 11.8 min (major), t_R= 23.7 min (minor), ee= 87 %

major diastereoisomer

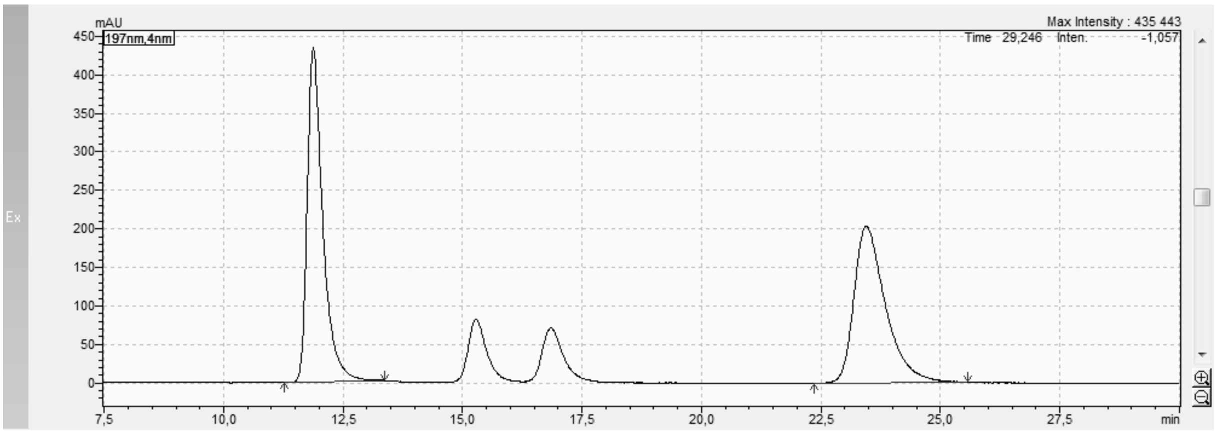

Results View - Peak Table

Peak Table | Compound | Group | Calibration Curve

Peak#	Ret. Time	Conc.	Area	Height	Similarity Index	Mark	Peak Start	Peak End	Area%
1	11,867	51,23068	9820502	433972	0,000000	M	11,275	13,365	51,231
2	23,436	48,76932	9348680	203799	0,000000	M	22,347	25,579	48,769
Total		100,00000	19169182	637771					100,000

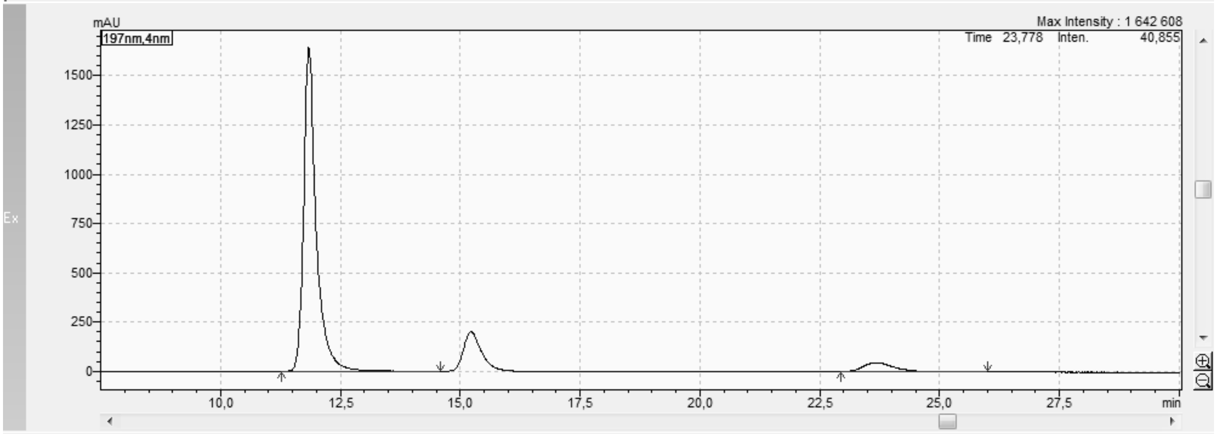

Results View - Peak Table

Peak Table | Compound | Group | Calibration Curve

Peak#	Ret. Time	Conc.	Area	Height	Similarity Index	Mark	Peak Start	Peak End	Area%
1	11,838	93,31889	29886077	1642979	0,000000	M	11,264	14,581	93,319
2	23,659	6,68111	2139676	46597	0,000000	M	22,955	26,016	6,681
Total		100,00000	32025754	1689576					100,000

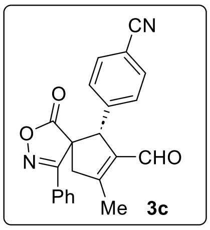

Column: IA column
mobile phase: *n*-heptane / propan-2-ol= 80:20
λ= 197 nm, V= 1 ml/min, t= 25 °C
t_R= 15.2 min (major), t_R= 17.0 min (minor), ee= 99 %

minor diastereoisomer

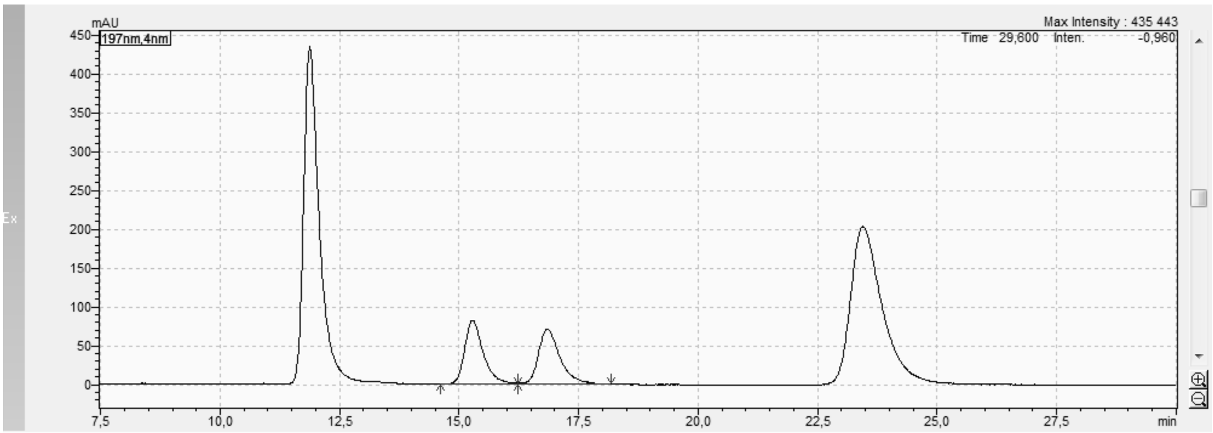

Results View - Peak Table

Peak Table | Compound | Group | Calibration Curve

Peak#	Ret. Time	Conc.	Area	Height	Similarity Index	Mark	Peak Start	Peak End	Area%
1	15,272	49,91991	2209601	81780	0,000000	M	14,613	16,235	49,920
2	16,849	50,08009	2216691	70764	0,000000	V M	16,235	18,176	50,080
Total		100,00000	4426291	152544					100,000

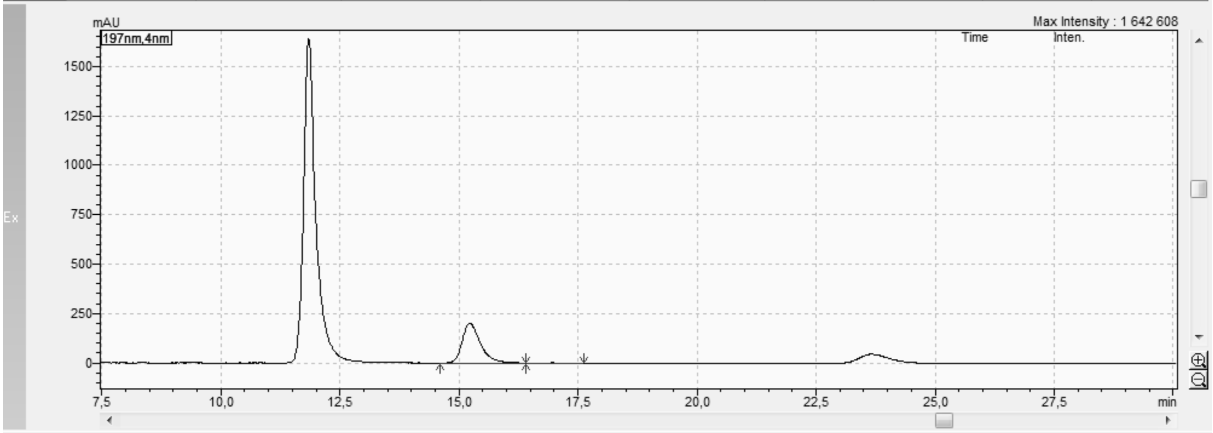

Results View - Peak Table

Peak Table | Compound | Group | Calibration Curve

Peak#	Ret. Time	Conc.	Area	Height	Similarity Index	Mark	Peak Start	Peak End	Area%
1	15,223	99,48762	5311968	200716	0,000000	M	14,613	16,405	99,488
2	16,976	0,51238	27358	1414	0,000000	M	16,405	17,621	0,512
Total		100,00000	5339326	202130					100,000

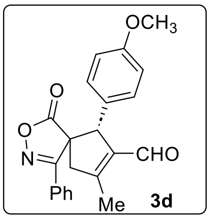

Column Lux-amylose column
mobile phase: *n*-heptane / propan-2-ol= 80:20
λ= 254 nm, V= 1.0 ml/min, t= 25 °C
t_R= 14.6 min (major), t_R= 26.4 min (minor), ee= 87 %

major diastereoisomer

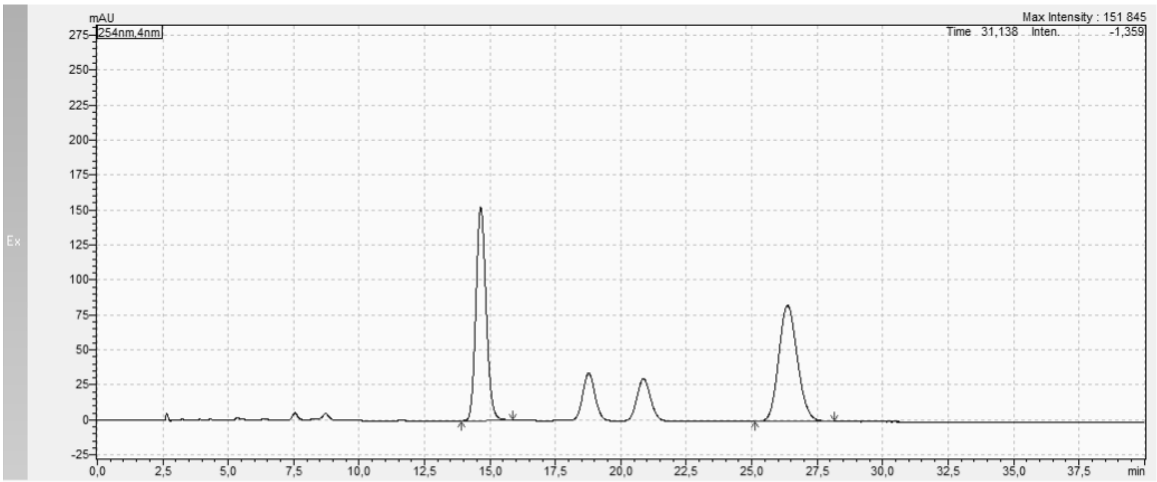

Results View - Peak Table

Peak Table | Compound | Group | Calibration Curve

Peak#	Ret. Time	Area	Height	Peak Start	Peak End	Mark	Conc.	Unit	Area%
1	14,645	4123960	152207	13,909	15,872	M	49,971		49,971
2	26,371	4128684	82898	25,120	28,149	M	50,029		50,029
Total		8252643	235105				100,000		100,000

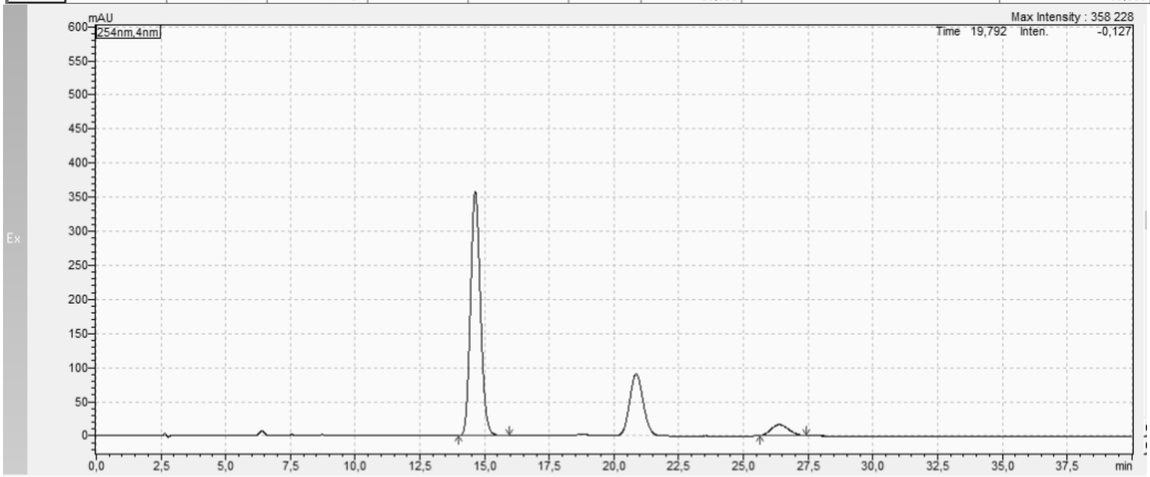

Results View - Peak Table

Peak Table | Compound | Group | Calibration Curve

Peak#	Ret. Time	Area	Height	Peak Start	Peak End	Mark	Conc.	Unit	Area%
1	14,637	9565487	358034	13,973	15,936	M	92,731		92,731
2	26,394	749819	15897	25,643	27,424	M	7,269		7,269
Total		10315306	373931				100,000		100,000

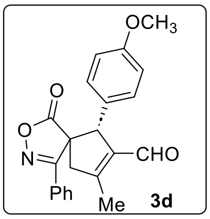

Column: Lux-amylose column
mobile phase: *n*-heptane / propan-2-ol= 80:20
λ= 254 nm, V= 1.0 ml/min, t= 25 °C
t_R= 18.8 min (minor), t_R= 20.9 min (major), ee= 96 %

minor diastereoisomer

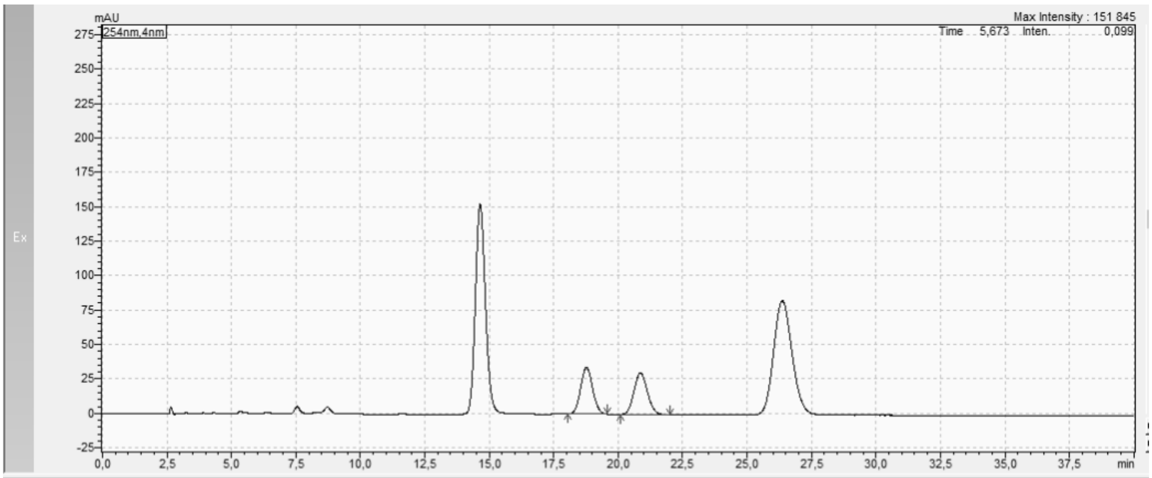

Results View - Peak Table

Peak Table | Compound | Group | Calibration Curve

Peak#	Ret. Time	Area	Height	Peak Start	Peak End	Mark	Conc.	Unit	Area%
1	18,767	1083029	33665	18,027	19,563	M	49,698		49,698
2	20,863	1096209	30164	20,107	22,016	M	50,302		50,302
Total		2179238	63828				100,000		100,000

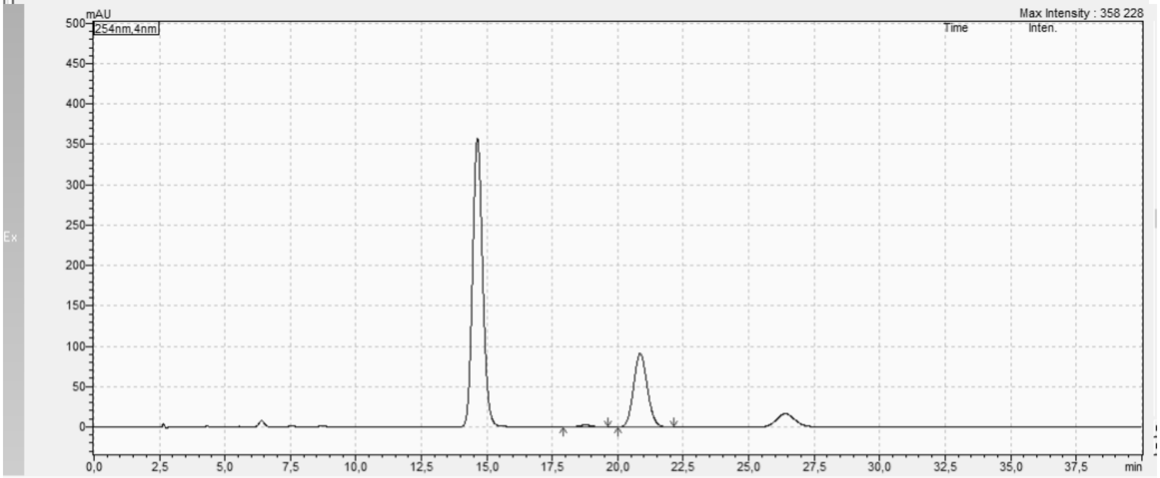

Results View - Peak Table

Peak Table | Compound | Group | Calibration Curve

Peak#	Ret. Time	Area	Height	Peak Start	Peak End	Mark	Conc.	Unit	Area%
1	18,757	61995	2171	17,931	19,605	M	1,836		1,836
2	20,855	3314512	90918	20,000	22,123	M	98,164		98,164
Total		3376507	93089				100,000		100,000

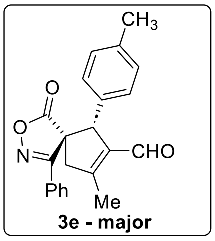

3e - major

Column: IB column
mobile phase: *n*-heptane / propan-2-ol= 80:20
λ= 254 nm, *V*= 1.0 ml/min, *t*= 25 °C
t_R= 14.0 min (major), t_R= 16.6 min (minor), ee= 85 %

major diastereoisomer

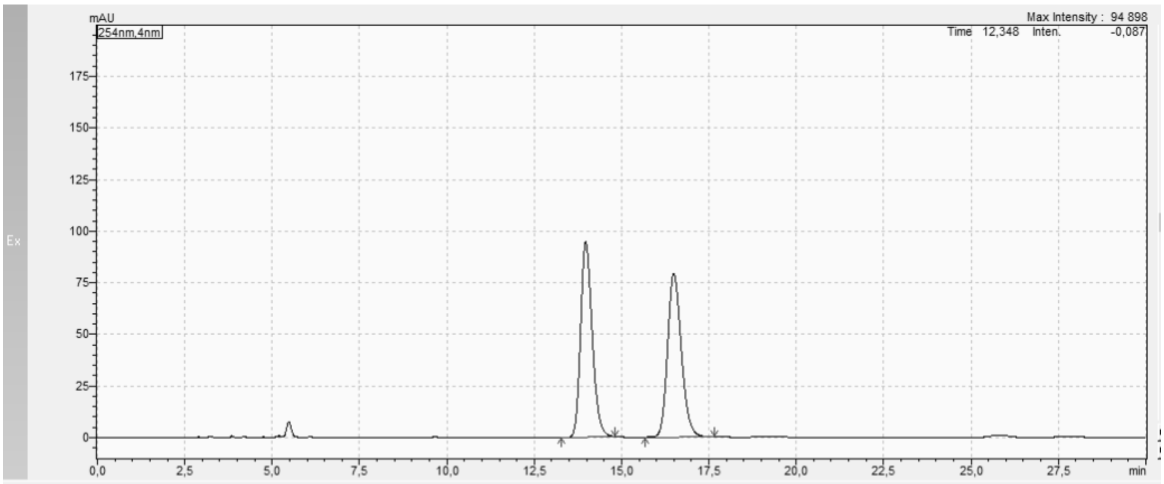

Results View - Peak Table

Peak Table | Compound | Group | Calibration Curve

Peak#	Ret. Time	Area	Height	Peak Start	Peak End	Mark	Conc.	Unit	Area%
1	13,981	2182581	94662	13,291	14,805	M	50,038		50,038
2	16,493	2179268	79329	15,659	17,643	M	49,962		49,962
Total		4361849	173991				100,000		100,000

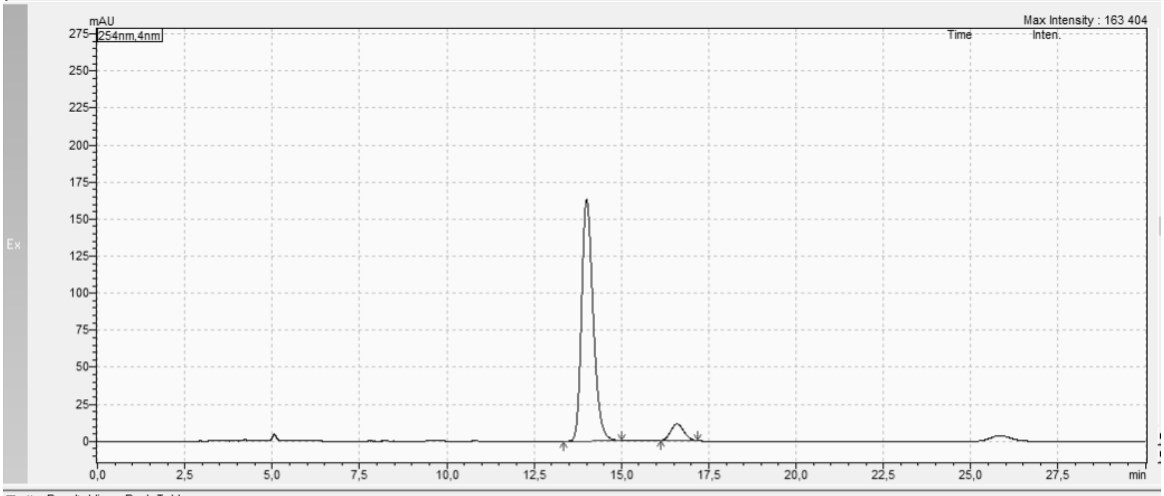

Results View - Peak Table

Peak Table | Compound | Group | Calibration Curve

Peak#	Ret. Time	Area	Height	Peak Start	Peak End	Mark	Conc.	Unit	Area%
1	14,006	3759203	163140	13,333	15,008	M	92,705		92,705
2	16,590	295798	11277	16,128	17,184	M	7,295		7,295
Total		4055001	174418				100,000		100,000

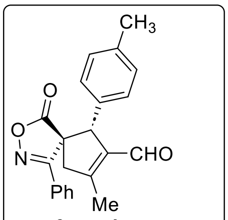

3e - minor

Column: IB column
mobile phase: *n*-heptane / propan-2-ol= 80:20
λ= 254 nm, *V*= 1.0 ml/min, *t*= 25 °C
t_R= 14.0 min (minor), t_R= 25.6 min (major), ee= 96%

minor diastereoisomer

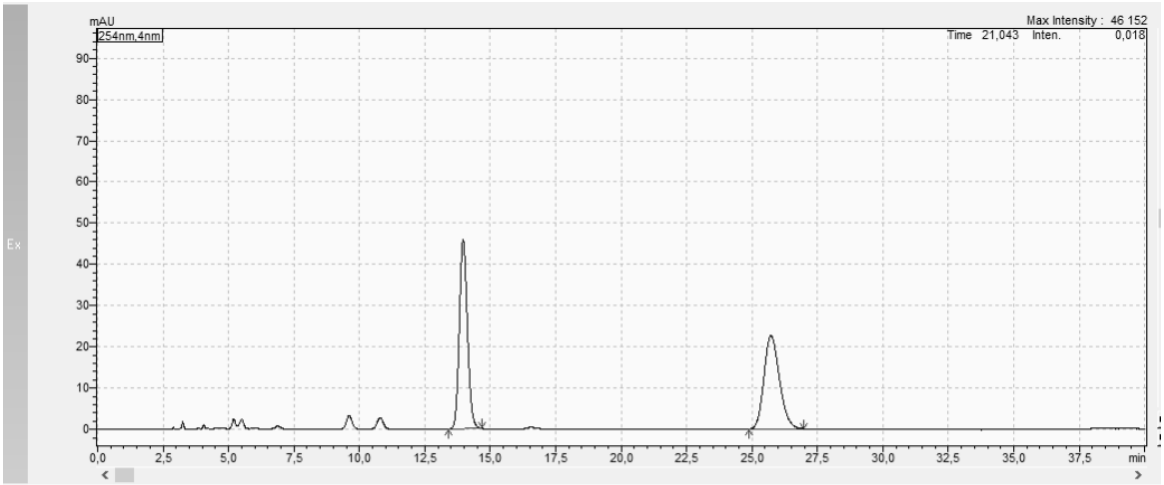

Results View - Peak Table

Peak Table | Compound | Group | Calibration Curve

Peak#	Ret. Time	Area	Height	Peak Start	Peak End	Mark	Conc.	Unit	Area%
1	13,962	983567	45973	13,397	14,667	M	51,317		51,317
2	25,724	933081	22672	24,885	26,955	M	48,683		48,683
Total		1916649	68644				100,000		100,000

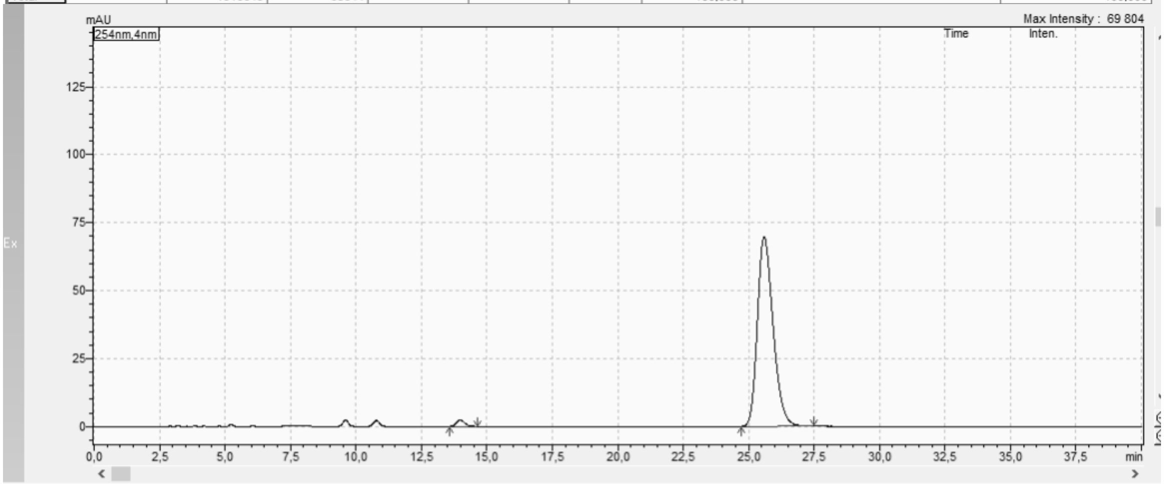

Results View - Peak Table

Peak Table | Compound | Group | Calibration Curve

Peak#	Ret. Time	Area	Height	Peak Start	Peak End	Mark	Conc.	Unit	Area%
1	13,992	50744	2306	13,579	14,645	M	1,717		1,717
2	25,595	2904810	69733	24,725	27,477	M	98,283		98,283
Total		2955553	72039				100,000		100,000

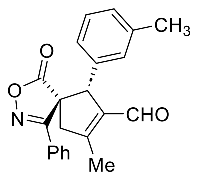

3f - major

Column: IA column
mobile phase: *n*-heptane / propan-2-ol= 80:20
λ= 190 nm, V= 0.5 ml/min, t= 25 °C
t_R= 13.7 min (major), t_R= 14.8 min (minor), ee= 85 %

major diastereoisomer

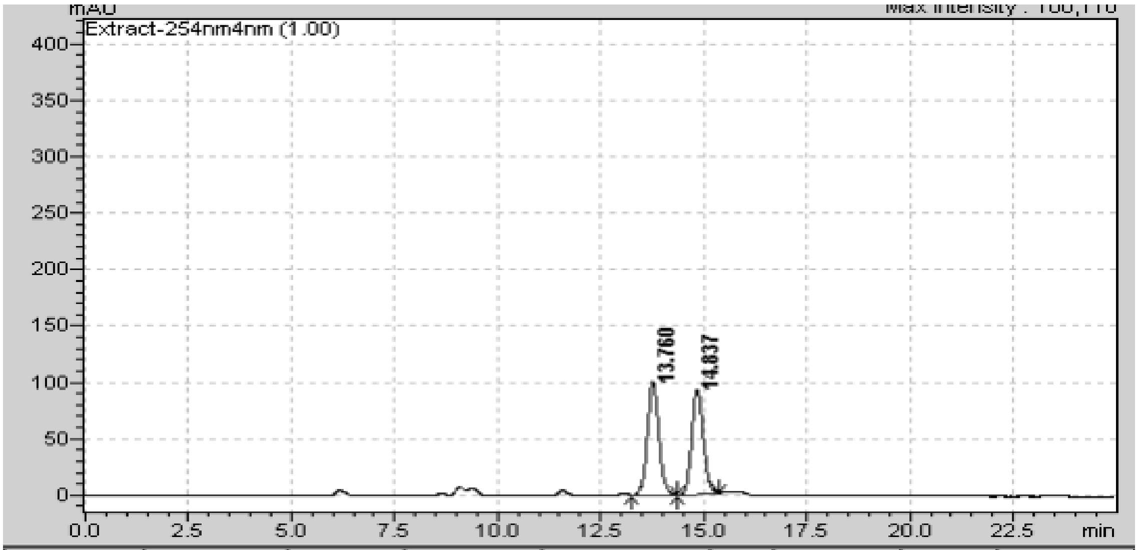

Ret. Time	Conc.	Area	Height	Similarity Inde	Mar	Peak Start	Peak En	Area%
13.760	50.07337	1920241	99830	0.000000		13.269	14.379	50.0734
14.837	49.92663	1914614	94748	0.000000		14.432	15.349	49.9266

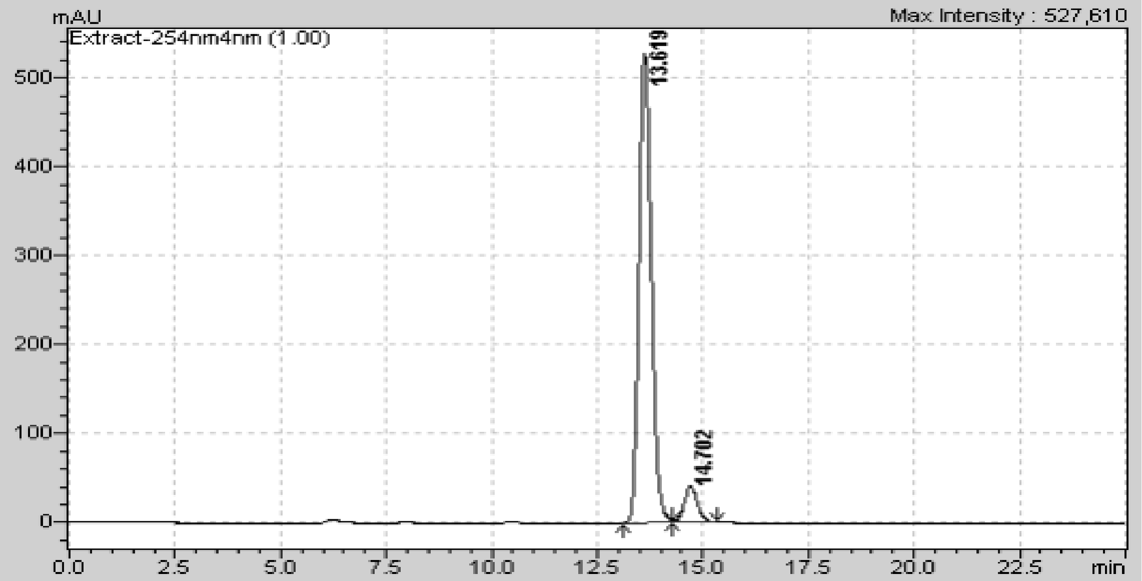

Ret. Time	Conc.	Area	Height	Similarity Inde	Mar	Peak Start	Peak En	Area%
13.619	92.43482	10706057	528115	0.000000		13.099	14.293	92.4348
14.702	7.56518	876221	40744	0.000000	V	14.293	15.317	7.5652

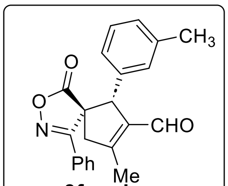

3f - minor

Column: IC column
mobile phase: *n*-heptane / propan-2-ol= 80:20
λ= 190 nm, V= 1 ml/min, t= 25 °C
t_R= 31.0 min (minor), t_R= 34.0 min (major), ee= 98 %

minor diastereoisomer

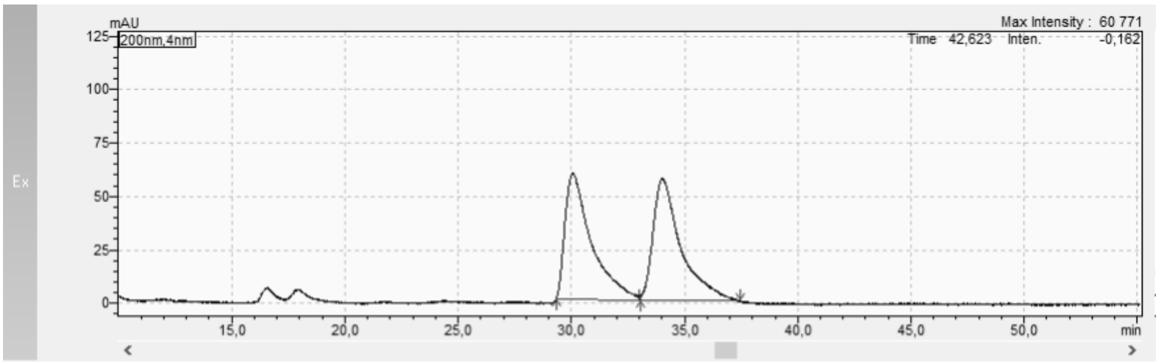

Results View - Peak Table

Peak Table | Compound | Group | Calibration Curve

Peak#	Ret. Time	Area	Height	Peak Start	Peak End	Mark	Conc.	Unit	Area%
1	30,051	4780429	58686	29,333	32,981	M	49,691		49,691
2	34,021	4839800	56931	33,035	37,440	M	50,309		50,309
Total		9620229	115618				100,000		100,000

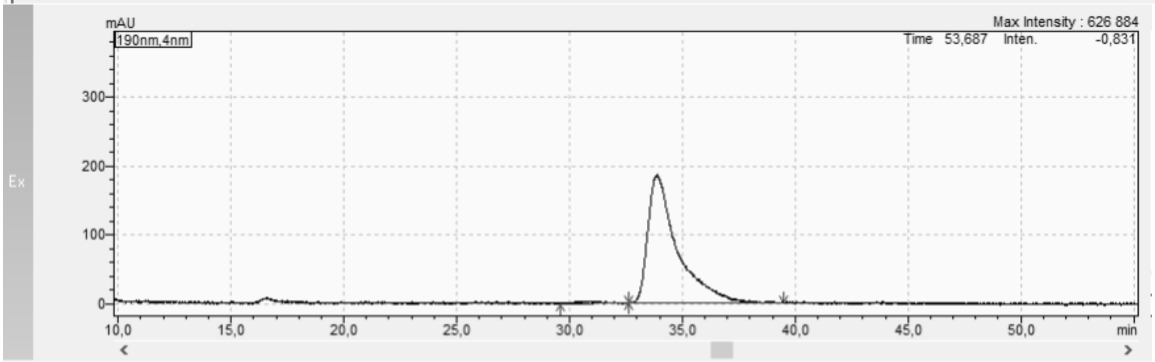

Results View - Peak Table

Peak Table | Compound | Group | Calibration Curve

Peak#	Ret. Time	Area	Height	Peak Start	Peak End	Mark	Conc.	Unit	Area%
1	30,979	164605	3105	29,621	32,608	M	0,969		0,969
2	33,882	16823651	185588	32,608	39,467	M	99,031		99,031
Total		16988256	188693				100,000		100,000

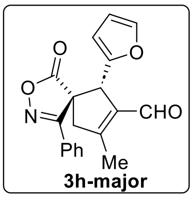

3h-major

Column: IB column
mobile phase: *n*-heptane / propan-2-ol= 80:20
λ= 200 nm, *V*= 1 ml/min, *t*= 25 °C
t_R= 17.5 min (major), t_R= 21.5 min (minor), ee= 78%

major diastereoisomer

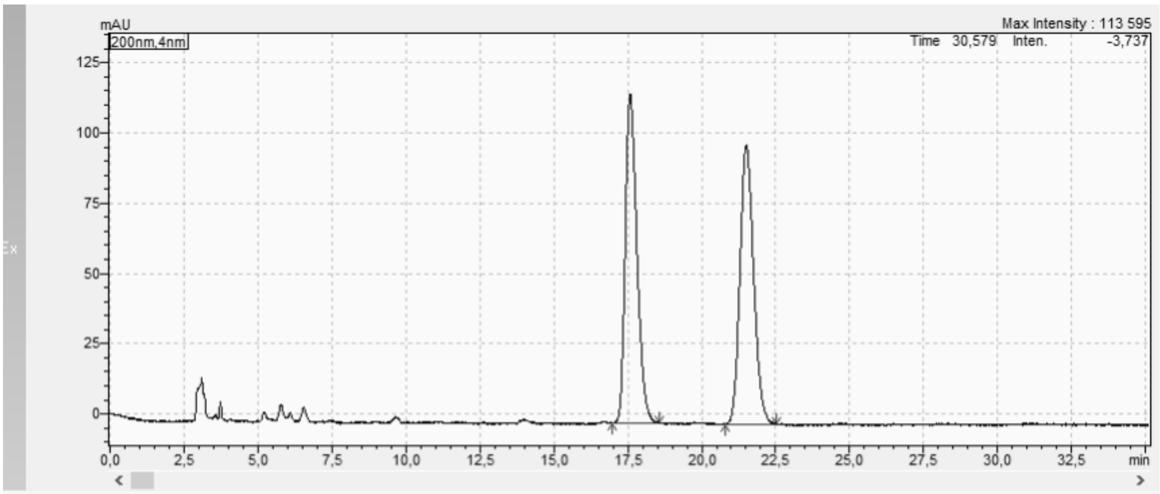

Results View - Peak Table

Peak Table | Compound | Group | Calibration Curve

Peak#	Ret. Time	Area	Height	Peak Start	Peak End	Mark	Conc.	Unit	Area%
1	17,583	3204939	116858	16,971	18,571	M	50,003		50,003
2	21,515	3204582	99167	20,800	22,528	M	49,997		49,997
Total		6409522	216024				100,000		100,000

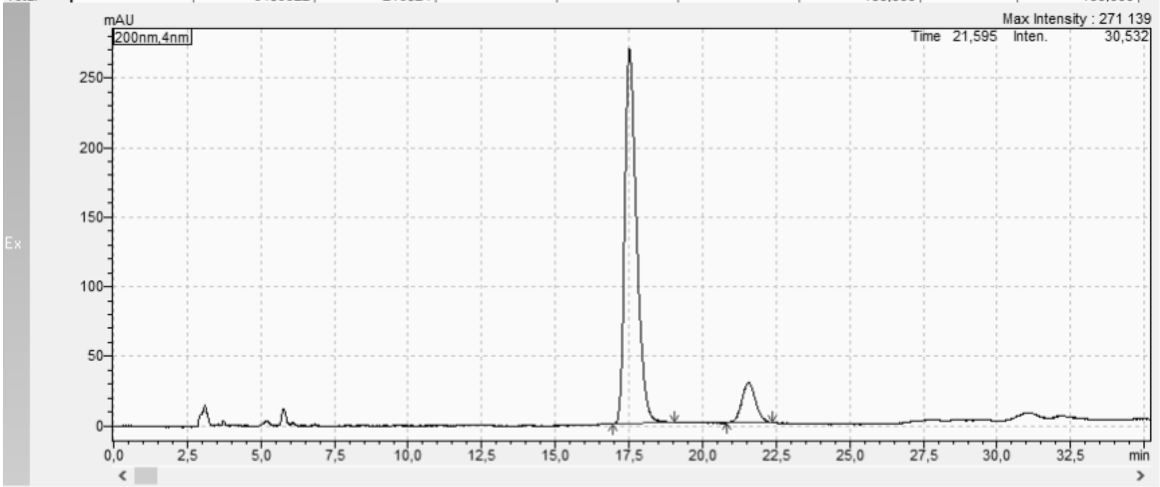

Results View - Peak Table

Peak Table | Compound | Group | Calibration Curve

Peak#	Ret. Time	Area	Height	Peak Start	Peak End	Mark	Conc.	Unit	Area%
1	17,516	7508260	269338	16,971	19,029	M	89,055		89,055
2	21,548	922770	28607	20,821	22,389	M	10,945		10,945
Total		8431031	297945				100,000		100,000

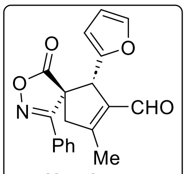

3h-minor

Column: IB column
mobile phase: *n*-heptane / propan-2-ol= 80:20
λ= 200 nm, V= 1 ml/min, t= 25 °C
t_R= 16.7 min (minor), t_R= 31.0 min (major), ee= 87%

minor diastereoisomer

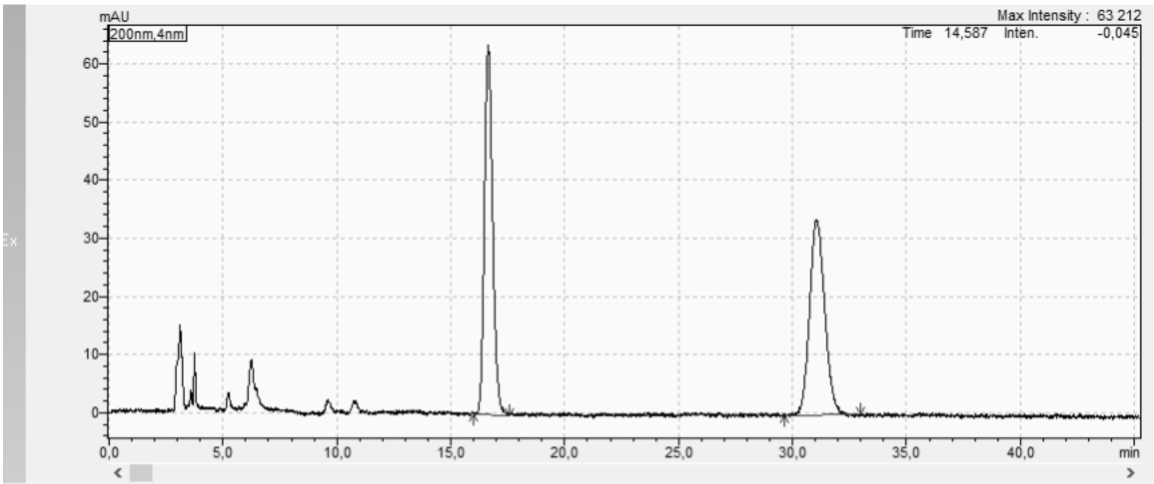

Results View - Peak Table

Peak Table | Compound | Group | Calibration Curve

Peak#	Ret. Time	Area	Height	Peak Start	Peak End	Mark	Conc.	Unit	Area%
1	16,647	1590781	63574	15,968	17,557	M	50,562		50,562
2	31,068	1555425	33710	29,664	32,971	M	49,438		49,438
Total		3146205	97285				100,000		100,000

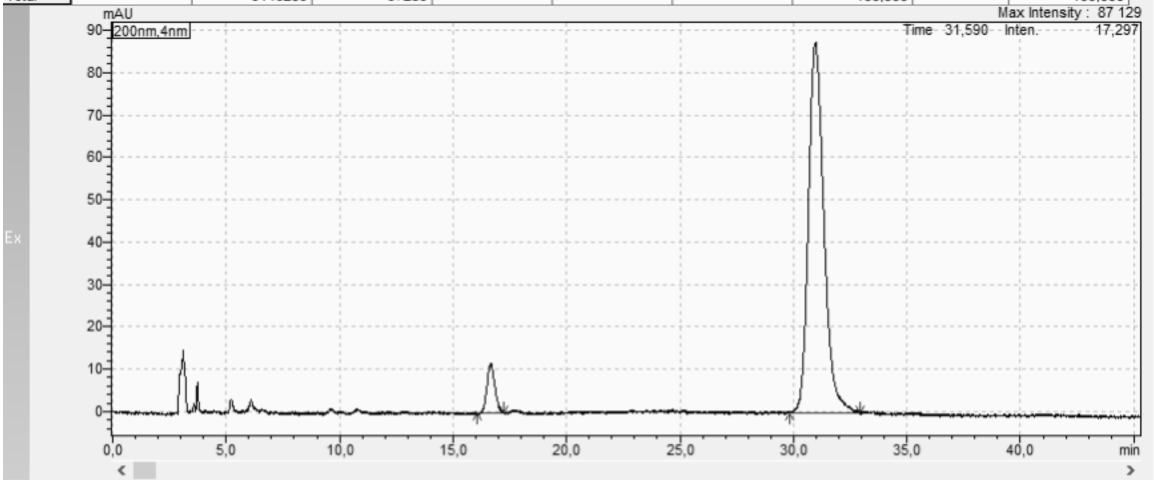

Results View - Peak Table

Peak Table | Compound | Group | Calibration Curve

Peak#	Ret. Time	Area	Height	Peak Start	Peak End	Mark	Conc.	Unit	Area%
1	16,676	277907	11567	16,085	17,205	M	6,251		6,251
2	30,973	4167631	87593	29,813	32,960	M	93,749		93,749
Total		4445538	99160				100,000		100,000

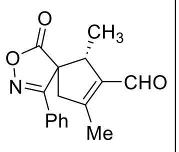

3i

Column: IC column
mobile phase: *n*-heptane / propan-2-ol= 90:10
λ= 190 nm, V= 1 ml/min, t= 25 °C
t_R= 26.2 min (minor), t_R= 29.6 min (major), ee= 70 %

major diastereoisomer

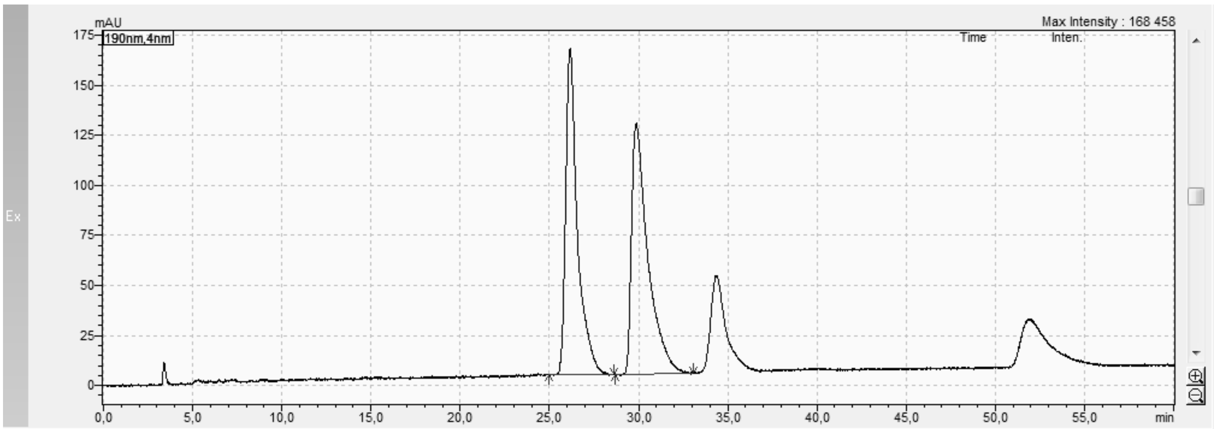

Results View - Peak Table

Peak Table | Compound | Group | Calibration Curve

Peak#	Ret. Time	Conc.	Area	Height	Similarity Index	Mark	Peak Start	Peak End	Area%
1	26,155	48,41965	7431876	162958	0,000000	M	24,971	28,619	48,420
2	29,864	51,58035	7917008	125149	0,000000	M	28,683	33,067	51,580
Total		100,00000	15348884	288107					100,000

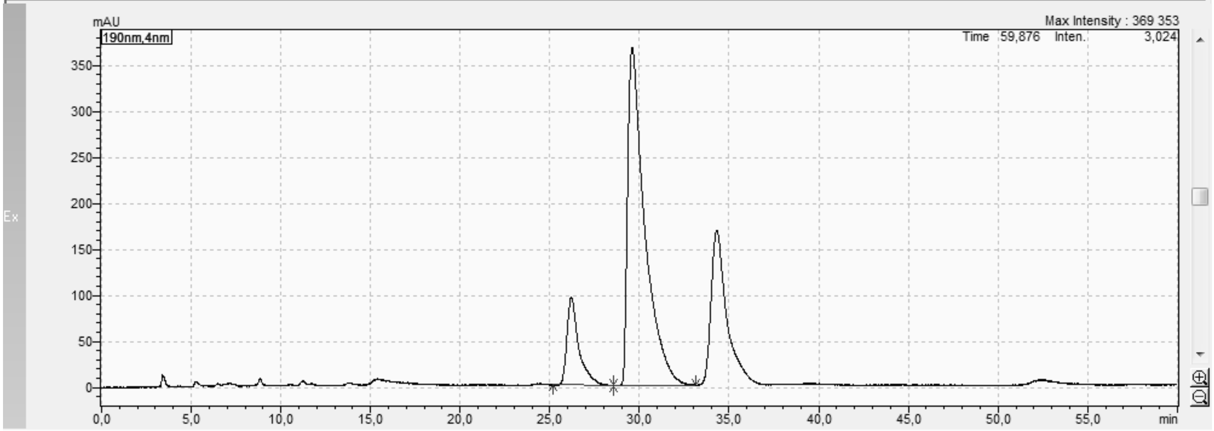

Results View - Peak Table

Peak Table | Compound | Group | Calibration Curve

Peak#	Ret. Time	Conc.	Area	Height	Similarity Index	Mark	Peak Start	Peak End	Area%
1	26,193	15,20139	4354646	95345	0,000000	M	25,184	28,555	15,201
2	29,584	84,79861	24291719	367055	0,000000	M	28,555	33,141	84,799
Total		100,00000	28646365	462399					100,000

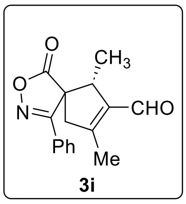

3i

Column: IC column
mobile phase: *n*-heptane / propan-2-ol= 90:10
λ= 190 nm, V= 1 ml/min, t= 25 °C
t_R= 34.3 min (major), t_R= 52.5 min (minor), ee= 90 %

minor diastereoisomer

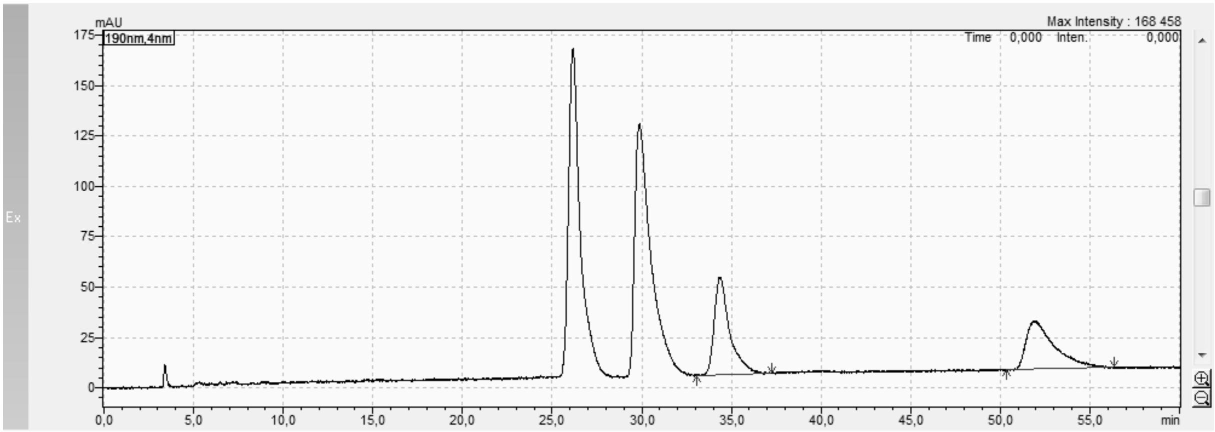

Results View - Peak Table

Peak Table | Compound | Group | Calibration Curve

Peak#	Ret. Time	Conc.	Area	Height	Similarity Index	Mark	Peak Start	Peak End	Area%
1	34,352	52,13741	2870021	48149	0,000000	M	33,067	37,259	52,137
2	51,888	47,86259	2634703	23959	0,000000	M	50,347	56,352	47,863
Total		100,00000	5504724	72108					100,000

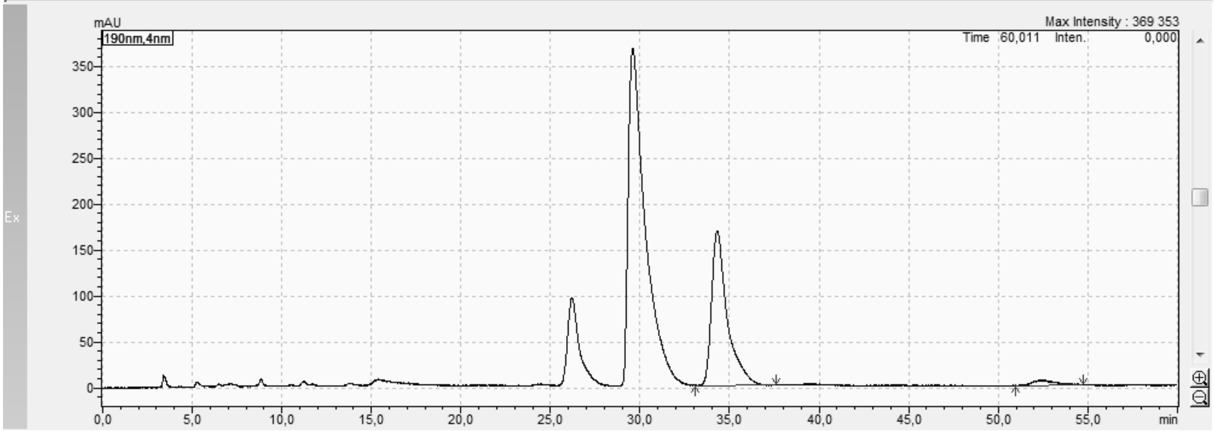

Results View - Peak Table

Peak Table | Compound | Group | Calibration Curve

Peak#	Ret. Time	Conc.	Area	Height	Similarity Index	Mark	Peak Start	Peak End	Area%
1	34,330	94,94198	10139175	168030	0,000000	M	33,077	37,600	94,942
2	52,469	5,05802	540163	5718	0,000000	M	50,965	54,741	5,058
Total		100,00000	10679338	173748					100,000

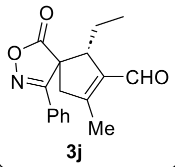

3j

Column: IB column
mobile phase: *n*-heptane / propan-2-ol= 80:20
λ= 200 nm, V= 0.5 ml/min, t= 25 °C
t_R= 19.1 min (major), t_R= 22.1 min (minor), ee= 76 %

major diastereoisomer

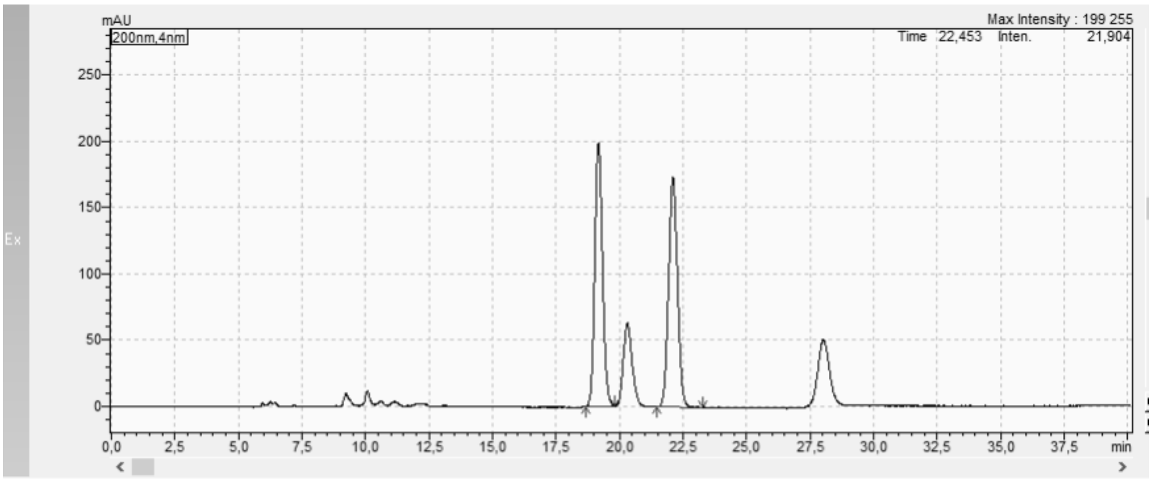

Results View - Peak Table

Peak Table | Compound | Group | Calibration Curve

Peak#	Ret. Time	Area	Height	Peak Start	Peak End	Mark	Conc.	Unit	Area%
1	19,172	4227122	199415	18,656	19,840	M	49,862		49,862
2	22,108	4250590	173946	21,461	23,264	M	50,138		50,138
Total		8477712	373362				100,000		100,000

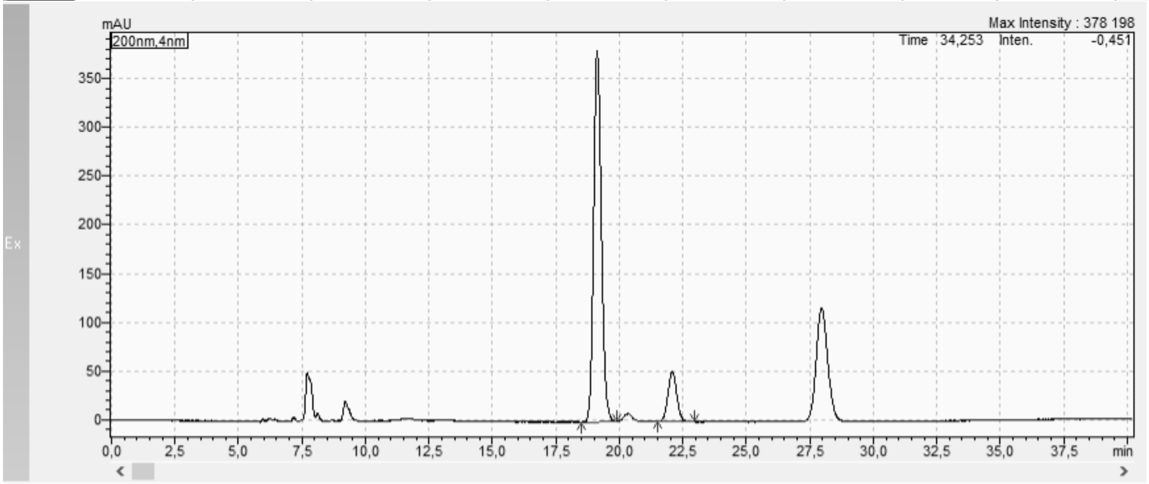

Results View - Peak Table

Peak Table | Compound | Group | Calibration Curve

Peak#	Ret. Time	Area	Height	Peak Start	Peak End	Mark	Conc.	Unit	Area%
1	19,125	8069541	380553	18,507	19,915	M	86,739		86,739
2	22,077	1233725	51058	21,493	22,976	M	13,261		13,261
Total		9303266	431612				100,000		100,000

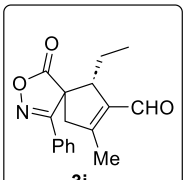

3j

Column: IB column
mobile phase: *n*-heptane / propan-2-ol= 98:2
λ= 200 nm, V= 1 ml/min, t= 25 °C
t_R= 20.3 min (minor), t_R= 28.0 min (major), ee= 90%

minor diastereoisomer

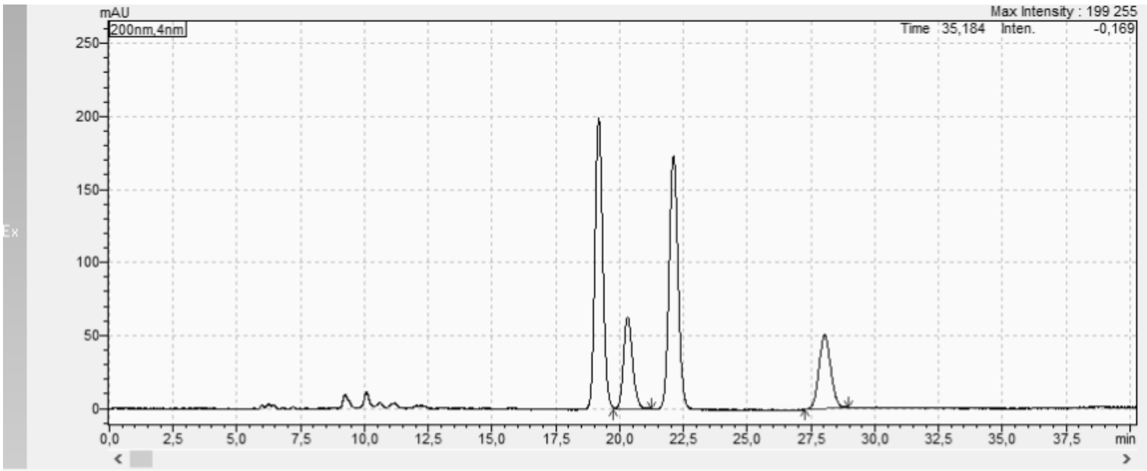

Results View - Peak Table

Peak Table | Compound | Group | Calibration Curve

Peak#	Ret. Time	Area	Height	Peak Start	Peak End	Mark	Conc.	Unit	Area%
1	20,308	1558579	63204	19,744	21,227	M	49,280		49,280
2	28,026	1604099	50446	27,253	28,971	M	50,720		50,720
Total		3162678	113650				100,000		100,000

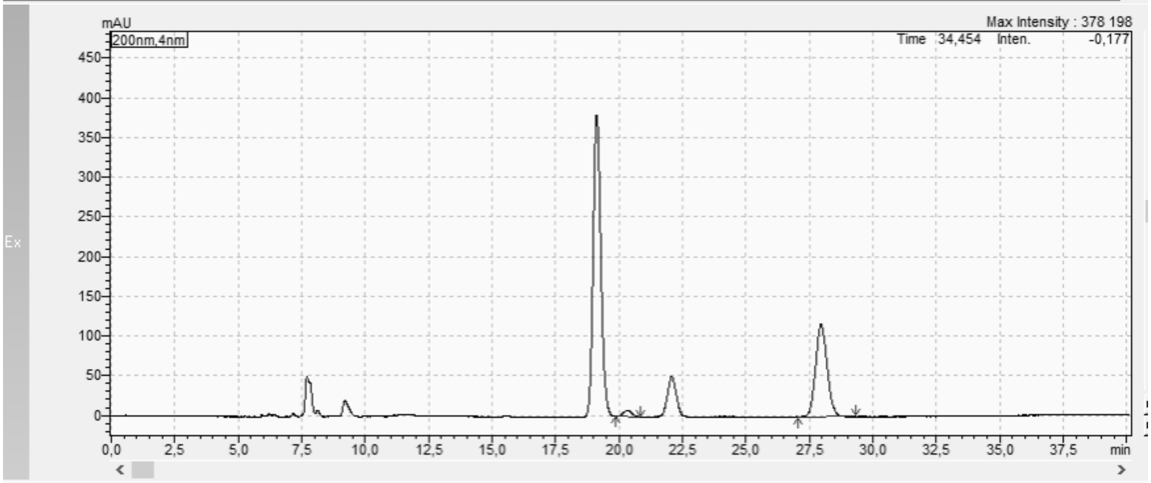

Results View - Peak Table

Peak Table | Compound | Group | Calibration Curve

Peak#	Ret. Time	Area	Height	Peak Start	Peak End	Mark	Conc.	Unit	Area%
1	20,345	180901	7754	19,883	20,843	M	4,680		4,680
2	27,965	3684517	116318	27,040	29,344	M	95,320		95,320
Total		3865418	124072				100,000		100,000

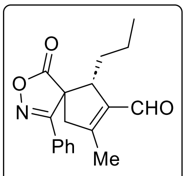

3k

Column: IC column
mobile phase: *n*-heptane / propan-2-ol= 90:10
λ= 200 nm, V= 1 ml/min, t= 25 °C
t_R= 13.1 min (minor), t_R= 16.1 min (major), ee= 74 %

major diastereoisomer

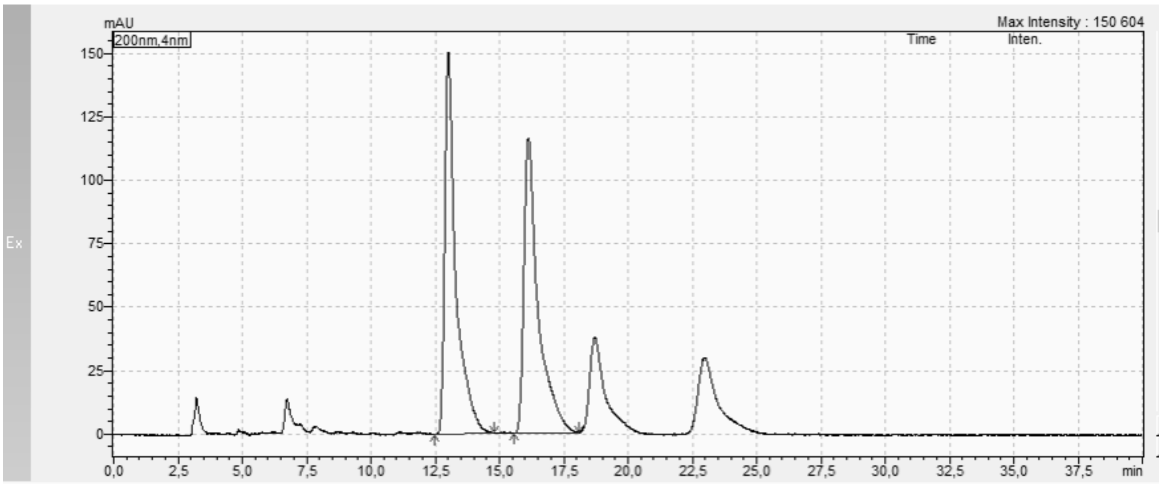

Results View - Peak Table

Peak Table | Compound | Group | Calibration Curve

Peak#	Ret. Time	Area	Height	Peak Start	Peak End	Mark	Conc.	Unit	Area%
1	13,014	4514087	150546	12,469	14,805	M	50,037		50,037
2	16,129	4507494	116502	15,584	18,069	M	49,963		49,963
Total		9021581	267048				100,000		100,000

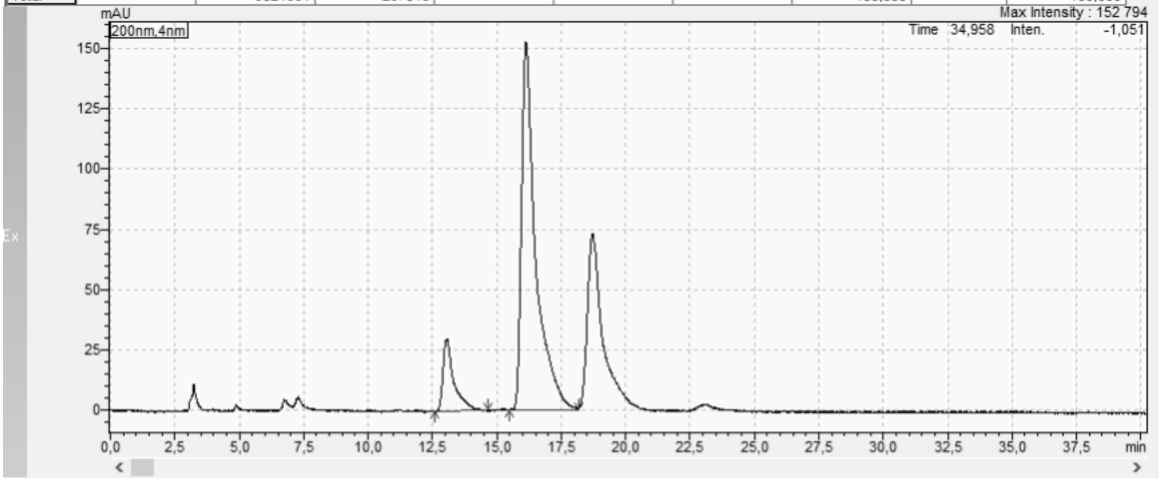

Results View - Peak Table

Peak Table | Compound | Group | Calibration Curve

Peak#	Ret. Time	Area	Height	Peak Start	Peak End	Mark	Conc.	Unit	Area%
1	13,067	896112	29976	12,597	14,656	M	13,309		13,309
2	16,129	5836928	152741	15,509	18,176	M	86,691		86,691
Total		6733040	182717				100,000		100,000

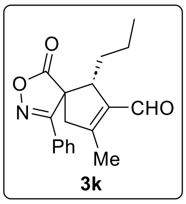

Column: IC column
mobile phase: *n*-heptane / propan-2-ol= 90:10
λ= 200 nm, *V*= 1 ml/min, *t*= 25 °C
t_R= 18.7 min (major), t_R= 23.1 min (minor), ee= 94 %

minor diastereoisomer

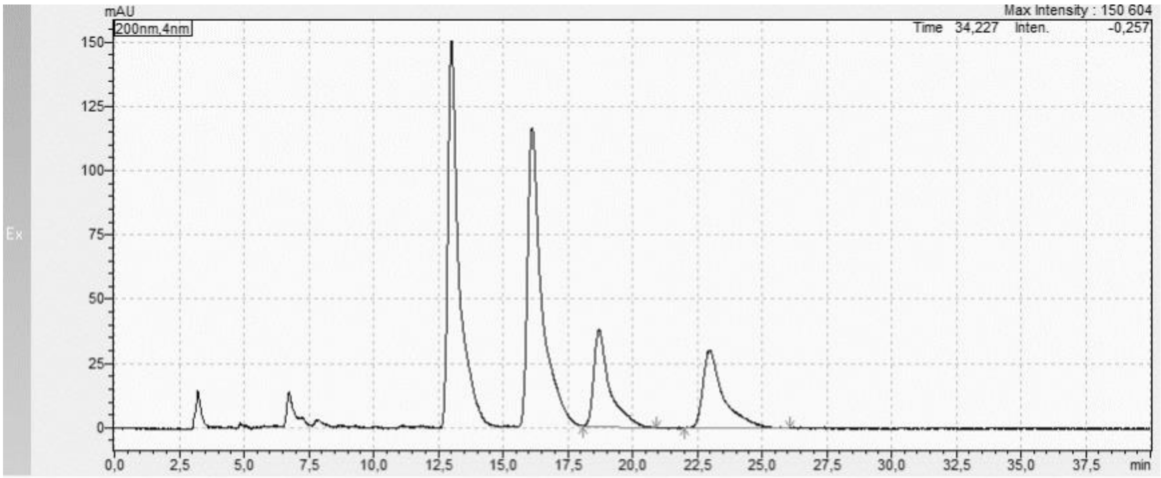

Results View - Peak Table

Peak Table | Compound | Group | Calibration Curve

Peak#	Ret. Time	Area	Height	Peak Start	Peak End	Mark	Conc.	Unit	Area%
1	18,714	1629082	38084	18,069	20,939	M	49,194		49,194
2	22,986	1682460	30470	22,005	26,080	M	50,806		50,806
Total		3311542	68554				100,000		100,000

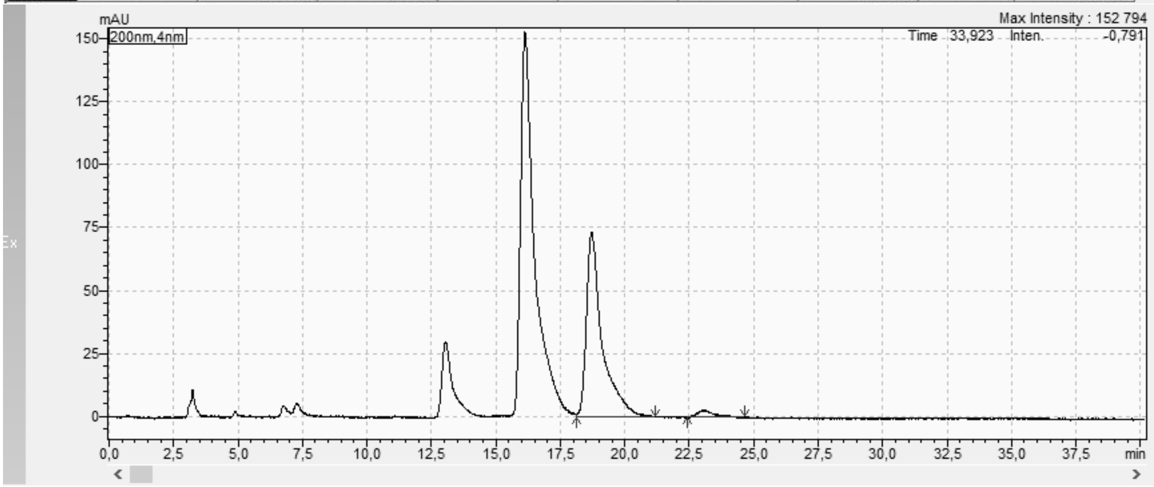

Results View - Peak Table

Peak Table | Compound | Group | Calibration Curve

Peak#	Ret. Time	Area	Height	Peak Start	Peak End	Mark	Conc.	Unit	Area%
1	18,717	3112882	73288	18,112	21,184	M	96,779		96,779
2	23,071	103596	2585	22,411	24,629	M	3,221		3,221
Total		3216478	75873				100,000		100,000

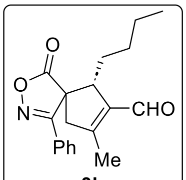

3l

Column: IC column
mobile phase: *n*-heptane / propan-2-ol= 90:10
λ= 200 nm, *V*= 1 ml/min, *t*= 25 °C
t_R= 11.7 min (minor), t_R= 13.9 min (major), ee= 80 %

major diastereoisomer

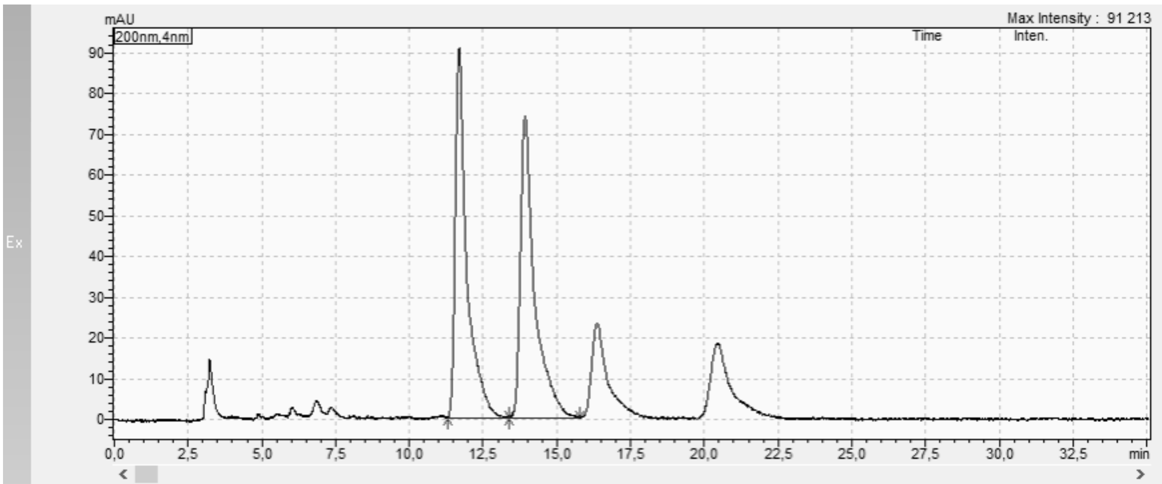

Results View - Peak Table

Peak Table | Compound | Group | Calibration Curve

Peak#	Ret. Time	Area	Height	Peak Start	Peak End	Mark	Conc.	Unit	Area%
1	11,689	2465074	90835	11,296	13,365	M	49,737		49,737
2	13,928	2491183	74169	13,365	15,797	V M	50,263		50,263
Total		4956256	165004				100,000		100,000

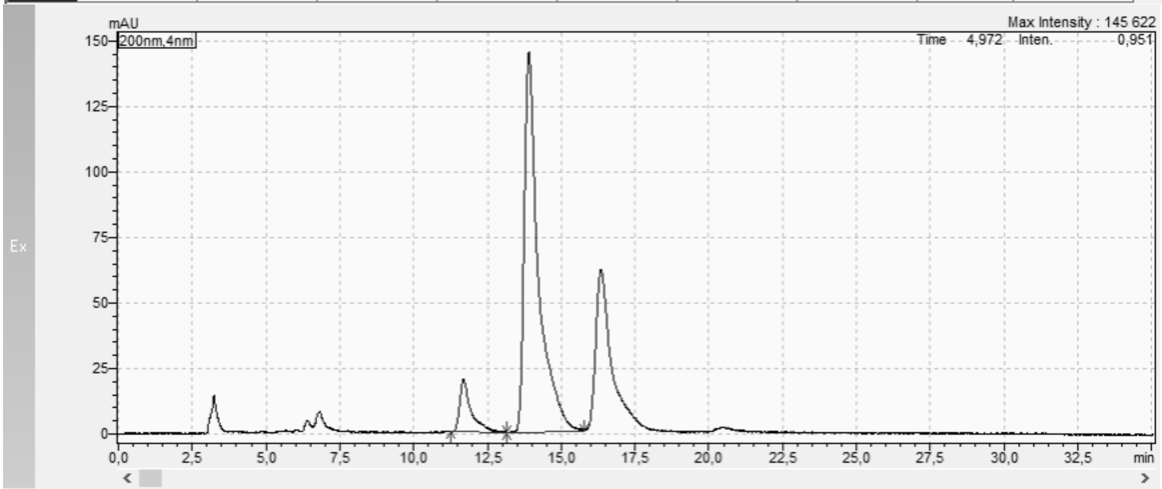

Results View - Peak Table

Peak Table | Compound | Group | Calibration Curve

Peak#	Ret. Time	Area	Height	Peak Start	Peak End	Mark	Conc.	Unit	Area%
1	11,693	543408	20028	11,264	13,173	M	10,056		10,056
2	13,903	4860209	144941	13,173	15,755	M	89,944		89,944
Total		5403618	164968				100,000		100,000

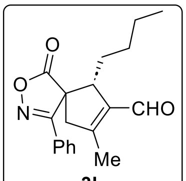

3l

Column: IC column
mobile phase: *n*-heptane / propan-2-ol= 90:10
λ= 200 nm, *V*= 1 ml/min, *t*= 25 °C
t_R= 16.4 min (major), t_R= 20.6 min (minor), ee= 95 %

minor diastereoisomer

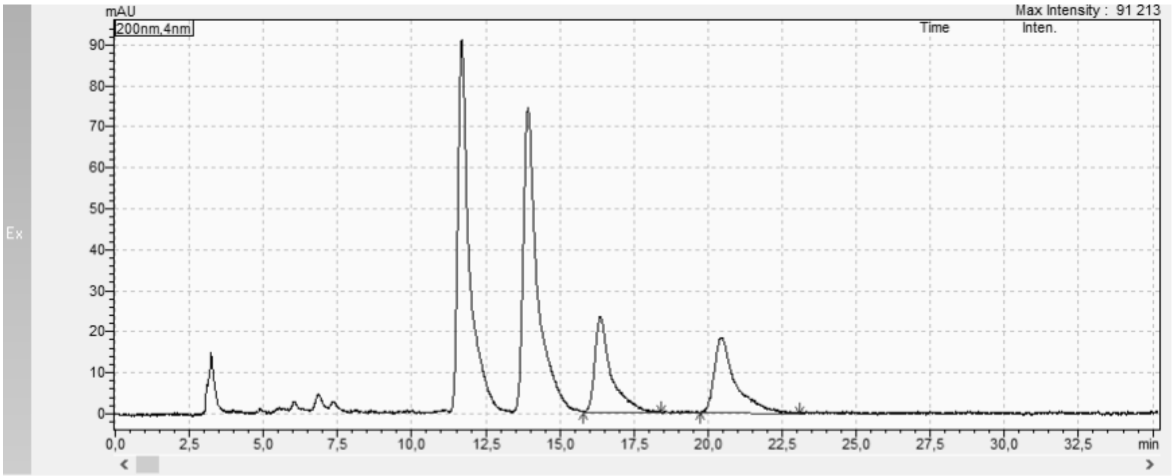

Results View - Peak Table

Peak Table | Compound | Group | Calibration Curve

Peak#	Ret. Time	Area	Height	Peak Start	Peak End	Mark	Conc.	Unit	Area%
1	16,355	892471	23343	15,797	18,400	M	49,362		49,362
2	20,473	915555	18422	19,744	23,104	M	50,638		50,638
Total		1808025	41765				100,000		100,000

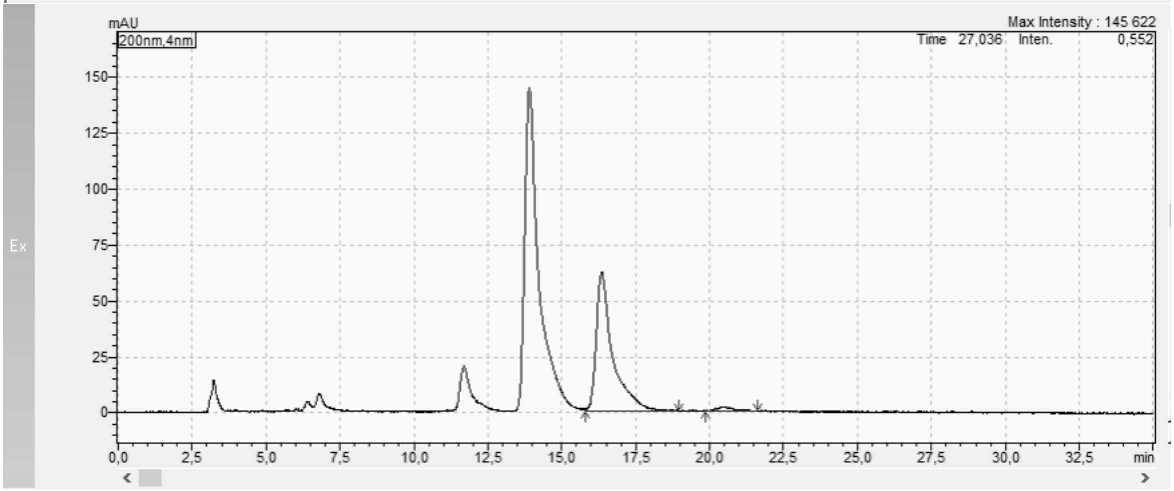

Results View - Peak Table

Peak Table | Compound | Group | Calibration Curve

Peak#	Ret. Time	Area	Height	Peak Start	Peak End	Mark	Conc.	Unit	Area%
1	16,358	2388010	61947	15,808	18,955	M	97,572		97,572
2	20,552	59416	1692	19,872	21,600	M	2,428		2,428
Total		2447426	63640				100,000		100,000

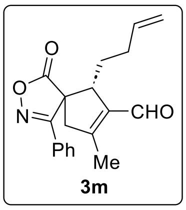

Column: IC column
mobile phase: *n*-heptane / propan-2-ol= 90:10
λ= 190 nm, V= 1 ml/min, t= 25 °C
t_R= 13.5 min (minor), t_R= 16.8 min (major), ee= 74 %

major diastereoisomer

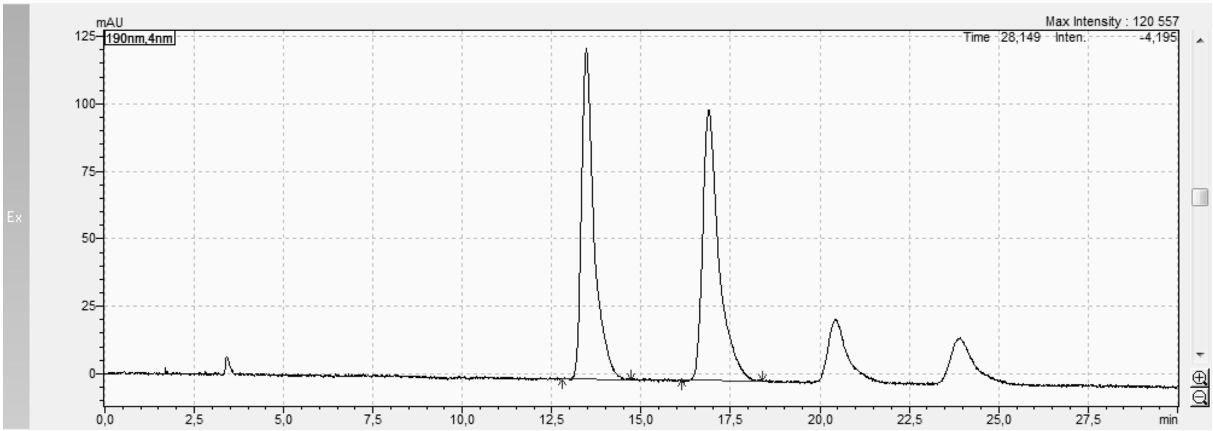

Results View - Peak Table

Peak Table | Compound | Group | Calibration Curve

Peak#	Ret. Time	Conc.	Area	Height	Similarity Index	Mark	Peak Start	Peak End	Area%
1	13,465	48,62825	2983837	122611	0,000000	M	12,789	14,720	48,628
2	16,885	51,37175	3152180	100064	0,000000	M	16,128	18,400	51,372
Total		100,00000	6136017	222675					100,000

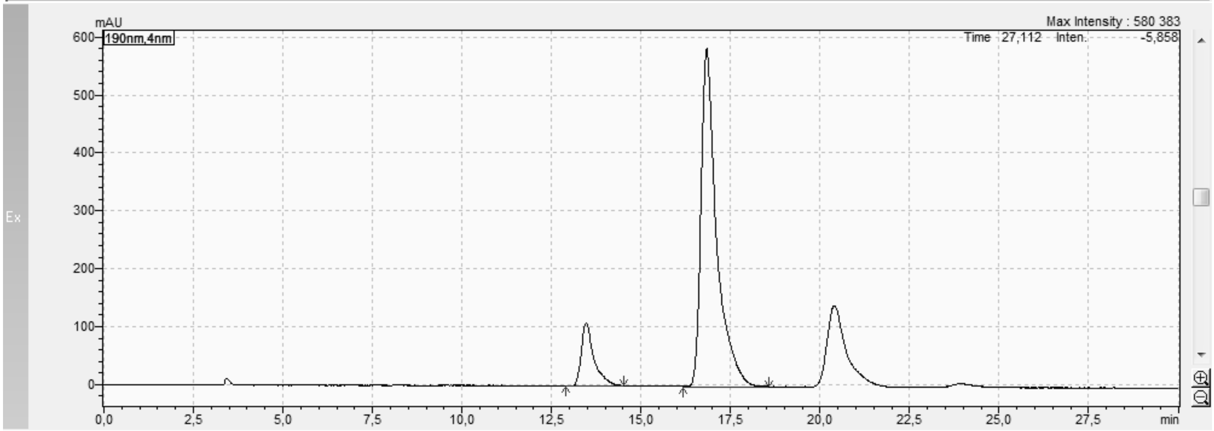

Results View - Peak Table

Peak Table | Compound | Group | Calibration Curve

Peak#	Ret. Time	Conc.	Area	Height	Similarity Index	Mark	Peak Start	Peak End	Area%
1	13,459	13,09472	2657738	108268	0,000000	M	12,896	14,517	13,095
2	16,827	86,90528	17638513	583893	0,000000	M	16,171	18,571	86,905
Total		100,00000	20296251	692162					100,000

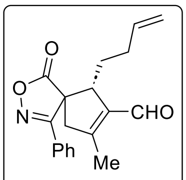

3m

Column: IC column
mobile phase: *n*-heptane / propan-2-ol= 90:10
λ= 190 nm, *V*= 1 ml/min, *t*= 25 °C
t_R= 20.4 min (major), t_R= 23.9 min (minor), ee= 91 %

minor diastereoisomer

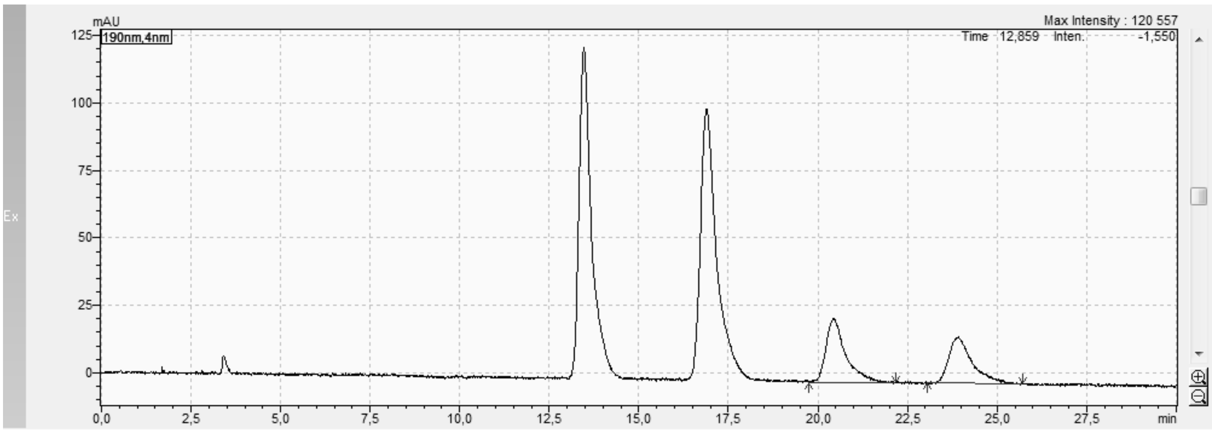

Results View - Peak Table

Peak Table | Compound | Group | Calibration Curve

Peak#	Ret. Time	Conc.	Area	Height	Similarity Index	Mark	Peak Start	Peak End	Area%
1	20,432	54,36784	910455	23705	0,000000	M	19,744	22,176	54,368
2	23,908	45,63216	764166	17162	0,000000	M	23,051	25,717	45,632
Total		100,00000	1674621	40867					100,000

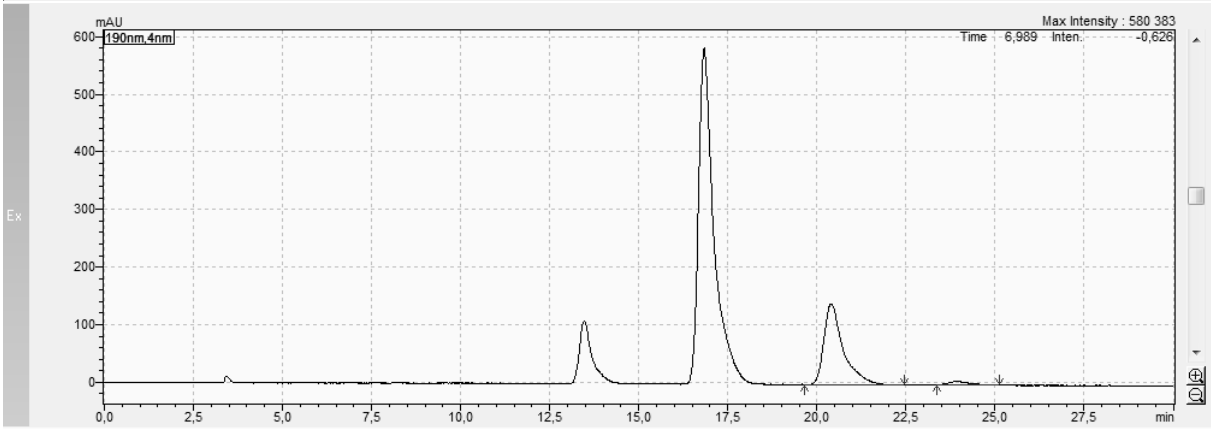

Results View - Peak Table

Peak Table | Compound | Group | Calibration Curve

Peak#	Ret. Time	Conc.	Area	Height	Similarity Index	Mark	Peak Start	Peak End	Area%
1	20,383	95,65563	5309598	140452	0,000000	M	19,648	22,453	95,656
2	23,923	4,34437	241145	6233	0,000000	M	23,360	25,120	4,344
Total		100,00000	5550743	146684					100,000

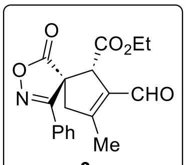

3n

Column: IB column
mobile phase: *n*-heptane / propan-2-ol= 70:30
λ= 254 nm, V= 1 ml/min, t= 25 °C
t_R= 9.6 min (major), t_R= 19.6 min (minor), ee= 80 %

major diastereoisomer

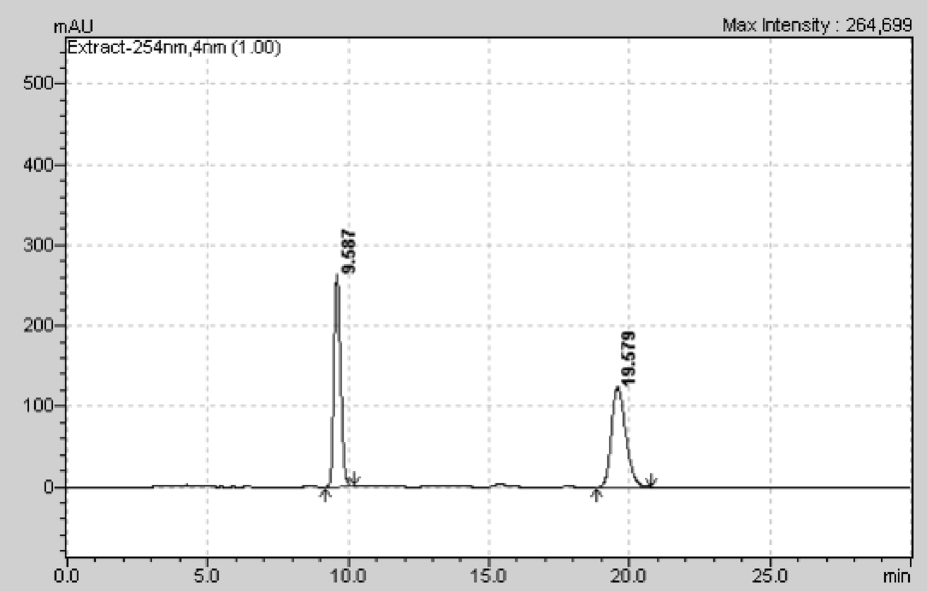

Ret. Time	Conc.	Area	Height	Similarity Inde	Mar	Peak Start	Peak En	Area%
9.587	49.89991	4338224	264512	0.000000		9.163	10.187	49.8999
19.579	50.10009	4355628	124096	0.000000		18.827	20.768	50.1001

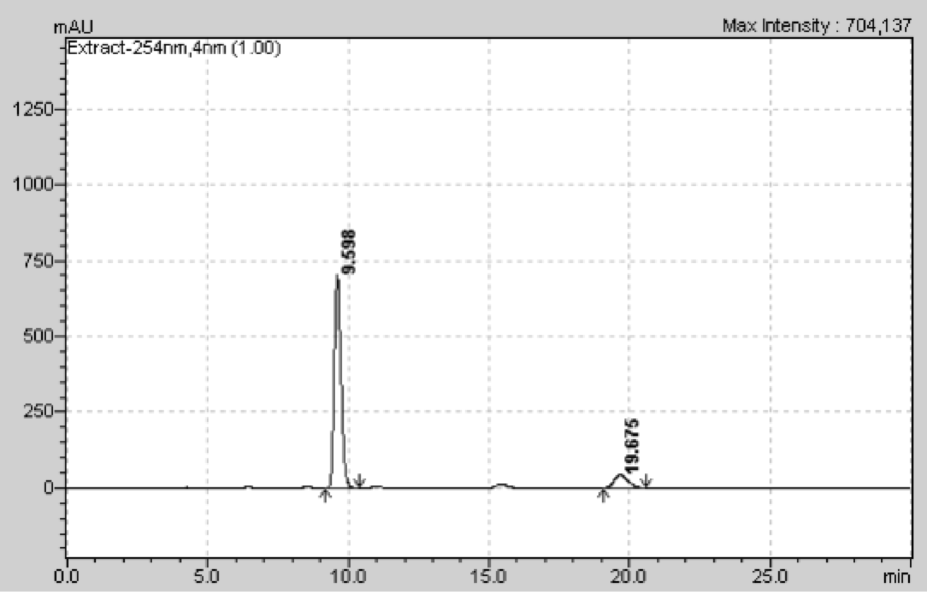

Ret. Time	Conc.	Area	Height	Similarity Inde	Mar	Peak Start	Peak En	Area%
9.598	89.00431	11658731	703737	0.000000		9.163	10.389	89.0043
19.675	10.99569	1440333	41916	0.000000		19.083	20.608	10.9957

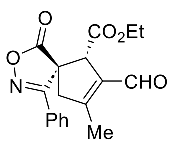

3n

Column: IB column
mobile phase: *n*-heptane / propan-2-ol= 70:30
λ= 254 nm, *V*= 1 ml/min, *t*= 25 °C
t_R= 12.9 min (minor), t_R= 15.4 min (major), ee= 76 %

minor diastereoisomer

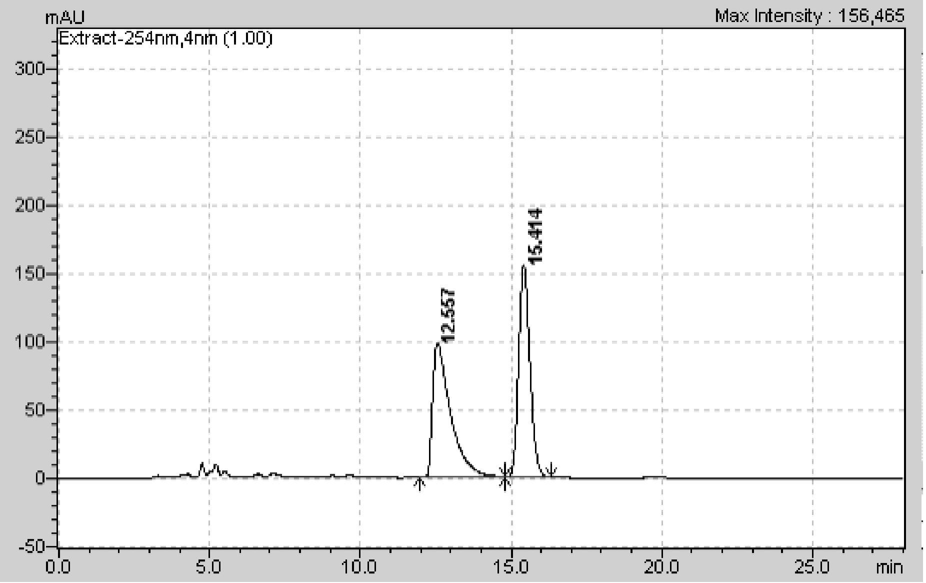

Ret. Time	Conc.	Area	Height	Similarity Inde	Mar	Peak Start	Peak En	Area%
12.557	50.12172	4119446	98066	0.000000		11.979	14.827	50.1217
15.414	49.87828	4099438	156142	0.000000	V	14.827	16.352	49.8783

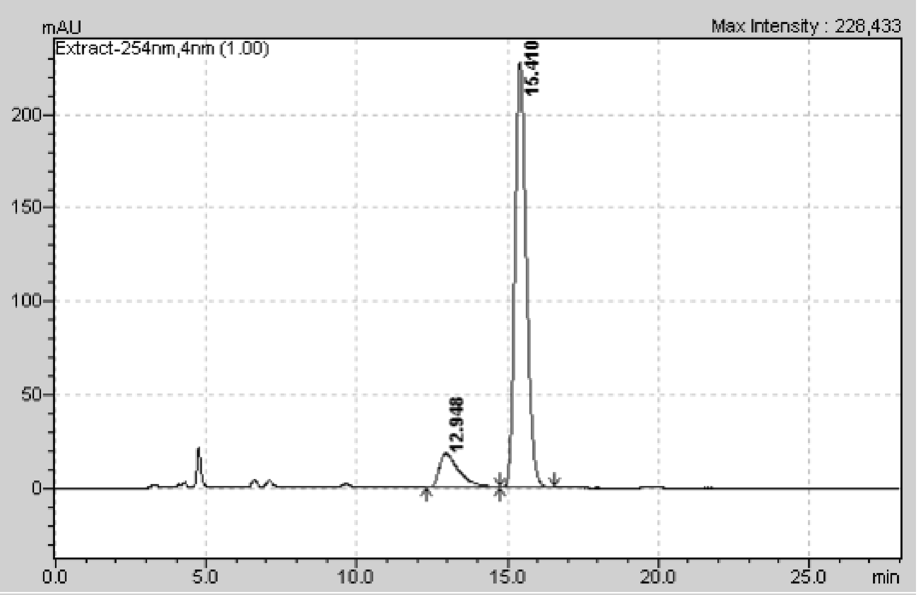

Ret. Time	Conc.	Area	Height	Similarity Inde	Mar	Peak Start	Peak En	Area%
12.948	12.28306	838474	18176	0.000000		12.288	14.752	12.2831
15.410	87.71694	5987793	227907	0.000000	V	14.752	16.544	87.7169

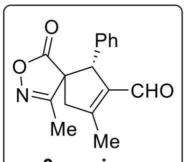

3o-major

Column: IA column
mobile phase: *n*-heptane / propan-2-ol= 90:10
λ= 233 nm, *V*= 1 ml/min, *t*= 25 °C
t_R= 15.7 min (major), t_R= 17.8 min (minor), ee= 88 %

major diastereoisomer

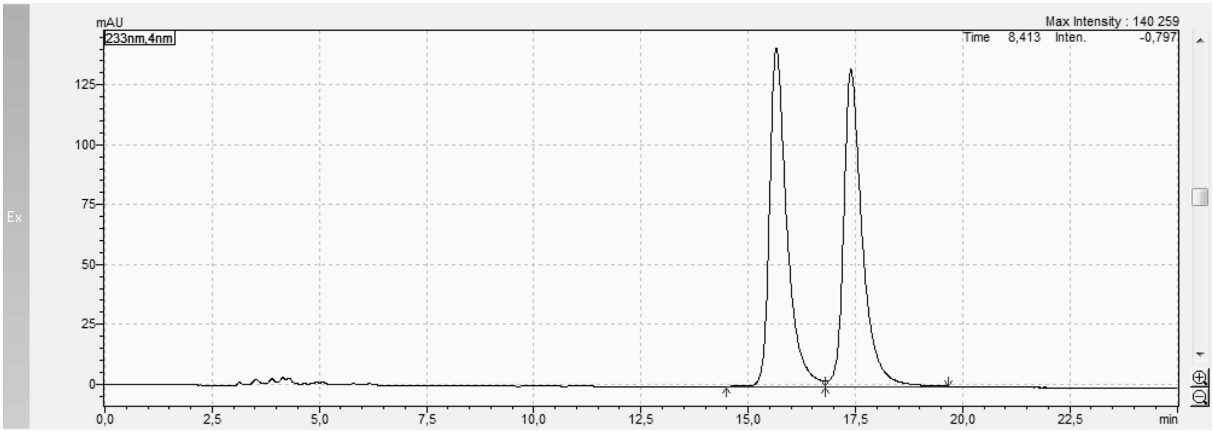

Results View - Peak Table

Peak Table | Compound | Group | Calibration Curve

Peak#	Ret. Time	Conc.	Area	Height	Similarity Index	Mark	Peak Start	Peak End	Area%
1	15,651	49,98599	3974142	141254	0,000000	M	14,485	16,811	49,986
2	17,392	50,01401	3976371	132615	0,000000	V M	16,811	19,669	50,014
Total		100,00000	7950513	273869					100,000

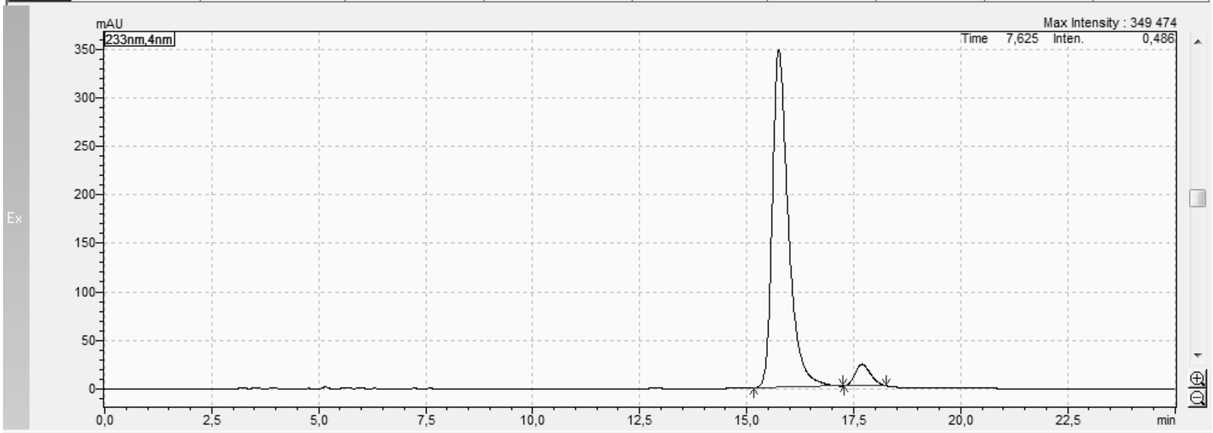

Results View - Peak Table

Peak Table | Compound | Group | Calibration Curve

Peak#	Ret. Time	Conc.	Area	Height	Similarity Index	Mark	Peak Start	Peak End	Area%
1	15,745	94,11480	9016449	347735	0,000000	M	15,168	17,259	94,115
2	17,688	5,88520	563818	22235	0,000000	M	17,269	18,261	5,885
Total		100,00000	9580267	369970					100,000

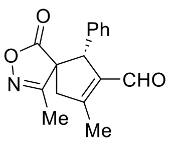

3o-minor

Column: IA column
mobile phase: *n*-heptane / propan-2-ol= 80:20
λ= 190 nm, V= 1 ml/min, t= 25 °C
t_R= 8.4 min (major), t_R= 9.3 min (minor), ee= 80 %

minor diastereoisomer

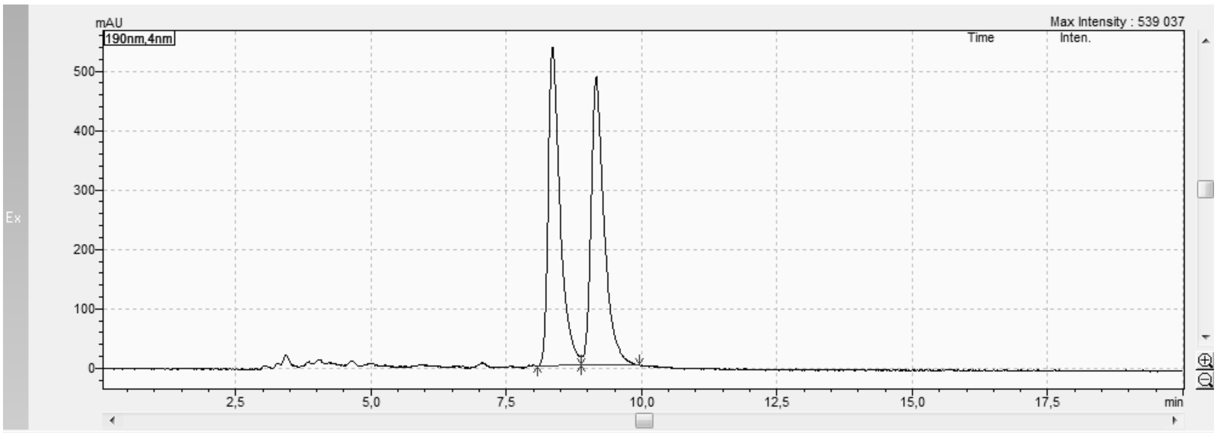

Results View - Peak Table

Peak Table | Compound | Group | Calibration Curve

Peak#	Ret. Time	Conc.	Area	Height	Similarity Index	Mark	Peak Start	Peak End	Area%
1	8,353	50,05665	8387765	548335	0,000000		8,085	8,885	50,057
2	9,155	49,94335	8368779	495772	0,000000	V	8,885	9,952	49,943
Total		100,00000	16756544	1044106					100,000

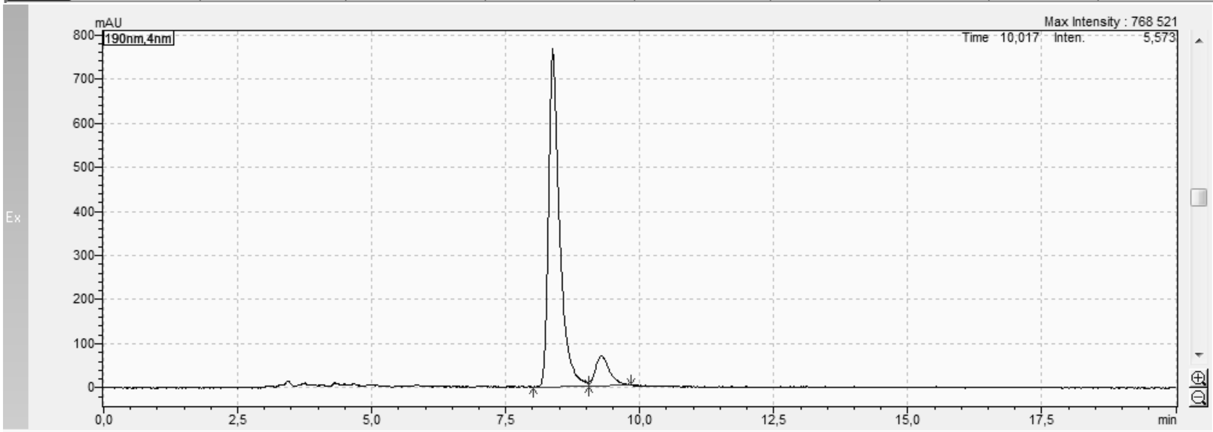

Results View - Peak Table

Peak Table | Compound | Group | Calibration Curve

Peak#	Ret. Time	Conc.	Area	Height	Similarity Index	Mark	Peak Start	Peak End	Area%
1	8,371	90,14043	11236067	792275	0,000000		8,011	9,045	90,140
2	9,278	9,85957	1229002	67241	0,000000	V	9,045	9,845	9,860
Total		100,00000	12465069	859516					100,000

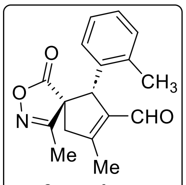

3p-major

Column: IA column
mobile phase: *n*-heptane / propan-2-ol= 95:5
λ= 190 nm, V= 1 ml/min, t= 25 °C
t_R= 20.3 min (minor), t_R= 22.0 min (major), ee= 82 %

major diastereoisomer

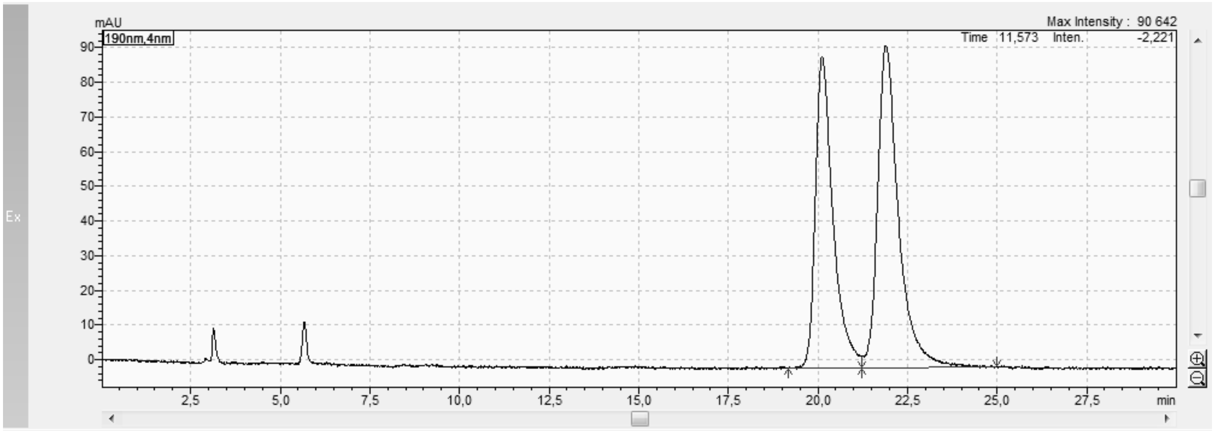

Results View - Peak Table

Peak Table | Compound | Group | Calibration Curve

Peak#	Ret. Time	Conc.	Area	Height	Similarity Index	Mark	Peak Start	Peak End	Area%
1	20,124	45,046	3076467	89574	0,000000	M	19,157	21,237	45,046
2	21,891	54,954	3753091	92816	0,000000	V M	21,237	24,992	54,954
Total		100,000	6829557	182389					100,000

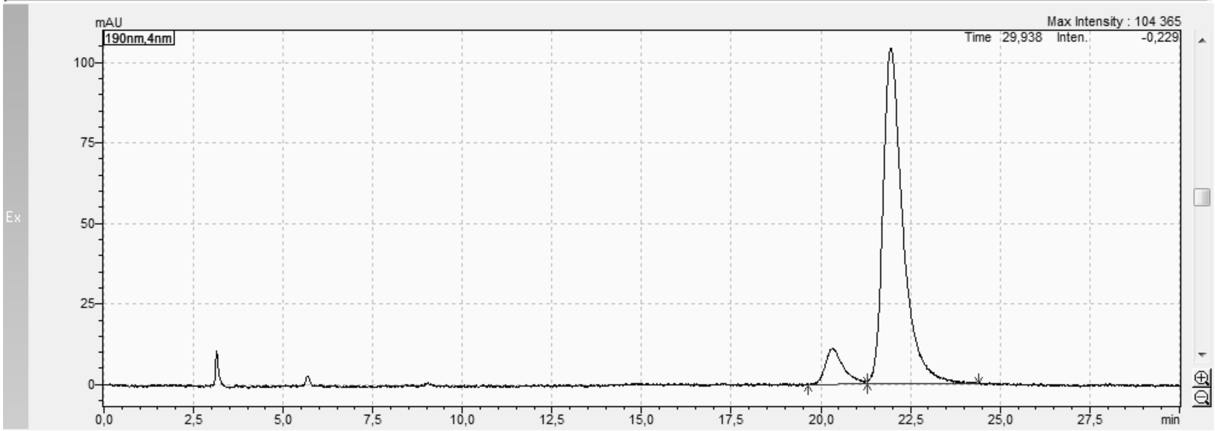

Results View - Peak Table

Peak Table | Compound | Group | Calibration Curve

Peak#	Ret. Time	Conc.	Area	Height	Similarity Index	Mark	Peak Start	Peak End	Area%
1	20,327	8,820	386141	11050	0,000000	M	19,648	21,291	8,820
2	21,955	91,180	3991702	104127	0,000000	V M	21,291	24,405	91,180
Total		100,000	4377843	115177					100,000

Column: IA column

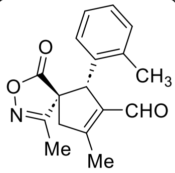

3p-minor

mobile phase: *n*-heptane / propan-2-ol= 97:3
λ= 223 nm, *V*= 1 ml/min, *t*= 25 °C
t_R= 26.7 min (major), t_R= 31.5 min (minor), ee= 69 %

minor diastereoisomer

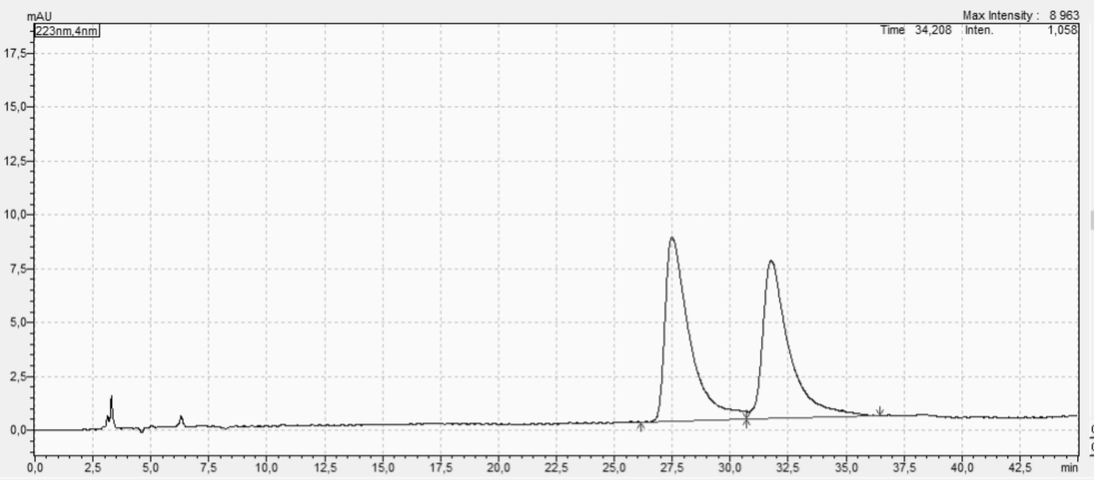

sults View - Peak Table

Compound Group Calibration Curve

Ret. Time	Area	Height	Peak Start	Peak End	Mark	Conc.	Unit	Area%
27,496	630390	8546	26,165	30,720	M	52,066		52,066
31,757	580363	7331	30,720	36,469	V M	47,934		47,934
	1210753	15877				100,000		100,000

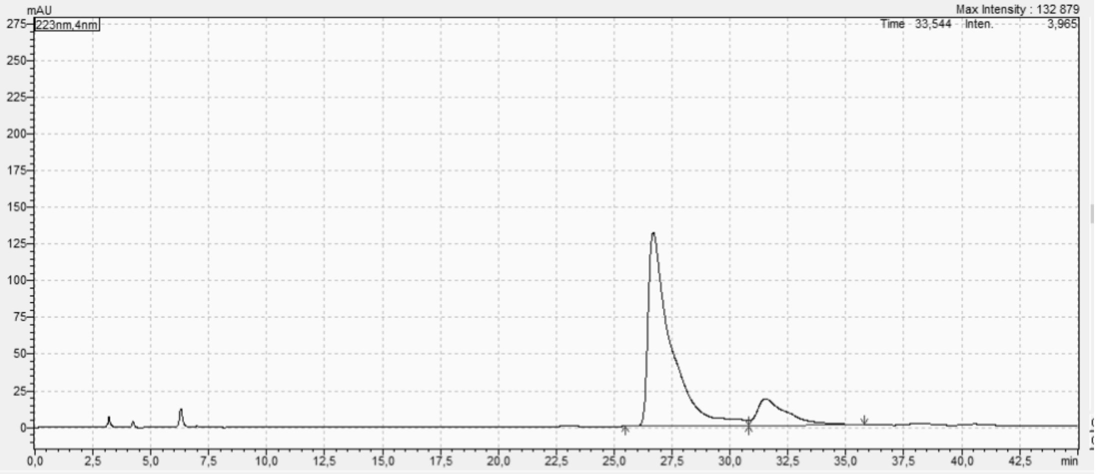

sults View - Peak Table

Compound Group Calibration Curve

Ret. Time	Area	Height	Peak Start	Peak End	Mark	Conc.	Unit	Area%
26,689	8961398	131934	25,472	30,816	M	84,453		84,453
31,532	1649703	18162	30,816	35,797	V M	15,547		15,547
	10611101	150096				100,000		100,000

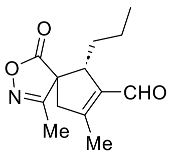

3q

Column: IB column
mobile phase: *n*-heptane / propan-2-ol= 95:5
λ= 202 nm, *V*= 1 ml/min, *t*= 25 °C
t_R= 21.1 min (major), t_R= 23.5 min (minor), ee= 77 %

major diastereoisomer

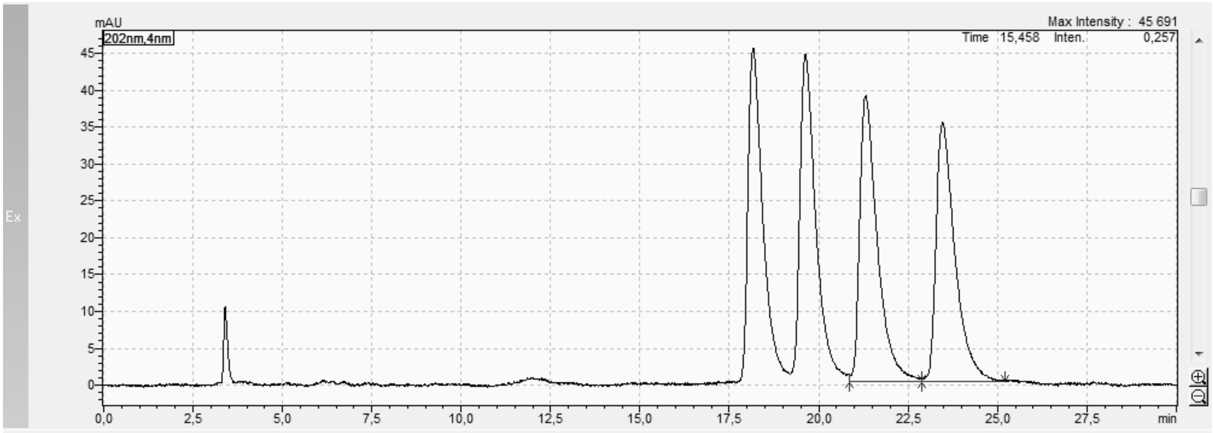

Results View - Peak Table

Peak Table | Compound | Group | Calibration Curve

Peak#	Ret. Time	Conc.	Area	Height	Similarity Index	Mark	Peak Start	Peak End	Area%
1	21,316	50,82337	1338926	38781	0,000000	M	20,875	22,891	50,823
2	23,462	49,17663	1295543	35275	0,000000	V M	22,891	25,205	49,177
Total		100,00000	2634469	74057					100,000

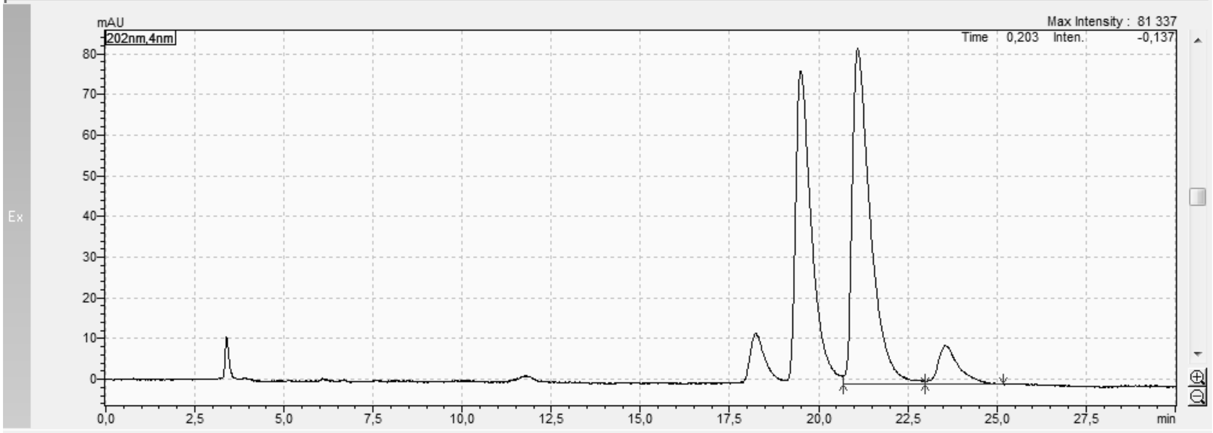

Results View - Peak Table

Peak Table | Compound | Group | Calibration Curve

Peak#	Ret. Time	Conc.	Area	Height	Similarity Index	Mark	Peak Start	Peak End	Area%
1	21,092	88,27648	2929958	82404	0,000000	M	20,693	22,987	88,276
2	23,539	11,72352	389112	9492	0,000000	V M	22,987	25,184	11,724
Total		100,00000	3319070	91896					100,000

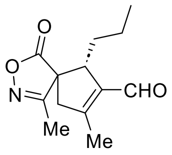

3q

Column: IB column
mobile phase: *n*-heptane / propan-2-ol= 95:5
λ= 202 nm, V= 1 ml/min, t= 25 °C
t_R= 18.2 min (minor), t_R= 19.4 min (major), ee= 74 %

minor diastereoisomer

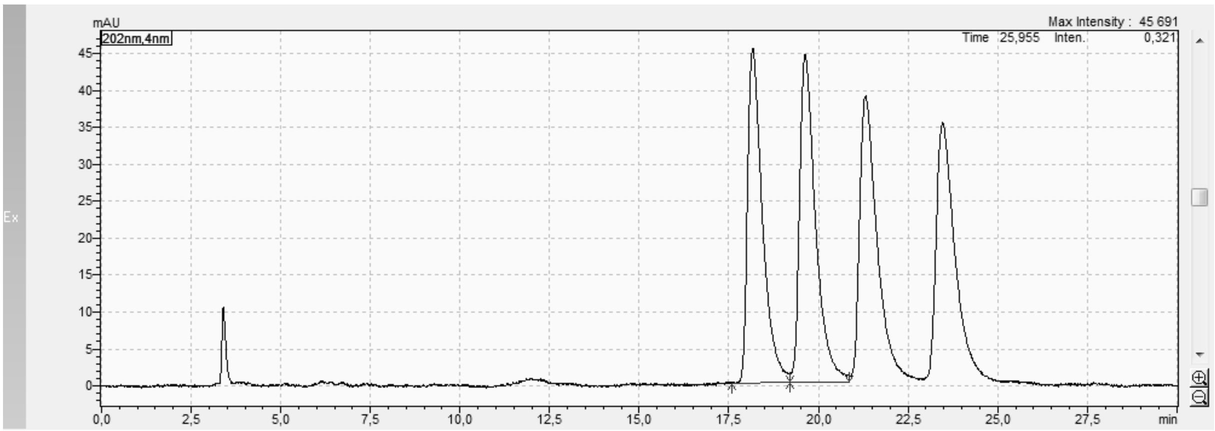

Results View - Peak Table

Peak Table | Compound | Group | Calibration Curve

Peak#	Ret. Time	Conc.	Area	Height	Similarity Index	Mark	Peak Start	Peak End	Area%
1	18,161	48,66875	1311861	45293	0,000000	M	17,568	19,189	48,669
2	19,616	51,33125	1383629	44461	0,000000	V M	19,189	20,875	51,331
Total		100,00000	2695490	89754					100,000

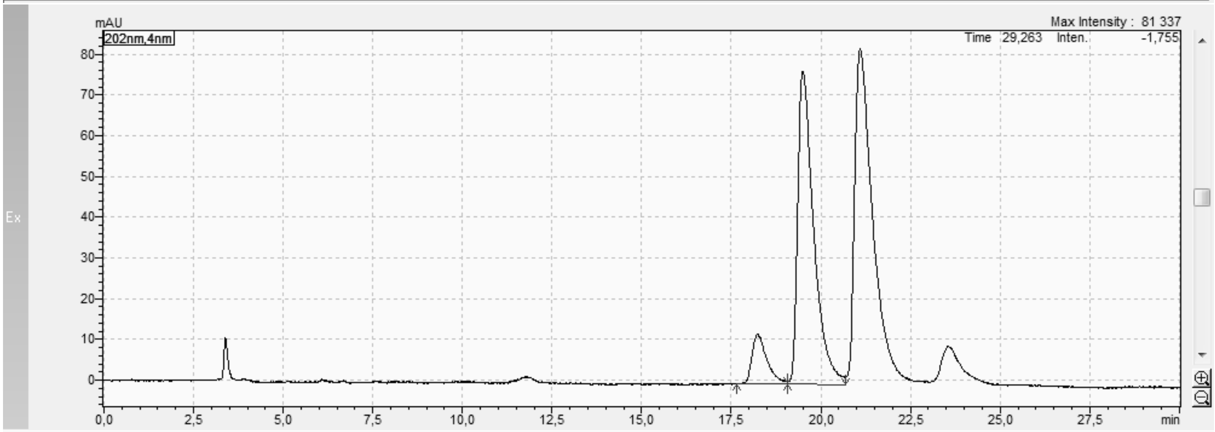

Results View - Peak Table

Peak Table | Compound | Group | Calibration Curve

Peak#	Ret. Time	Conc.	Area	Height	Similarity Index	Mark	Peak Start	Peak End	Area%
1	18,238	12,91303	353573	12185	0,000000	M	17,653	19,083	12,913
2	19,494	87,08697	2384534	76747	0,000000	V M	19,083	20,693	87,087
Total		100,00000	2738107	88932					100,000

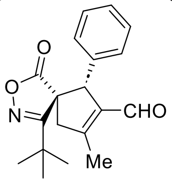

3r

Column: IC column
mobile phase: *n*-heptane / propan-2-ol= 80:20
λ= 210 nm, *V*= 1 ml/min, *t*= 25 °C
t_R= 13.8 min (major), t_R= 19.0 min (minor), ee= 94 %

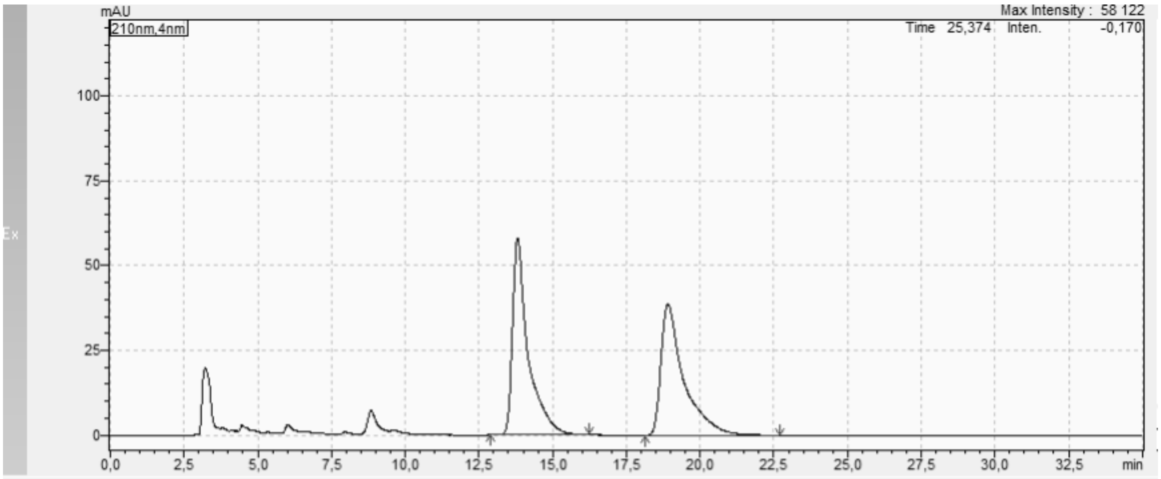

Results View - Peak Table

Peak Table | Compound | Group | Calibration Curve

Peak#	Ret. Time	Area	Height	Peak Start	Peak End	Mark	Conc.	Unit	Area%
1	13,810	2134979	57998	12,896	16,235	M	50,507		50,507
2	18,914	2092121	38682	18,133	22,709	M	49,493		49,493
Total		4227100	96680				100,000		100,000

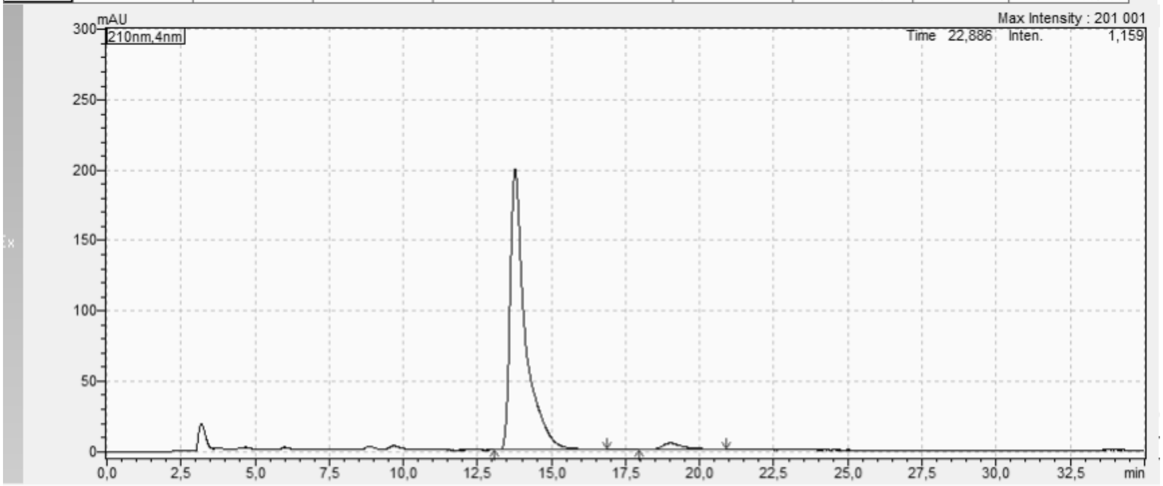

Results View - Peak Table

Peak Table | Compound | Group | Calibration Curve

Peak#	Ret. Time	Area	Height	Peak Start	Peak End	Mark	Conc.	Unit	Area%
1	13,773	7095689	199785	13,067	16,875	M	96,770		96,770
2	19,017	236848	4529	17,963	20,896	M	3,230		3,230
Total		7332537	204314				100,000		100,000

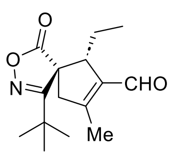

3s

Column: IB column
mobile phase: *n*-heptane / propan-2-ol= 95:5
λ= 240 nm, V= 1 ml/min, t= 25 °C
t_R= 11.8 min (major), t_R= 13.0 min (minor), ee= 85 %

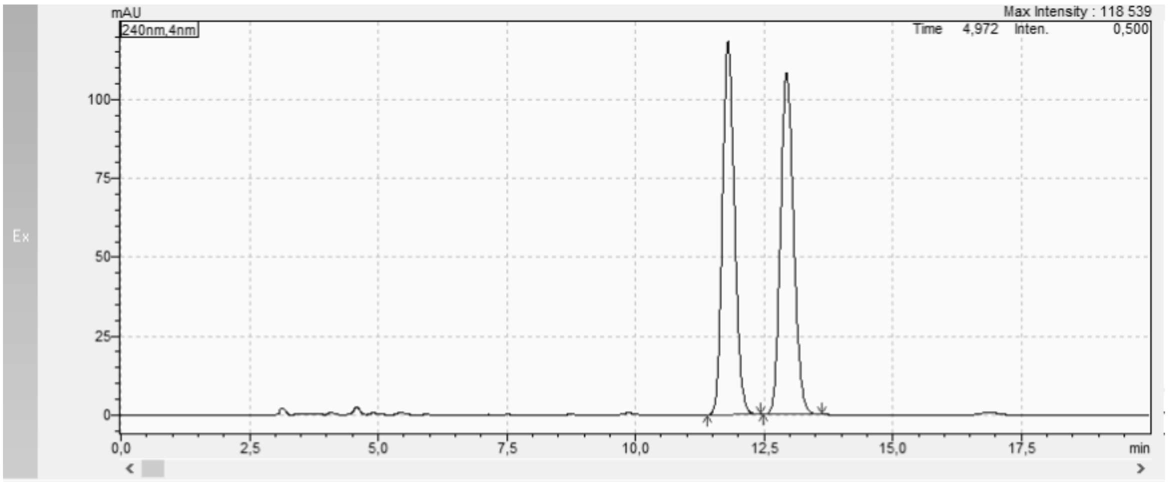

Results View - Peak Table

Peak Table | Compound | Group | Calibration Curve

Peak#	Ret. Time	Area	Height	Peak Start	Peak End	Mark	Conc.	Unit	Area%
1	11,797	1923924	118433	11,392	12,427	M	49,892		49,892
2	12,936	1932257	108384	12,480	13,621	M	50,108		50,108
Total		3856181	226817				100,000		100,000

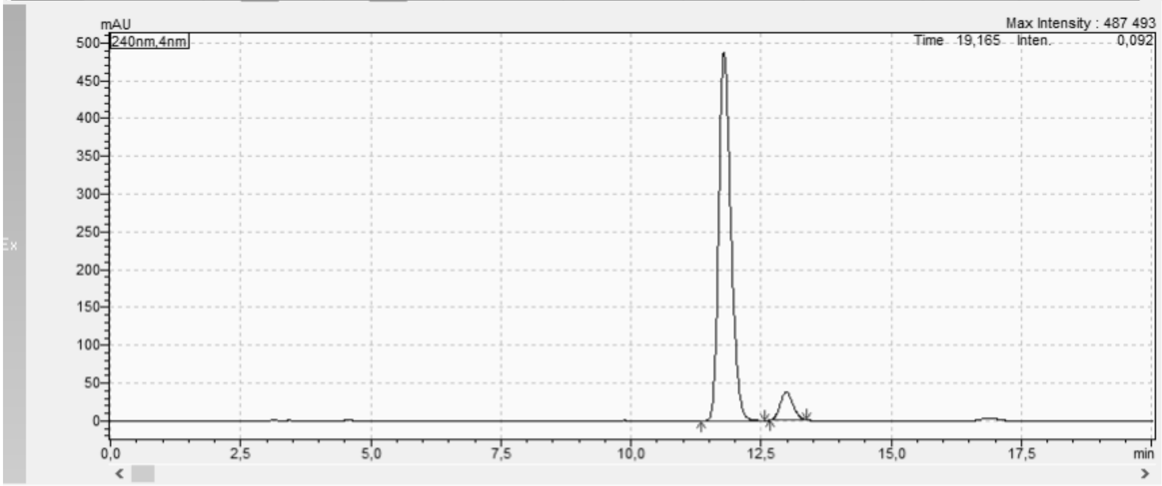

Results View - Peak Table

Peak Table | Compound | Group | Calibration Curve

Peak#	Ret. Time	Area	Height	Peak Start	Peak End	Mark	Conc.	Unit	Area%
1	11,787	7918529	487299	11,339	12,565		92,617		92,617
2	12,984	631212	37032	12,683	13,376	M	7,383		7,383
Total		8549741	524332				100,000		100,000

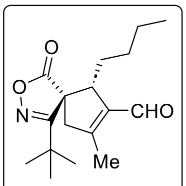

3t

Column: IB column
mobile phase: *n*-heptane / propan-2-ol= 98:2
λ= 240 nm, V= 1 ml/min, t= 25 °C
t_R= 13.1 min (major), t_R= 14.3 min (minor), ee= 90 %

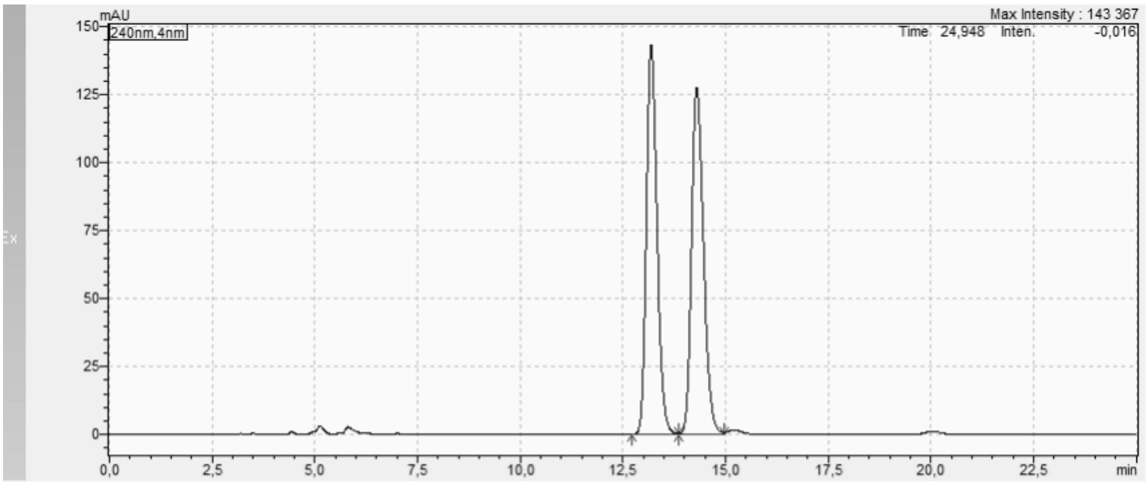

Results View - Peak Table

Peak Table | Compound | Group | Calibration Curve

Peak#	Ret. Time	Area	Height	Peak Start	Peak End	Mark	Conc.	Unit	Area%
1	13,187	2570394	143261	12,704	13,856		50,045		50,045
2	14,298	2565809	127537	13,856	14,965	V	49,955		49,955
Total		5136203	270797				100,000		100,000

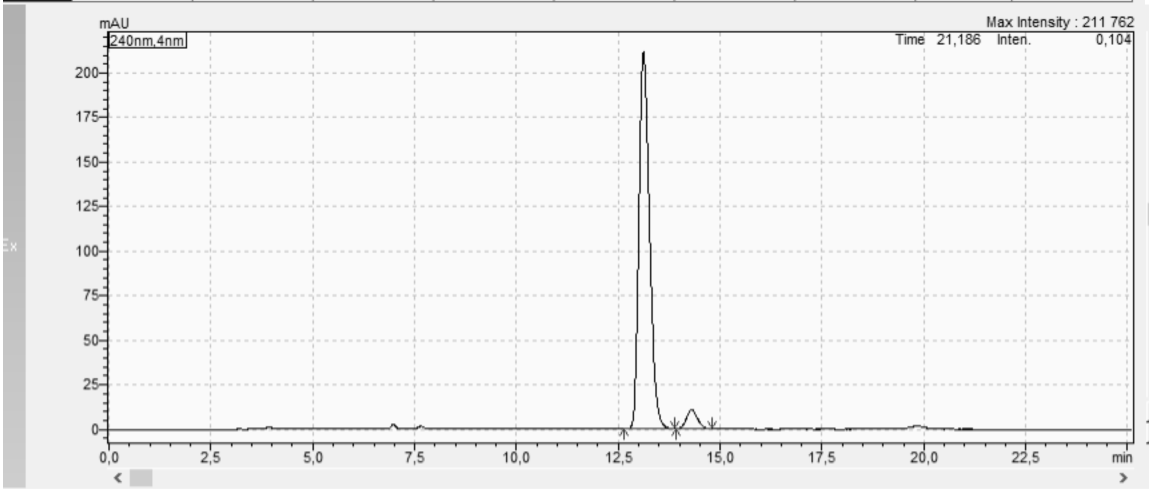

Results View - Peak Table

Peak Table | Compound | Group | Calibration Curve

Peak#	Ret. Time	Area	Height	Peak Start	Peak End	Mark	Conc.	Unit	Area%
1	13,118	3781935	211510	12,629	13,899	M	94,901		94,901
2	14,300	203213	10743	13,931	14,816	M	5,099		5,099
Total		3985149	222253				100,000		100,000

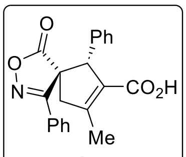

4

Column: IG column
mobile phase: *n*-heptane / propan-2-ol= 70:30
λ= 198 nm, *V*= 1 ml/min, *t*= 25 °C
t_R= 5.9 min (major), t_R= 9.0 min (minor), ee= 88 %

major diastereoisomer

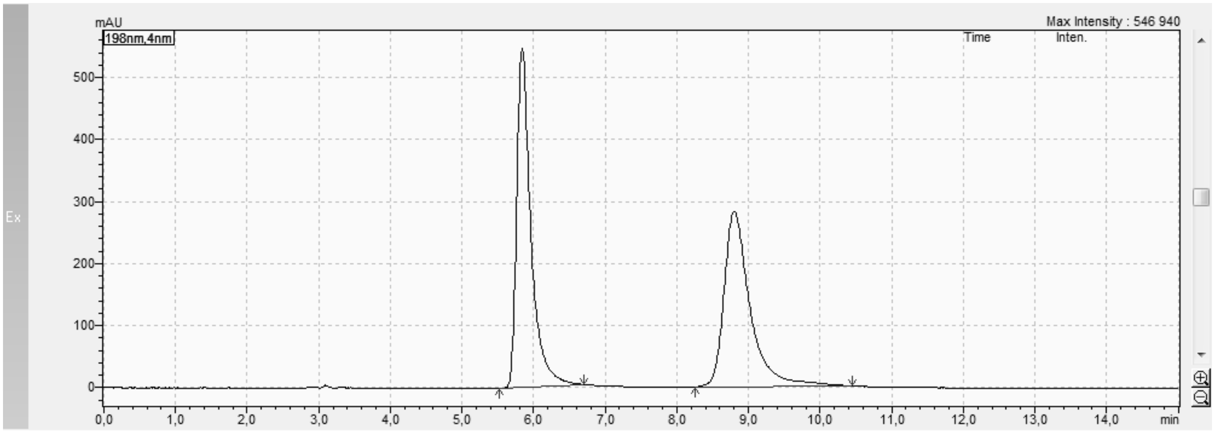

Results View - Peak Table

Peak Table | Compound | Group | Calibration Curve

Peak#	Ret. Time	Conc.	Area	Height	Similarity Index	Mark	Peak Start	Peak End	Area%
1	5,841	51,163	7511714	545792	0,000000	M	5,525	6,699	51,163
2	8,802	48,837	7170283	282678	0,000000	M	8,256	10,453	48,837
Total		100,000	14681997	828470					100,000

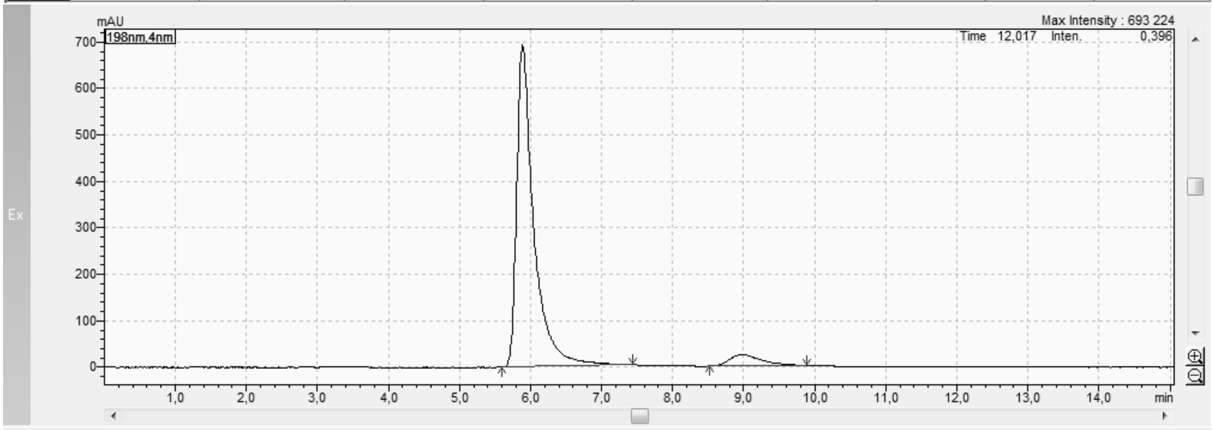

Results View - Peak Table

Peak Table | Compound | Group | Calibration Curve

Peak#	Ret. Time	Conc.	Area	Height	Similarity Index	Mark	Peak Start	Peak End	Area%
1	5,887	93,810	11561976	692362	0,000000	M	5,589	7,435	93,810
2	8,968	6,190	762891	24348	0,000000	M	8,512	9,888	6,190
Total		100,000	12324867	716710					100,000

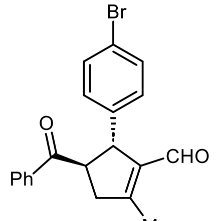

5

Column: IA column
mobile phase: *n*-heptane / propan-2-ol= 95:5
λ= 190 nm, V= 1 ml/min, t= 25 °C
t_R= 19.8 min (minor), t_R= 21.3 min (major), ee= 84 %

one diastereoisomer

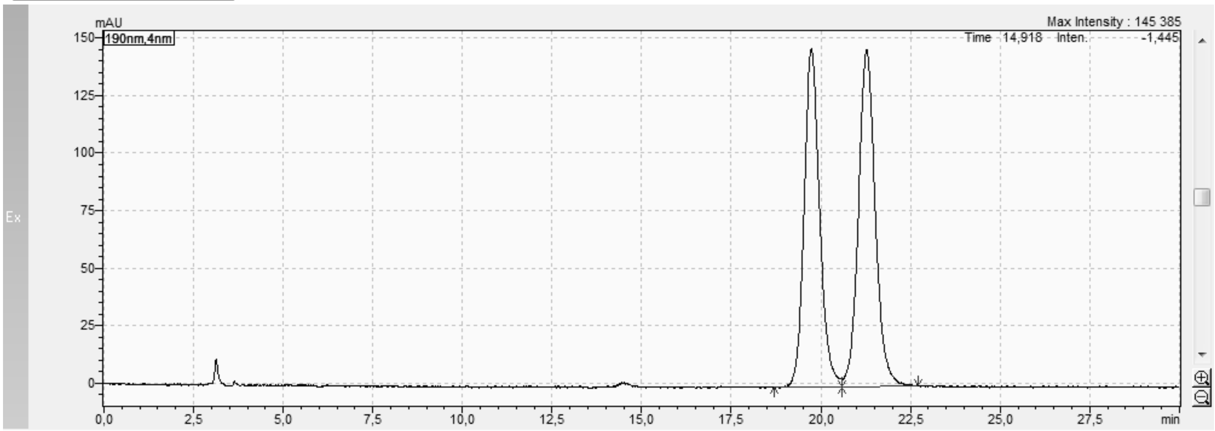

Results View - Peak Table

Peak Table | Compound | Group | Calibration Curve

Peak#	Ret. Time	Conc.	Area	Height	Similarity Index	Mark	Peak Start	Peak End	Area%
1	19,734	47,975	4403988	146944	0,000000	M	18,688	20,587	47,975
2	21,278	52,025	4775819	146320	0,000000	V M	20,587	22,699	52,025
Total		100,000	9179807	293264					100,000

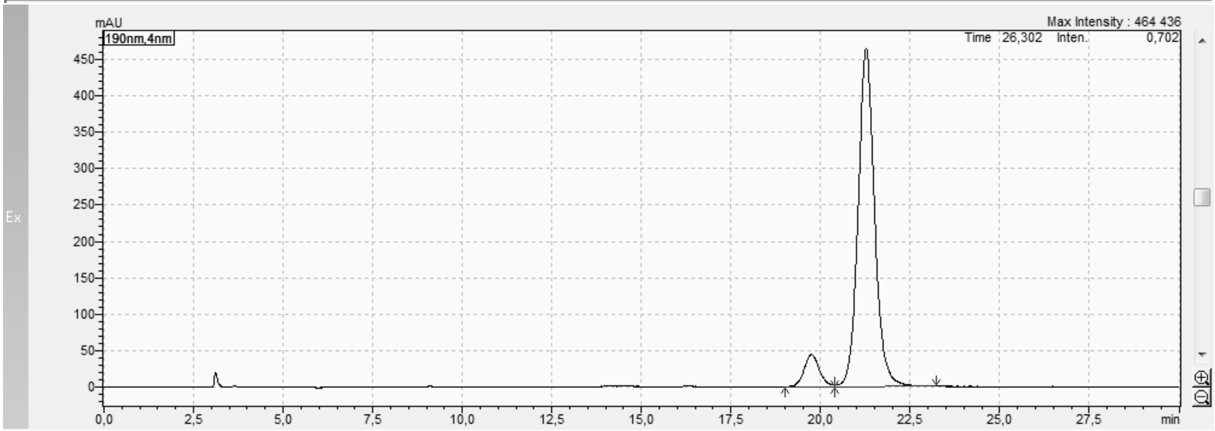

Results View - Peak Table

Peak Table | Compound | Group | Calibration Curve

Peak#	Ret. Time	Conc.	Area	Height	Similarity Index	Mark	Peak Start	Peak End	Area%
1	19,753	8,177	1321405	44220	0,000000	M	18,997	20,395	8,177
2	21,273	91,823	14838721	463406	0,000000	V M	20,395	23,243	91,823
Total		100,000	16160126	507626					100,000

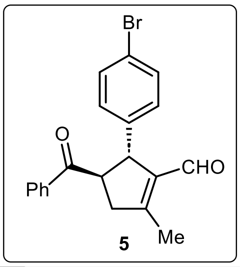

Column: IA column
mobile phase: *n*-heptane / propan-2-ol= 95:5
λ= 190 nm, *V*= 1 ml/min, *t*= 25 °C
t_R= 19.8 min (minor), t_R= 21.5 min (major), ee= 84 %

ONE POT one diastereoisomer

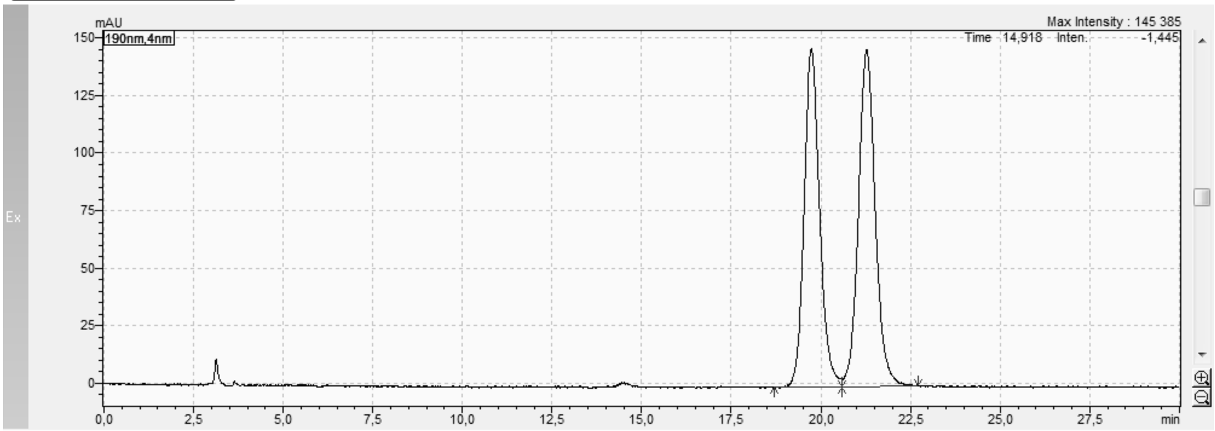

Results View - Peak Table

Peak Table | Compound | Group | Calibration Curve

Peak#	Ret. Time	Conc.	Area	Height	Similarity Index	Mark	Peak Start	Peak End	Area%
1	19,734	47,975	4403988	146944	0,000000	M	18,688	20,587	47,975
2	21,278	52,025	4775819	146320	0,000000	V M	20,587	22,699	52,025
Total		100,000	9179807	293264					100,000

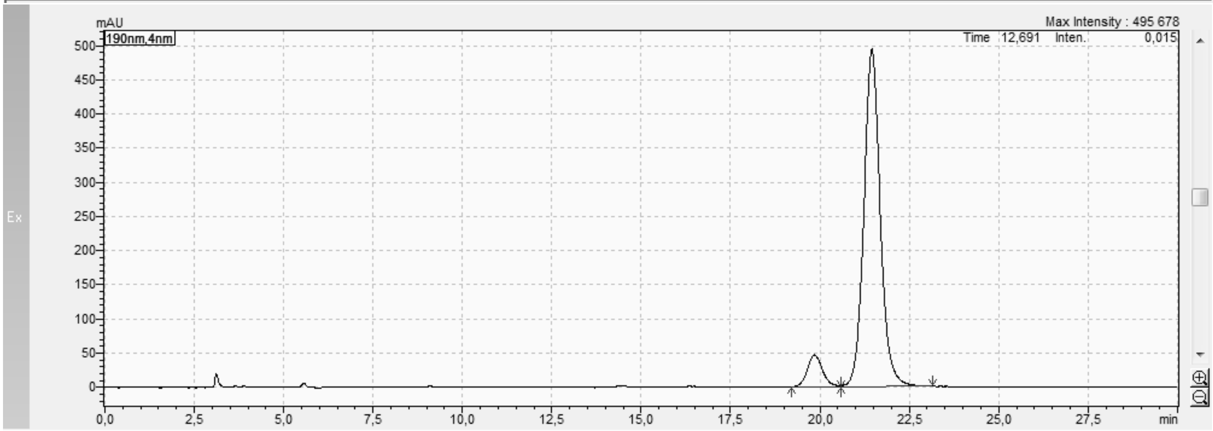

Results View - Peak Table

Peak Table | Compound | Group | Calibration Curve

Peak#	Ret. Time	Conc.	Area	Height	Similarity Index	Mark	Peak Start	Peak End	Area%
1	19,845	8,161	1397219	47154	0,000000	M	19,200	20,597	8,161
2	21,452	91,839	15722561	494553	0,000000	V M	20,597	23,168	91,839
Total		100,000	17119781	541707					100,000

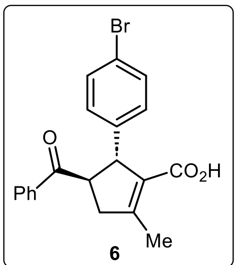

6

Column: IG column
mobile phase: *n*-heptane / propan-2-ol= 70:30
λ= 200 nm, V= 1 ml/min, t= 25 °C
t_R= 13.2 min (minor), t_R= 15.6 min (major), ee= 84 %

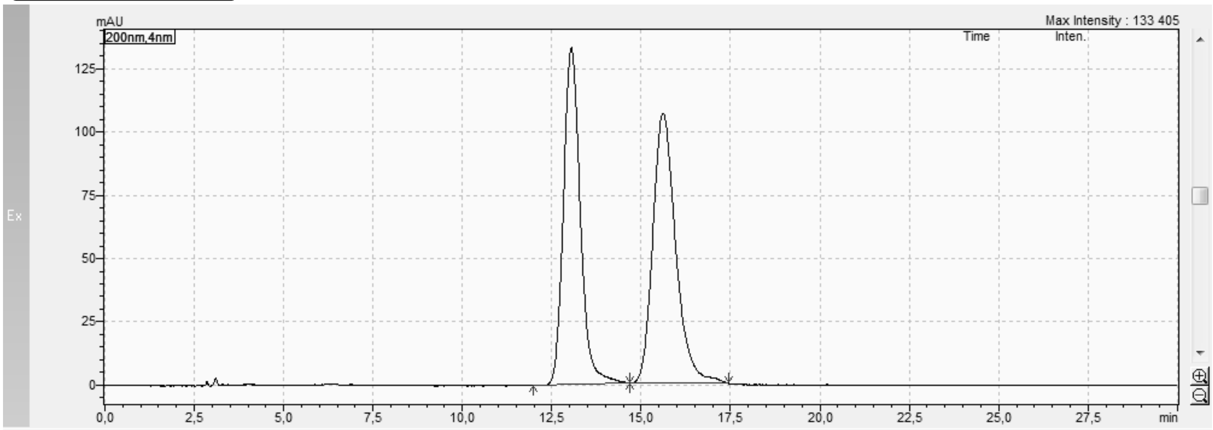

Results View - Peak Table

Peak Table | Compound | Group | Calibration Curve

Peak#	Ret. Time	Conc.	Area	Height	Similarity Index	Mark	Peak Start	Peak End	Area%
1	13,048	47,865	4358733	133094	0,000000	M	11,989	14,688	47,865
2	15,623	52,135	4747553	106546	0,000000	M	14,688	17,440	52,135
Total		100,000	9106286	239640					100,000

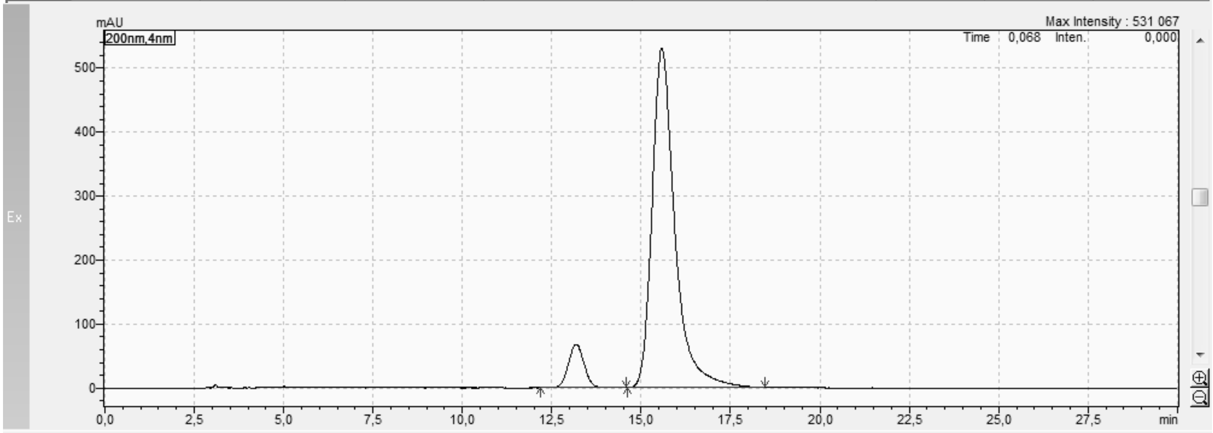

Results View - Peak Table

Peak Table | Compound | Group | Calibration Curve

Peak#	Ret. Time	Conc.	Area	Height	Similarity Index	Mark	Peak Start	Peak End	Area%
1	13,178	8,058	2052631	67804	0,000000	M	12,192	14,581	8,058
2	15,577	91,942	23420202	529908	0,000000	M	14,613	18,453	91,942
Total		100,000	25472834	597712					100,000
