# Supplementary figures and images for: Enantioselective Synthesis of Spirocyclic Isoxazolones Using a Conia-Ene Type Reaction
Source: J Org Chem. 2025 Mar 5;90(10):3615–27. doi: 10.1021/acs.joc.4c02921 (PMC11915384; doi:10.1021/acs.joc.4c02921)

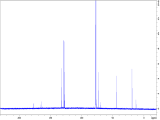

Supplement: Supplementary file 2 — jo4c02921_si_002.zip [file jo4c02921_si_002.zip › FIDs/1a/2/pdata/1/thumb.png]
